# Cerebellar deep brain stimulation for chronic post-stroke motor rehabilitation: a phase I trial

**DOI:** 10.1038/s41591-023-02507-0

**Published:** 2023-08-14

**Authors:** Kenneth B. Baker, Ela B. Plow, Sean Nagel, Anson B. Rosenfeldt, Raghavan Gopalakrishnan, Cynthia Clark, Alexandria Wyant, Madeleine Schroedel, John Ozinga, Sara Davidson, Olivia Hogue, Darlene Floden, Jacqueline Chen, Paul J. Ford, Lauren Sankary, Xuemei Huang, David A. Cunningham, Frank P. DiFilippo, Bo Hu, Stephen E. Jones, Francois Bethoux, Steven L. Wolf, John Chae, André G. Machado

**Affiliations:** 1 Department of Neurosciences, Lerner Research Institute, Cleveland Clinic, Cleveland, OH USA; 2 Center for Neurological Restoration, Neurological Institute, Cleveland Clinic, Cleveland, OH USA; 3 Department of Biomedical Engineering, Lerner Research Institute, Cleveland Clinic, Cleveland, OH USA; 4 Cerebrovascular Center, Neurological Institute, Cleveland Clinic, Cleveland, OH USA; 5 Physical Medicine and Rehabilitation, Neurological Institute, Cleveland Clinic, Cleveland, OH USA; 6 Department of Neurosurgery, Neurological Institute, Cleveland Clinic, Cleveland, OH USA; 7 Department of Quantitative Health Sciences, Lerner Research Institute, Cleveland Clinic, Cleveland, OH USA; 8 Department of Neurology, Neurological Institute, Cleveland Clinic, Cleveland, OH USA; 9 Department of Diagnostic Radiology, Imaging Institute, Cleveland Clinic, Cleveland, OH USA; 10 Neuroethics, Cleveland Clinic, Cleveland, OH USA; 11 Physical Medicine and Rehabilitation, Case Western Reserve University, Cleveland, OH USA; 12 Center for Rehabilitation Research, MetroHealth Systems, Cleveland, OH USA; 13 Cleveland FES Center, Cleveland, OH USA; 14 Department of Nuclear Medicine, Imaging Institute, Cleveland Clinic, Cleveland, OH USA; 15 Center for Movement Science and Physical Therapy, Division of Physical Therapy Education, Department of Rehabilitation Medicine, Emory University School of Medicine, Atlanta, GA USA

**Keywords:** Stroke, Medical research, Translational research

## Abstract

Upper-extremity impairment after stroke remains a major therapeutic challenge and a target of neuromodulation treatment efforts. In this open-label, non-randomized phase I trial, we applied deep brain stimulation to the cerebellar dentate nucleus combined with renewed physical rehabilitation to promote functional reorganization of ipsilesional cortex in 12 individuals with persistent (1–3 years), moderate-to-severe upper-extremity impairment. No serious perioperative or stimulation-related adverse events were encountered, with participants demonstrating a seven-point median improvement on the Upper-Extremity Fugl-Meyer Assessment. All individuals who enrolled with partial preservation of distal motor function exceeded minimal clinically important difference regardless of time since stroke, with a median improvement of 15 Upper-Extremity Fugl-Meyer Assessment points. These robust functional gains were directly correlated with cortical reorganization evidenced by increased ipsilesional metabolism. Our findings support the safety and feasibility of deep brain stimulation to the cerebellar dentate nucleus as a promising tool for modulation of late-stage neuroplasticity for functional recovery and the need for larger clinical trials. ClinicalTrials.gov registration: NCT02835443.

## Abstract (web summary)

A phase I trial of cerebellar deep brain stimulation to enhance chronic post-stroke motor rehabilitation supports the safety and feasibility of the approach while providing encouraging evidence of motor improvements.

## Main

Ischemic stroke can have devastating consequences to individuals and their families while simultaneously carrying a high social and economic burden. Major advances have been achieved in prevention and treatment of stroke by means of population-health management of risk factors and acute interventions. Innovation and development of new technologies have played a substantial role in improving outcomes in the early hours after insult, including advances in emergency healthcare delivery networks, imaging technologies and medical devices. The same cannot be said, however, for the post-acute phase where, despite substantial effort and investment, technological leaps have been slower. Even with contemporary techniques, up to 50% of survivors experience chronic disability^1^ after stroke and often require the assistance of others to complete activities of daily living.

Neuroplasticity is a well-documented phenomenon that is associated with gradual spontaneous or therapy-driven improvements in post-stroke motor function. The extent of recovery varies considerably across individuals and is known to depend largely on lesion location and size^2^, the timing of acute and post-acute interventions^3,4^, age^5^, genetic factors^6^ and preexisting comorbidities^7,8^. Harnessing the potential of neuroplasticity and modulating its extent and timing remains a major frontier in medicine with vast upside and has been the focus of our group. A wide range of neurostimulation-based treatment approaches aimed at modulating neuroplasticity and improving outcomes are currently under exploration at preclinical and clinical phases. These include targeting ipsilesional cortex directly using noninvasive techniques or surgically implanted electrode grids^9,10^ as well as efforts aimed at the peripheral nervous system that include the recently approved use of vagal nerve stimulation (VNS)^11^.

**Fig. 1:**
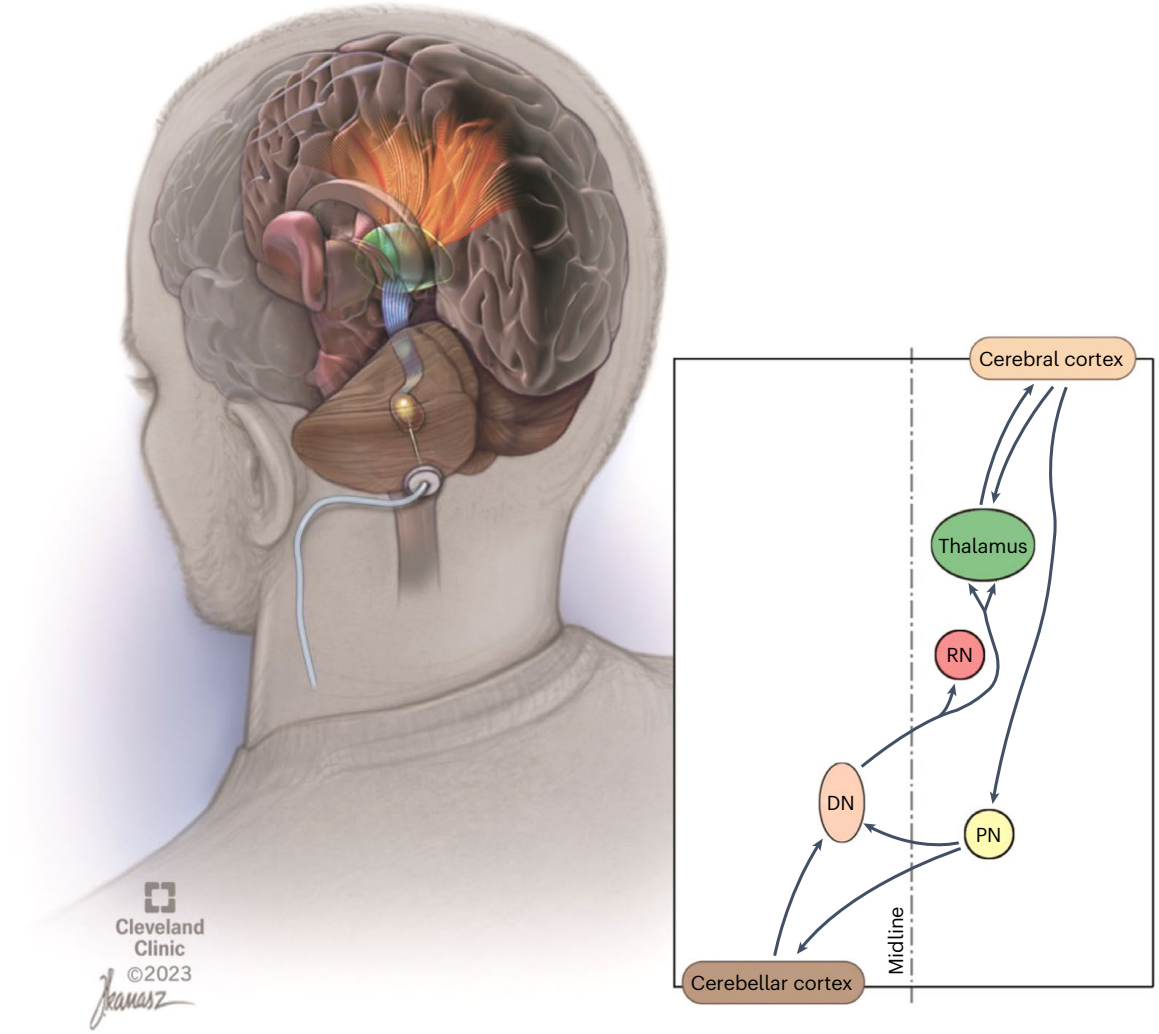
Illustrated overview of dentatothalamocortical pathway depicting a single deep brain stimulation lead implanted in the left dentate nucleus (brown). The crossed dentatothalamic projections (blue in upper-left illustration) terminate across multiple contralateral thalamic (green) nuclei that, in turn, project (orange), to broad regions of cerebral cortex. The dentatothalamocortical pathway represents the ascending component of a robust, reciprocal loop interconnecting the cerebral cortex with the contralateral cerebellar hemisphere. DN is shown in brown. RN, red nucleus; PN, pontine nuclei.

We have proposed and investigated a new, invasive surgical approach for extending the degree and temporal window of neuroplasticity after ischemic and traumatic insults to the brain. Specifically, the approach involves continuous stimulation of the cerebellar dentate nucleus (DN) to modulate neural activity and ipsilesional cortical excitability through activation of the robust, endogenous dentatothalamocortical pathway (Fig. 1)^12^. This central hypothesis is supported by extensive invasive and noninvasive electrophysiological investigation of the cerebellothalamocortical pathway over the past decades in the feline^13–16^, rodent^17^ and nonhuman primate^18^ models as well as in humans^19–21^. The crossed, reciprocal cerebello-cortical pathways have been shown to be highly relevant to motor function and post-stroke rehabilitation^22^, as originally evidenced by crossed-cerebellar diaschisis phenomenology and its influence on recovery of motor function^23^. Our prior work in preclinical rodent models of ischemia^24–27^, corroborated by subsequent, independent work^28^, supports our central hypothesis that DN deep brain stimulation (DBS) can promote recovery of function and ipsilesional cortical reorganization. Behavioral improvements were further associated with increments in ipsilesional cortical excitability, synaptogenesis, reorganization of motor representation of the affected limb, and greater expression of markers of long-term potentiation.

Here we report a first-in-human translation of this research in a group of 12 stroke survivors with persistent, moderate-to-severe upper-extremity impairment. In this open-label, phase I clinical trial, we evaluated the safety and feasibility of surgical implantation and chronic DBS of the cerebellar DN. Overall, we found that our surgical approach and chronic stimulation was feasible and well tolerated in the target population, with no study-related serious adverse events encountered across the trial. Additionally, participants underwent a battery of secondary assessments to begin to characterize and understand the effect of therapy on upper-extremity motor impairment and function. We report significant improvements in motor function across all individuals who, at the time of enrollment, had even minimal residual distal motor function in the affected upper extremity and correlated effects on cortical metabolism. Of particular interest to stroke survivors, the magnitude of benefit was not found to depend on time after stroke, with robust improvements observed in participants who enrolled as late as 3 years after their index event. These early findings support that this new neuromodulation-based approach is safe and feasible in a moderately-to-severely impaired post-stroke population and shows promise for promoting recovery of function and influencing neuroplastic processes after cerebral ischemia, even in the later stages of disability.

## Results

### Patient disposition

**Fig. 2:**
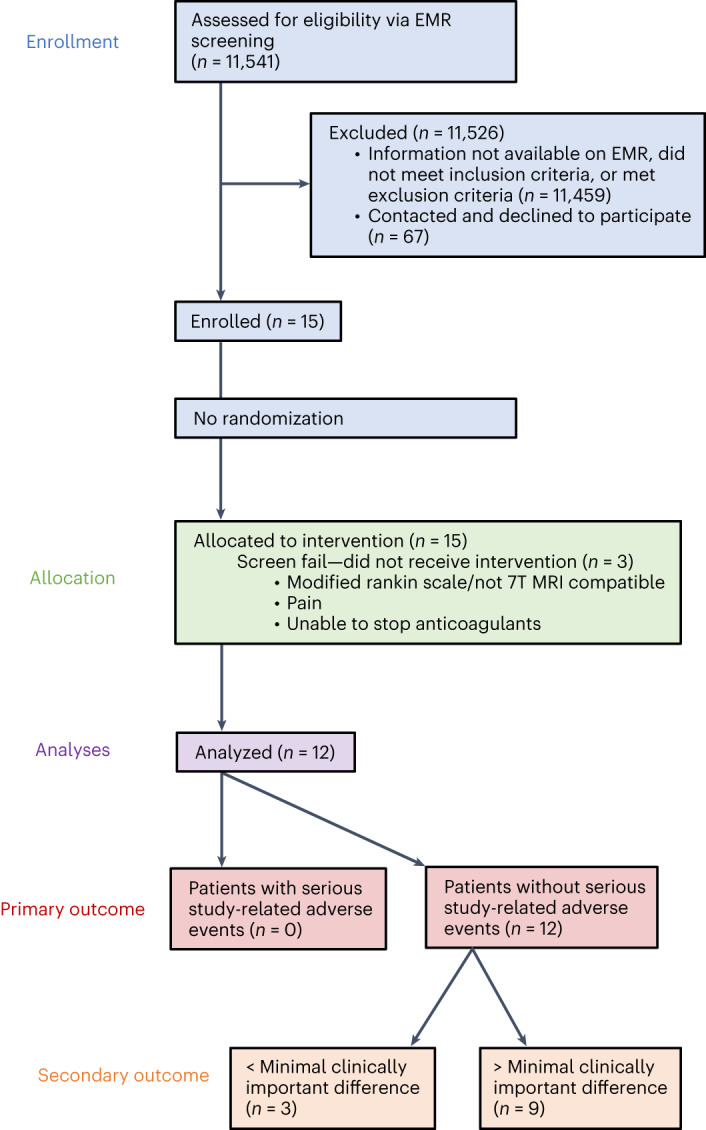
CONSORT diagram and outcomes for an open-label, single-arm phase I study of DBS to enhance post-stroke rehabilitation. EMR, electronic medical record.

**Fig. 3:**
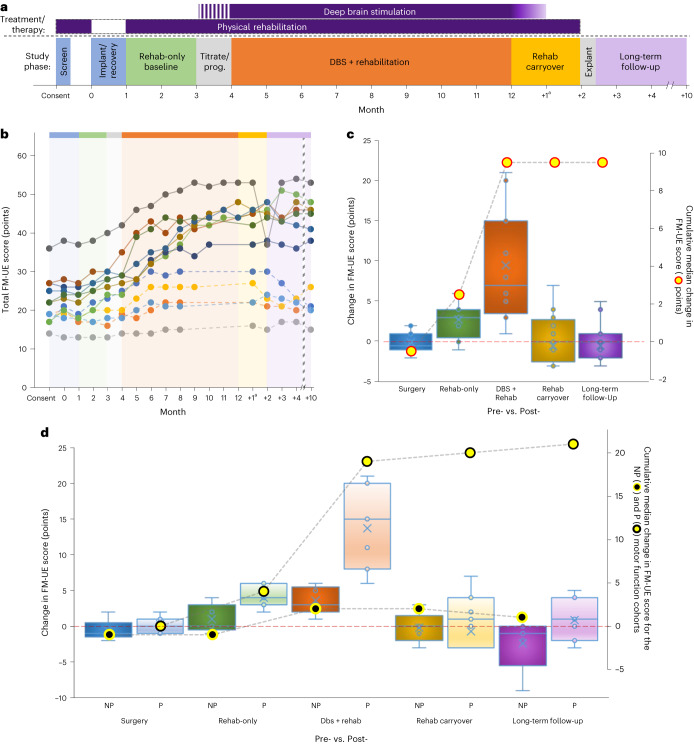
Trial overview and trial-related data for the FM-UE. **a**, Overall design of the phase I trial. Study phase color shading is used here and in Extended Data Fig. 1 and Supplementary Fig. 3. **b**, Individual FM-UE scores for each participant across the trial. The absence of a marker for a particular month (DBS + rehab phase) signifies that the participant met the criteria for DBS + rehab discontinuation and transitioned to the carryover phase. Dashed lines denote participants classified as non-preserved (NP) distal extremity motor function at enrollment. Note that the interconnecting lines are provided solely as a visual aid. **c**, Box-and-whisker plots representing the change scores (left *y* axis) for each of the surgical (month 1 minus 0), rehab-only (month 3 minus 1), DBS + rehab (month 8–12 (maximum achieved) minus 4), rehab carryover (month ‘+2’ minus month 8–12) and long-term follow-up (month ‘+10’ minus 8–12) trial phases. A score of zero signifies no change in impairment for that phase, with higher values reflecting improvement (that is, decreased impairment). The overlaid line plot represents the cumulative median change (right *y* axis) across the trial (*n* = 12 independent participants; two-sided Wilcoxon signed-rank test; *P = 0.004; **P < 0.001). **d**, Box-and-whisker change plots and cumulative line plots as a function of NP versus preserved (P) baseline distal extremity motor function. The overlaid line plots show the cumulative change (right *y* axis) observed across the trial (NP, black-filled, yellow circle; P: yellow-filled, black circle; *n* = 12 independent participants). For both **c** and **d**, boxes depict the median (horizontal line) within quartiles 1–3 (bounds of box). Whiskers extend to minimum and maximum values. Blue circles represent individual participant data points, while the blue ‘X’ depicts the average. ^a^As described in the text, the DBS + rehab phase was a minimum of 4 months but extended up to 8 months for participants who showed ongoing improvement. As such, the ‘+1’ time point reflects the measurement taken 1 month after DBS was turned OFF regardless of the DBS + rehab phase duration. The timing of all subsequent follow-up assessments was re-indexed to the final month of DBS + rehab.

Electronic medical record screening was used to identify individuals with a first-time, unilateral, ischemic stroke affecting the middle cerebral artery territory, sparing the diencephalon and basal ganglia, 12–36 months before surgery. Each candidate had to show persistent moderate-to-severe upper-extremity hemiparesis and sufficient upper-extremity motor ability to engage in rehabilitation (Methods) and meet inclusion and exclusion criteria (Supplementary Information and Fig. 2). A total of 11,541 individuals were assessed for eligibility, with 11,459 not having sufficient information on electronic medical records, failing to meet inclusion criteria or meeting exclusion criteria. Of the 82 individuals contacted regarding participation, 67 declined. As a result, a total of 15 individuals were enrolled, with 3 candidates subsequently classified as screen failures and exiting the study before surgery (Supplementary Information). The 12 remaining candidates participated in an open-label, non-randomized, single-arm trial, where individual participation spanned 20–24 months and included monthly assessments performed to record safety data and secondary metrics (Fig. 3a). Participant recruitment began on 1 June 2016. The first and last candidates for the trial were enrolled on 26 October 2016 and 6 August 2020, respectively. The final participant appointment for data collection was in November 2022. All patient appointments and data collection were performed at Cleveland Clinic Main Campus in Cleveland, Ohio. All participants underwent unilateral implantation of a single DBS lead in the contralesional DN (Supplementary Fig. 1) at the Cleveland Clinic between December 2016 and September 2020. The cohort had a mean age of 57.4 ± 6.5 years (range 48–70 years), time after stroke of 2.2 ± 0.7 years and an Upper-Extremity Fugl-Meyer Assessment (FM-UE) score of 22.9 (±6.2) points. Four participants were female and seven had dominant-side paresis. Participant demographics and clinical characteristics are provided in Table 1, a lesion probability map is provided in Supplementary Fig. 2, and final DBS settings are provided in the Supplementary Table 1.

**Table 1:** Participant demographics and clinical information

Participant	Age (year)^a^	Sex	Side of paresis	Dominant hand	Years after stroke^a^	Presurgical baseline			DBS + rehab
						FM-UE		AMAT	Duration (months)
						(Total)	(Hand)^b^		
01	58	F	L	R	1.6	17	3	2.4	8
02	58	F	L	R	1.2	17	1	1.2	4
03	53	M	R	R	2.4	19	1	1.5	4
04	51	M	L	R	1.6	14	1	1.1	4
05	59	F	L	R	2.6	19	1	1.9	5
07	57	M	R	R	1.8	19	2	1.6	4
08	70	F	R	R	1.9	27	4	3.7	6
10	58	M	L	L	2.9	27	6	3.6	5
11	48	M	R	R	1.7	36	4	2.9	8
13	54	M	L	R	2.0	22	4	3.0	8
14	54	M	L	L	3.4	25	2	2.7	8
15	69	M	L	L	3.2	22	5	3.1	5
Mean ± s.d.	57.4 ± 6.5				2.2 ± 0.7	22 ± 6	2.8 ± 1.7	2.4 ± 0.9	5.8 ± 1.8

^a^At implant

^b^FM-UE: part C sub-score

### Primary outcomes: safety and feasibility

The study accumulated 168 participant-months of DBS implant experience and 72 months of DN stimulation experience, with no device failures and no study-related, serious adverse events throughout the trial. A total of 51 adverse events were recorded during the trial, including 21 deemed related to study participation. Table 2 summarizes the incidence of adverse events during the study, while Extended Data Table 1 provides a summary of adverse events by participant. A complete listing of all adverse events by study phase is provided in Supplementary Table 2. Notably, there were no hemorrhages, infections, deaths or major perioperative complications. Transient effects of DN-DBS were noted during programming, where the objective was to identify the stimulation thresholds to side effects. All stimulation-related effects resolved with reprogramming.

**Table 2:** Incidence of adverse events during the trial

	Episodes	Participants
	*n*	*n* (%)
AE information		
Total AEs	51	12 (100)
Treatment-related AEs	21	9 (75)
Total SAEs	1	1 (8)
Treatment-related SAEs	0	0 (0)
AEs leading to withdrawal	0	0 (0)
Deaths	0	0 (0)
AEs by study phase		
Surgical	10	7 (58)
Rehab-only	4	4 (33)
Programming	12	7 (58)
DBS + rehab	7	5 (42)
Rehab carryover	5	5 (42)
Explant	4	3 (25)
Long-term follow-up	9	7 (58)

AE, adverse event; SAE, serious adverse event; rehab, rehabilitation.

### Secondary outcomes: motor impairment and function

Our evaluation of changes in motor impairment and function during the trial focused primarily on differences observed across five key intervals (Fig. 3a): (1) pre-surgery versus post-surgery (month 1 versus 0); (2) over the 2-month, rehab-only baseline period (no DBS: month 3 versus 1); (3) over the experimental, DBS + rehab phase (month 8–12 versus 4); (4) across the 2-month, rehab-carryover phase in the absence of DBS (month ‘+2’ versus month 8–12); and, finally, (5) at long-term follow-up, 10 months after termination of the experimental treatment phase (month ‘+10’ versus month 8–12). Figure 3b depicts each participant’s FM-UE score for each monthly visit. Those data are further summarized as change scores across each of the five phases of the study in Fig. 3c. Overall, we recorded a median decrease of 0.5 points (not significant) on the FM-UE between the pre-surgery and post-surgery time points for the full sample of participants, supporting the safety of the surgical implant procedure. Thereafter, we observed a modest three-point (*P* = 0.004) median improvement across the pre-stimulation, rehab-only phase followed by an additional seven-point improvement when rehabilitation was combined with DN-DBS (DBS + rehab phase; *P* = 0.0005). During the 2-month, rehab-carryover phase, which consisted of continued physical rehabilitation as DBS was weaned (weekly 25% amplitude reductions over the first month) and then OFF (second month), no further change in FM-UE was observed (median change in FM-UE = 0; not significant). Finally, the median FM-UE score for the full cohort again remained unchanged at the end of the long-term follow-up phase, supporting the durability of the previously realized, treatment-related gains.

**Extended Data Fig. 1:**
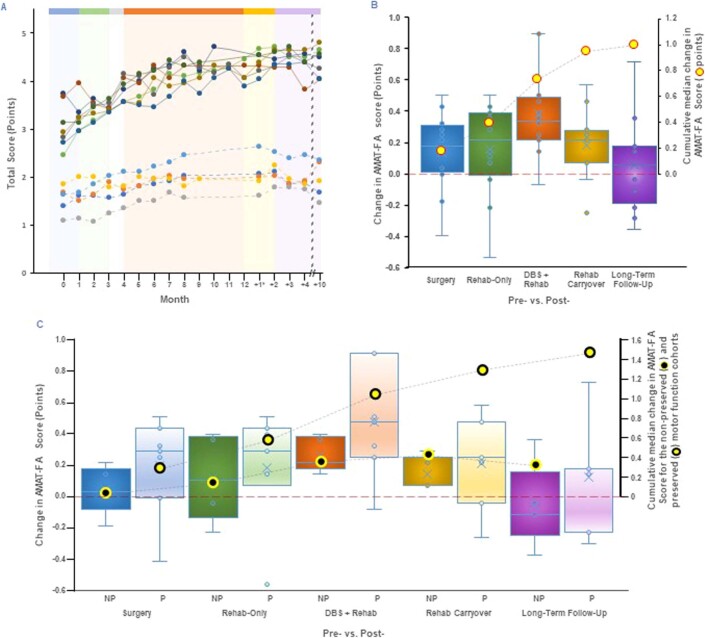
Changes in Participant AMAT-FA. **a**) Monthly AMAT Functional Ability scores depicted as in Fig. 3b. **b**) Box-and-whisker plots representing the phase-specific change scores for the AMAT Functional Ability subscale as per Fig. 3c. **c**) Change and cumulative line plots for the AMAT Functional Ability subscale separated by preservation of distal function at baseline as per Fig. 3d. Boxes depict the median (horizontal line) within quartile 1–3 (bounds of box). Whiskers extend to minimum and maximum values. Blue circles within each box-and-whisker plot represent individual participant data points, while the blue ‘x’ shows the average for each condition (n = 12 independent subjects; two-sided Wilcoxon signed rank test; *p = 0.016; **p < 0.001).

To learn if reductions in impairment would be associated with gains in function, given that both are important indicators of recovery, we also evaluated surgical and treatment-related changes using the Arm Motor Ability Test (AMAT). Individual scores for each study participant during the trial are presented for the functional ability (FA) subscale in Extended Data Fig. 1a, with data for quality of movement (QoM) provided in Supplementary Fig. 3a. The corresponding change scores are again provided for each study phase in Extended Data Fig. 1b and Supplementary Fig. 3b for FA and QoM, respectively. Of note, improvements in FM-UE were accompanied by significant improvements in both the FA (0.34 points; *P* = 0.0010) and QoM (0.45 points; *P* = 0.0005) subscales. The FA change was within the previously reported clinically important difference (CID) range (0.29 to 0.40) based upon patient perception^29^ and exceeded the 0.21 threshold used in a prior neurostimulation-based trial focused on upper-extremity rehabilitation^9^. A further modest, but significant, gain within the FA subscale was also noted following cessation of DBS during the rehab-carryover phase (0.21 points, *P* = 0.0161).

No significant treatment-related effects were observed across any of the remaining secondary metrics, including the Nine-Hole Peg Test, the Bilateral Box and Block Test, the Short Form Health Survey (SF-12), the EuroQol (EQ-5D), the Beck Depression Inventory or the Beck Anxiety Inventory. Detailed pretreatment/posttreatment scores are provided for the motor outcome metrics in Extended Data Table 2, with data analysis tables provided for each in the Supplementary Table 3.

### Effect of distal motor function preservation at enrollment

A post hoc analysis was performed to characterize the effect of level of preservation of distal motor function at enrollment on treatment-related changes in impairment and function. Preservation was defined as the presence, at screening, of active extension of the wrist and two digits plus active thumb abduction/extension, repeatable three times over a 1-min period, similar to what was applied successfully in the EXCITE and more recent VNS trials^11,30^. For the DBS + rehab phase, participants who entered the study with at least some distal preservation of motor function (P; *n* = 7) displayed a median gain of 15 points on the FM-UE over the DBS + rehab phase. This contrasted with a three-point gain for those who entered the study with no distal preservation (non-preserved) of motor function (*n* = 5; Fig. 3d; *P* = 0.007). A similar separation was observed on the AMAT, where participants in the preserved subgroup showed a change in AMAT (ΔAMAT)-FA of 0.46, while those in the non-preserved subgroup showed a ΔAMAT-FA of 0.21 (Extended Data Fig. 1c). Only the change for the preserved subgroup reached significance (*P* = 0.03) and exceeded the patient perception-based 0.29 to 0.40 CID range previously published^29^. A similar pattern was observed for the AMAT-QoM, with change scores of 0.46 (*P* = 0.03) and 0.36 (*P* = 0.06) observed for the preserved and non-preserved sub-cohorts, respectively (Supplementary Fig. 3c).

### Effect of time post-stroke on treatment-related gains

**Extended Data Fig. 2:**
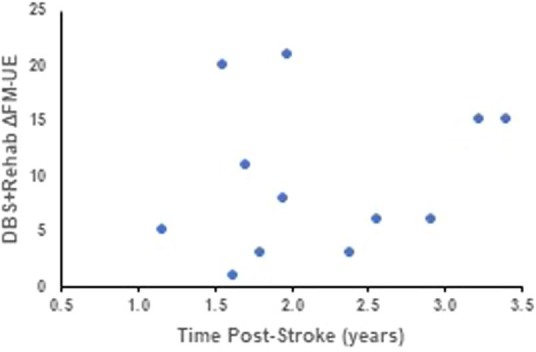
Participant Time post-stroke and DBS-related changes in FM-UE. Relationship between DBS-related change in FM-UE and time post-stroke for all participants. The data do not support an effect of time post-stroke on potential motor benefit.

As it is generally considered that there is an optimal period for facilitating post-stroke motor recovery, marked by a nonlinear process that begins to plateau by several months after injury^4,31^, we sought to determine whether the timing of DN-DBS treatment onset after stroke influenced the degree of improvement observed within our small sample. As summarized in Extended Data Fig. 2, we found no such relationship with FM-UE change scores across the DBS + rehab phase.

### Exploratory outcomes: brain metabolism

**Fig. 4:**
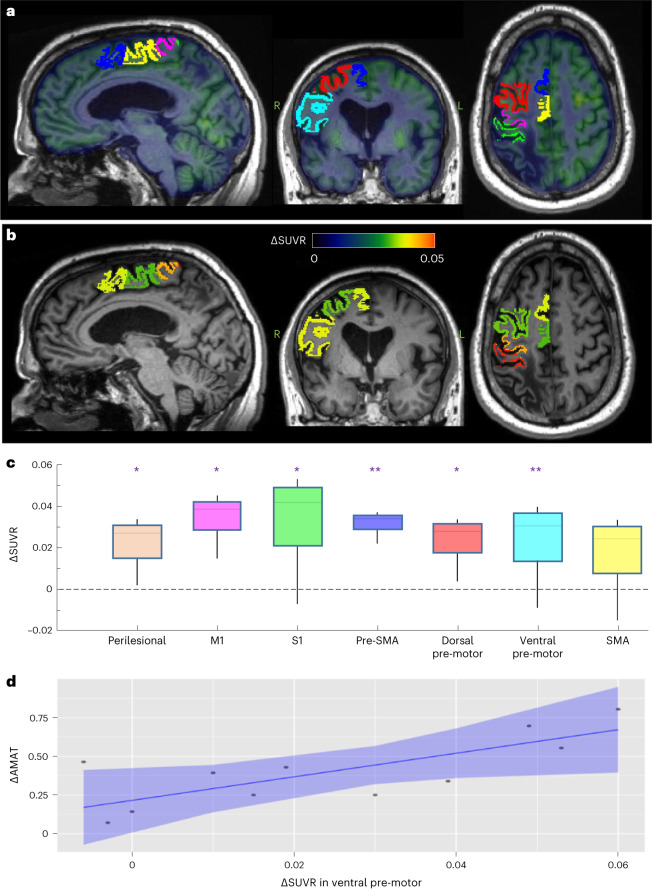
Change in brain metabolism associated with DN-DBS combined with rehabilitation and relationship to treatment-related changes in arm function. **a**, Magnetic resonance imaging (MRI) overlayed by PET from a 58-year-old male participant with a right hemisphere stroke lesion. The ipsilesional motor-associated cortical regions are identified as: primary motor (M1), fuchsia; primary somatosensory (S1), green; pre-supplementary area (pre-SMA), blue; dorsal pre-motor, red; ventral pre-motor, cyan; SMA, yellow. **b**, In the same participant shown in **a**, the average ΔSUVR adjusted for uptake time in response to DN-DBS combined with rehabilitation in the ipsilesional motor-associated cortical regions is shown. **c**, The change in brain metabolism in response to DN-DBS combined with rehabilitation in perilesional and ipsilesional motor-associated cortical regions for all participants. Linear mixed-effects models were used to test for significant change in the mean SUVR between rehab-only and rehab-carryover phases of the trial, with time point and ^18^F-FDG uptake time (that is, time between 18-FDG injection and start of PET scan) as fixed effects, with participant as the random effect intercept, and applying the default unstructured covariance structure. The box is the median and interquartile range, the whiskers are the maximum and minimum of the average ΔSUVR adjusted for uptake time, *n* = 11 participants. Two-sided *P* values with no adjustments for multiple comparison: perilesional *P* = 0.007, M1 *P* = 0.007, S1 *P* = 0.002, pre-SMA *P* = 0.0002, dorsal pre-motor *P* = 0.004, ventral pre-motor *P* = 0.0006, SMA *P* = 0.08. *P < 0.05, **P < 0.001. **d**, A significant association was identified between change in brain metabolism and change in arm function (as measured by ΔAMAT) in ipsilesional ventral pre-motor cortex. The line is the linear fit adjusting for uptake time and the error bands are the confidence intervals. *F*-statistic *P* = 0.04. Leave-one-out cross-validation: root mean squared error = 0.197; *R*^2^ = 0.247; mean absolute error = 0.176.

^18^F-fluorodeoxyglucose (FDG) positron-emission tomography/computed tomography (PET/CT) was used to characterize metabolic changes across ipsilateral perilesional cortex before and after the DBS + rehab phase, with comparisons made between data acquired toward the end of the rehab-only phase and again during the latter half of the DBS carryover phase. In both cases, data were collected with DBS turned OFF as our interest was in characterizing treatment-related metabolic effects in the absence of any potential confounding effect of ongoing stimulation delivery. As illustrated in Fig. 4, a comparison of the pre-DBS + rehab and post-DBS + rehab phase data revealed a significant increase in metabolism, after adjusting for uptake time, in perilesional cortex (average change in standardized uptake value ratio (SUVR), ΔSUVR = 0.026; standard error (s.e.) = 0.007; *P* = 0.007) and in five of the six ipsilesional motor-associated cortical areas (M1, ΔSUVR = 0.039, s.e. = 0.011, *P* = 0.007; S1, ΔSUVR = 0.042, s.e. = 0.009, *P* = 0.002; pre-SMA, ΔSUVR = 0.034, s.e. = 0.006, *P* = 0.0002; dorsal pre-motor, ΔSUVR = 0.028, s.e. = 0.007, *P* = 0.004; ventral pre-motor, ΔSUVR = 0.030, s.e. = 0.006, *P* = 0.0006). These changes were associated with a change in AMAT after adjusting for uptake time in the ventral pre-motor cortical region (Fig. 4c; β1 = 7.61, s.e. = 3.04, *P* = 0.04). No other ipsilesional motor-associated brain region showed a statistically significant association with a change in AMAT. None of the contralesional control occipital regions showed a significant change in metabolism over the treatment interval or change associated with change in AMAT after adjusting for uptake time. None of the ipsilesional motor-associated cortical regions or contralesional control occipital regions showed a significant association between change in metabolism over the treatment interval and change in FM-UE after adjusting for uptake time.

## Discussion

Data from this phase I trial of 12 individuals with chronic, moderate-to-severe post-stroke motor impairment support the safety and feasibility of chronic stimulation of the ascending cerebellothalamocortical pathway. Furthermore, it provides an in-human demonstration of significant and clinically meaningful effects of DN-DBS combined with rehabilitation on motor impairment^11,32^ and distal motor function^29^. This cohort with chronic, upper-extremity motor deficits was enrolled 1 to 3 years following their index middle cerebral artery infarction and underwent unilateral DBS targeting the contralesional cerebellar DN area without any major perioperative complications (for example, infection, hemorrhage or death). Although open-label, DN-DBS-related improvements on the FM-UE and AMAT met or exceeded established minimal CID thresholds. Finally, a post hoc analysis revealed that participants with preservation of some degree of distal motor function at enrollment showed significantly larger effects than those without such preservation.

Stroke remains the leading cause of physical disability in the industrialized world, with post-acute care costs exceeding those of acute hospital care^1^. As most survivors are left with substantial residual impairment despite contemporary rehabilitation efforts, there is growing interest in developing adjuvant, neurostimulation-based treatments to synergistically influence mechanisms underlying rehabilitation and improve long-term outcomes. While noninvasive approaches continue to be investigated, more recent efforts have begun to explore invasive, surgical alternatives, including direct cortical^9^ and VNS^11^. In contrast to current noninvasive technologies, these surgically implanted options can provide more continuous, at-home or demand-based, modulatory effects. One of the earliest efforts in this realm sought to enhance chronic post-stroke upper-extremity function through direct, epidural, electrical stimulation of perilesional cortex. Despite promising preclinical rodent and nonhuman primate as well as early clinical trial data, data from the phase III trial failed to meet its intended primary end point^9^. Reasons for this translational failure remain unclear but may in part be attributable to the overall integrity of the corticospinal pathways across participants^9^ or the limited capability of cortical surface stimulation to fully penetrate the gyrencephalic human brain^33^. Finally, and more recently, a randomized-controlled trial has shown that VNS was associated with improved outcomes compared to surgical controls, albeit with modest treatment effects^11^.

We observed significant and meaningful benefits in the form of both a reduction of motor impairment and gains in motor function in this cohort. It is noteworthy that these improvements were achieved: (1) in patients with overall poor function at enrollment, evidenced by a mean FM-UE of 22.9 points, and deemed to have poor natural prognosis and (2) after participants underwent a 3-month period of supervised rehabilitation aimed at minimizing the impact of renewed rehabilitation alone on impairment and function. In line with prior reports^9,34^, severity appeared to play a role in the degree of individual benefit achieved, as participants with at least minimally preserved distal motor function showed a robust, 15-point median improvement during the DBS + rehab phase. Lastly, we did not observe a correlation between time after stroke and motor improvements associated with DBS + rehab. As such, participants who were enrolled at 3 years after stroke still demonstrated effects of large magnitude, which counters the expectation that time after stroke might limit treatment-related benefit. These findings are important as they support the potential of DN-DBS to widen the therapeutic window of opportunity for stroke survivors. Overall, these observations provide encouragement for further investigation and valuable insight for refining the target population in the next, randomized-controlled, trial.

Given our prior animal model evidence that potentiation of corticomotor excitability and ipsilesional reorganization mediates the therapeutic effects of DN-DBS^24–27^, we applied PET as an exploratory approach to characterize treatment-related changes in cerebello-cortical physiology. In comparing pre-DBS + rehab versus post-DBS + rehab data, we observed significant group-level changes in perilesional metabolism, with subregional changes in metabolism in the majority of motor-associated cortices. In particular, changes in pre-DBS + rehab versus post-DBS + rehab PET data in ventral pre-motor cortex were found to be correlated with gains on the AMAT. Unlike FM-UE, which is a measure of gross motor impairment, AMAT is a measure of distal motor function and coordination and is therefore more physiologically dependent on the evolutionary specialization of motor cortices on distal control and finger movement^35^. Other ipsilesional motor-associated brain regions showed significant gains in pre-DBS + rehab versus post-DBS + rehab PET data that were not statistically significantly associated with functional changes. The lack of significant association between functional changes and metabolic changes in other motor areas may be consequent to effect variances in a small sample due to variabilities in stroke location or in damage along the dentatothalamocortical pathway. Overall, these findings are in line with our preclinical data and corroborate the effects of DN-DBS on ipsilesional cortical reorganization in late stages after stroke.

The study has limitations inherent to an early stage, Phase 1 investigation. Overall interpretation of the data presented is limited by the open-label nature of the design, the heterogeneity in baseline impairment level across the sample population, and, finally, the limited size of that same sample that are typical of a phase I neurological device trial. The latter two issues likely contributed to the lack of observed effects on several of the additional secondary measures applied, including the Nine-Hole Peg Test, the Bilateral Box and Block Test and the EQ-5D. Next, although we made some effort to overcome confounds related to chronic physical deconditioning before initiating DBS by including a rehabilitation-only phase, we are not able to distinguish, with certainty, the effects of rehabilitation from the effects of DBS. This is particularly the case given that structured rehabilitation has been shown to result in meaningful improvements even in chronic post-stroke stages^36^. Thus, while the magnitude of effects observed during the rehab+DBS phase are encouraging, only a subsequent, randomized-controlled trial will truly measure the contribution of DN-DBS to chronic recovery. To that end, all analyses of secondary measures should be considered exploratory, with the primary purpose of informing subsequent trials. The duration of treatment and improvement in function are intrinsically linked in our sample, as continued improvement was a prerequisite for additional time on treatment. Notably, no patients in the non-preserved group demonstrated a level of improvement to have treatment time extended. Finally, interpretation of the PET data presented were similarly limited by the small sample size, variability in stroke lesion location, and the lack of a control group.

In summary, this phase I study presents the first evidence of safety and feasibility of DN-DBS in individuals with chronic post-stroke hemiparesis with encouraging rehabilitative effects and associated neurophysiological gains observed across the DBS + rehab phase. This emerging intervention has shown translational potential to modulate the magnitude of neuroplastic reorganization toward recovery of function and to extend its time window to late phases of disability.

## Methods

### Patients and study design

Eligible individuals suffered a first-time, unilateral, ischemic stroke in the middle cerebral artery territory that spared the diencephalon and basal ganglia 12–36 months before surgery. Individuals with persistent moderate-to-severe upper-extremity hemiparesis as defined by an FM-UE score of ≤42 and sufficient upper-extremity motor ability to engage in rehabilitation (that is, a score of ≥1 on the FM-UE elbow flexion, elbow extension or finger mass flexion or extension) were included. Exclusion criteria included excessive spasticity or contracture of the upper-extremity muscles (that is, Modified Ashworth Scale = 4), severe cognitive impairment (Mini Mental State Examination < 24), as well as surgical, imaging or transcranial magnetic stimulation-related contraindications as detailed in the Supplementary Information. Participants also were required to possess criterion physiologic motor pathway response defined as image-guided, transcranial magnetic stimulation-elicited motor evoked potentials of ≥100 μV peak-to-peak size (in at least 6/10 trials) in partially contracted (15–25% of maximum voluntary contraction) paretic finger extensor digitorum communis or a proximal muscle (including forearm or wrist flexor muscles). Sex was not considered in the study design and sex-based analyses were not performed due to the small sample size.

Candidates were enrolled in an open-label, non-randomized, single-arm trial with a target enrollment of 12 individuals. Participation spanned 20–24 months, with monthly assessments performed to record safety data and secondary metrics (Fig. 3a). Following enrollment, participants underwent 1 month of upper-extremity rehabilitation twice per week to rule out potential for recovery with rehabilitation therapy alone. Thereafter, each participant underwent surgical implantation of the DBS system, with the lead implanted in the DN contralateral to the stroke-affected cerebral hemisphere in light of the predominantly crossed nature of this ascending cerebello-cortical projection. After a 1-month post-operative recovery without rehabilitation, twice-weekly physical rehabilitation was resumed for an additional 2 months (rehab-only phase). The enrollment of participants who had completed rehabilitation after stroke and the additional inclusion of 3 months of structured rehabilitation before activation of DBS was aimed at establishing a baseline before activating DBS and reducing confounds related to the effects of rehabilitation alone or exercise overcoming the effects of chronic deconditioning. Participants then entered the programming phase (4–10 weeks in tandem with a once-weekly therapy visit), during which the optimal stimulation parameters were determined. Once established, participants entered the DBS + rehab phase, where DBS was delivered continuously (that is, 24 h a day) for a minimum period of 4 months (maximum of 8 months) while participants continued twice-weekly sessions of in-clinic rehabilitation combined with an at-home program. At the end of the DBS + rehab phase, each participant entered the rehab-carryover phase and was weaned from stimulation, with pulse amplitude reduced in increments of 25% per week over 1 month followed by an additional month of continued rehabilitation therapy without stimulation. If treatment-related gains did not drop by more than 50% after stimulation was discontinued, participants underwent surgical explant of the DBS hardware followed by three long-term follow-up visits at 1, 2 and 6 months after explant.

The study protocol and subsequent amendments were approved by the Food and Drug Administration (investigational device no. G150237; 12/2015) with local approval established by the Institutional Review Board (IRB) of the Cleveland Clinic. Written informed consent was obtained before study-specific testing and re-consent to protocol changes was obtained when applicable. The informed consent process was actively monitored by two neuroethicists (P.F. and L.S.) and an independent Data Monitoring Committee (DMC). The DMC was composed of two neurosurgeons experienced in DBS and one physical medicine and rehabilitation physician. The DMC had the authority to halt the study in case of an unanticipated event or terminate the study in case of a second unanticipated event that did not have an acceptable etiology or resolution.

### Rehabilitation

The rehabilitation protocol was focused on highly repetitive, challenging and salient upper-extremity task practice administered by a physical therapist or assistant^37^. Sessions consisted of adaptive task practice, where the difficulty of the task was segmentally graded based on individual performance, and repetitive task practice, where a functional task was repeated continuously to reinforce successful performance^30^. The ratio of repetitive task practice to adaptive task practice alternated from 1:1 to 1:2 approximately every 4 weeks to maximize motor learning and standardize the rehabilitation dose between participants^38^. Tasks within the specified ratio were selected based on the level of motor impairment and collaborative goal setting between the participant and the therapist. Each session lasted 1–1.5 h, twice weekly and was supplemented with a targeted home exercise program of 3–5 exercises to be performed on days when formal therapy was not conducted. The number of sessions was reduced to one per week during the programming phase (Fig. 3a; month 3) for schedule accommodation as well as to minimize study fatigue given that DBS programming was being performed during separate, parallel sessions, up to two times per week. Rehabilitation sessions returned to twice weekly with supplemental home exercise thereafter (month 4) for the DBS + rehabilitation phase. Sessions were primarily conducted in person with the option of virtual rehabilitation sessions when travel was restricted, including coronavirus disease 2019-related disruptions.

### Deep brain stimulation

#### Surgical implantation and programming

Participants underwent stereotactic implantation of a single DBS lead in the area of the cerebellar DN contralateral to the lesioned cerebral hemisphere using a frame-based technique similar to that used in DBS for movement disorders (Supplementary Fig. 1)^39^. All participants received an 8-channel lead (Vercise or Vercise Cartesia, Boston Scientific) with electrode arrays up to 15.5 mm in length. Additional surgical details are provided in the Supplementary Information.

Between the rehab-only and the DBS + rehab phases, participants underwent up to 16 programming visits. First, a traditional monopolar review was performed to characterize stimulation-related side effects for each of the eight contacts of the DBS lead and delineate the upper limit of the parameter space. Thereafter, the acute effects of DN-DBS on motor task execution and task-related electroencephalography were examined according to specified combinations of electrode polarity, pulse frequency, pulse width and pulse amplitude.

#### DBS + rehab phase

At the end of programming, participants had their devices activated for a period of 4 to 8 months with continued rehabilitation. With a single exception, all had their device programmed for 30-Hz stimulation, consistent with our preclinical work^27,40^, with pulse amplitude adjusted individually based on side-effect thresholds and exploratory event-related electrophysiology. After a minimum of 4 months, DBS + rehab continued only if the participants demonstrated an average rate of change of >3.2 points as calculated across the prior 3 months^41^. The safety and efficacy data presented reflect the maximum duration of combined DN-DBS plus rehabilitation for each participant.

### Outcome measures

The primary end point was safety as measured by the incidence of serious adverse events during study participation. Among the secondary measures, the first motor outcome metric for characterization of the effects of the investigative treatment on upper-extremity impairment was the FM-UE, as it was the measure that determined the participant’s eligibility for the study under the inclusion and exclusion criteria. Additional secondary measures included indices of distal motor FA and quality of life as well as metabolic changes characterized by ^18^F-FDG PET/CT (Supplementary Table 4).

### Adverse events—primary outcome

Adverse events, including serious safety events, device-related events and unanticipated events were actively monitored in scheduled and unscheduled visits by the research team. The definitions for adverse events and serious adverse events followed the US Food and Drug Administration guidelines where adverse events were defined as any untoward medical occurrence, unintended disease or injury or any untoward clinical signs (including an abnormal laboratory finding) in participants, users or other persons whether or not related to the investigational medical device. Adverse events included all hospitalizations and events related to the investigational device or the comparator. This included events related to the procedures involved (any procedure in the clinical investigation plan). For users or other persons, this was restricted to events related to the investigational medical device. Adverse events did not include conditions preexisting to the participant’s enrollment. Preexisting conditions were not reported as adverse events unless the condition had an increased occurrence or intensity. Serious adverse events were defined as adverse events that (a) led to a death; (b) led to a serious deterioration in health that resulted in a life-threatening illness or injury, resulted in a permanent impairment of a body structure or a body function, required in-patient hospitalization or prolongation of existing hospitalization, resulted in medical or surgical intervention to prevent life-threatening illness or injury or permanent impairment to a body structure or a body function, or resulted in a substantial disruption in ability to conduct normal life functions; (c) led to fetal distress, fetal death or a congenital abnormality or birth defect; (d) when the event did not fit the above outcomes, but the event may have jeopardized the patient and may have required medical or surgical intervention (treatment) to prevent one of the other outcomes. It did not include in-patient hospitalization for a planned study procedure. Serious adverse events included device deficiencies that might have led to a serious adverse event if (a) suitable action had not been taken or (b) intervention had not been made. A planned hospitalization for preexisting conditions, or a procedure required by the Clinical Investigation Plan, without a serious deterioration in health or to prevent life-threatening illness or injury or permanent impairment to a body structure or a body function, was not considered to be a serious adverse event. Adverse events were reviewed by the principal investigator and DMC, and recorded in accordance with Good Clinical Practice and Food and Drug Administration Code of Federal Regulations for Medical Devices guidelines. Source documentation was provided to the study sponsors. Monthly checks of DBS hardware integrity were established by recording electrical impedance values.

### Efficacy assessment—unblinded

All efficacy assessments were performed, unblinded and in person, as secondary measures for this phase 1 trial. Data were collected monthly from the presurgical baseline through 4 months after the DBS + rehab phase (Fig. 3a: month 0 through ‘+4’) followed by a final, long-term follow-up assessment 10 months after the end of the DBS + rehab phase (Fig. 3a: ‘+10’ visit at 18–22 months after surgery). The variability in study timeline across participants was directly related to the duration of the individual participant’s DBS + rehab phase, which varied between 4 and 8 months depending upon the observed rate of recovery (see the summary of changes to protocol in the Supplementary Information). Secondary measures, including the FM-UE and AMAT, were included to offer estimates of treatment efficacy defined in the context of upper-extremity impairment and FA. The first motor outcome measure, used to determine inclusion/exclusion eligibility and then repeated throughout the study, was the FM-UE; a widely used, disease-specific impairment index designed to assess post-stroke hemiplegia recovery^42^. The total score was derived by summing its four subsections (A, upper extremity; B, wrist; C, hand; and D, coordination/speed (maximum total = 66 points)), with lower scores indicating greater impairment. Initial assessments were performed at screening and presurgical baseline. The threshold for minimal CID was established as five points, consistent with established standards^32^. The second motor outcome measure examined was the AMAT, which serves as a measure of distal motor function and coordination. Both the functional assessment and QoM subscales were scored. Combined, the FM-UE and AMAT are considered to provide a comprehensive view of motor behavior changes that are relevant to the post-stroke population.

Several other secondary measures were collected as part of this initial phase I exploration, including the Nine-Hole Peg Test, the Bilateral Box and Block Test, the Bimanual Grip Test, the Modified Ashworth Scale, the Short Form Health Survey (SF-12), the EQ-5D, the Beck Anxiety Inventory and the Beck Depression Inventory. These additional secondary metrics were included to monitor for any possible (then unknown) effects of DN-DBS on non-motor domains, including effects on spasticity, anxiety and depression; however, no significant changes were observed in this limited sample. A complete listing of secondary endpoints is provided in the Supplementary Table 4.

#### Brain imaging

Brain imaging included T1-weighted volumetric MRI of the head (Magnetom Prisma; Siemens Healthineers) performed before implantation of the DBS lead. FDG PET/CT studies were performed (Biograph TruePoint; Siemens Healthineers), adding physical therapy to the routine clinical protocol for cerebral metabolic imaging. After fasting at least 4 h, ^18^F-FDG was injected, followed by 30 min of hand physical therapy during the uptake period. PET data were acquired for 15 min, beginning with an average of 38 min (range 29–71 min) after injection. For one participant, the images acquired at the rehab-only phase had excessive motion artifact and were omitted from analyses involving this time point. For each participant, PET images were aligned with the MRI^43^ and stroke lesions were manually segmented on the MRI. Cortical segmentation was performed using FreeSurfer^44^. Perilesional cortex was defined as cortical tissue within a 10-mm dilation of the lesion using FSL^45^. The ipsilesional motor-associated cortical regions were defined using the Human Motor Area Template and consisted of primary motor, primary somatosensory, supplementary motor, pre-SMA, dorsal and ventral pre-motor regions^46^. Contralesional occipital cortex was not expected to be affected by dentate stimulation; therefore, we defined nine control cortical regions in the occipital lobe from the FreeSurfer segmentation (cuneus gyrus, middle occipital gyrus, superior occipital gyrus, occipital pole, calcarine sulcus, middle occipital and lunatus sulci, superior and transverse occipital sulci, anterior occipital sulcus, occipitotemporal and lateral occipital sulci). Mean SUVRs in the perilesional, ipsilesional motor-associated and control occipital cortical regions were calculated relative to the mean radioactivity of contralesional cortex, excluding the Human Motor Area Template regions. As we are reporting mean values for each region, small inaccuracies in the regional borders are not expected to have a meaningful impact on our interpretation.

### Statistical analysis

Change scores for each secondary outcome measure were calculated individually across five separate time periods: pre-surgery versus post-surgery (month 1 minus consent), pre-rehab-only versus post-rehab-only (month 3 minus month 1), pre-DBS + rehab versus post-DBS + rehab (month 8–12 minus month 4), pre-rehab carryover versus post-rehab carryover (month +2 minus month 8–12) and post-DBS + rehab to end of long-term follow-up (month ‘+10’ versus month 8–12). Due to skewed distributions, change scores are summarized using medians with interquartile ranges. Wilcoxon signed-rank tests were used to test for a significant change at each time period by comparing change scores for each phase to zero. Descriptive statistics with confidence intervals, for the full sample and for the sample stratified by functional preservation at baseline, are presented in the Supplementary Information. Tests were two tailed with significance indicated by *P* < 0.05 and carried out using SAS Studio v3.6. Sample size was based on safety. Analyses of secondary outcomes are considered preliminary and to be confirmed in future controlled trials. Thus, correction for multiple comparisons has not been made^47^.

Statistical analyses of PET data focused on the effect of therapy and its correlation with change in arm function per the AMAT (version 13). Linear mixed-effects models^48^ were used to test for significant change in the mean SUVR of perilesional and ipsilesional, motor-associated and contralesional occipital (control) cortical regions between rehab-only and rehab-carryover phases of the trial, with time point and ^18^F-FDG uptake time (that is, time between 18-FDG injection and start of PET scan) as fixed effects, participant as the random effect intercept, and applying the default unstructured covariance structure. Normality of model residuals and random effect intercept were evaluated using the Shapiro–Wilk normality test. Model fit was evaluated using Bayes information criterion and visual examination of residuals. Linear models^49^ using change in ^18^F-FDG uptake time as a covariate were used to test for a significant association between change in AMAT and change in the mean SUVR of perilesional, and ipsilesional motor-associated, and contralesional occipital cortical regions between rehab-only and rehab-carryover phases of the trial. The *F*-statistic of each model was calculated, and model performance was evaluated using leave-one-out cross-validation.

### Reporting summary

Further information on research design is available in the Nature Portfolio Reporting Summary linked to this article.

## Online content

Any methods, additional references, Nature Portfolio reporting summaries, source data, extended data, supplementary information, acknowledgements, peer review information; details of author contributions and competing interests; and statements of data and code availability are available at 10.1038/s41591-023-02507-0.

## Supplementary information

Supplementary Information Supplementary Figs. 1–3, Tables 1–4, study protocols 1–2 with summary of changes, DMC charter, summary of screen failures, detailed inclusion/exclusion criteria and surgical procedure details.

Reporting Summary

## Figures and Tables

**Extended Data Table 1:**
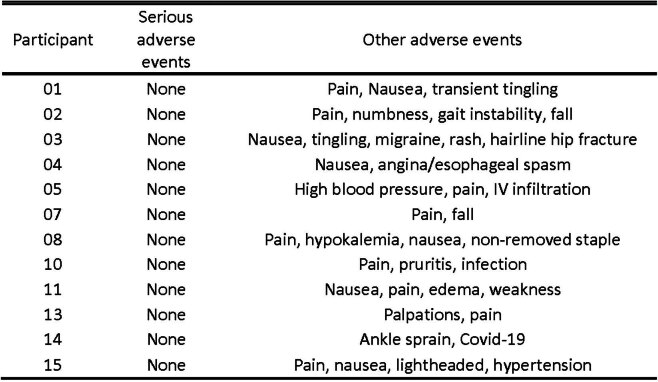
Summary of adverse events by participant

A listing of adverse events and serious adverse events by participant.

**Extended Data Table 2:**
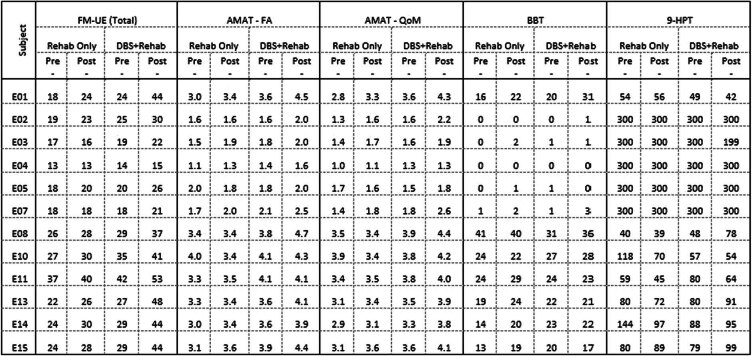
Detailed pre-/post-assessments scores, by subject, across the rehab-only and DBS + rehab Study phases

A listing of pre-/post-assessments scores, by participant, across the rehab-only and DBS + rehab study phases.

## Data Availability

Raw data related to safety and feasibility, the primary endpoints of the study, can be shared upon request and review by Cleveland Clinic and the study sponsors. Secondary endpoint data and PET imaging data, analyzed or raw, may also be shared. Depending on the data that are requested, we will need to consult with the IRB and sponsors before sharing. Cleveland Clinic has regulations related to data sharing, in particular data that could be used as identifiers. The investigators and the IRB will need to verify that data sharing would be acceptable and within policy for patient protection and within the limits of the informed consent provided by participants when enrolling in the study. The investigators will also have to consult the sponsors before data sharing. Patient-related information not included in this report was collected as part of a clinical trial and may be subject to patient confidentiality. Given the restricted study population and small sample size, even though any dataset will be stripped of identifiers before release for sharing, we believe that there remains the possibility of deductive disclosure of participants by unique combinations of characteristics. Therefore, we will make the data and associated documentation available to users only under a data-sharing agreement that provides for a commitment to: (1) using the data only for research purposes and not to identify any individual participant; (2) securing the data using appropriate computer technology; and (3) destroying or returning the data after analyses are completed. Requests for data can be sent to A.G.M. All requests will be answered within 4 weeks. We anticipate that data will be shared, if there are no risks or low risks to the participants.
