# Supplementary material for: Cerebellar deep brain stimulation for chronic post-stroke motor rehabilitation: a phase I trial
Source: Nat Med. 2023 Aug 14;29(9):2366–74. doi: 10.1038/s41591-023-02507-0 (PMC10504081; doi:10.1038/s41591-023-02507-0)
Supplement: Supplementary file 1 — Supplementary Figs. 1–3, Tables 1–4, study protocols 1–2 with summary of changes, DMC charter, summary of screen failures, detailed inclusion/exclusion criteria and surgical procedure details. [file 41591_2023_2507_MOESM1_ESM.pdf]

nature medicine

Article https://doi.org/10.1038/s41591-023-02507-0

Cerebellar deep brain stimulation for chronic post-stroke motor rehabilitation: a phase I trial

In the format provided by the authors and unedited

Cerebellar deep brain stimulation for chronic post-stroke motor rehabilitation: a phase I trial

SUPPLEMENT

Table of Contents

1. **Screen Failures**
2. **Detailed Inclusion/Exclusion Criteria**
3. **Surgical Procedure Details (Supplement Figure 1)**
4. **Lesion Probability Map (Supplement Figure 2)**
5. **Final DBS+Rehab Parameters (Supplement Table 1)**
6. **Detailed Adverse Event Data by Study Phase (Supplement Table 2)**
7. **AMAT – Quality of Movement Data (Supplement Figure 3)**
8. **Data Analysis Tables for Secondary Metrics (Supplement Table 3)**
9. **Complete Listing of Secondary Metrics (Supplement Table 4)**
10. **Original Protocol**
11. **Final Protocol**
12. **Summary of Changes to the Protocol**
13. **DMC Charter**

1. Screen Failures

Three subjects were excluded from the study during the Screening Phase for the following reasons:

- Failed modified Rankin (score of 4 or greater)--INCLUSION ITEM 8
- Unable to discontinue anticoagulants for safe implant of DBS lead--EXCLUSION ITEM 17
- Investigator discretion: Pain that prohibited the conduct of therapy

2. Detailed Inclusion / Exclusion Criteria

Subjects must meet all of the following general inclusion criteria.

1. First-time ischemic stroke 12 to 36months (365 to 1095 days) prior to implant. The index stroke must have been documented by computerized tomography (CT) or magnetic resonance imaging (MRI).
2. Unilateral stroke in the territory of the middle cerebral artery (MCA) sparing the diencephalon and the basal ganglia.
3. 40 to 70 years of age.
4. Transcranial Magnetic Stimulation criterion: Ability to elicit a muscle evoked potential (MEP) in response to TMS delivered to scalp. In a contracted state of the paretic muscle (ranging from 20-50% of maximum voluntary contraction), the ability to evoke reliable criterion MEPs ($\geq 100\mu V$ in 5/10 trials).
5. Medically and neurologically stable as determined by the investigator based on the medical history, physical and neurological examination.
6. Severe residual unilateral upper-extremity hemiparesis defined as ≤ 20 on the upper extremity subscale of the Fugl- Meyer Assessment (FMA-UE) of the affected side. The FMA-UE shall remain 25 or below (FMA-UE ≤ 25) after baseline rehab in order to be eligible for implant.
7. A score ≥ 1 on the FMA-UE elbow flexion, elbow extension, or finger mass.
8. A Modified Rankin Scale score (mRS) < 4.
9. Absent or moderate spasticity in affected limb in any region (shoulder internal rotators and adductors, elbow flexors, wrist flexors or finger flexors) defined as a score of <4 on the modified Ashworth Spasticity Scale.
10. A score of ≥24 on the Mini Mental State Examination.

The subject must not meet any of the following general exclusion criteria.

11. Primary hemorrhagic stroke or major hemorrhagic transformation (Note: Individuals who experience an ischemic stroke with minor hemosiderin in the parenchyma are not excluded.)
12. Any progressive neurological or physical condition other than the index stroke impairing function of the target extremity.
13. Moderate to severe hemispatial neglect or anosognosia involving the affected side of the body.
14. Any other neurological condition that could reduce the safety of study participation including central nervous system vasculitis, intracranial tumor, intracranial aneurysm, multiple sclerosis, or arteriovenous malformations.
15. Pain in the affected limb greater than or equal to 5 on a 0-10 NRS.
16. Evidence of a severe sensory deficit as measured by a score of 2 on the Sensory item (item 8) of the NIH Stroke Scale.
17. Unable to discontinue anticlotting therapy (i.e., antiplatelets and/or anticoagulants) at least 10 days prior to surgery. In the event of a hemorrhagic complication, resuming anticoagulation may be contraindicated for several months.
18. Seizures since the time of stoke, with or without use of antiepileptic agents.
19. Diagnosis of an underlying seizure disorder or epilepsy
20. Change in oral spasticity medications 2 weeks prior to consent or Botox injections in the affected arm within 4 months prior to consent, and/or intention to initiate anti-spasticity medications or Botox injections during study follow-up through 12 months post implantation.
21. Major active psychiatric illness that may interfere with treatment, such as psychotic disorders or severe personality disorders.
22. Untreated or inadequately treated depression defined by a score of 20 or greater on the Beck Depression Inventory-II at the time of enrolment.
23. A diagnosis of dementia.
24. Chronic, uncontrolled high blood pressure, history of severe and unmanaged cardiovascular disorder.

25. Contraindication to magnetic resonance (MR) imaging, e.g., weight incompatible with scanner, implanted metallic devices or electrical devices (pacemaker, defibrillator, spinal cord stimulator).
26. Severe and poorly managed medical disorders that, in the opinion of the PI, requires exclusion from the study due to expected risk likely greater than expected for the stroke population.
27. Enrolled in another device, biologic or pharmaceutical study within 30 days of consent in the current study, (i.e., patient cannot be enrolled if participation in another study was not completed at least 30 days prior to consent.)
28. Non-pregnant women. Women of childbearing potential must be using acceptable forms of contraception. Pregnancy will result in exclusion or discontinuation from the study.
29. Undergone a decompressive hemicraniectomy.
30. Patient has significant chronic small vessel ischemic disease, vertebrobasilar vascular disease, and/or any other structural abnormalities of the cerebellum, cerebellar peduncles, and brain stem that would preclude safe placement of the DBS lead.
31. Patient has a condition that, in the opinion of the investigator, would significantly increase the risk for interfere with study compliance, safety or outcome.

Detailed Transcranial Magnetic Stimulation-related Inclusion Criterion

The extensor digitorum communis (EDC) of the impaired upper extremity is the target muscle. TMS stimulation will be applied to the ipsilesional hemisphere (contralateral to the impaired limb). Single-pulse TMS (Magpro) will be delivered using a figure-of-eight coil. The coil will be placed tangentially on the scalp with the handle oriented backwards and laterally at 45° from mid-sagittal axis. Patient's MRI will be used to localize and guide the coil using frameless stereotaxy. Stereotaxic localization of the coil will help ensure that application of TMS is repeatable and consistent. The location specifically would lie in the motor cortex. In this region, we would identify the location of the "hot spot". The "hot spot" is defined as the scalp site where single pulse stimulation (0.2 Hz) results in reliable muscle evoked potentials (MEPs) (criterion: >=100µV in 5/10 trials) in the active state of the muscle (20-50% of maximum voluntary contraction) at the

lowest intensity. Closely spaced surface EMG electrodes will be placed over the mid-belly of the target muscle.

An individual meets the inclusion criterion if there is an ability to elicit MEPs that meet the above criterion. An inability to elicit MEPs that meets the above criterion at any location over the ipsilesional motor cortex at maximum intensity of the device will exclude the potential candidate from being enrolled in the study. A seizure at any point during the testing of the TMS inclusion criteria will discontinue all further testing and exclude the potential candidate from being enrolled. Since patients below UE FMA <20 will be recruited as well, it is likely that such patients EDC may not evoke any MEP. In such cases, electrodes will be applied to proximal muscles that are more likely to have spared pathways in patients with severe damage. We will elicit MEPs in triceps and middle deltoid muscles as alternatives. Electrodes will be placed over the muscle belly at the junction of the middle and the lower third of the muscle. Electrodes will be applied in a direction that is parallel to the direction of the muscle fibers.

3. Deep Brain Stimulation Surgical Procedure

Participants underwent pre-operative anatomical MRI within the month prior to surgery. On the day of surgery, a stereotactic head frame (Leksell, Elekta, Stockholm) was placed under general anesthesia, followed by a stereo-CT. The images were subsequently co-registered to a preoperative, volumetric CT and MRI for creation of a surgical plan using a commercially-available surgical planning station (Stealth, Medtronic, Minneapolis or BrainLab, Munich). The surgical plan was based on a posterior fossa entry point with the tip of the lead targeted to the white matter immediately anteromedial to the DN. A lateral to medial and inferior to superior trajectory was designed to avoid superficial vasculature and the horizontal fissure of the cerebellum. Subjects remained under general anesthesia in the prone position throughout the surgical procedure. Immediately prior to insertion, when the cannula was aligned at the level of the dura, an intra-operative stereo-image was acquired with the O-arm (Medtronic, Minneapolis) and again co-registered to the pre-operative plan to confirm that the trajectory of the cannula was consistent with stereotactic plan. Deviations from the plan were corrected using an x/y stage over the arm supported by the stereotactic arc. A second intra-operative scan was acquired after lead implantation to confirm placement accuracy prior to anchoring and closure. Once the muscle and fascia were approximated, a temporary lead extension was affixed to the outer end of the lead and tunneled subcutaneously to the posterior surface of the shoulder. The system remained externalized for one week, after which the participant was brought back to the OR for implantation of the pulse generator in the ipsilateral infraclavicular area using standard technique. One-month post-surgery, participants underwent an additional volumetric CT scan, which was subsequently co-registered to the pre-operative MRI and used to generate 3D models of the DN and corresponding lead locations (BrainLab, Munich, Germany).

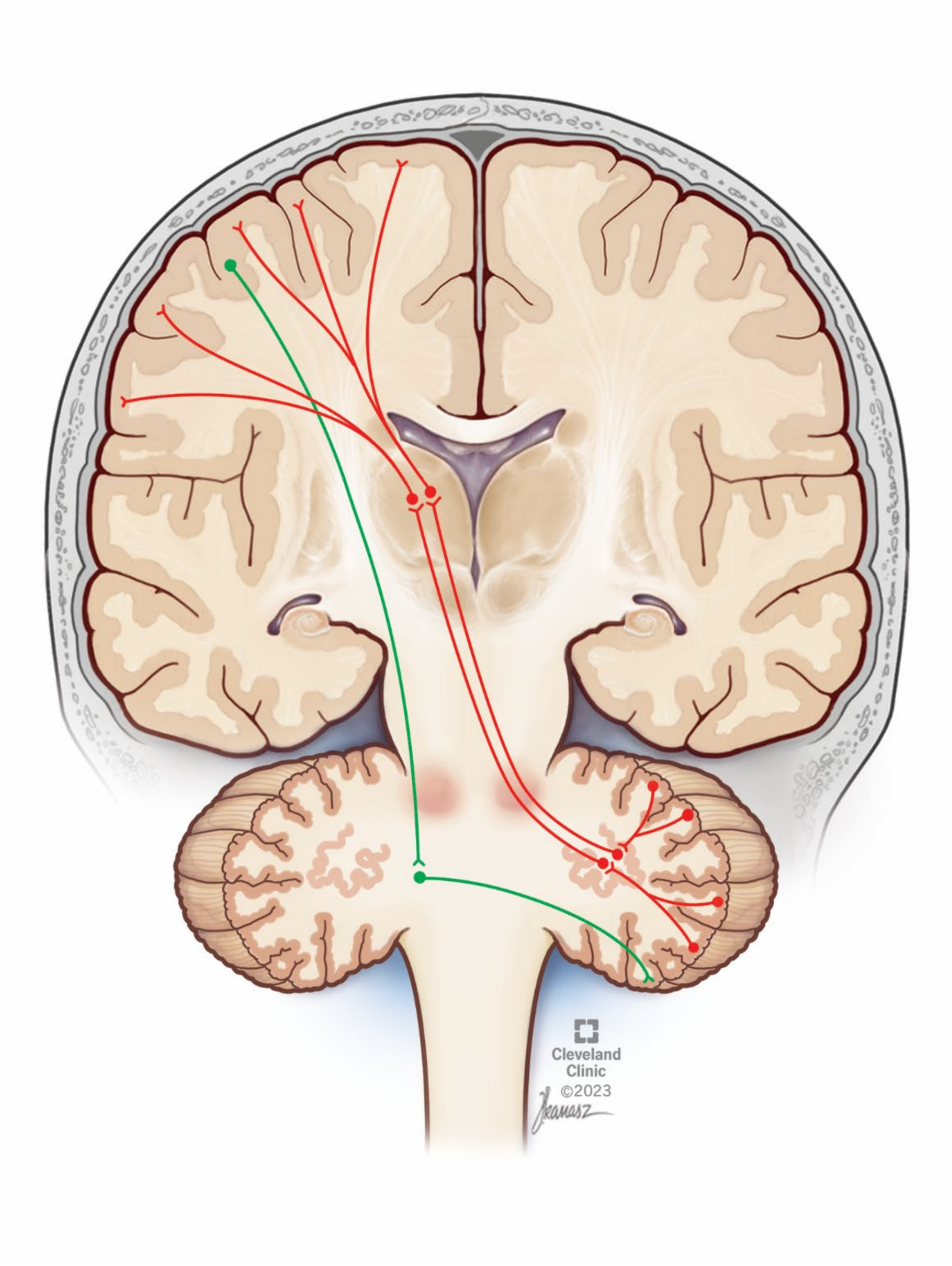

1A

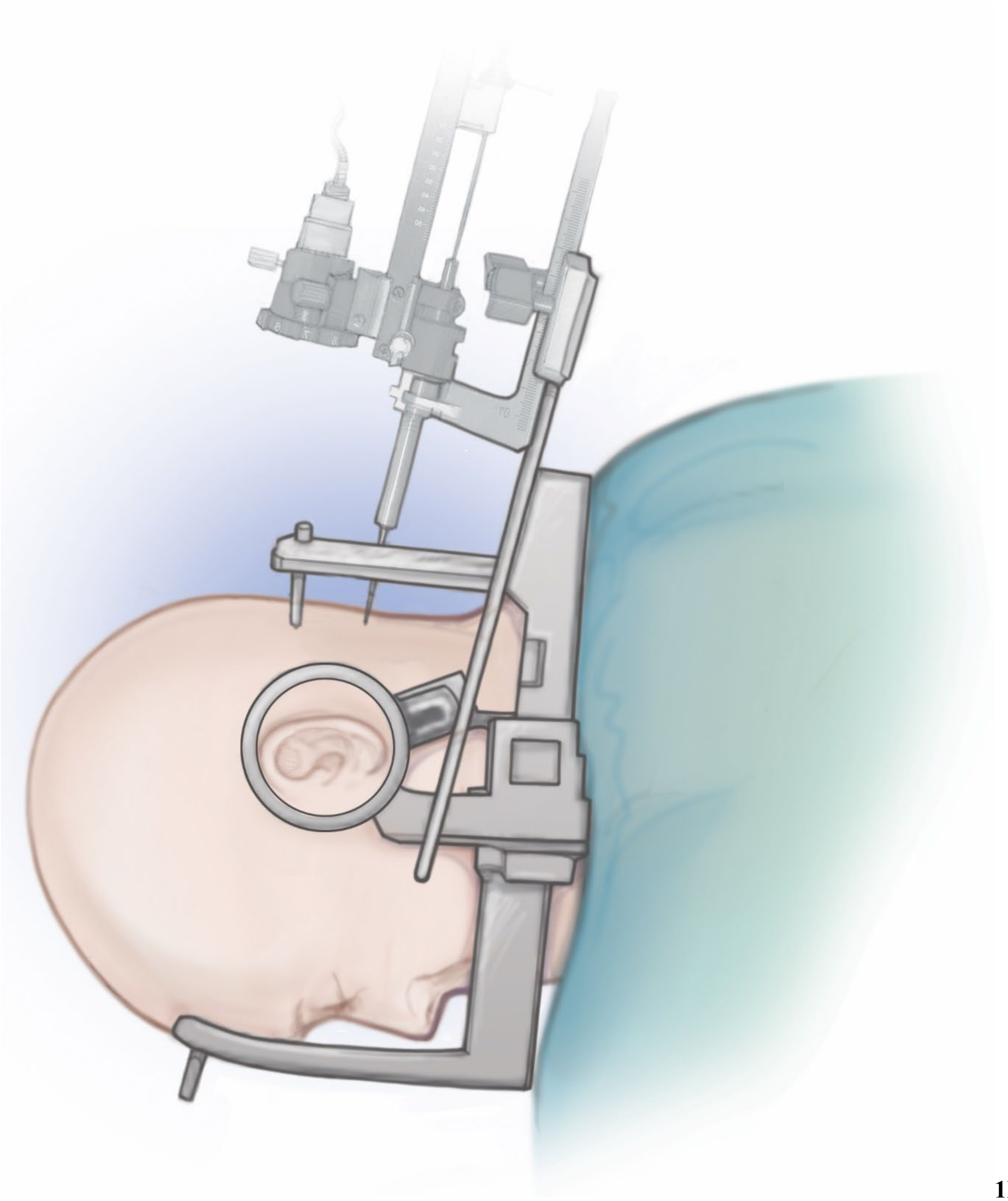

1B

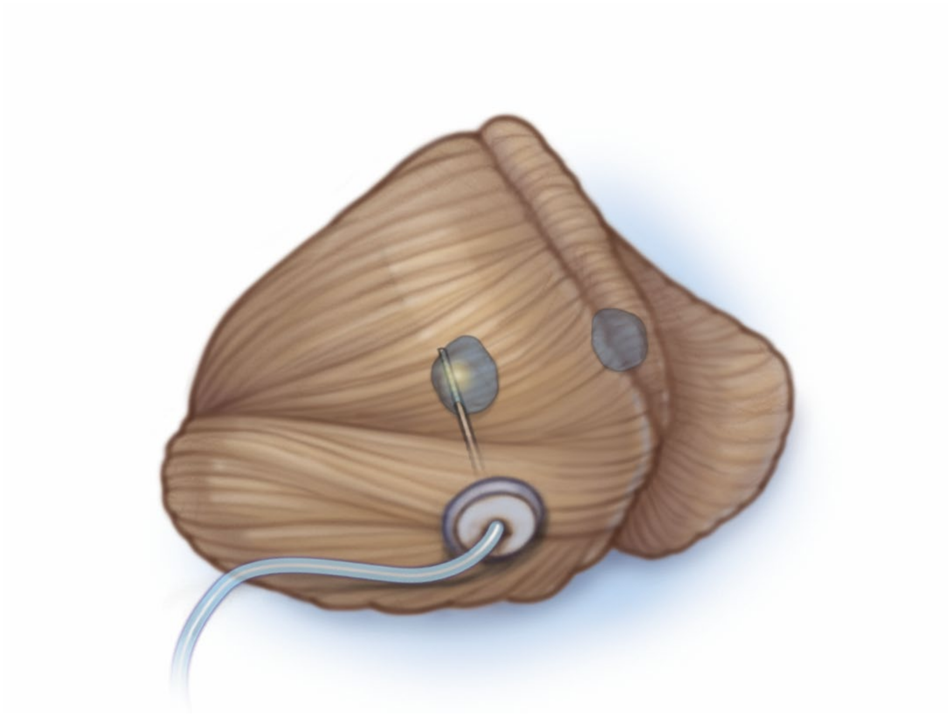

1C

Figure 1. Dentate nucleus deep brain stimulation surgery**.** (A) Surgery targets the ascending dentatothalamocortical (red) pathway through implantation of an 8-contact DBS lead at its origin in the cerebellar dentate nucleus. (B) Surgery is performed with the patient in the prone position under general anesthesia using a Leksell stereotactic frame, with the lead implanted via a burrhole craniotomy in the posterior fossa. (C) A 3D-model rendered from co-registration of one participant's pre-operative MRI and post-implant CT depicts the lead entering through the cerebellar cortex on a lateral-to-medial, inferior-to-superior trajectory targeting the left cerebellar dentate nucleus (blue).

4. Lesion Probability Map

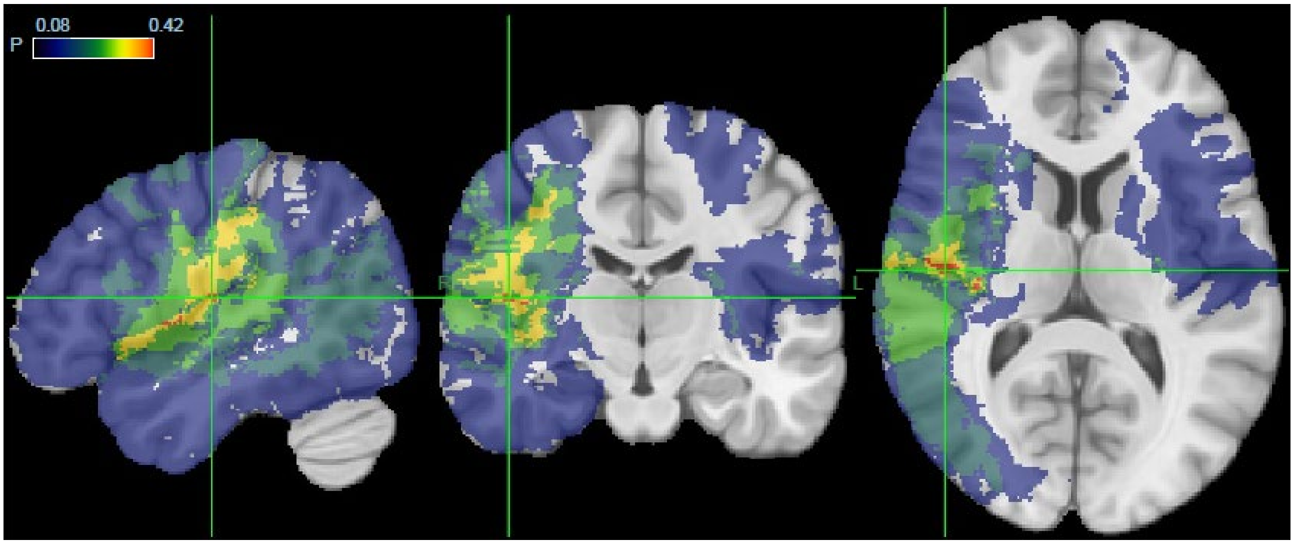

Figure 2: Lesion probability map. The probability of stroke lesion is shown in MNI152 space. Values range from 0.08 (in dark blue, where 1 of 12 patients exhibited a lesion) to 0.42 (in red, where 5 of 12 patients exhibited a lesion).

5. Final DBS + Rehab Phase Parameters

Table 1: Final DBS+Rehab DBS Parameters

		Cathode		Anode				
Participant	Directional DBS Lead?	Contacts (-)[a]	Distribution (%)	Contacts (+)[a]	Distribution (%)	Rate	Pulse Width (µs)	Amplitude (mA)
01	No	5/6/7	34/33/33	IPG	100	30	90	6.0
02	No	4/5/6	34/33/33	IPG	100	185	90	4.4
03	No	4/5/6	34/33/33	IPG	100	30	200	6.0
04	Yes	2/5	50 each	1/3/4/6/7/8	17 each	30	90	8.0
05	Yes	2/3/4	34/33/33	5/6/7	34/33/33	30	90	6.0
07	Yes	5/6/7	34/33/33	8	100	30	90	6.0
08	Yes	1/2/3/4	25 each	8	100	30	90	6.0
10	Yes	1/2/3/4	25 each	8	100	30	90	6.0
11	No	4/5/6	34/33/33	8	100	30	90	9.0
13	No	5/6	50 each	8	100	30	90	6.0
14	No	4/5	50 each	8	100	30	90	7.5
15	No	2/3/4	34/33/33	8	100	30	90	6.0

[a]Numbers represent each contact activated. Multiple numbers signify parallel use of multiple contacts.

6. Detailed Adverse Events by Study Phase

Table 2: Detailed Adverse Events by Study Phase

Summary of all adverse events since the start of the study, irrespective of study relatedness†.

Study Phase (Device Status)	Subj	Brief description	Intervention	Serious	Anticipated in protocol/ consent	Study † relatedness	Category (e.g., AE, SAE, UADE)	Did AE result in stopping treatment
Post Implant (Device OFF)	001, 003, 011	Post-anesthesia nausea	medication	no	yes	yes	AE	no
	001	Post-surgical hip discomfort due surgical positioning	none	no	yes	yes	AE	no
	005, 007, 008, 010, 011	Post-surgical head pain, headache or head sensitivity	medication	no	yes	yes	AE	no
	010	Pruritus	changed medication	no	yes	yes	AE	no
Therapy Baseline (Device Off)	008, 015	Surgical site pain	None or medication	no	yes	yes; device	AE	no
	010	IPG Site redness	antibiotic	no	yes	yes; device	AE	no
	013	Eye pain	none	no	yes	no	AE	no
Programming (Device ON < 1-hour increments)	001, 003, 004, 015	Nausea or vomiting	modified DBS parameters	no	yes	yes; stimulation	AE	no
	002	Eye infection (conjunctivitis)	medication (antibiotics)	no	yes	no	AE	no
	003	Allergic contact dermatitis (e.g., Poison Ivy)	oral & topical medication	no	no	no	AE	no
	003	Transient weakness	modified DBS parameters	no	yes	yes; stimulation	AE	no
	004	Nausea and vomiting during DBS programming	modified DBS parameters	no	yes	yes; stimulation	AE	no
	004	Angina/esophageal spasm (in the evening while device OFF)	none	no	no	no	AE	no
	008	Discomfort from EEG electrodes	modified EEG electrode placement	no	no	no	AE	no
	008	Elevated blood pressure	Medication adjustment	no	no	no	AE	no
	008	Hypokalemia	oral potassium (40 mEq)	no	yes	no	AE	no
	008	Unusual behaviors (due to cannabis use)	Negative workup	no	no	no	AE	no
	013	Stubbed toe, with periungual redness	Medication, podiatry consult	no	yes	no	AE	no

DBS+Rehab (Device ON)	001	Brief “pins and needles” in back of the head after lighting strike outside subject’s house	none	no	yes	possibly	AE	no
	005	High blood pressure (forgot to refill routine BP medication)	medication (Norvasc)	no	yes	no	AE	no
	008	Headache and back pain (due to car accident)	medication (Tylenol and Lidoderm);	no	no	no	AE	no
		Hypokalemia and Emergency Department	oral and IV potassium	yes	no	no	SAE	no
		Leg Cramping	Tylenol	no	no	no	AE	no
	011	Transient hand edema with grip weakness attributed to overuse	Medrol 4mg dosepak and rest	no	yes	no	AE	no
	015	Orthostatic hypertension	medication adjusted (hypertension by PCP)	no	yes	no	AE	no
Rehab Carryover (Device OFF)	002	Numbness in the leg a few days after DBS turned OFF (history of numbness)	ice / elevate	no	yes	possibly	AE	no
	003	Fall/hairline hip fracture (slipped on ice)	rest / non-loadbearing; pain medication	no	yes	no	AE	no
	005	Tooth pain	Tooth extracted	no	no	no	AE	no
	014	Rolled ankle leaving clinic	ice / elevate	no	yes	no	AE	no
Long-term Follow-up (Device Explanted)	002	Abdominal pain (extensive history of abdominal issues)	follow-up w/ gastroenterologist	no	no	no	AE	no
		Leg numbness/tingling and gait issues with history of spine disease	Medication, referral neurologist; PT	no	yes	no	AE	no
		Fall (tripped in a parking lot)	seen by PCP	no	yes	no	AE	no
	003	Migraine with an episode of vomiting (history of migraines)	medication (nausea and migraine)	no	no	no	AE	no
		Rash on abdomen	Topical cream	no	yes	yes; post-explant	AE	no
	005	Rib pain (due to car accident)	Chest x-ray, medication	no	no	no	AE	no
		IV access infiltrated	new IV placed	no	yes	yes; at explant	AE	no
	007	Mild shoulder pain due to fall	none	no	yes	no	AE	no
	008	One staple not removed during surgical follow-up	Staple removed	no	yes	yes; explant procedure	AE	no

	011	Post-surgical headache	medication	no	yes	yes; explant procedure	AE	no
	013	Felt fast heart rate / palpitations	Cardiac workup (EKG, x-ray, D-dimer etc.)	no	yes	no	AE	no
	014	Subject reported positive test for Covid-19 / SARS-Cov-2	Ibuprofen	no	no	no	AE	no
	015	Orthostatic hypertension; Mild symptoms represented at EOS	medication management by PCP	no	yes	no	AE	no

† Per the protocol (see §8.1.7), study relatedness is cataloged as "implant procedure, DBS device, or stimulation with consideration of the strength of the temporal relationship to the implant procedure, onset or cessation of stimulation and the presence or performance of the DBS system ..." The investigator makes the initial relatedness determination which is later adjudicated by the DMC during the next scheduled meeting.

7. AMAT – Quality of Movement Data

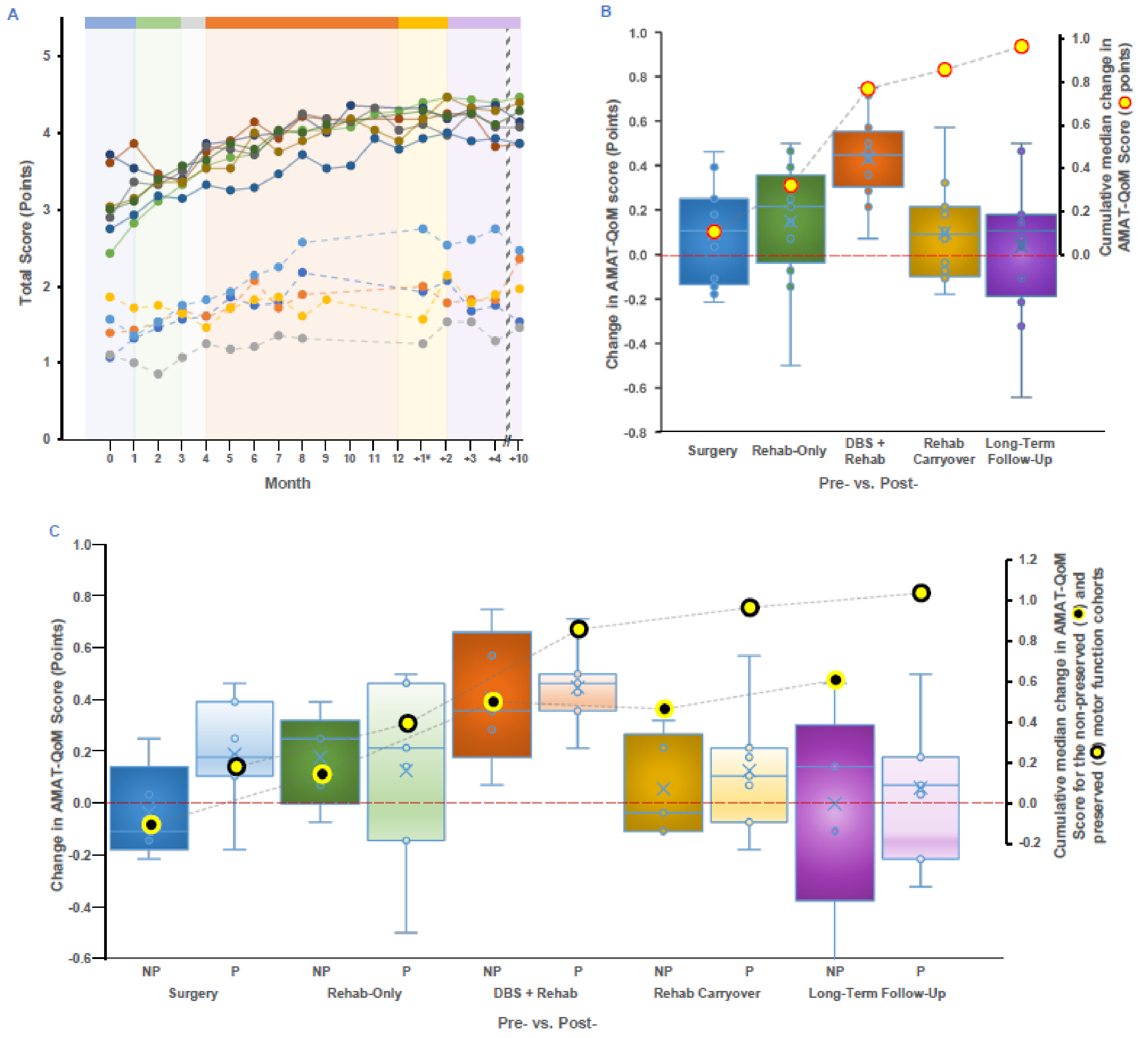

Figure 3: Trial overview and trial-related data for the AMAT Quality of Movement (QoM) subscale. A) Overall design of the open-label, non-blinded Phase I trial. All efficacy assessments were recorded monthly. Study phase color shading is used throughout remainder of the figure. B) Individual AMAT-QoM scores for each participant across the trial. The absence of a score marker for a given month during the DBS+Rehab

phase signifies that the participant had met the criteria for DBS+Rehab discontinuation and transitioned to the carry-over phase. Dashed lines are used to highlight participants classified as non-preserved (NP) with regards to UE distal function at enrollment. Note that the inter-connecting lines are shown as a visual aid and not intended to convey continuity or linearity of change. C) Box-and-whisker plots representing the change scores (left y-axis) on the AMAT-QoM across each of the individual surgical (Month 1 minus 0), Rehab-Only (Month 3 minus 1), DBS+Rehab (Month 8-12 [max achieved] minus 4), Rehab Carryover (Month '+2' minus Month 8-12), and Long-term follow-up (Month '+10' minus 8-12) phases of the trial. A change score of zero signifies that no effect on impairment was observed across that phase, with higher values reflecting improvement (i.e., decreased impairment). The overlaid line plot represents the accumulation of median change (right y-axis) combined sequentially across each of the five phases. D) Box-and-whisker change plots and cumulative line plots for the AMAT-QoM data as function of non-preserved (NP) versus preserved (P) baseline distal extremity motor function. The overlaid line plots show the cumulative change (right y-axis) observed across each phase of the trial (NP: Solid red circle; P: White-filled red circle).

8. Data Analysis Tables for Secondary Metrics

Table 3: Data Analysis Tables for Secondary Metrics

FMA-UE

	Full sample<br>*Median (IQR)*<br>*[95% CI for median]*<br>*S*<br>*p*	Less impaired<br>*Median (IQR)*<br>*[95% CI for median]*<br>*S*<br>*p*	More impaired<br>*Median (IQR)*<br>*[95% CI for median]*<br>*S*<br>*p*
Pre-/Post-Surgery	-0.5 (-1.0 – 1.0)<br>[-0.92, 0.75]<br>-1.5<br>0.998	NA	NA
Rehab-Only	3.0 (1.0 – 4.0)<br>[1.31, 4.19]<br>26.5<br>0.0039	4.0 (3.0 – 6.0)<br>[2.59, 5.41]	0.0 (0.0 – 2.0)<br>[-1.48, 3.48]
DBS + Rehab	7.0 (4.0 - 15.0)<br>[5.18, 13.82]<br>39.0<br>0.0005	15.0 (8.0 - 20.0)<br>[8.44, 19.0]	3.0 (3.0 - 5.0)<br>[1.18, 6.02]
Rehab Carryover	0.0 (-2.0 - 2.5)<br>[-4.09, 3.09]<br>2.0<br>0.8359	1.0 (-3.0 - 4.0)<br>[-7.59, 6.16]	0.0 (-1.0 - 0.0)<br>[-2.89, 2.49]
Long-term Follow-up	0.0 (-2.0 – 1.0)<br>[-2.76, 1.76]<br>-3.5<br>0.7148	1.0 (-2.0 – 4.0)<br>-1.84, 3.55]	-1.0 (-2.0 -0.0)<br>[-7.10, 2.30]

*IQR = interquartile range. 95% CI for median is calculated assuming normality. S=Signed rank test statistic

AMAT - Composite

	Full sample<br>*Median (IQR)*<br>*[95% CI for median]*<br>*S*<br>*p*	Less impaired<br>*Median (IQR)*<br>*[95% CI for median]*	More impaired<br>*Median (IQR)*<br>*[95% CI for median]*
Rehab-Only	0.196 (0.027 – 0.348)<br>[-0.03, 0.33]<br>22.0<br>0.0923	0.214 (-0.036 – 0.464)<br>[-0.16, 0.48]	0.107 (0.089 – 0.321)<br>[-11, 0.41]
DBS + Rehab	0.411 (0.250 – 0.517)<br>[0.27, 0.54]<br>39.0<br>0.0005	0.429 (0.339 – 0.696)<br>[0.23, 0.68]	0.250 (0.250 – 0.482)<br>[0.12, 0.55]
Rehab Carryover	0.125 (0.0 – 0.259)<br>[0.01, 0.27]<br>27.0<br>0.0327	0.161 (0.018–0.339)<br>[-0.06, 0.40]	0.018 (-0.018-0.214)<br>[-0.09, 0.29]

Long-term Follow-up	0.018 (-0.223 - 0.170)<br>[-0.210. 0.185]<br>5.0<br>0.7334	0.018 (-0.393 - 0.268)<br>[0.286, 0.274]	0.018 (-0.054 – 0.071)<br>[0.468, 0.424]

*IQR = interquartile range. 95% CI for median is calculated assuming normality. S=Signed rank test statistic

AMAT - MFA

	Full sample<br>*Median (IQR)*<br>*[95% CI for median]*<br>*S*<br>*p*	Less impaired<br>*Median (IQR)*<br>*[95% CI for median]*	More impaired<br>*Median (IQR)*<br>*[95% CI for median]*
Rehab-Only	0.215 (0.018 – 0.393)<br>[-0.036, 0.352]<br>21.0<br>0.1060	0.286 (0.071 – 0.429)<br>[-0.142, 0.510]	0.107 (-0.036 – 0.357)<br>[-0.199, 0.442]
DBS + Rehab	0.339 (0.214 – 0.482)<br>[0.201, 0.561]<br>38.0<br>0.0010	0.464 (0.250 – 0.893)<br>[0.143, 0.785]	0.214 (0.214 – 0.357)<br>[0.133, 0.296]
Rehab Carryover	0.214 (0.071 – 0.268)<br>[0.043, 0.320]<br>0.0161	0.250 (-0.036 – 0.464)<br>[-0.050, 0.468]	0.071 (0.071 – 0.214)<br>[0.017, 0.268]
Long-term Follow-up	0.054 (-0.160 – 0.179)<br>[-0.138, 0.244)<br>5.5<br>0.6934	0.178 (-0.214 – 0.179)<br>[-0.174, 0.427]	-0.107 (-0.107 - -0.036)<br>[0.370, 0.271]

*IQR = interquartile range. 95% CI for median is calculated assuming normality. S=Signed rank test statistic

AMAT - MQM

	Full sample<br>*Median (IQR)*<br>*[95% CI for median]*<br>*S*<br>*p*	Less impaired<br>*Median (IQR)*<br>*[95% CI for median]*	More impaired<br>*Median (IQR)*<br>*[95% CI for median]*
Rehab-Only	0.214 (0.0 – 0.321)<br>[-0.030, 0.328]<br>23.0<br>0.0728	0.214 (-0.143 – 0.464)<br>[-0.196, 0.451]	0.250 (0.0714 – 0.250)<br>[-0.045, 0.403]
DBS + Rehab	0.446 (0.321 – 0.535)<br>[0.308, 0.556]<br>39.0<br>0.0005	0.464 (0.357 – 0.500)<br>[0.309, 0.589]	0.357 (0.286 – 0.571)<br>[0.082, 0.732]
Rehab Carryover	0.089 (-0.089 – 0.214)<br>[-0.039, 0.236]<br>18.0<br>0.1704	0.107 (-0.071 – 0.214)<br>[-0.094, 0.349]	-0.036 (-0.107 – 0.214)<br>[-0.189, 0.303]
Long-term Follow-up	0.107 (-0.162 – 0.266)<br>[-0.161, 0.261]<br>9.0<br>0.519	0.071 (-0.217 - 0.357)<br>[-0.184, 0.356]	0.143 (-0.107 – 0.143)<br>[-0.512, 0.512]

*IQR = interquartile range. 95% CI for median is calculated assuming normality. S=Signed rank test statistic

NHPT

	Full sample<br>*Median (IQR)*<br>*[95% CI for median]*<br>*S*<br>*p*	Less impaired<br>*Median (IQR)*<br>*[95% CI for median]*	More impaired<br>*Median (IQR)*<br>*[95% CI for median]*
Rehab-Only	0.0 (-11.37 – 0.95)<br>[-24.29, 23.66]<br>-4<br>0.6406	-8.37 (-46.38 – 1.89)<br>[-36.82, 6.08]	0.0 (0.0 – 0.0)<br>[-36.89, 78.42]
DBS + Rehab	0.0 (07.31 – 6.55)<br>[-29.51, 14.91]<br>-2<br>0.8125	1.65 (-7.31 – 10.34)<br>[-13.65, 20.43]	0.0 (0.0 – 0.0)<br>[-76.01,35.76]
Rehab Carryover	0.0 (-29.64 – 6.79)<br>[-55.02, 33.43]<br>-2<br>0.8438	2.46 (-29.64 – 6.79)<br>-29.21, 15.65]	0.0 (0.0 – 0.0)<br>[-141.07, 109.85]
Long-term Follow-up	0.0 (-13.59 – 0.30)<br>[-26.03, 51.90]<br>0.0<br>>0.9999	-12.48 (-39.11 – 0.30)<br>[-68.06, 84.44]	0.0 (0.0 – 0.0)<br>[-33.09, 70.35]

*IQR = interquartile range. 95% CI for median is calculated assuming normality. S=Signed rank test statistic

BAI

	Full sample<br>*Median (IQR)*<br>*[95% CI for median]*<br>*S*<br>*p*	Less impaired<br>*Median (IQR)*<br>*[95% CI for median]*	More impaired<br>*Median (IQR)*<br>*[95% CI for median]*
Rehab-Only	0.0 (-1.0 – 1.0)<br>[-2.01, 2.35]<br>1.5<br>0.9141	0.0 (-4.0 – 1.0)<br>[-4.38, 3.81]	1.0 (0.0 – 1.0)<br>[-1.04, 2.64]
DBS + Rehab	-1.0 (-3.5 – 0.0)<br>[-3.01, -0.66]<br>-18<br>0.0078	-1.0 (-3.0 – 0.0)<br>[-3.54, 0.11]	-1.0 (-4.0 - -1.0)<br>[-4.32, 0.32]
Rehab Carryover	0.0 (0.0 – 1.0)<br>[-0.44, 2.6-]<br>5.5<br>0.2500	0 (0.0 - 0.0)<br>[-0.53, 0.53]	1.0 (0.0 – 5.0)<br>[-1.38, 6.58]
Long-term Follow-up	1.0 (0.0-3.0)<br>[0.113, 3.054]<br>15.5<br>0.0391	0.0 (-4.0 0 1.0)<br>[-4.38, 3.81]	2.0 (0.0 – 4.0)<br>[-1.08, 6.28]

*IQR = interquartile range. 95% CI for median is calculated assuming normality. S=Signed rank test statistic

BBT

	Full sample<br>*Median (IQR)*<br>*[95% CI for median]*	Less impaired<br>*Median (IQR)*<br>*[95% CI for median]*	More impaired<br>*Median (IQR)*<br>*[95% CI for median]*

	S<br>*p*	*S*<br>*p*	*S*<br>*p*
Rehab-Only	1.5 (0.0 – 5.5)<br>[0.51, 4.32]<br>21<br>0.0352	5.0 (-1.0 – 6.0)<br>[0.33, 6.81]	0.0 (1.0 – 1.0)<br>[-0.24, 1.83]
DBS + Rehab	0.0 (-1.0 – 1.5)<br>[-1.27, 3.44]<br>5.5<br>0.6133	-1.0 (-1.0 – 5.0)<br>[-2.92, 6.07]	0.0 (0.0 – 1.0)<br>[-1.02, 1.82]
Rehab Carryover	0.5 (-0.5 – 3.0)<br>[-0.24, 4.07]<br>15<br>0.0859	3.0 (0.0 – 8.0)<br>[-0.07, 6.92]	0.0 (-1.0 – 0.0)<br>[-1.24, 0.84]
Long-term Follow-up	0.0 (-1.0- 7.50)<br>[-3.730, 14.618]<br>8.0<br>0.2969	7.0 (-2.0 – 9.0)<br>[-7.27, 26.50]	0.0 (-1.0 – 0.0)<br>[-1.08, 0.28]

*IQR = interquartile range. 95% CI for median is calculated assuming normality. S=Signed rank test statistic

<u>BDI</u>

	Full sample<br>*Median (IQR)*<br>*[95% CI for median]*<br>*S*<br>*p*	Less impaired<br>*Median (IQR)*<br>*[95% CI for median]*	More impaired<br>*Median (IQR)*<br>*[95% CI for median]*
Rehab-Only	-2.5 (-6.0 - -0.5)<br>[-6.55, -0.11]<br>-24<br>0.0332	-2.0 (-3.0 – 0.0)<br>[-4.76, 1.90]	-8.0 (-5.0 – -3.0)<br>[-13.45, 1.45]
DBS + Rehab	-1.0 (-6 – 0)<br>[-5.01, 0.17]<br>-15<br>0.0859	-1.0 (-4.0 – 0.0)<br>[-5.53, 0.39]	0.0 (-8.0 – 1.0)<br>[-9.02, 4.62]
Rehab Carryover	0.5 (-0.5 – 2.0)<br>[-0.86, 2.86]<br>0.3750	0.0 (-2.0 – 1.0)<br>[-1.95, 1.66]	1.0 (1.0 – 4.0)<br>[-1.75, 6.95]
Long-term Follow-up	0.0 (-1.5 – 2.5)<br>[-1.21, 4.38]<br>8.0<br>0.3867	0.0 (-2.0 – 0.0)<br>[-2.07, 0.93]	3.0 (2.0 – 8.0)<br>[-2.18, 11.38]

*IQR = interquartile range. 95% CI for median is calculated assuming normality. S=Signed rank test statistic

<u>EQ-5D</u>

	Full sample<br>*Median (IQR)*<br>*[95% CI for median]*<br>*S*<br>*p*	Less impaired<br>*Median (IQR)*<br>*[95% CI for median]*	More impaired<br>*Median (IQR)*<br>*[95% CI for median]*
Rehab-Only	0.0 (-1.0 – 0.0)<br>[-1.65, 0.15]<br>-8<br>0.1563	0.0 (-1.0 – 0.0)<br>[-1.75, 0.61]	0.0 (-1.0 – 0.0)<br>[-3.13, 1.15]

DBS + Rehab	0.0 (-3.0 – 0.0)<br>[-2.30, 0.30]<br>-9<br>0.1563	-1.0 (-4.0 – 0.0)<br>[-3.25, 0.11]	0.0 (0.0 – 1.0)<br>[-3.03, 2.63]
Rehab Carryover	0.50 (0.0 – 3.0)<br>-0.18, 2.52<br>0.1094	0.0 (0.0 – 3.0)<br>[-0.31, 2.31]	1.0 (-1.0 – 4.0)<br>[-2.39, 5.19]
Long-term Follow-up	0.50 (0.0 – 1.50)<br>[-0.16, 1.49]<br>9.0<br>0.156	0.0 (0.0 – 1.0)<br>[-0.48, 1.62]	1.0 (1.0- 2.0)<br>[-1.24, 2.84]

*IQR = interquartile range. 95% CI for median is calculated assuming normality. S=Signed rank test statistic

<u>MAS</u>

	Full sample<br>*Median (IQR)*<br>*[95% CI for median]*<br>*S*<br>*p*	Less impaired<br>*Median (IQR)*<br>*[95% CI for median]*	More impaired<br>*Median (IQR)*<br>*[95% CI for median]*
Rehab-Only	0.5 (-2.5 – 1.5)<br>[-0.42, 4.23]<br>-1<br>1.0<br>n = 8	1.0 (-1.0 – 2.00<br>[-2.26, 2.60]<br>n = 6	-2.0 (-4.0 – 0.0)<br>[-27.41, 23.41<br>n = 2
DBS + Rehab	-1.0 (-2.0 – 0.0)<br>[-2.63, 0.42]<br>-11.5<br>0.1953<br>n = 10	-2.0 (-3.0 - -1.0)<br>[-3.69, 0.26]<br>n =7	0.0 (-1.0 – 2.0)<br>[-3.46-4.13]<br>n = 3
Rehab Carryover	0 (-1.0 – 2.5)<br>[-1.05, 2.39]<br>7<br>0.4570	1.0 (-2.0 – 3.0)<br>[-2.10, 3.53]	0.0 (-1.0 – 0.0)<br>[-2.52, 3.71]
Long-term Follow-up	1.50 (0.5 – 2.5)<br>[-0.17, 3.01]<br>21.0<br>0.0664	2.0 (-1.0 – 4.0)<br>[-1.58, 4.44]	1.0 (1.0 – 2.0)<br>[-0.02, 2.82]

*IQR = interquartile range. 95% CI for median is calculated assuming normality. S=Signed rank test statistic

9. Complete Listing of Secondary Metrics

Table 4: Complete Listing of All Secondary Metrics

Measure	Domain
Fugl-Meyer Assessment, Upper Extremity (FM-UE)	Impairment Index
Arm Motor Ability Test, Version 13 (AMAT)	Activities of daily living
9-Hole Peg Test (9-HPT)	Fine motor coordination
Bilateral Box and Block Test (BBT)	Gross manual dexterity
Bimanual Grip Test	Dexterity and fine motor control
Modified Ashworth (MAS)	Muscle spasticity
Short Form Health Survey (SF-12)	Self-report quality of life
EuroQol (EQ-5D)	Health-related quality of life
Beck Anxiety Inventory (BAI)	Self-report anxiety
Beck Depression Inventory (BDI)	Self-report depression
18F-fluorodeoxyglucose positron-emission tomography	Brain metabolism
Transcranial magnetic stimulation - Motor evoked potentials	Cortical excitability
Transcranial magnetic stimulation - Motor maps	Cortical reorganization

Electrical Stimulation of the Dentate Nucleus area (EDEN) for Improvement of Upper Extremity Hemiparesis due to Ischemic Stroke: A Safety and Feasibility Study

Protocol Number: REDD 0002

December 4, 2015

Sponsor Information:
Enspire DBS Therapy, Inc.
2 Oliver St. STE 616
Boston, MA 02446

Contact Person:
[redacted]

PROTOCOL SYNOPSIS

Electrical Stimulation of the Dentate Nucleus area (EDEN) for Improvement of Upper Extremity Hemiparesis due to Ischemic Stroke: A Safety and Feasibility Study	
**Study Overview**	
**Study Objective**	The objective of this study is to document the safety and patient outcomes of electrical stimulation of the dentate nucleus area for the management of chronic, moderate to severe upper extremity hemiparesis due to ischemic stroke.<br><br>The proposed study is a first in human safety and feasibility study intended to provide preliminary data to design a future pilot study.
**Test Device**	Boston Scientific, Inc. Vercise™ Deep Brain Stimulation System.
**Investigational Treatment**	Unilateral electrical stimulation of the cerebellar dentate nucleus on the side ipsilateral to the hemiparesis (contralesional to stroke).
**Study design**	
**Study Design**	Prospective, open-label, single arm, safety and feasibility study
**Control**	Due to heterogeneity of ischemic stroke, each patient will serve as his or her own control. Comparison will be made across time (e.g. rehabilitation vs. test, and follow-up)
**Investigative Sites**	One site (Cleveland Clinic).
**Number of Subjects**	Up to 5 subjects enrolled. After the first subject is implanted, no additional subjects will be implanted until data from 5 months of follow-up from the first subject have been reported to the FDA and the FDA approves additional subjects.<br><br>Note: Enspire's goal for the study is to eventually enroll 12 subjects total, this protocol will be amended when initial data is available from the first subjects. FDA and IRB approvals are needed to expand the study to 12 subjects total.
**Patient Population**	Survivors of an initial ischemic stroke between 12 and 24 months post stroke with residual severe unilateral, upper extremity hemiparesis defined as ≤ 20 on the upper extremity subscale of the Fugl-Meyer Assessment.

**Primary Endpoint**	The primary endpoint will be the incidence of all serious adverse events, including Serious Adverse Events (SAEs), Serious Adverse Device Events (SADEs), and Unanticipated (Serious) Adverse Device Events (UADE), from the time of enrollment through follow-up.<br><br>All serious adverse events will be further categorized as procedure-, DBS device-, and stimulation-related.<br>All non-serious adverse events will also be tabulated and reported.
**Secondary Endpoints**	• Arm Motor Ability Test (AMAT)<br>• Bilateral Box and Block Test (BBT)<br>• Bimanual Grip strength test<br>• EuroQol (EQ-5D)<br>• Fugl-Meyer Assessment, Upper Extremity (FMA-UE)<br>• 9-Hole Peg Test<br>• Short Form Health Survey (SF-12)
**Other Endpoints**	• Beck Anxiety Inventory (BAI)<br>• Beck Depression Inventory (BDI)<br>• PET
**Statistical Hypothesis**	No formal statistical hypotheses are proposed for this feasibility study.
**Study Phases**	
**Study Phases Summary**	• Baseline<br>• DBS Implant Procedure<br>• Postoperative Recovery (4 weeks)<br>• Rehab Baseline (8 weeks)<br>• DBS Programming (4 weeks) with rehab continuation<br>• Testing: Stimulation of the dentate nucleus + Rehab (16 weeks)<br>• Rehab Follow-up (4 weeks)<br>• Long Term Follow-up<br>• DBS Explant Procedure<br>• Explant Follow-up (4 weeks)
**Rehabilitation Program**	All patients will undergo an outpatient rehabilitation program after postoperative recovery. The frequency and duration of therapy sessions will be two times per week for 1-1.5 hours of treatment time over a 2 hour scheduled contact interval. In addition to the formal outpatient therapy sessions, participants will sign a behavioral contract that obligates them and their

	caregivers to continue with their upper extremity rehabilitation program, as specified by the treating therapist, at home.
**Testing (Test Treatment + Rehab)**	Subjects will receive stimulation of the dentate nucleus area and continue their ongoing rehabilitation program (test treatment + rehab) for 16 weeks.
**Rehab Follow-up**	Once the testing phase (test treatment + rehab) has concluded, investigators will gradually wean OFF the device. This will be to assess if gains achieved during the testing phase will persist without continuous stimulation. Loss of more than 50% of improvements achieved during the testing phase will prompt the investigators to turn the devices back ON. Improvements will be indexed with sub-scores (proximal, distal and total limb) of the Fugl-Meyer Scale. Subjects will continue their ongoing rehabilitation program for 4 weeks.
**Long-term Follow-up and Study Completion**	Once the Rehab Follow-up phase is completed, subjects will be followed until their one-year post implant follow-up. There will be no mandatory structured rehabilitation provided by the study and the device will remain OFF until explanted during this phase. Then the device will be explanted as per study procedure.<br><br>Subjects will have a final follow up visit 6-month post explant. At the conclusion of the 6-month post explant follow-up the subject will have completed this protocol.

CONTENTS

1.0 INTRODUCTION 10
1.1 General Overview 10
1.2 Purpose 10
1.3 Duration of the Investigation 11
1.4 Number of Sites and Subjects 11
1.4.1 Staged Implant 11
2.0 DEVICE DESCRIPTION 11
2.1 Name of Device 11
2.2 Vercise DBS System Principle of Operation 13
2.3 Vercise DBS System and TMS System Regulatory Status 13
2.4 Proposed Indications for Use 13
2.5 Manufacturing information 14
2.6 Labeling 14
3.0 BACKGROUND AND SIGNIFICANCE 14
3.1 Overview of Stroke Epidemiology 14
3.2 Acute Management of Stroke 14
3.3 Management of Risk of Recurrent Stroke 15
3.4 Disability and Recovery from Stroke 15
3.5 Rehabilitation of Post-Stroke Upper Extremity Paresis 16
3.6 Treatments for Upper Extremity Paresis 17
3.6.1 Motor Training 17
3.6.2 Non-invasive Neurostimulation Therapies for Post-Stroke Motor Recovery 17
3.6.3 Epidural Cortical Stimulation 18
3.7 Unmet Medical Need 18
3.8 Deep Brain Stimulation Overview 19
3.9 Clinical Rationale of DBS for Enhancing Post-stroke Motor Recovery 20
4.0 REPORT OF PRIOR INVESTIGATIONS 21
4.1 Literature Review 22
4.2 Summary of Preclinical Studies 24
4.3 Conclusion 26

5.0 STUDY DESIGN 26

5.1 Study Objective 26

5.2 Primary Endpoint 26

5.3 Secondary Endpoints 27

5.3.1 Impairment 27
5.3.2 Activity 28
5.3.3 Participation/Quality of Life 28
5.3.4 Other 28

6.0 SUBJECT SELECTION 29

6.1 Study Patient Population 29

6.1.1 General Inclusion Criteria 29
6.1.2 General Exclusion Criteria 30
6.1.3 Transcranial Magnetic Stimulation Subject Inclusion Criterion 31

6.2 Subject Screening 33

6.3 Subject Enrollment 34

7.0 STUDY VISITS 34

7.1 Study Schedule 34

7.1.1 Schedule of Study Visits 36
7.1.2 Schedule of Study Assessments 36

7.2 Baseline Evaluation 38

7.2.1 Unilateral DBS Electrode Implantation 38
7.2.2 Hospital Discharge 42
7.2.3 Postoperative Recovery Period 42

7.3 Rehabilitation 43

7.3.1 Description of the Rehabilitation Program 43

7.4 Device Programming 44

7.4.1 Transcranial Magnetic Stimulation (TMS) 45
7.4.2 Safety of Transcranial Magnetic Stimulation and DBS 46
7.4.3 Testing phase (stimulation + rehabilitation) 47

7.5 Rehab Follow-up 48

7.6 Long-Term Follow-up: 48

7.7 Explant 49

7.8 Exit of Participation 49

7.8.1 Loss to Follow-Up 49
7.8.2 Subject Withdrawal From Study 49

7.9 Unscheduled Visits 50

7.10 Protocol Deviations 50

8.0 SAFETY REPORTING 50

8.1 Definitions and Classification 50

8.1.1 Adverse Event (AE) 51
8.1.2 Serious Adverse Event (SAE) 51
8.1.3 Device deficiency 52
8.1.4 Adverse Device Effect (ADE) 52
8.1.5 Serious Adverse Device Effect (SADE) 52
8.1.6 Unanticipated (Serious) Adverse Device Effect (UADE) 52
8.1.7 Relatedness to DBS Device 52

8.2 Device Deficiencies 53

8.3 Deaths 53

9.0 DATA MANAGEMENT 53

9.1 Completion of Case Report Forms 53

9.2 Data Review 53

9.3 Data Analysis Plan 54

10.0 STUDY ADMINISTRATION 54

10.1 Data Monitoring Committee 54

10.1.1 DMC Responsibilities 54
10.1.2 Monitoring for Safety 55
10.1.3 DMC Meetings 55
10.1.4 Making Recommendations 56
10.1.5 DMC Decision Guidelines 56

10.2 Study Registration. 57

11.0 SCIENTIFIC SOUNDNESS 57

12.0 RISK/BENEFIT ANALYSIS 57

12.1 Potential Benefits of Electrical Stimulation of the Dentate Nucleus 57

12.2 Potential Risks of Electrical Stimulation of the Dentate Nucleus 57

12.2.1 Risks Associated with Vercise DBS System Implantation Procedure 58
12.2.2 Risks Associated with Vercise DBS System 59
12.2.3 Risks Associated with Deep Brain Stimulation Therapy 61
12.2.4 Risk Minimization 62

12.3 Risk Benefit Analysis 63

13.0 MONITORING PROCEDURES 64

13.1 Monitoring Procedures 64
13.1.1 Monitoring Reports 64
13.2 Final Site Visit 64

14.0 COMPLIANCE 65

14.1 Statement of Compliance 65
14.2 Device Accountability 65
14.3 Sponsor Responsibilities 65
14.3.1 Sponsor Records 66
14.3.2 Sponsor Reports 66
14.3.3 Sponsor Inspections 66
14.4 Investigator Responsibilities 67
14.4.1 Protection of Human Subjects 67
14.4.2 Investigator Records 67
14.4.3 Investigator Reports 68
14.4.4 Investigative Center Inspections 68

15.0 INFORMED CONSENT MATERIALS 70

16.0 REFERENCES 70

List of Tables

Table 1. Description of Devices 12
Table 2. Schedule of Study Visits........ 36
Table 3. Schedule of Study Assessments 37
Table 4. Risks of Deep Brain Stimulation Implant Procedure 59
Table 5. Literature Review on Risk of Recurrent Stroke Post Ischemic Stroke........ 60
Table 6. Investigator and Sponsor Reporting Responsibilities........ 69

List of Figures

Figure 1: DBS Vercise System........ 12
Figure 2: Study Timeline........ 34
Figure 3: Study Flow Diagram 35
Figure 4. Illustration of Patient Position and the Placement of the Head Frame........ 39
Figure 5. Illustration of Electrode Placement for the Dentate Nucleus Target 40
Figure 6. Final DBS System Placement 42

1.0 INTRODUCTION

1.1 General Overview

Stroke is a disease of epidemiological proportions in the industrialized world and a leading cause of long-term disabilities. Approximately 795,000 people in the United States alone suffer strokes every year[1]. While the majority of patients will survive the acute phase, persistent neurological sequelae likely will jeopardize quality of life and productivity, with approximately 50% of survivors still exhibiting some degree of hemiparesis at 6 months after stroke and 30% requiring assistance with activities of daily living.[2] According to the American Heart Association Statistics Committee and Stroke Statistics Subcommittee, the estimated indirect and direct cost of stroke for 2014 is U$ 36.5 billion.[1] These numbers underscore the need for translational research aimed at enhancing motor outcomes after stroke.

The duration and type of rehabilitation provided varies depending on an individual's level of impairment, progress in therapy and other factors such as patient preferences, and insurance coverage for rehabilitation. Deep brain stimulation (DBS) is a treatment that utilizes surgically implanted electrodes and an electrical pulse generator, sometimes called a "brain pacemaker" or neurostimulator, to deliver electrical pulses to specific targeted subcortical brain structures. It is routinely used to manage movement disorders such as Parkinson's disease and essential tremor. In this feasibility study, we propose to evaluate the effects of DBS of the dentate nucleus on motor recovery of chronically hemiparetic patients due to ischemic stroke.

1.2 Purpose

The purpose of this feasibility study is to obtain clinical experience and scientific evidence to evaluate the feasibility, safety and effectiveness of electrical stimulation of the dentate nucleus of the cerebellum for the treatment of chronic upper extremity hemiparesis due to ischemic stroke. The results of this study are expected to provide preliminary data on the risks and benefits of the therapy, and adequate information on which to base decisions regarding the need for, and the design and conduct of, future trials of deep brain stimulation (DBS) of the dentate nucleus of the cerebellum for post-stroke upper extremity hemiparesis.

The goals of this feasibility study are to:

- develop preliminary scientific evidence to evaluate the feasibility of DBS therapy;
- calculate estimates of event rates that can be used for sample size determination for future pivotal studies;
- evaluate the success of procedures for recruitment and enrollment of patients for any future pivotal trials;
- learn about the electrophysiological signatures and imaging correlates that may indicate the ideal location for electrode implantation in future clinical trials. (Based on the experience in DBS for movement disorders, these signatures also are expected to streamline the surgical procedure in the future.)

1.3 Duration of the Investigation

The estimated duration of this feasibility study is 2 years from the time of first subject enrollment to the last follow-up visit for all patients enrolled.

1.4 Number of Sites and Subjects

The study will enroll up to 5 subjects at one investigative surgical site, the Cleveland Clinic (CC). Patients will undergo evaluation, surgery and DBS programming and follow-up at the Cleveland Clinic main campus and hospital. Patients will receive rehabilitation and have follow-up outcome assessments conducted at the Cleveland Clinic.

1.4.1 Staged Implant

The study will be conducted with a staged FDA approval process. Initially up to 5 subjects will be enrolled with the limitation that only one may be implanted with the DBS System. Safety data for the first implanted subject will be reported to the FDA at the following time points:

1. One week after initial implantation surgery
2. After the first exposure to the investigational treatment (DBS + TMS exposure in the Titrate/Programming phase)
3. After the completing of one month of the testing phase in which therapy DBS is being continuously applied.

After the first subject has completed one month of therapy (Stim + rehab) an IDE Supplement will be submitted to the FDA to request expanding the number of subjects that can be implanted with the device. In addition, the protocol may be amended to allow enrollment of subjects that are moderately to severely impaired as defined by a UE Fugl-Meyer of < 47. Initially, the first subject implanted will be more severely impaired as defined by a UE Fugl-Meyer of ≤ 20.

2.0 DEVICE DESCRIPTION

2.1 Name of Device

This study will be conducted with the Boston Scientific Vercise deep brain stimulation system. The Vercise DBS system is used in other investigational stud(ies) under other IDEs. The Food and Drug Administration (FDA) has not approved the Vercise DBS system for marketing and for this patient population and the method of deep brain stimulation utilized in this study. The Vercise™ deep brain stimulation system is a Class III implantable device, and the proposed study is a significant risk study. Therefore, this study requires United States (US) FDA approval of an Investigational Device Exemption (IDE), and local Institutional Review Board (IRB) approval prior to initiating the study.

The Vercise™ deep brain stimulation system is designed to deliver targeted electrical stimulation through implanted electrodes and neurostimulator. The Vercies system components to be used in this study are listed in Table 1.

Table 1. Description of Devices

System	Device Component	Model
Vercise™ Parkinson's Control System	Neurostimulator	Vercise™ Neurostimulator
	DBS Lead	30cm or 45cm
	Extension	55cm
	Accessories	Lead Boot<br>Lead Stop<br>Torque Wrench<br>Suture Sleeve (1cm, 1cm split, 2.3cm 4cm)<br>Remote Control<br>Charger<br>Vercise™ External Trial Stimulator<br>Vercise™ Clinician Programmer<br>OR Cable and Extension<br>IR Interface<br>IR Interface Holder<br>USB Serial Cable<br>Power Converter, International??<br>Tunneling tool (shaft and handle)<br>Stimulator Header Plugs<br>Torque Wrench<br>SureTek™ Burr Hole Cover

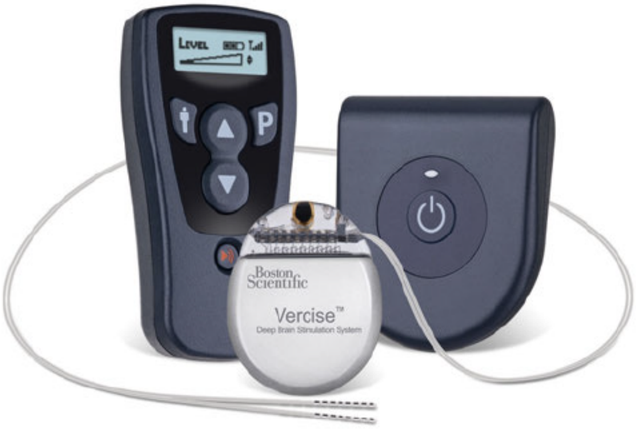

Figure 1: DBS Vercise System

2.2 Vercise DBS System Principle of Operation

For the specific steps on how to operate the Vercise system, refer to the device labeling, (DBS Vercise Physician Manual and Vercise Burr Hole Cover Manual).

Deep brain stimulation is treatment that utilizes surgically implanted electrodes and a neurostimulator to deliver electrical energy to specific targeted structures deep within either one or both hemispheres of the brain.

For the proposed indication the aim is to deliver chronic unilateral electrical stimulation to the dentate nucleus of the cerebellum. The hypothesis proposed here (and demonstrated in our pre-clinical studies) is that stimulation of the dentate will chronically activate the dentate-thalamo-cortical pathway and promote rehabilitation beyond the effects of physical therapy alone. Stimulation is expected to enhance cortical excitability and facilitate plasticity, cortical reorganization and, consequently, motor recovery of upper extremity hemiparesis.

2.3 Vercise DBS System and TMS System Regulatory Status

In 2012, Boston Scientific Corporation announced receipt of CE Mark approval for use of its Vercise™ Deep Brain Stimulation (DBS) System for the treatment of Parkinson's disease.

The Vercise DBS System has both CE Mark and TGA (Australia Therapeutic Goods Administration) approval for the treatment of Parkinson's disease. It also has CE Mark for intractable primary and secondary dystonia, and is available for sale in Europe, Israel, Australia and select countries in Latin America.

More recently, in 2014, Boston Scientific announced receipt of CE Mark for the Vercise™ Deep Brain Stimulation (DBS) System for the treatment of tremor, including the most common form of this movement disorder known as essential tremor (ET).

In the U.S., the Vercise DBS System is investigational and not approved for sale. Boston Scientific's IDE clinical trial (G120075) to support PMA application began enrollment in the U.S. in mid-2013 to evaluate the safety and effectiveness of the Vercise DBS System for the treatment of Parkinson's disease. The use of transcranial magnetic stimulation (TMS) is not approved with DBS and the use of TMS during the study after the DBS system is implanted is investigational.

The TMS System, MagPro R30 Magnetic Stimulator and MCF-B65 Coil is manufactured by Tonia Elektronik A/S. These devices have 510(k) clearances for other intended use. The intended use of TMS used in this protocol is investigational.

2.4 Proposed Indications for Use

The Vercise deep brain stimulation system will be used in this proposed feasibility study in the management of chronic, post-stroke, upper extremity hemiparesis in addition to rehabilitation therapy in individuals 12 to 24 months post stroke who have residual moderate to severe upper extremity motor impairment, defined as a Fugl-Myer Upper Extremity subscale score ≤ 20.

2.5 Manufacturing information

Manufacturing information for the Vercise Deep Brain Stimulation System is available in Boston Scientific's approved IDE G120075.

Manufacturing information for the MagPro R30 Magnetic Stimulator and MCF-B65 Coil is available in Tonica Elektronik A/S cleared 510ks (K 091940 and K061645, K150641 and Master File 1554).

2.6 Labeling

The Vercise DBS System components and the TMS system to be used in this clinical study will bear a label with the following statement: "CAUTION--Investigational device. Limited by Federal (or United States) law to investigational use."

3.0BACKGROUND AND SIGNIFICANCE

3.1 Overview of Stroke Epidemiology

Cerebral vascular accident or stroke is the neurological injury that occurs because of brain ischemia or hemorrhage. The World Health Organization standard definition of stroke is a focal (or at times global) neurological impairment of sudden onset and lasting more than 24 hours (or leading to death) and of presumed vascular origin[3].

The incidence of stroke in the United States is estimated to 795,000 of which approximately 185,000 are recurrent strokes[1, 4]. Of all strokes approximately 85% have an ischemic etiology; intracerebral and subarachnoid hemorrhage account for the remainder. Mortality from stroke is high, and varies with etiology. In persons 45 to 64 years of age, the 30-day mortality rate for ischemic stroke is 8%-12%, and 37%-38% for hemorrhagic stroke[3]. In persons 65 years of age or older the overall 30-day mortality is 12.6%; 8.1% for ischemic strokes and 44.6% for hemorrhagic strokes. Overall, in individuals age 40 years and older, 21% of men and 24% of women are dead one year after a first stroke; 47% of men and 51% of women are dead within 5 years of a first stroke. [5, 6]

Stroke is a leading cause of serious and long-term disability in the United States. The estimated total direct and indirect cost of stroke in the US is $36.5 billion. The mean lifetime cost of an ischemic stroke has been estimated at $140,080 for inpatient care, rehabilitation, and follow-up care for lasting deficits. [1, 4]

3.2 Acute Management of Stroke

Today the approach to management of acute stroke is highly proactive. The goal of acute management of stroke is to ensure rapid diagnosis and treatment. Rapid identification and diagnosis of stroke facilitates the administration of the most effective acute stroke therapies including intravenous tissue plasminogen activator (tPA) for ischemic stroke, early management of blood pressure and other neurointensive care measures, surgical and endovascular options when indicated, management of complications of stroke, and institution of appropriate secondary prevention strategies.

In ischemic stroke, the emphasis in the acute setting is on maximizing reperfusion in order to minimize tissue injury.[7] There are several clinical trials and emerging technologies aimed at facilitating post-stroke rehabilitation. Timely restoration of blood flow to the structurally intact ischemic areas around the infarct improves the potential for limiting the extent of the injury.

3.3 Management of Risk of Recurrent Stroke

Individuals that have experienced an initial ischemic stroke are at high risk for recurrent stroke. The annual risk has been estimated to be anywhere from 3% to 22% with an average of about 15%. Reports of rates of recurrent ischemic stroke vary due to methodological differences in analysis, as well as differences in age, gender, coexistent morbidities and risk factors, and background medical treatments among the cohorts studied. The American Heart Association reports 5-year recurrence rates for individuals that survive a first stroke (of any type) as a function of age, gender and race. These 5-year recurrence rates range from a low of 10% in black men aged 40 to 69 years to a high of 32% for black women over the age of 70 years.

3.4 Disability and Recovery from Stroke

Recovery after stroke displays a nonlinear pattern with the largest improvements occurring within the first three months and improvement gradually stabilizing thereafter[8, 9]. The course of recovery from stroke varies according to the type and severity of the stroke, patient characteristics, and treatment regimen. A majority of stroke survivors, from 50% to 70%, achieve functional independence. However, 15% to 30% of stroke survivors are permanently disabled, and 20% require institutional care at three months post onset.[10, 11]

Up to 85% of stroke survivors experience some degree of upper extremity hemiparesis, and only 25% to 55% of these individuals achieve functional recovery of their arm.[10, 11]

Rehabilitation involves a comprehensive, multidisciplinary effort to utilize medical, therapy, social, vocational, and educational techniques to assist the stroke survivor to achieve maximal recovery. Rehabilitation includes strategies to optimize recovery of neurologic impairment, to teach compensatory strategies and skills for activities of daily living and independent living, and to provide psychosocial support to the patient and their families.

Patients recover after stroke in two ways. First, patients recover through spontaneous recovery of neurological function, through treatments that limit the extent of damage from the stroke, and through other interventions that enhance neurological functioning. Secondly, patients are able to adapt and learn to accomplish their daily activities within the limitations of their neurological impairments.

Rehabilitation typically includes some combination of physical therapy, occupational therapy, speech therapy, family education, and home therapy programs. Therapy may be provided in the acute, sub-acute or outpatient settings.

Rehabilitation techniques directed at reducing motor impairments are based on the concepts of neuroplasticity and reorganization of cerebral activity. While the primary motor cortex is well defined anatomically, there are secondary motor areas that originate motor neural pathways. Improvements in motor function can be promoted by promoting cortical reorganization of the primary motor representation as well as vicariation of function from secondary motor areas including the premotor cortex[12]. Modifications in neural networks are use dependent, and active participation by patients with motor impairments in therapy programs directly influences the process of functional reorganization in the brain to enhance neurological recovery.[12, 13]

The increased availability of advanced neuroimaging techniques such as positron emission tomography (PET) and function magnetic resonance imaging (fMRI) in the last decade has made human research on the mechanisms and biology of motor recovery after stroke possible. New techniques for stroke rehabilitation are emerging that take advantage of advances in understanding of the mechanisms of stroke recovery and the concept of brain plasticity.

3.5 Rehabilitation of Post-Stroke Upper Extremity Paresis

Current practice guidelines for stroke rehabilitation recommend early initiation of rehabilitation once a patient is medically stable, and that patients receive as much therapy "as needed" to recover motor function. Rehabilitation care often requires a multidisciplinary team of experienced professionals. This may include physical medicine, nursing, physical therapy, occupational therapy, kinesiology, speech-language therapy, psychology, and recreational therapy.[14 15]

Rehabilitation for post-stroke upper extremity paresis includes both compensatory and restorative approaches. Compensatory approaches focus on training the individual to utilize alternative strategies and tactics to improve motor function and ability to carry out daily activities. Restorative therapies focus on motor retraining and recruiting surviving areas of the brain to perform the functions of the damaged areas.

There are a variety of rehabilitation techniques available, including neurodevelopmental therapy, sensory motor integration, proprioceptive neuromuscular facilitation, constraint-induced movement therapy, mental imagery and biofeedback. Rehabilitation programs are usually individualized for the patient based on their presenting deficits and medical status. The patient's tolerance for therapy depend on multiple factors including the severity of the stroke, medical stability, cognitive status, communication ability, level of function, and the extent of social support.

In this study, the investigation will not evaluate early rehabilitation strategies for strokes. Instead, we will re-initiate rehabilitation intervention after dentate nucleus DBS implantation in a fashion similar to strategies applied in the post-stroke, chronic outpatient rehabilitation phase. It is the investigator's expectation that chronic dentate nucleus electrical stimulation will open a new window for effectiveness from rehabilitation training in the chronic post-stroke phase.

3.6 Treatments for Upper Extremity Paresis

There are multiple treatment strategies for post-stroke rehabilitation. These include motor training and non-invasive stimulation. Invasive stimulation with epidural stimulation, as discussed below, has been attempted, but has not achieved the desired outcomes to date.

3.6.1 Motor Training

There are several motor training techniques, including task-specific training[16], neurodevelopmental therapy[17], and constraint induced movement therapy (CIMT)[18]. Accumulating evidence over the past decade indicates that task specific training oriented toward repetition with or without shaping can lead to significant improvement in functionally based upper extremity movement capability following stroke[19]. Constraint-induced movement therapy (or CIMT) is a unique form of upper extremity “forced use” therapy and truly represents a subset of functional training. CIMT uses repetitive task practice or adaptive task practice through intense training. This modality has been evaluated in the multi-site EXtremity Constraint Induced Therapy Evalution (EXCITE) Trial. The study showed that CIMT resulted in sustained benefits in patients who had suffered strokes 3 to 9 months prior[20].

3.6.2 Non-invasive Neurostimulation Therapies for Post-Stroke Motor Recovery

The use of electrical stimulation to facilitate post-stroke recovery seems intuitive. The goals can be defined as boosting neural function of the perilesional and other spared areas of the nervous system in order to promote plasticity and neurorehabilitation. Techniques for non-invasive stimulation include functional electrical stimulation (FES), transcranial magnetic stimulation (TMS) and transcranial direct current stimulation (tDCS). Electrostimulation delivered to the peripheral neuromuscular system to improve movement and function following a stroke is an option for the treatment of upper extremity hemiparesis that is under study. A Cochrane meta analysis that reviewed the data from existing trials concluded that while the current data suggests some benefit from electrical stimulation, the current data is insufficient to conclude that the therapy is effective[21]. Currently these therapies are not broadly utilized and remain under research. Repetitive transcranial magnetic stimulation (rTMS) has been under evaluation for several years. Clinical studies published to date involve small numbers of patients, and apply this therapy for the treatment of motor impairment from subcortical strokes[22, 23]. There is uncertainty regarding patient selection and the most appropriate stimulation parameters as well as site of stimulation for clinical application. For example, the optimal frequency for stimulation remains a matter of debate as well as whether stimulation is best applied to the perilesional motor cortex or to secondary cortical areas. Furthermore, contralateral stimulation remains an option but it is unclear if it should be aimed at a particular subpopulation of patients. We have recently reviewed the use, limitations and alternative approaches for TMS and tDCS[12].

3.6.3 Epidural Cortical Stimulation.

Chronic epidural cortical stimulation can be applied in humans as well as in animal models. There are no currently available commercial systems for chronic cortical stimulation in humans. However, clinicians often make off-label use of spinal cord stimulation hardware to implant epidural leads over the motor cortex and connect to implanted pulse generators. The most common utilization of cortical stimulation in the U.S. and Europe is for pain management, following the initial publication by Tsubokawa and collaborators[24]. However, outcomes are limited and reviews have shown that the method is less efficacious than initially thought, particularly for post-stroke pain[25]. Motor cortex stimulation has also been hypothesized to promote perilesional plasticity and to facilitate post-stroke recovery. The approach once held great promise for promoting motor rehabilitation following stroke based on preclinical work in animal models[26-29]. Early phase clinical trials also showed promising [30, 31]; however a large randomized clinical trial failed to meet its intended endpoints[32]. The reason for the lack of success of the phase III trial is not completely clear, though methodological issues may have contributed to the negative results.

It is possible that chronic epidural cortical stimulation is not a viable option for post-stroke motor rehabilitation in humans. The cerebral cortices of the rodent and some non-human primate models (i.e., squirrel monkey) are relatively flat in comparison to the profound convolutions found in the human brain, resulting in a more consistent pattern of organization of the pyramidal and inter-neurons across large expanses of cortex. In humans, the orientation of neurons in relation to the dura can vary dramatically depending on their location relative to the crown or sulcus of a gyri. Cathodal and anodal stimulation have different effects on the cortical surface and the orientation of the neuron plays a key role in determining its response to epidural stimulation.[33, 34] Hence, it is conceivable that in human clinical trials the effects of epidural stimulation had a net neutral effect or negative effect, or perhaps simply did not achieve a necessary volume of effect, due to this variation in the neuron orientation relative to the epidural stimulating electrodes. One of the potential benefits of the approach proposed herein lies in taking advantage of the dentatothalamocortical pathway fibers directing the effects of stimulation to the perilesional cortical area, regardless of the orientation of neurons in the corresponding gyri.

3.7 Unmet Medical Need

Upper extremity hemiparesis initially is seen in up to 85% of patients following a stroke and by 6 months post stroke between 45% and 75% of patients continue to have some degree of upper extremity functional impairment, and as many as 30% to 60% of patients are unable to use their affected arm functionally after discharge from rehabilitation.

In a study of 102 severely impaired acute stroke survivors with almost total upper extremity paresis (mean Upper Extremity Fugal-Myer score of 7 out of a possible 66 points), only 38% showed some recovery of dexterity in the hemiplegic arm and only 4.4% achieved complete functional recovery at six months post stroke. All patients had experienced ischemic strokes in the region of the middle cerebral artery (MCA), and

had participated in a randomized trial of acute rehabilitation approaches involving 5 hours of therapy a week for 20 weeks[35].

Although extensive research is under way on acute interventional therapies aimed at tissue protection and reduction of infarct volume, relatively few efforts have been made at enhancing plasticity and recovery of function in the chronic phase after strokes. The prevalence of stroke survivors in the United States is greater than five million individuals, highlighting the importance of investigation on emerging therapies aimed at alleviating the burden of permanent hemiparesis on individuals, family and society. There is immense need for novel therapies to be tested given the limited benefits observed to-date with existing technologies aimed at promoting post-stroke rehabilitation.

3.8 Deep Brain Stimulation Overview

Deep brain stimulation (DBS) is treatment that utilizes surgically implanted electrodes and a neurostimulator to deliver electrical energy to specific targeted structures deep within either one or both hemispheres of the brain.

Neurologists and neurosurgeons have used microelectrode recording and stimulation since the 1960s to locate and map specific subcortical structures in the basal ganglia, diencephalom and cerebellum. Microelectrode recording has also been extensively performed in the dentate nucleus region that will be targeted in this study.

Early experiences with subcortical electrical stimulation were acquired during stereotactic lesioning procedures such as pallidotomies and thalamotomies. In these procedures, surgeons used depth probes to generate heat by radiofrequency to ablate structures thought to be involved in the pathology of movement disorders such as essential tremor. During thalamotomies for tremor, neurosurgeons would electrically stimulate the thalamic nucleus prior to ablation. The purpose was to observe if electrical stimulation would influence tremors in the awake patient prior to making the permanent, non reversible lesions (the "thalamotomy"). This experience showed pioneers in the field that electrical stimulation at high frequency could control tremors for the short period of time during which stimulation was tested. This indicated that, possibly, chronic electrical stimulation could chronically modulate tremors or other symptoms of movement disorders. The first report of long-term chronic electrical stimulation with DBS therapy for the treatment of tremor was published in 1991 by Drs Benebid and Pollak of the University of Grenoble in France[36]. The first FDA approved use (1997) of DBS was for the treatment of essential tremor or tremor due to Parkinson's disease. Since then DBS has been considered for the treatment of a variety of neurological disorders, and Medtronic's DBS Therapy has been approved for use in the United States by the FDA for:

- Unilateral thalamic stimulation of the ventral intermediate nucleus of the thalamus (VIM) for tremor,
- Bilateral stimulation of the internal globus pallidus (GPi) or the subthalamic nucleus (STN) for Parkinson's Disease
- Unilateral or bilateral stimulation of the internal globus pallidus (GPi) or the subthalamic nucleus (STN) for dystonia, under a Humanitarian Device Exemption.

- Bilateral stimulation of the anterior limb of the internal capsule, AIC, for treatment of chronic, severe, treatment-resistant obsessive compulsive disorder (OCD), under a Humanitarian Device Exemption.

The literature provides a number of additional reports of clinical experience with DBS for treatment of disorders including rare movement disorders, epilepsy, pain, Tourette's syndrome, and Alzheimer's disease.[37-44 45-47]

3.9 Clinical Rationale of DBS for Enhancing Post-stroke Motor Recovery

The proposed IDE feasibility study is designed to evaluate a novel use of DBS for enhancing motor recovery in patients with chronic, upper extremity weakness following an ischemic stroke. The brain target selected for electrical stimulation is the dentate nucleus of the cerebellum. The clinical rationale for the use of DBS and specifically the selection of the dentate nucleus as the stimulation target is based on an understanding of the anatomy and physiology of the brain, and the neurophysiology of motor function.

The cerebral cortex is tightly and reciprocally linked to the cerebellum, with the majority of the fibers decussating at the level of the brainstem to connect with the contralateral hemisphere. The bulk of the projections from the cerebral cortex to the cerebellum is carried by the corticopontocerebellar pathway, with a synapse at the pontine nuclei in the brainstem. This pathway reaches the cerebellum through the middle cerebellar peduncle and its interruption is believed to cause massive deafferentation of the cerebellum. This lack of afferent input is believed to be the cause of decreased metabolism and activity that represent the hallmark of crossed cerebellar diaschisis (CCD)[48-51], as reflected in imaging studies such as positron emission tomography (PET)[52, 53] and single photon emission tomography (SPECT)[54-58]. The corticopontine system is the corticofugal part of this two-step pathway. The projections from the motor cortex reach the pons with the crossover to the contralateral cerebellar hemisphere occurring after this connection6.

The dentatothalamocortical (DTC) pathway, the ascending part of cerebro-cerebellar interactions, is the main connection from the cerebellar hemispheres to the cerebral cortex. The fibers exiting the dentate decussate at the level of the inferior colliculus and surround the contralateral red nucleus (where a minority of the fibers terminates). The majority of the fibers terminate in the ventroposterolateral pars oralis (VPLo), mediodorsal and ventrolateral pars caudalis (VLc) nuclei of the thalamus. These thalamic nuclei project, in a somatotopically organized fashion, to the primary motor cortex as well as premotor and parietal regions. The dentatothalamic projections were once thought to project only to the primary motor cortex. However, more recent elegant studies using virus retrograde labeling in the primate brain demonstrated that the projections extend to the premotor and parietal regions[59-61]. This is important for our research because stimulation carried by the dentatothalamocortical pathway would not likely produce much benefit if carried only to the motor cortex, which is expected to be injured by the MCA infarct that produces hemiparesis. Because the

dentatothalamocortical pathway in fact projects to prefrontal and parietal areas, it can therefore carry stimulation to areas that are potentially preserved after MCA infarction.

At the pontocerebellar level, the projections from the pontine nuclei to the cerebellum are mossy fibers, with excitatory input to the granule fibers (and consequently to the Purkinje cells). There are also excitatory collateral branches to the deep cerebellar nuclei. The output of the cerebellar cortex to the deep nuclei is inhibitory, mediated by the Purkinje cells. The dentate nucleus, however, maintains an excitatory discharge pattern. This highly active output may be driven by extra-cerebellar excitatory input to the nucleus that overcomes the inhibitory input from the cerebellar cortex. In the scenario of disruption of the corticopontocerebellar pathway from a hemispheric stroke, part of the excitatory input to the cerebellar hemisphere will be damaged, accounting for the reduction of metabolism that is translated as CCD. The dentate nucleus also loses the excitatory input from the collateral contribution. The reduced excitatory output from the dentate nucleus has been linked to worsened motor outcome seen in patients with CCD[62]. A central part of the rationale for our hypothesis is based on CCD and its negative effect on motor outcomes. If reduced cerebello-cerebral output results in poor motor outcomes, it is possible that enhancing the cerebello-cerebro output (or normalizing it) may enhance motor outcomes.

The dentate nucleus was selected as the primary target in this study in order to activate the output of the DTC pathway and, consequently, enhance cortical excitability in individuals recovering from ischemic strokes. An alternative target for this therapeutic approach would be the motor thalamus. However in order to encompass the entirety of the influence of the DTC pathway, at least three thalamic nuclei would have to be stimulated simultaneously (MD, VPLo and VLc). Additionally, our approach aimed at the dentate may allow us to influence cortical excitability not only directly through the DTC pathway but also indirectly through enhancement of intrinsic thalamocortical rhythms. It is possible that focal thalamic stimulation alone could result in a more direct, "meaningless" stimulation of the cortex. Furthermore, the concentration of DTC pathway fibers at the dentate nucleus output region, projecting to the motor, premotor and parietal cortices, makes this target an anatomically advantageous node for targeting with a single DBS electrode, thus reducing risk associated with surgical intervention. For a detailed review on the rationale for stimulating the cerebellothalamocortical pathway to promote motor recovery please refer to Machado and Baker, 2012[63].

4.0 REPORT OF PRIOR INVESTIGATIONS

Clinical investigation of DBS in the cerebellar dentate nucleus for the treatment of post-stroke upper extremity hemiparesis is in the feasibility stage of development. We have conducted several preclinical studies to assess the efficacy of this emerging therapy as well as possible mechanisms underlying the motor improvements that have been observed.

In addition, a thorough literature review was conducted to learn of prior studies reporting safety data related to stereotactic targeting the cerebellar nuclei and posterior fossa approaches. This review provides preliminary evidence for the safety of the proposed deep brain stimulation therapy.

4.1 Literature Review.

Human stereotactic neurosurgery is a broad field within neurosurgery, dating back to the late 1940's when Spiegel and Wycis pioneered the first apparatus for stereotactic neurosurgery in humans[64-67]. For decades, the most common stereotactic procedures involved insertion of probes or electrodes for lesioning selected brain targets. Most commonly, the purpose was to ablate a brain nucleus involved in the pathophysiology of a movement disorder such as essential tremor or Parkinson's disease. A few neurosurgeons were key to the development of pallidotomies and thalamotomies, procedures that were popularized in the 1960's and 1970's and are still used today. Among many, the works of Hassler and Dieckman[68-75], Leksell and Laitinen,[71, 76, 77] Talairach[78, 79] can be highlighted.

In the 1980's, chronic subcortical stimulation (deep brain stimulation) was pioneered by Benabid and colleagues in Grenoble, France[36, 80]. During the past two decades, deep brain stimulation gradually replaced stereotactic lesioning procedures as the method of choice for the surgical treatment of movement disorders. The reason for this technical migration was not only the exploration of new cerebral targets such as the subthalamic nucleus but also, <u>safety</u>. Direct comparisons have been made between thalamotomies and deep brain stimulation of the ventral intermedial nucleus (VIM) for the treatment of tremor. Although the efficacy is similar, the rate of complications with deep brain stimulation is significantly lower, as demonstrated by Tasker in 1997[81]. A 2001 study compared a historical series of stereotactic thalamotomies to a cohort of patients with VIM DBS[82]. Although the effects in tremor suppression were very similar between both groups, complications were more common among patients with thalamotomies, particularly intracerebral hemorrhages (35% vs. 0%). Likewise, cognitive deterioration and hemiparesis occurred, respectively, in 29% and 12% of patients who had undergone thalamotomies, but in none of those with thalamic stimulation. For this feasibility study IDE proposal, the importance of Tasker's and Pahwa's studies is to indicate that, although ablative stereotactic procedures such as thalamotomies can be considered safe, stimulation is likely safer.

The surgical target for the present study is the region of the cerebellar dentate nucleus. Although there is only limited literature documenting the safety of implantation of deep brain stimulation electrodes in this region, a large body of evidence exists for the safety of ablative procedures of the dentate nucleus (dentatotomy).

Surgical exploration of the dentate nucleus of the cerebellum in humans was pioneered and developed by Siegfried, as early as 1968.[83] The purpose of these surgical procedures was not to improve motor function (as is the case in the present study) but, rather, to alleviate spasticity and hyperkinetic disorders. A total of 72 dentatotomies for spasticity were analysed. No neurological morbidity to dentatotomy was observed "as long as the lesion does not extend to the medial nuclei." [83, 84] Although mistargetting is a small but present risk in stereotactic neurosurgery, a permanent lesion causes a non reversible effect – as probably seen by Siegfried in lesions that were too medial. Deep brain stimulation is reversible – an adverse effect related to stimulation, when noted, can be reversed. In addition to establishing outcomes, Siegfried's experience also provides valuable knowledge of the human functional anatomy.

These studies report the safety and feasibility of performing radiofrequency intentional ablations of the dentate. Much like the experience with deep brain stimulation and ablation for the thalamus for movement disorders, it is expected that deep brain stimulation may be a safer method of targeting the cerebellar nucleus than ablative procedures. The analogy can also be traced in surgery for psychiatric disorders where DBS of the ventral striatum and anterior limb of the internal capsule (now approved under a Humanitarian Device Exemption) was found to be as safe or safer than lesions performed by radiofrequency ablation or radiosurgery in the anterior limb of the internal capsule.

Functional explorations of the dentate nucleus in humans were also performed and elegantly reported by Slaughter and Nashold[85-88]. These authors evaluated the deep cerebellar structures with microelectrodes and macroelectrodes, demonstrating the feasibility and safety of recording from this region in patients.

Chronic electrical stimulation of the dentate nucleus was first reported by Schvarcz in 1980.[89] The initial experience in four patients demonstrated good efficacy in improving spasticity (as previously seen with dentatotomies), with no complications reported. In Schvarcz et al 1980(6), the authors report on one patient with cerebral palsy who was implanted via a posterior fossa approach (radiography indicates an approach similar to the approach in this study) with the patient in the sitting position. The report does not indicate the exact model of hardware other than that it was a "platinum multipolar electrode array" by Medtronic. The authors indicate that "acute stimulation produced a noticeable improvement (in spasticity) and the patient was percutaneously stimulated for 10 weeks. Pre and postoperative neurological, psychiatric and speech examinations were performed independently. Posture, balance and spasticity were much improved. Speech was improved. Psychological testing showed "improved psychological status. Higher intellectual performances were improvement, alertness and concentration abilities were increased and verbal output was significantly enhanced"

These findings were later corroborated by the same group, in a larger series of 22 patients.[90-92] In the study by Schvarcz et al, 1982, stimulation was initially tested for 8-12 weeks with an externalized lead and the system was then internalized. The report does not specify the surgical approach other than to indicate that the patients were operated in the sitting position. We have assumed that as for their 1980 study the approach was also through the posterior fossa although a radiograph is not shown in the second paper. The report does not indicate the exact model of hardware that was utilized other than to indicate that the device was from Medtronic. Clinical benefits were measured by electrophysiological measures (EMG) but long-term clinical outcomes are not reported.

The use of modern deep brain stimulation hardware in deep cerebellar structures was reported in 2003 by Galanda and Horvarth. A series of 3 patients showed that stimulation was effective in alleviating spasticity. No severe complications were reported. The authors noted reversible improvements in mood associated with stimulation.[93] In Galanda and Horvath 2003(8), the authors report on three patients who underwent deep cerebellar stimulation for 3, 8 and 29 months. The surgical approach was via the posterior fossa (radiography shows an approach that is equivalent to the

present study) with a stereotactic headframe. The patients were implanted with Medtronic 3387 lead models and then internalized with a Soletra Model, Medtronic. Monopolar stimulation was tested at 100 Hz, 500 microseconds and up to 4V. The authors increase stimulation until postural changes were aggravated and then reduced the amplitude, resulting in a transient pleasurable sensation and reduction in spasticity. Chronic stimulation with the internalized system was set to 185V, 210 microseconds and 0.5-2.5V. The authors report no complications and report improvements in mood and posture, spasticity. Activities of daily living were improved.

A follow-up study reported on one additional patient and the long term effects of deep cerebellar stimulation in the previously implanted individuals[94]. Chronic implantation of electrodes was accomplished via a suboccipital (posterior fossa) approach, as planned in this feasibility study. The report by the same authors in 2007(9) is similar. The surgery was also done via a suboccipital approach and aimed at the deep cerebellar nuclei and anterior lobe region. The patients were implanted with Medtronic leads model 3387 or 3389 and Medtronic pulse generators were also by Medtronic model Soletra or Kinetra. Patients were implanted either unilaterally or bilaterally. The surgical approach was the same and the amplitudes and settings were the same as the prior publication. The authors concluded that “chronic stimulation of the anterior lobe of the cerebellum seems to be an effective and safe treatment for patients with cerebral palsy”.

4.2 Summary of Preclinical Studies

The Machado laboratory at the Lerner Research Institute has conducted several studies in animal models of stroke to assess the effects of chronic deep cerebellar stimulation on motor recovery as well as possible neurophysiological mechanisms underlying the observed effects.

There are extensively validated models of stroke in the rodent model, including behavioral measures. The rodent stroke model have been well characterized [95-100] for producing predictable and stable motor deficits and the methods for measuring motor function - including skilled reaching - in the rodent model have been shown to be reproducible, reliable and to be sensitive to the effects of treatment [95-109]. The preclinical data presented stem from experimentation that utilized validated methods for both inducing a stroke [95 97-102 110] as well as validated measures for measuring motor outcome after stroke [99 104, 105 107-109 111, 112]. All techniques utilized for assessing the mechanisms underlying the observed effects have also been reported in the neuroscience literature.

The dentate nucleus anatomy, physiology and projections have been very well characterized in the rodent model and correlate strongly with the anatomy, physiology and projections in the primate [59, 113-118]. In other words, it is possible to produce reliable changes in the activity and excitability of the contralateral hemisphere in the rodent model in a fashion similar to that already demonstrated in the non-human primate model. Dr. Rispal-padel studied the baboon models and demonstrated that stimulation of the dentate nucleus produced activation of the contralateral cerebral hemisphere [114 119 120]. We have reproduced and expanded on these findings in the rodent model. We

demonstrated that stimulation of the lateral cerebellar (dentate) nucleus can produce strong activation of the contralateral hemisphere, thus establishing the functional correlation between the cerebello-thalamo-cortical pathway of the primate and the rodent [121, 122]. We have further studied the rodent electrophysiology and relevance of the dentato-thalamocortical pathway to show that the effects of stimulation on cortical excitability are frequency-dependent and that stimulation can sustainably enhance cortical excitability over time. These findings are strong corroboration to the presence and physiological relevance for this pathway in the rodent as it is for the human and primate.

Our first study in the rodent model of ischemia was published in 2009[38]. The study assessed the effects of chronic stimulation of the lateral cerebellar nucleus (LCN), the equivalent of the dentate nucleus in primates, in Wistar rats that had suffered and survived large middle cerebral artery infarctions. The goal of these studies are to evaluate if chronic stimulation can promote recovery after well-established strokes rather than attempt to reduce the size of impact of the initial injury. For this reason, animals that survive the initial injury are allowed to recover for two weeks before stimulation is initiated. In this first proof of principle study we assessed the effects of chronic stimulation at 20 Hz, 50 Hz or 100 Hz compared to sham (electrode implanted but stimulation not activated). All animals underwent training in a motor task (the Montoya Staircase Task) prior to stroke and then underwent daily training in the same task during the stimulation period. While animals receiving 100 Hz chronic stimulation of the LCN showed no signs of improved recovery compared to SHAM, animals receiving stimulation at 50 Hz showed a trend for better outcomes. However, the best results were observed with stimulation at 20 Hz, which was associated with significantly better recovery than training alone (sham). The results not only pointed towards a first demonstration of viability for this approach, it also pointed that the results of stimulation were clearly frequency-dependent.

Because the frequency-dependent effects of stimulation of the DTC were poorly characterized in the literature, we conducted experiments specifically aimed at addressing the effects of LCN stimulation on cortical excitability, indexed by the magnitude of intracortical microstimulation motor evoked potentials[121, 122]. Animals implanted with deep cerebellar leads in the LCN underwent continuous 10-min blocks of stimulation at various frequencies separated by 10-min blocks of no stimulation. The following frequencies were tested in a pseudorandom order: 10, 20, 30, 40, 50 and 100 Hz. While the net effects of LCN stimulation at 100 Hz were net inhibitory (reduced the amplitude of motor evoked potentials) frequencies of 50 Hz and below had an excitatory effect. Specifically, stimulation at 30 Hz produced the greatest and most sustained effects on cortical excitability[121].

The next step of experimentation was to test the long-term effects of 30 Hz stimulation on long-term motor outcomes. The purpose of this experimentation was three fold: a) to assess the reproducibility of effects of chronic LCN stimulation on post-stroke motor recovery; b) assess the effects of stimulation specifically set at 30 Hz, given that it produced the most robust and sustained effects on cortical excitability; c) evaluate the synergistic effects of chronic stimulation with motor training. In prior experiments all

animals underwent both motor training and stimulation (or sham) but stimulation had not been delivered *during* motor training. This study[123] showed that a) the results were reproducible and animals receiving stimulation recovered to a significantly greater extent than animals undergoing motor training alone; b) 30 Hz stimulation was associated with excellent recovery of motor function, animals returned to the pre-stroke baseline in the motor task and c) stimulation was well tolerated during motor training.

Given the consistency of the behavioral improvements associated with chronic stimulation of the LCN, our group then dedicated its efforts to assess the mechanisms underlying the effects of stimulation. In this recent work[124] it was found that a) chronic LCN stimulation enhances motor recovery, further reproducing the prior results once again; b) that chronic LCN stimulation promotes the expression of markers of long-term potentiation in the perilesional cortex compared to sham; c) that recovery and expression of such markers are associated with repairing of motor representation of the proximal and distal forepaw in the perilesional cortex and d) that chronic stimulation is associated with synaptogenesis.

4.3 Conclusion

Together, the preclinical studies indicate that chronic stimulation has reproducible effects in promoting motor recovery after cortical ischemia in the rodent model. Furthermore, it suggests that the mechanisms underlying behavioral improvements are likely involve electrophysiological facilitation with enhancement of cortical excitability as well as promotion of perilesional reorganization and cortical plasticity.

5.0 STUDY DESIGN

5.1 Study Objective

This is a prospective, open-label, non-randomized, first in human, early feasibility study. The objective of this study is to obtain preliminary data on the safety and feasibility of deep brain stimulation in the dentate nucleus area of the cerebellum when used to treat patients with chronic, moderate to severe, upper extremity hemiparesis due to ischemic stroke.

5.2 Primary Endpoint

The primary endpoint will be the incidence of all serious adverse events, including Serious Adverse Events (SAEs), Serious Adverse Device Events (SADEs), and Unanticipated (Serious) Adverse Device Events (UADE), from the time of enrollment through follow-up. The incidence of all UADE rates will be categorized according to procedure, device, and stimulation.

All non-serious adverse events will also be tabulated and reported.

5.3 Secondary Endpoints

To characterize how the investigative treatment (DBS + Rehab) effects patients' upper extremity motor function and quality of life, a variety of tests will be performed at baseline and repeated at selected time points during the study.

The assessments will evaluate impairments, activity limitations and participation restrictions which are World Health Organization's (WHO) framework for measuring health and disability based on the International Classification of Functioning, Disability and Health (IFC).

5.3.1 Impairment

Fugl-Meyer Assessment, Upper Extremity (FMA-UE)

The FMA-UE is a disease specific impairment index designed specifically for assessment of recovery from post-stroke hemiplegia. The scale has 33 items assessing upper extremity impairment. Each item is scored as 0 = cannot perform, 1 = performs partially, or 2 = performs fully. A total score of 66 points indicates normal upper extremity motor function, and a score of 0 represents complete motor impairment.

The FMA-UE is a highly recommended instrument for stroke assessment by the Neurology Section of the American Physical Therapy Association's Stroke Taskforce (StrokEDGE) Reliability. Validity has been assessed[125-129] and norms for stoke are available[130].

9-Hole Peg Test

The bilateral 9 Hole Peg Test (9-HPT) is a simple, timed test of fine motor coordination. Reliability and validity have been assessed[131] and norms for stoke are available[132]. The test involves the subject placing 9 dowels (9 mm in diameter and 32 mm long) in 9 holes. Subjects are scored on the amount of time it takes to place and remove all nine pegs. Two scores are collected, one for each hand. An iPad Based disability assessment tool will be used to determine the exact time of insertion and removal of each pin and the total time to complete one cycle[133].

Bilateral Box and Block Test

The Box and Block Test (BBT) is a standard assessment tool used by occupational therapists to assess gross manual dexterity. The score on the test represents the number of blocks that can be transported from one compartment of a box to another within one minute, and requires that the participant extend the wrist to both retrieve and place the blocks from one box to another. BBT has demonstrated excellent test-retest reliability for acute and chronic stroke[131] plus excellent interrater reliability for upper extremity paresis[125].

Bimanual Grip Test

Kinetic features of manual dexterity and fine motor control during a task that resembles an activity of daily living will assess level of recovery in bimanual function. A two-transducer system will be used to evaluate a realistic and frequently performed

bimanual task, that is, opening and closing of a jar using different configurations of the hands to open the lid and stabilize the jar[7, 134-136].

5.3.2 Activity

Arm Motor Ability Test (AMAT)

The AMAT is designed to evaluate qualitative and quantitative aspects of an individual's ability to use their upper extremity in the execution of activities of daily living. The AMAT includes 13 ADL activities involving one to three component tasks or movement segments. Each of tasks is timed and rated according based on the extent to which the impaired extremity was used to complete the task (aka functional ability), and the quality of the movement. AMAT is a recommended instrument for stroke assessment by the Neurology Section of the American Physical Therapy Association's Stroke Taskforce (StrokEDGE) with excellent test-retest reliability and interrator reliability[137] and concurrent validity with the Fugl-Meyer Assessment[138].

5.3.3 Participation/Quality of Life

Short Form Health Survey (SF-12)

The Short Form Health Survey is a patient-reported survey of patient health. Scores from the 12 questions result in two scales of mental and physical functioning and overall health-related quality of life. For stoke norms have been established[139] and reliability[140] and consistency[139] validity[141]. Short form Health Survey is a recommended instrument for stroke assessment by the Neurology Section of the American Physical Therapy Association's Stroke Taskforce (StrokEDGE).

EuroQol (EQ-5D)

The EuroQol (EQ-5D) is a standardized instrument for use as a measure of health outcome. The EQ-5D captures health-related quality of life states consisting mobility, self-care, usual activities, pain/discomfort, anxiety/depression. EQ-5D has been assessed from stoke[142].

5.3.4 Other

Beck Anxiety Inventory (BAI)

The Beck Anxiety Inventory (BAI) is a 21-question multiple-choice self-report inventory that is used for measuring the severity of an individual's anxiety.

Beck Depression Inventory (BDI)

The Beck Depression Inventory (BDI) is a 21-question multiple-choice self-report inventory, one of the most widely used instruments for measuring the severity of depression. Normative data and internal consistency has been established for the BDI for both Acute[143, 144] and Chronic Stroke[143].

Positron Emission Tomography (PET)

PET studies will be used as an exploratory, measure to assess possible mechanisms of post-stroke motor function recovery and possible mechanisms underlying the effects of deep brain stimulation of the cerebellar dentate nucleus.

Although this part of the investigation is not expected to provide direct beneficence to each patient, we anticipate that it may provide important information regarding the neural networks that participate in post-stroke motor recovery and the effects of DBS. This exploration of mechanism may inform the optimization of the intervention. It is possible, however, that due to BDS artifact, images acquired will not be useful in the research. The investigators will work to filter the artifact as much as possible in order to make this data scientifically meaningful.

PET will be acquired at the following phases:

- At baseline, prior to DBS lead implantation
- During the rehabilitation phase
- During the rehabilitation + stimulation phase
- During the rehabilitation follow up phase

6.0SUBJECT SELECTION

6.1 Study Patient Population

Subject will not participate in any study-specific tests or procedures before written informed consent. The study will begin only after the study has received IDE approval from the FDA and Institutional Review Board approval.

Subjects participating in the study shall be medically and neurologically stable individuals who have experienced an ischemic stroke 12 to 24 months and who continue to have residual, severe, unilateral, upper extremity hemiparesis. Severity of upper extremity hemiparesis is operationally defined as a score of ≤ 20 on the upper extremity subscale of the Fugl-Meyer Assessment (FMA-UE).[1]

Subjects will be selected to participate in the study based on the inclusion and exclusion criteria described below. Subjects will not be excluded on the basis of gender or minority status.

6.1.1 General Inclusion Criteria

Subjects must meet all of the following general inclusion criteria.

1. First-time ischemic stroke 12 to 24 months (365 to 720 days)[2] prior to implant. The index stroke must have been documented by computerized tomography (CT) or magnetic resonance imaging (MRI).
2. Unilateral stroke in the territory of the middle cerebral artery (MCA) sparing the diencephalon the basal ganglia.

[1] Revised as per FDA feedback, August 27, 2015 – Section 2.2.6 of this IDE
[2] Revised as per FDA feedback, August 27, 2015 – Section 2.2.6 of this IDE

3. 40 to 70 years of age.[3]
4. Transcranial Magnetic Stimulation criterion: Ability to elicit a muscle evoked potential (MEP) in response to TMS delivered to scalp. In a contracted state of the paretic muscle (ranging from 20-50% of maximum voluntary contraction), the ability to evoke reliable criterion MEPs (≥ 100µV in 5/10 trials).
5. Medically and neurologically stable as determined by the investigator based on the medical history, physical and neurological examination.
6. Severe residual unilateral upper-extremity hemiparesis defined as ≤ 20 on the upper extremity subscale of the Fugl- Meyer Assessment (FMA-UE) of the affected side. The FMA-UE shall remain 25 or below (FMA-UE ≤ 25) after baseline rehab in order to be eligible for implant.
7. A score ≥ 1 on the FMA-UE elbow flexion, elbow extension, or finger mass.
8. A Modified Rankin Scale score (mRS) < 4.
9. Absent or moderate spasticity in affected limb in any region (shoulder internal rotators and adductors, elbow flexors, wrist flexors or finger flexors) defined as a score of <4 on the modified Ashworth Spasticity Scale.
10. A score of ≥24 on the Mini Mental State Examination.

6.1.2 General Exclusion Criteria

The subject must not meet any of the following general exclusion criteria.

11. Primary hemorrhagic stroke or major hemorrhagic transformation (Note: Individuals who experience an ischemic stroke with minor hemosiderin in the parenchyma are not excluded.)
12. Any progressive neurological or physical condition other than the index stroke impairing function of the target extremity.
13. Moderate to severe hemispatial neglect or anosognosia involving the affected side of the body.
14. Any other neurological condition that could reduce the safety of study participation including central nervous system vasculitis, intracranial tumor, intracranial aneurysm, multiple sclerosis, or arteriovenous malformations.
15. Pain in the affected limb greater than or equal to 5 on a 0-10 NRS.
16. Evidence of a severe sensory deficit as measured by a score of 2 on the Sensory item (item 8) of the NIH Stroke Scale.
17. Unable to discontinue anticlotting therapy (i.e. antiplatelets and/or anticoagulants) at least 10 days prior to surgery. In the event of a hemorrhagic complication, resuming anticoagulation may be contraindicated for several months.

[3] Revised as per FDA feedback, August 27, 2015 – Section 2.2.6 of this IDE

18. Seizures since the time of stoke, with or without use of antiepileptic agents.
19. Diagnosis of an underlying seizure disorder or epilepsy
20. Change in oral spasticity medications 2 weeks prior to consent or Botox injections in the affected arm within 4 months prior to consent, and/or intention to initiate anti-spasticity medications or botox injections during study follow-up through 12 months post implantation.
21. Major active psychiatric illness that may interfere with treatment, such as psychotic disorders or severe personality disorders.
22. Untreated or inadequately treated depression defined by a score of 20 or greater on the Beck Depression Inventory-II at the time of enrolment.
23. A diagnosis of dementia.
24. Chronic, uncontrolled high blood pressure, history of severe and unmanaged cardiovascular disorder.
25. Contraindication to magnetic resonance (MR) imaging, e.g. weight incompatible with scanner, implanted metallic devices or electrical devices (pacemaker, defibrillator, spinal cord stimulator).
26. Severe and poorly managed medical disorders that, in the opinion of the PI, requires exclusion from the study due to expected risk likely greater than expected for the stroke population.
27. Enrolled in another device, biologic or pharmaceutical study within 30 days of consent in the current study, (i.e. patient cannot be enrolled if participation in another study was not completed at least 30 days prior to consent.)
28. Non-pregnant women. Women of childbearing potential must be using acceptable forms of contraception. Pregnancy will result in exclusion or discontinuation from the study.
29. Undergone a decompressive hemicraniectomy.
30. Patient has significant chronic small vessel ischemic disease, vertebrobasilar vascular disease, and/or any other structural abnormalities of the cerebellum, cerebellar peduncles, and brain stem that would preclude safe placement of the DBS lead.
31. Patient has a condition that, in the opinion of the investigator, would significantly increase the risk for interfere with study compliance, safety or outcome.

6.1.3 Transcranial Magnetic Stimulation Subject Inclusion Criterion

Transcranial magnetic stimulation (TMS) is a non-invasive method for mapping cortical motor representation. When TMS is used over the motor cortex at low stimulus intensities, it indirectly stimulates the corticospinal tract.[13, 145] This stimulation can be recorded as motor-evoked potentials via surface or indwelling electrodes at the target

peripheral nerves. The proposed study will utilize the MagPro R30 mangetic stimulator for the TMS testing.[146]

TMS is included as an inclusion criterion in order to select subjects most likely to benefit from the proposed feasibility study of DBS therapy in post-stroke survivors. It is expected that verifying the integrity of the corticospinal tract (CST) with TMS will select subjects that are most likely to benefit from enhanced cortical excitability produced by DBS.

TMS Inclusion Criterion

The extensor digitorum communis (EDC) of the impaired upper extremity is the target muscle. TMS stimulation will be applied to the ipsilesional hemisphere (contralateral to the impaired limb). Single-pulse TMS (Magpro) will be delivered using a figure-of-eight coil . The coil will be placed tangentially on the scalp with the handle oriented backwards and laterally at 45° from mid-sagittal axis. Patient's MRI will be used to localize and guide the coil using frameless stereotaxy. Stereotaxic localization of the coil will help ensure that application of TMS is repeatable and consistent. The location specifically would lie in the motor cortex. In this region, we would identify the location of the "hot spot". The "hot spot" is defined as the scalp site where single pulse stimulation (.2 Hz) results in reliable muscle evoked potentials (MEPs) (criterion: >=100μV in 5/10 trials) in the active state of the muscle (20-50% of maximum voluntary contraction) at the lowest intensity. Closely spaced surface EMG electrodes will be placed over the mid-belly of the target muscle.

An individual meets the inclusion criterion if there is an ability to elicit MEPs that meet the above criterion. An inability to elicit MEPs that meets the above criterion at any location over the ipsilesional motor cortex at maximum intensity of the device will exclude the potential candidate from being enrolled in the study. A seizer at any point during the testing of the TMS inclusion criteria will discontinue all further testing and exclude the potential candidate from being enrolled. Since patients below UE FMA <20 will recruited as well, it is likely that such patients EDC may not evoke any MEP. In such cases, electrodes will be applied to proximal muscles that are more likely to have spared pathways in patients with severe damage. We will elicit MEPs in triceps and middle deltoid muscles as alternatives. Electrodes will be placed over the muscle belly at the junction of the middle and the lower third of the muscle. Electrodes will be applied in a direction that is parallel to the direction of the muscle fibers.

TMS Procedure

Single-pulse TMS (Magpro) will be delivered using a figure-of-eight coil (70mm, MagPro). The coil will be placed tangentially on the scalp with the handle oriented backwards and laterally at 45° from mid-sagittal axis. Patient's MRI will be used to localize and guide the coil using frameless stereotaxy. The software will register the MRI with patient's cranial landmarks. Stereotaxic localization of coil will ensure that application of TMS is repeatable and consistent.

MT will be determined for the contralateral muscle as explained in original guidelines for use of TMS in motor systems. While patients rest their hands on a flat surface, TMS-evoked responses, called motor evoked potentials (MEP), will be recorded using

surface electromyography via bipolar electrodes (silver-silver chloride, 8 mm diameter) positioned over muscle belly. The skin overlying the forearm will be cleansed and rubbed briskly to reduce inter-electrode skin impedance to below 10 Kohm. A ground electrode will be secured over the acromion after the skin is prepared so differences in impedance between each ground electrode to reference electrode array is less than 5 Kohm.

Essentially, using stereotactic guidance and patient's MRI, TMS would be delivered to a number of candidate sites located in the motor cortices. These sites will be targeted one by one at intensities starting from 50% (expressed as 50% of the maximum output of the TMS device). Intensity will be increased till a site is found to elicit criterion-level MEPs (criterion: >=100μV in 5/10 trials) in the contralateral muscle. Intensity will be titrated so the lowest TMS intensity that generates criterion-level MEPs is identified. MT will be defined as the lowest TMS intensity that generates criterion-level, minimally perceptible MEPs in the muscle. The site that elicits criterion-level MEPs at MT intensity will be called the 'hotspot'. In patients with severe paresis, MT may not be elicited in resting state of the muscle; in this case, patients will be asked to contract their paretic muscle (20-50% contraction) to facilitate elicitation of MEPs. In such cases, the lowest intensity required to generate criterion-level MEPs in the paretic muscles will be termed active MT or aMT. Location of 'hotspot' and its position on patent's MRI will be registered with the stereotactic software so that at each visit we can reliably target the hotspot. With treatment and recovery over time, location of hotspot can shift/reorganize, and intensity of MT/aMT can adapt. To account for this possibility, besides targeting original locations using original MT values, changes in location of hotspot and changes in MT will also be identified to reliably track recovery.

MRI-guided stereotactic software ensures consistency in application of TMS. The software registers patient's head, and their MRI with the location of the coil in 3-D space. As such, the software informs the investigator of real-time location of all three elements. The software provides constant information about displacement of the coil in 3 axes (x, y, and z) and its rotation in 3 axes, i.e. yaw, pitch, and roll. This information is offered as graphical illustrations of real-time relation between elements, and as data reflecting the degree of mismatch, if any. Once a hotspot is identified and labeled as a target for TMS application, then the stereotactic software ensures that there is no linear displacement or rotation of the coil in any plane with respect to the original targeting of the site. If there is any displacement or rotation, then the software alerts the investigator by showing 'in red' as to what and where the error lies. Errors and their corrections are all displayed in real-time so adjustments can be made online during testing.

6.2 Subject Screening

All potential study subjects will be approached for screening in the study. It is expected that eligible participants will be competent to provide informed consent. Each potential study subject will be given an explanation of the study, instructed regarding the screening process and any required testing, educated on the possible risks and benefits to participating, and be asked to provide written informed consent.

An independent consent monitor will conduct a separate interview to verify that patients understand the consent well. This will provide an added layer of safety that is good in a first-in-human study.[4]

Written informed consent will be obtained prior to any study-specific testing. Study personnel will explain that even after written informed consent is provided, subsequent MRI or other testing may demonstrate that the candidate is not a suitable subject for DBS treatment and the candidate will not continue participation in the study

6.3 Subject Enrollment

A subject is enrolled in the study after they have signed the informed consent, and it has been determined by the investigator that they meet all of the inclusion criteria and none of the exclusion criteria.

7.0STUDY VISITS

7.1 Study Schedule

Figure 2 provides a study timeline and provides a study flow diagram from the point of subject consent through completion of follow-up visits. Schedule of Study Visits

Table 2 displays the schedule of study visits during the study phases.

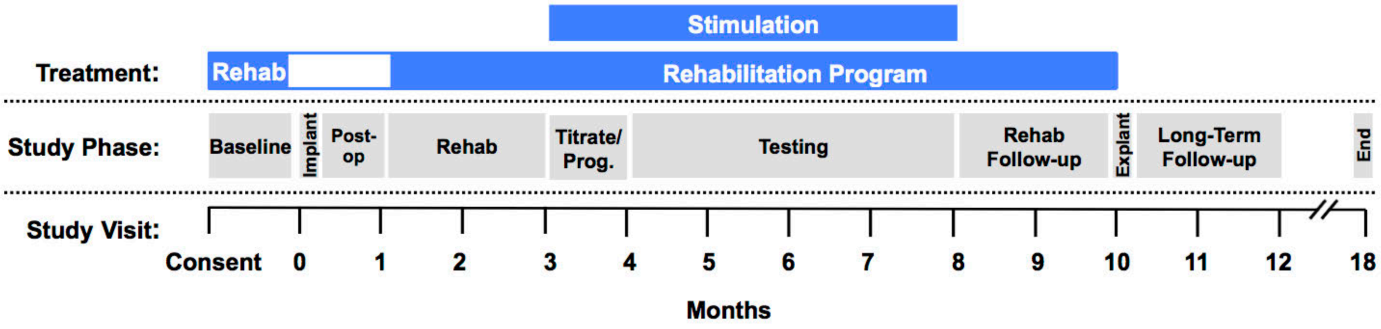

Figure 2: Study Timeline

[4] An independent consent monitor is added as per discussion in face-to-face meeting on July 20, 2015 with FDA.

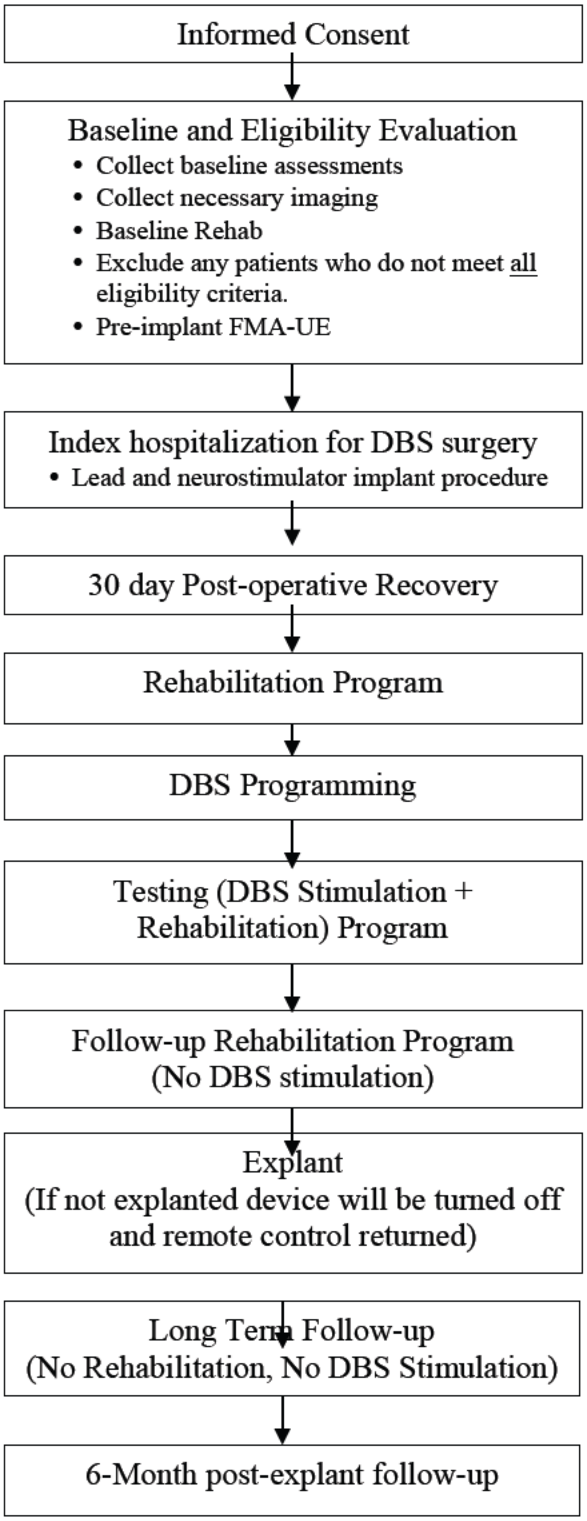

Figure 3: Study Flow Diagram[5]

---

[5] Updated pre FDA feedback, August 27, 2015- Section 2.2.6 of this IDE

7.1.1 Schedule of Study Visits

Table 2. Schedule of Study Visits[6]

Study Phase	Visit	Schedule
Baseline	As needed to complete screening for eligibility and baseline evaluations including pre-implant FMA-UE	~Up to two months
DBS Implantation Hospitalization	DBS Electrode Implant, and DBS neurostimulator Implant Procedure	1 day, with an expected 2-3 day hospitalization.
Postoperative Recovery (4 weeks)	N/A	Discharge = day 0
Rehab (8 weeks)	Month-1 Visit<br>Month-2 Visit	Starts day 28 (± 10 days)<br>day 56 (± 10 days)
Programming (4 weeks)	Month-3 Visit	Starts day 84 (± 10 days)<br>Up to 8 visits in a period of up to 4 weeks for programming the DBS system prior to the testing phase
Testing: stimulation of the dentate nucleus + Rehab 2x/week (16 weeks)	Month-4 Visit<br>Month-5 Visit<br>Month-6 Visit<br>Month-7 Visit<br>Month-8 Visit	Starts day 112 (± 10 days)<br>day 140 (± 10 days)<br>day 168 (± 10 days)<br>day 196 (± 10 days)<br>day 224 (± 10 days)
Rehab Follow-up (8 weeks)	Month-9 Visit<br>Month-10 Visit	Starts day 252 (± 10 days)<br>day 280 (± 10 days)
Explant	Explantation procedure	Overnight observation in the hospital. Explantation will occur within 2 months of prior visit (≤ day 336)
Long-term Follow-up	Month-11 Visit<br>Month-12 Visit	336 (± 10 days)<br>365 (± 10 days)
Study Completion	Month-18[7]	547 (± 20 days)

7.1.2 Schedule of Study Assessments

Table 3 provides an overview of the schedule of assessments required for the study. Study assessments, at other unscheduled visits, will be performed as clinically indicated and corresponding data shall be reported on appropriate study case report forms.

[6] Revised as per FDA feedback, August 27, 2015 – Section 2.2.6 of this IDE
[7] Updated to reflect protocol changes with the "baseline" rehab period added.

Table 3. Schedule of Study Assessments[8]

Study Phase:	Screening	Baseline	Implant	Recovery	Rehab		Prog.	Testing (Rehab + Stim)					Rehab Follow Up		Explant	Long-term follow-up
					Monthly Visits:											
					1	2	3	4	5	6	7	8	9	10		
Demographics	X															
Medical History	X															
Stroke History	X															
Physical Exam	X															
Neurological Exam	X															
NIH Stroke Scale (Item 8)	X															
Ashworth Scale	X															
Neuropsychological evaluation	X							X						X		
Laboratory Tests (Blood and Urine)	X		X													
Structural MRI	X															
PET		X				X				X				X		
Diffusion Tensor Imaging		X														
CT scan			X													
Transcranial Magnetic Stimulation	X		X				X	X	X	X	X	X				
9-Hole Peg Test		X			X	X	X	X	X	X	X	X	X	X		X
Arm Mobility Action Test (AMAT)		X			X	X	X	X	X	X	X	X	X	X		X
Beck Depression Inventory (BDI)		X			X	X	X	X	X	X	X	X	X	X		X
Bilateral Box and Block Test (BBT)		X			X	X	X	X	X	X	X	X	X	X		X
Bimanual Grip Strength		X			X	X	X	X	X	X	X	X	X	X		X
EuroQol (EQ-5D)		X			X	X	X	X	X	X	X	X	X	X		X
Fugl-Meyer Assessment, (FMA-UE)	X	X			X	X	X	X	X	X	X	X	X	X		X
Short Form Health Survey (SF-12)		X			X	X	X	X	X	X	X	X	X	X		X
Concomitant Medications		X	X	X	X	X	X	X	X	X	X	X	X	X		X
Adverse Events			X	X	X	X	X	X	X	X	X	X	X	X	X	X
DBS Device Check			X	X	X	X	X	X	X	X	X	X	X	X		
Rehabilitation Sessions		X			X	X	X	X	X	X	X	X	X	X		

[8] Revised as per FDA feedback, August 27, 2015 – Section 2.2.6 of this IDE

7.2 Baseline Evaluation

The following is a list of all required screening and baseline evaluation assessments:

- Medical History
- Stroke History
- Rehabilitation History
- Physical Examination (pertinent to their medical and neurological status)
- Neurological Examination (Include assessment of spasticity with the Ashworth Scale, and sensory assessment with the NIH Stroke Scale Item 8)
- Neuropsychological Examination
- Rating with all primary and secondary outcome measures
- Diffusion Tensor Imaging (DTI)
- FMA-UE
- PET

Assessments collected during screening will be used for baseline to avoid duplication. At the conclusion of the baseline testing, enrolled patients will be scheduled for DBS implant surgery.

During the baseline evaluation the subject will undergo a period of baseline rehab prior to implantation to avoid enrolling patients that can still benefit from physical therapy. The rehabilitation will include 4 weeks of rehab. As described in §7.4.1 "Description of the Rehabilitation Program" the therapy sessions will occur two times per week for 1-1.5hrs and focus on functional tasks using adaptive task practice (ATP) and repetitive task practice (RTP). Subjects shall be rescreened at least 2 weeks prior to surgery and the FMA-UE shall remain 25 or below (FMA-UE ≤ 25) after baseline rehab in order to be eligible for implant. DBS Implantation Procedure. [9]

The subject will be admitted to the Cleveland Clinic for the DBS surgery. The inpatient hospital stay is expected to require 1 to 3 days to complete the implantation of the DBS lead and pulse generator.

7.2.1 Unilateral DBS Electrode Implantation

The brain target site is the dentate nucleus of the cerebellum ipsilateral to the side of the body with upper extremity weakness (contralateral to the lesioned hemisphere). The brain target site will be localized for stereotactic implantation of the DBS lead using CT scans and MRI. A preoperative MRI will be fused with a preoperative stereotactic CT, to be acquired with the Leksell head frame depicts the position of the patient and the placement of the stereotactic head frame.

[9] Revised as per FDA feedback, August 27, 2015 – Section 2.2.6 of this IDE

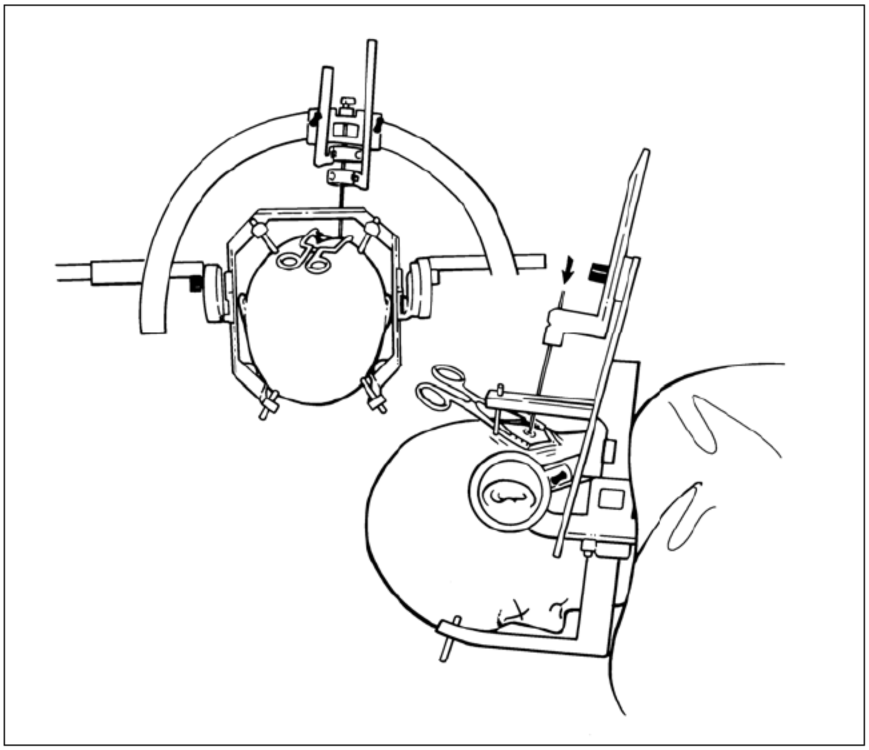

Figure 4. Illustration of Patient Position and the Placement of the Head Frame

The target will be selected on triplanar reformatted MR images by means of direct targeting. The intended target is the output of the dentate nucleus, as it projects to the superior cerebellar peduncle. In the proposed dentate nucleus region, implantation of the electrode will assume a trajectory from posterior to anterior, extending from an entry point in the occipital surface of the cerebellum to the rostral limit of the dentate nucleus and its output. The surgical goal will be to place the contacts flanking the area of interest which ranges from the region immediately rostral to the dentate nucleus (where white matter output of the nucleus projects to the superior cerebellar peduncle) to the caudal-lateral region of the dentate nucleus.

The trajectory will be planned using standard stereotactic techniques as to enter the occipital surface of the cerebellum, from a lateral to medial approach. As for any stereotactic procedure, the trajectory will avoid dural sinuses and the midline. Variability in the trajectory angles of approach to the dentate nucleus will depend on individual vascular anatomy. As with other stereotactic procedures, it is anticipated that there will be some variance in the location of superficial vessels of the cerebellum. Superficial vessels will be avoided and trajectories that will avoid the topography of larger sub-cortical vessels will be favored. The trajectories will also avoid the IV ventricle. The trajectories will therefore transverse the following structures:

1. Skin, muscle over the planned burr hole
2. Occipital bone, lateral to the midline and medial and inferior to the sigmoid and transverse sinuses, respectfully. It will be superior to the foramen magnum.

3. Dura mater, arachnoid and pia. These will be coagulated with bipolar coagulation prior to opening as we routinely do in stereotactic procedures at Cleveland Clinic.
4. Cerebellar cortex
5. Cerebellar white matter
6. Dentate nucleus

The patient will be under general endotracheal anesthesia. In this protocol, we anticipate patients to be positioned prone under general anesthesia. If the choice is for general anesthesia, we will ask the anesthesia team to maintain anesthesia with agents that minimize interference with intraoperative evoked responses during the intraoperative physiology time. Intraoperative imaging (fluoroscopy or O-Arm acquisition) will be used to provide intraoperative feedback, following the routine for frame-based DBS lead implantation at Cleveland Clinic.

An O-Arm will be utilized to acquire three-dimensional images intraoperatively. Once the electrode is inserted with stereotactic techniques, a volumetric stereo image with the O-Arm will be acquired and co-registered with the preoperative image using the planning station. Then the extent of error between the intended location of the lead and the actual location of the lead will be estimated. Large differences between the intended and actual location of the lead, such as 1.5 mm, may require the lead to be relocated.

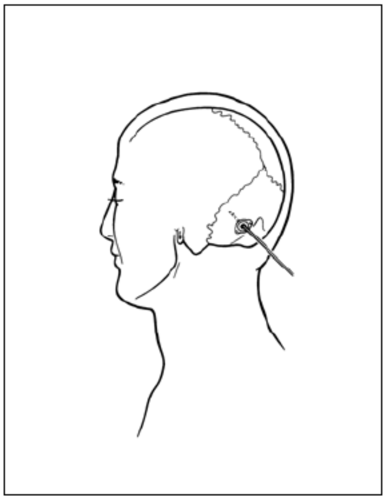

Figure 5. Illustration of Electrode Placement for the Dentate Nucleus Target

7.2.1.1 Intraoperative Macroelectrode Testing

Electroencephalography will be used during surgery for evoked potentials. Evoked potentials will be generated from the cerebellar electrode and recorded by EEG. In addition, the patient will also be monitored by median nerve somatosensory evoked potentials (SEPs). It is anticipated that the effective contact for dentate stimulation will generate evoked potentials around and outside the infarcted zone in the motor, pre-motor and parietal regions of the brain.

Once the DBS electrode is inserted, macroelectrode testing will be performed. Test stimulation with the DBS electrode will be attempted. Direct observation, EMG and EEG recordings will be used to evaluate motor and electrophysiological effects of macrostimulation with an external stimulator. In addition, MEPs and SSEPs may be used as test stimulation while evaluating the effects of deep brain stimulation of the dentate nucleus on the magnitude of these evoked potentials.

It is anticipated that in the first few subjects implanted in the study, that these electrophysiological measures would not be used to confirm or refine lead location. However, as experience is gained during the course of this investigation, it is possible that some electrophysiological measures will be used to confirm or refine lead location. As such, the electrophysiological measures to be collected in this study will be exploratory but may be used in some subjects to confirm or refine the location of the lead.

Undesirable effects (i.e. motor contractions at low thresholds) will be evaluated by increasing stimulation amplitude until a motor response is noted (i.e. motor threshold). All stimulation will be performed within charge density safety limits. Any effects observed during surgery such as motor contractions are expected to be fully reversible and not associated with any long-term deficits.

1. For each of the electrical contacts in the DBS lead, the amplitude will be set at 1mA, the pulse width at 90 microseconds, and the frequency at 30 Hz. The patient will be monitored for SSEPs, EEG and EMG. The amplitude will be increased in increments of 0.5mA or 1.0mA. The maximum amplitude will be determined by charge density limits, as discussed below. In addition, we will consider an amplitude "maximal" and no longer increase the amplitude if motor contractions are noted.

2. Once each of the electrical contacts has been tested as outlined above, additional parameters may be tested depending on intraoperative conditions (i.e. stability of vital signs, etc.). The testing, as outlined above, will be repeated at the same frequency (30 Hz) but a pulse width of 120 microseconds.

3. Finally, depending on surgical progress and anesthesiology, the testing will be repeated at a frequency of 100 Hz and pulse width of 90 microseconds.

After the final location for the lead is selected in the target area, the lead will be secured in the burr hole ring. Then the lead will be coiled in an upwards direction and the wound closed with a connector protecting the tip of the DBS lead. The wound will then be closed in standard surgical fashion.

7.2.1.2 Implantation of the Neurostimulator

The neurostimulator will be implanted in the standard fashion, similar to the procedure routinely performed at Cleveland Clinic for the management of movement disorders. Briefly, the site for implantation will be planned for the infraclavicular region. The plan is to implant the neurostimulator under the same anesthesia as for implantation of the DBS lead. However, prepping and draping will be re-done specifically for this step of the implantation procedure. A tunneling tool will be used to create a tunnel from the head to the chest and an extension cable will be passed. The pocket for placement of the neurostimulator will be created subcutaneously and the neurostimulator will be implanted and connected to the extension. The proximal end of the extension will be connected to the DBS lead and the wounds will then be approximated in the standard fashion.

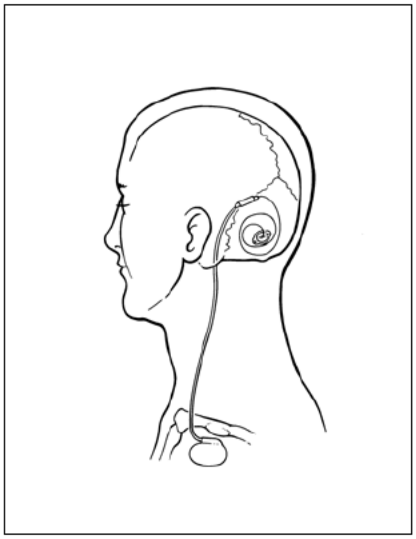

Figure 6. Final DBS System Placement

7.2.1.3 Positron Emission Tomography (PET):

PET will be used to add to the knowledge of the physiology of the dentatothalamocortical pathway and on the mechanisms of deep brain stimulation of the dentate nucleus.

7.2.2 Hospital Discharge

Prior to hospital discharge the patient will be assessed to determine whether any adverse events occurred during the hospitalization. All adverse events shall be documented. Medications will be reviewed and changes documented.

7.2.3 Postoperative Recovery Period

All subjects will be discharged from the hospital with the DBS system programmed OFF. During the month following hospital discharge the patient will recover from implant surgery and will not receive DBS stimulation or any rehabilitation therapy.

At the conclusion of the postoperative recovery period, the patients will return for a general clinical exam, and all patients will be asked about the occurrence of adverse events since hospital discharge. All adverse events shall be documented. All patients and/or caregiver will be trained on care and recharging their devices as per patient labeling.

7.3 Rehabilitation

An outpatient rehabilitation therapy program will be initiated at the start of the Rehabilitation phase and continue throughout the Programing, Testing phase (Stimulation + Rehab) and Rehab Follow-up phase.

During the Rehabilitation phase there will be two outcome assessment visits (one per month) in addition to visits for physical therapy. In each visit patients will be asked about the occurrence of adverse events since their last study visit. All adverse events shall be documented. Medications will be reviewed and changes will be recorded. Patients also will be evaluated with the primary and secondary outcome measure.

7.3.1 Description of the Rehabilitation Program

The rehabilitation method is focused on practice of functional tasks using adaptive task practice (ATP) and repetitive task practice (RTP). Tasks will be chosen based on each individual's perception of their relevance in concert with optimizing therapy goals and reducing upper extremity motor impairment. Therapy will be provided by physical therapists or occupational therapists.

Stroke survivors who are candidates for deep brain stimulation present with limitations in movement across several upper extremity joints to the point where they cannot perform meaningful functions with the impaired upper extremity either independently or bilaterally (using both arms) much beyond perhaps placing the impaired arm into the sleeve of a shirt or jacket. Following the procedure, training must exploit the acquired movement capabilities across several joints beginning with the shoulder and moving down the upper extremity to the digits. The activation of these joints will be done to increase the active range of motion (AROM) the patient can achieve at each joint; for example, shoulder flexion to bring the arm onto a table and elbow extension to reach towards an object. AROM will then be integrated into activities that are progressively segmented towards a functional goal. Such segmentation is referred to as adaptive task practice (ATP) also called "shaping". The patient will be encouraged to increase their AROM in progressive stages with much verbal encouragement and praise based upon whether they can move faster or more times per unit of time. As reaching the target (for example, to pick up a can or cup) is achieved, the goal becomes better quality of function through repetitive task practice (RTP), repeating the task to completion over many efforts to reinforce the success that was first achieved through the shaping procedure.

The determination around which tasks to practice cannot be prescriptive or patients will not comply with task practice whether in the clinic or in the home. Therefore we determine together those tasks which we call "challenges" and which are determined by the patient based upon how the patient values their importance. This negotiated approach fosters compliance and reinforces the desire to engage in more tasks. With each new task, we progress from the adaptive task practice mode to the repetitive task practice mode. The amount of time in this progression is dictated by patient compliance and reacquisition of more motion. Most often patients select tasks related to grooming, eating, and bathing. Men often choose tasks that might be described as more physical and woman tasks that they associate with optimizing their functional behaviours prior to sustaining a stroke. The rate of progression is dependent on the severity of stroke and the motivation of the patient, so total numbers of tasks and their sequence can vary considerably. The ultimate goal is to make the patient motivated to undertake self-directed and sustained task reacquisition.

The therapy sessions will follow the same frequency and duration throughout the study. The frequency and duration of therapy sessions will be two times per week for 1-1.5 hours of treatment time over a 2-hour contact interval. In addition to the formal outpatient therapy sessions, participants will sign a behavioral contract that obligates them and their caregivers to continue with the upper extremity rehabilitation program at home.

During the Baseline Rehabilitation period of 4 weeks the patient will participate in 8 therapy sessions. During the Rehabilitation Period patients will participate in 16 therapy sessions. For sessions 1 through 8, therapy will involve a 1:1 ratio of repetitive task practice (RTP) activities to adaptive task practice (ATP) activities. Sessions 9 through 16 will include a 1:2 ratio of RTP to ATP activities. This entire sequence of 16 sessions will be repeated at the start of Testing Period so that the same ratiometric relationships in training is used when their stimulation is turned ON. After they repeat the task ratios they will advance in their training based on the progress of the patient and recommendations of the therapist.

Throughout the study, the frequency and duration of outpatient rehabilitation sessions and the proportion of time spent on RTP and ATP exercises will be documented. Patients and caregivers also will be expected to record the type, frequency, and duration of home practice activities.

Therapists from the Cleveland Clinic will be providing the rehabilitation therapy and performing the outcome assessments for the study. To ensure consistency and compliance with the study protocol, therapists will receive training on the rehabilitation methods and administration of the outcome measures employed for the study.

7.4 Device Programming

The study team at the Cleveland Clinic Center for Neurological Restoration (CNR) will be responsible for programming and device follow-up. Programming of the device will utilize standard DBS techniques for assessing effects and side effects

during a cathode survey of the DBS lead. In addition, programming will be guided by measuring the effects of acute stimulation on the magnitude of motor evoked potentials (MEPs) evoked with transcranial magnetic stimulation (TMS).

The primary purpose of this programming phase is to select the optimal chronic stimulation parameters to use during the testing (Stim + Rehab) phase of the study. The principal investigator will be responsible for determining the final stimulation settings. Given the nature of this feasibility study, the selection of initial stimulation parameters for testing will be partially based on available animal data on deep cerebellar stimulation. It is expected, from the results of these animal studies (see preclinical data section), that stimulation frequency will be set in the range of 20-25 Hz. Final selection of chronic frequency, active contacts, pulse widths and amplitudes will be based on the titration testing using a systematic cathode survey.

A cathodal survey will be performed by activating, in a systematic fashion, each contact as the cathode. All stimulation testing will be conducted below the safe change density limit of 30 $\mu C/cm^2$/phase. as described in the Boston Scientific Implant Manual. It has been demonstrated that TMS can induce currents in a DBS lead resulting in stimulation at the electrodes [147-150]. During the delivery of TMS, induced current will be included in the calculation of charge density to ensure that the overall charge density never exceeds 30 $\mu C/cm^2$/phase. The induced current will be calculated utilizing the measured contact impedance and an estimate of voltage conservatively estimates twice that reported by Rossi (i.e. 2 V). The charger density will be calculated, based on the programmed pulse width, overall current (induced current plus programmed current), and contact surface area.

Each cathode will be tested with stepwise increments in amplitude from 0 to a maximum of 10 V. Pulse widths will be initially set at 90 microseconds and will not exceed 210 microseconds. Amplitudes will be increased until a side effect is noted or reported by the patient or the charge density limit is reached. Patient reports of subjective responses such as paresthesia, changes in the visual field or perception, or other unpleasant psychological or perceptual changes also will be considered in defining a maximal amplitude threshold for each contact.

In addition to direct observation, transcranial magnetic stimulation will be used during DBS programming to determine the motor threshold (MT) and measure the effects of dentate deep brain stimulation on motor evoked potential amplitude.

Any concern for long-term side effects will be avoided by reprogramming stimulation as soon as a side effect is noted during the programing phase.

7.4.1 Transcranial Magnetic Stimulation (TMS)

Transcranial magnetic stimulation (TMS) will be used with the goal of identifying changes (increments) in cortical excitability that may result from cerebellar stimulation. The objective is to select an optimal stimulation setting that will not cause adverse effects while at the same time resulting in a measurable increment of cortical excitability. TMS will be done at baseline (prior to stimulation testing), and intermittently throughout the systematic cathode survey. Enhancement of cortical excitability will be defined as a reduction in the motor threshold (aMT) elicited by

TMS and gain in corticospinal output will be defined as an increase in the amplitude of the motor evoked potentials elicited by TMS. To measure changes in corticospinal output, we will adopt a new standard- recruitment or input-output curve. The curve is plotted by investigating the MEP responses of a given muscle at incremental intensities. The curve is believed to reflect the output and the gain of the descending corticospinal tracts. In general, it has been established that all skeletal muscles demonstrate a sigmoidal curve—as stimulus intensity increases, a steep increase in recruitment occurs that eventually plateaus due to maximal activation of all available CST. The slope of this curve is generally stunted in patients with stroke, but change in slope across the incremental intensities is a strong indicator of increase in corticospinal output and its gain. In addition, the ipsilesional motor cortex will be mapped in such fashion as to establish the motor representation of the affected forelimb in the ipsilesional cortex during the study (i.e. before and after training and training + stimulation) In patients who are below FMA < 20, standard recruitment curves or input-output curves can be challenging to acquire. This is because these curves rely on eliciting MEPs at several different TMS intensities, ranging from 90-150% aMT. Since in patients with severe deficit (FMA < 20) aMTs are typically high, it would preclude testing of curves at high intensities. We will study additional metrics that will serve as alternatives in such a case. We will record MEP amplitude in test muscle at 120% aMT (EDC typically, but triceps or middle deltoid in patients who have no MEP in EDC). While patients voluntarily contract their target muscle (EDC, triceps, middle deltoid) to 50-100%, we will apply TMS to ipsilesional and contralesional M1 separately. TMS can evoke transient suppression of on-going EMG in the paretic muscles that reflects change in intra-cortical excitability in ipsilesional M1 and inter-hemispheric influence from contralesional cortices. In our work [151] and of others, these metrics are acquired feasibly in patients with severe impairment as well.

7.4.2 *Safety of Transcranial Magnetic Stimulation and DBS*

To address any safety concerns about the proposed use of TMS to evaluate cortical excitability during interoperative stimulation testing with the DBS electrode implanted, and during follow-up with the DBS system implanted, a literature review was performed.

A significant body of literature clearly documents the safety of the proposed use of TMS to evaluate cortical excitability during testing of deep brain stimulation parameters with only a slight modification of standard TMS protocol. At least two independent groups have developed phantom skull models to test whether TMS induced currents in the scalp-coiled leads could result in damage to the patient's brain or to the implanted stimulators. Both studies concluded that stimulation over the skull at 1 cm from the coiled leads induced very small transient currents in the electrode wires that remain well below the amplitudes used for DBS and are therefore considered to be safe. Kumar (1999) [169] further showed that TMS over the scalp leads does not deliver damaging currents to the stimulators. The only potential concern raised by these studies was that inadvertent TMS directly over the neurostimulator could cause the neurostimulator to malfunction. Since the original phantom study by Kumar greater than 50 patients have undergone TMS evaluations

of cortical excitability during deep brain stimulation. These have included TMS during DBS in either bilateral or ipsilateral basal ganglia and/or thalamus in patients with Parkinson's Disease, severe dystonia,[170] essential tremor, and intractable epilepsy. In all these DBS studies, no adverse effects of TMS were reported. The only modifications to standard TMS procedures was that in some of these cases, patients wore protective jackets that served as a cue for investigators to protect against the potential for inadvertent TMS application directly over the neurostimulator.

For TMS performed during post-neurostimulator implantation DBS programming, care will be taken to avoid stimulation near the neurostimulator. A Protective foam jacket with ≥ 7 cm thickness over the site of the neurostimulator will be worn by patients to avoid stimulation near the neurostimulator. Further, the expected induced currents in the DBS electrodes will be even smaller than those reported in the phantom models because the DBS lead will be coiled under the muscle layer of the contralateral suboccipital region, relatively far from the placement of the TMS coil above the motor cortex. If the neurostimulator is placed on the non-paretic side of the body, which would be closer to the test hemisphere (ipsilesional) for TMS, then caution will be practiced in additional ways. Patients who require greater than 80% of TMS intensity will not be included. We do not believe that this extra precaution would be exclusionary because we will measure and record all TMS variables in the active state of the paretic muscle. To still ensure that neurostimulator does not malfunction, we will take a printout of all DBS parameters at beginning and will keep checking during TMS testing and at the end to ensure that values do not vary with TMS. TMS has been delivered with DBS in cases where exit site for leads is close to the motor cortex, i.e. in proximity to scalp site where TMS is applied. But, in our proposed project, the leads will be significantly posterior and thus are expected to have little interference from TMS. Therefore, the specific DBS protocol stimulating the DTC, and the proposed application, and safeguards for TMS testing will allow a safe synergistic application of both modalities in this feasibility study.

The literature cited above specifically address the use and safety of TMS to assess cortical excitability in patients with implanted DBS devices, and supports the conclusion that TMS over the scalp will not cause damaging stimuli to the patient's brain, to the DBS electrode or pulse generator following the testing procedures described in this section.

7.4.3 Testing phase (stimulation + rehabilitation)

After completing the Rehabilitation Phase and DBS programing the DBS stimulation will be initiated. The Subject will continue their rehabilitation program. Settings for chronic deep brain stimulation will be selected based on the prior DBS programming phase. However, the investigators will repeat the procedures for DBS programming which will include TMS monthly during the Testing phase (rehabilitation + DBS). This procedure will be done in order to account and compensate for possible accommodation of the neural network to stimulation during the testing phase.

During the testing phase visits, all patients will be asked about the occurrence of adverse events since their last study visit. All adverse events shall be documented. Medications will be reviewed and changes will be recorded.

Outcome measures will be assessed once/month. Programming of the neurostimulator will be performed in the same manner as the initial device programming and subjects will have their stimulation parameters adjusted. Subjects will be programmed to stimulation settings found to be optimal for enhancement of cortical excitability while avoiding side effects. Again, TMS will be used to index cortical excitability.

The goal is to re-measure cortical excitability, again indexed by TMS, and learn if there are changes with chronic stimulation. If changes are noted, the DBS will be re-programmed in the same fashion as during the programming phase. If changes are not noted, DBS will be checked (i.e. evaluation of impedances, ascertain that stimulation is ON) but not reprogrammed

A cathodal survey will be performed by activating, in a systematic fashion, each contact as the cathode. All stimulation testing will be conducted below the safe charge density limit of 30 microcoulombs/cm2/phase as described in the Boston Scientific Implant Manual. Each cathode will be tested with stepwise increments in amplitude from 0 to a maximum of 10 mA. Pulse widths will be initially set at 90 microseconds and will not exceed 210 microseconds. Amplitudes will be increased in stepwise fashion in increments no greater than 1 mA at a time until a side effect is noted or reported by the patient or the charge density limit is reached. Patient reports of subjective responses such as paresthesia, changes in the visual field or perception, or other unpleasant psychological or perceptual changes also will be considered in defining a maximal amplitude threshold for each contact.

7.5 Rehab Follow-up

During this phase the subject will have their stimulation gradually reduced and subjects will be weaned of continuous DBS. Weaning will occur during the course of one month at weekly visits. In each visit, the power will be reduced by approximately one quarter of the power used at the end of the testing phase until the power is reduced to zero.

During each visit patients will be asked about the occurrence of adverse events since their last study visit. All adverse events must be documented/reported. Medications will be reviewed and changes will be recorded. Patients will complete primary and secondary outcome measures. During the last visit, a general clinical exam will be performed.

7.6 Long-Term Follow-up:

Long-term follow-up continues until the patient reaches the study conclusion at 12 months post implant. Patients will be monitored for adverse events and will be assessed for any continuing changes in any of the primary or secondary outcome

measures. No formal rehabilitation therapy is required by the study protocol during long-term follow-up.

7.7 Explant

After Rehab Follow-up subjects will have the DBS system explanted at the end of the Long-Term Follow-up. All subjects will return 6 months after explant for a post-explant follow-up to evaluate any adverse events due to the device or therapy.

In the event that treatment (Stim + Rehab) demonstrates benefit but decreases significantly once stimulation is discontinued, a Compassionate Use IDE supplement will be submitted to FDA requesting that subjects may keep their DBS system ‘on’ and continue their DBS therapy while Enspire pursues the next subsequent study. A significant decrease in benefit will be defined as a loss of more than 50% of the motor gains attributed to deep cerebellar stimulation (i.e. gains achieved only after stimulation was activated). In order to continue receiving therapy the subjects will re-consent for this longer-term study.

In the event that a subject does not have the device removed, the system will be turned OFF and the Remote Control and Charger will be returned. Subjects that do not have the device removed will be followed up after 6 months and adverse events will be monitored. Enspire will seek FDA approval for a long term safety surveillance study (e.g. up to 5 years) for these subjects (i.e. subjects that has the DBS system implanted but turned off). A separate informed consent will be done with this surveillance study.

7.8 Exit of Participation

All subjects who exit from the study shall have the reason for their exit documented. Reasons include: completion of study, lost-to-follow-up, subject withdrawal, discontinue due to adverse event, physician-directed subject withdrawal, and death.

7.8.1 Loss to Follow-Up

Every attempt shall be made to have all subjects complete the study visit schedule. A subject will not be considered lost-to-follow-up unless efforts to obtain compliance are unsuccessful. At a minimum, the effort to obtain follow-up information must include three documented attempts to make contact via telephone or email, and if contact via phone or email is not successful, then a certified letter from the Principal Investigator shall be sent to the subject’s last known place of residence.

7.8.2 Subject Withdrawal From Study

All study subjects have the right to withdraw their consent and withdraw participation at any time during the study. Whenever possible, the site staff should get written documentation from the subject that wishes to withdraw consent for future study participation. If the site’s staff is unable to obtain written

documentation, all known information regarding the subject's withdrawal shall be recorded. If the patient has received the implant at the time of withdrawal from the study the device will be programmed to OFF and zero volts. Explant of the device will be recommended unless there is a medical reason that contraindicates procedure for explanting.

7.9 Unscheduled Visits

All unscheduled visits shall be documented and any adverse events reported during an unscheduled visit shall be recorded. Performance of study assessments at unscheduled visits should be done as clinically indicated, and results documented on the appropriate study case report forms.

7.10 Protocol Deviations

The Principal Investigator, co-investigators and study staff must avoid all protocol deviations. The investigators should not implement any deviation from, or changes to, the IDE protocol without prior review and documented approval from the IRB of the deviation, except where necessary in an emergency to eliminate an immediate hazard to subjects, or when the change(s) involves only logistical or administrative aspects of the trial (e.g., change of monitor(s), change of telephone number(s)). If such a deviation is due to an emergency, the principal investigator will report the deviation to the Sponsor immediately, but no later than 24 hours after its occurrence. The sponsor of the study will seek FDA approval through an IDE supplement for any changes to the study protocol that might affect the rights, safety or welfare of the subjects or scientific soundness of the study.

8.0SAFETY REPORTING

Adverse events will be assessed and documented by the investigator at all study visits The investigational site will provide source documentation to the Sponsor to facilitate review and adjudication of AEs.

The principal investigator or the co-investigator is to report all deaths, life threatening events, and any unanticipated, serious adverse events to the Sponsor within 24 hours for investigation and required reporting to FDA, device manufacturers and IRB within the required timeframe. This includes Unanticipated Adverse Device Effects (UADE). The principal investigator will support the investigation and follow up of any serious adverse events until resolved.

8.1 Definitions and Classification

The following definitions are from Good Clinical Practice (ISO 14155:2011) and FDA Code of Federal Regulations for Medical Devices (21 CFR 812.3(s))

8.1.1 Adverse Event (AE)

An AE is any untoward medical occurrence, unintended disease or injury or any untoward clinical signs (including an abnormal laboratory finding) in subjects, users or other persons whether or not related to the investigational medical device.

AE includes all hospitalizations and events related to the investigational device or the comparator. This includes events related to the procedures involved (any procedure in the clinical investigation plan). For users or other persons this is restricted to events related to the investigational medical device.

AE does not include conditions pre-existing to the subject's enrollment. Pre-existing conditions will not be reported as AEs unless the condition has an increased occurrence or intensity.

8.1.2 Serious Adverse Event (SAE)

An SAE is any AE that:[10]

(a) led to a death,
(b) led to a serious deterioration in health that either:
- (1) resulted in a life-threatening illness or injury,
- (2) resulted in a permanent impairment of a body structure or a body function,
- (3) required in-patient hospitalization or prolongation of existing hospitalization,
- (4) resulted in medical or surgical intervention to prevent life threatening illness or injury or permanent impairment to a body structure or a body function,
- (5) resulted in a substantial disruption in ability to conduct normal life functions.

(c) led to fetal distress, fetal death or a congenital abnormality or birth defect.
(d) when the event does not fit the above outcomes, but the event may jeopardize the patient and may require medical or surgical intervention (treatment) to prevent one of the other outcomes.

SAE does not include in-patient hospitalization for a planned study procedure. Planned study-related in-patient hospitalization is not an SAE.

SAE includes device deficiencies that might have led to a serious adverse event if (a) suitable action had not been taken or (b) intervention had not been made or (c) if circumstances had been less fortunate. These are handled under the SAE reporting system. A planned hospitalization for pre-existing condition, or a procedure required by the Clinical Investigation Plan, without a serious deterioration in health or to prevent life threatening illness or injury or permanent impairment to a body structure or a body function, is not considered to be a serious adverse event.

[10] Updated as per FDA feedback, August 27, 2015, Question 14

8.1.3 *Device deficiency*

A Device Deficiency is any inadequacy of a medical device related to its identity, quality, durability, reliability, safety or performance, such as malfunction, misuse or use error and inadequate labelling. A Device Deficiency occurs in any case where the device does not perform in its intended function and when used in accordance with the device labelling.

8.1.4 *Adverse Device Effect (ADE)*

An ADE is an AE related to the use of an investigational medical device.

ADE includes any adverse event resulting from insufficiencies or inadequacies in the instructions for use, the deployment, the implantation, the installation, the operation, or any malfunction of the investigational medical device. This includes any event that is a result of a use error or intentional misuse.

8.1.5 *Serious Adverse Device Effect (SADE)*

An SADE is an ADE that has resulted in any of the consequences characteristic of a serious adverse event.

8.1.6 *Unanticipated (Serious) Adverse Device Effect (UADE)*

A UADE is a SADE which by its nature, incidence, severity or outcome has not been identified in the protocol or in the investigational device manuals.

Per 812.3(s) this includes any SAE caused by, or associated with, a device, if that SAE was not previously identified in nature, severity, or degree of incidence in the investigational plan or application (including a supplementary plan or application), or any other unanticipated serious problem associated with a device that relates to the rights, safety, or welfare of subjects.

8.1.7 *Relatedness to DBS Device*

Implantation procedure-, device-, and stimulation-related adverse events are defined as any adverse events that are considered by the investigator and evaluated by the Sponsor to be related to the implant procedure, DBS device, or stimulation, with consideration of the strength of the temporal relationship to the implant procedure, onset or cessation of stimulation and the presence or performance of the DBS system, and the presence or absence of an alternative etiology such as the underlying disease, co-morbidities, and concomitant drugs/treatments.

The probability that a particular AE is related to the procedure, device or stimulation shall be coded on the AE case report form as *unrelated*, *possibly related* and *related*. Probability ratings are based on the temporal relationship to intervention, the likelihood that the symptom could have been produced by the participant's clinical state, the environment or other interventions, whether the participant's symptom course follows a known pattern of response to the intervention, and the experience and judgment of the investigators.

8.2 Device Deficiencies

All Device Deficiency shall be documented and reported in the IDE Annual report, it will include those that require explanation of the device. If a Device Deficiency results in an adverse event for the subject, including those that require explanation of the device, will be reported as an adverse event and will be classified appropriately. Device Deficiency that do not result in an adverse event for the subject do not need to be recorded as an AE, as they are not considered an AE.

If a Device Deficiency occurs with the Vercise system the investigators will follow the procedures outlined in the device labeling regarding return of the product to Boston Scientific, Inc. This applies to deficiencies that are identified prior to implantation as well as those that that require explanation of the device.

8.3 Deaths

Each subject death shall be reported. A copy of the death certificate and a copy of the autopsy report, if available, should be obtained. Any other source documents relied upon to make a determination of death classification and cause of death will also be filed with the subject's study documents. Data Management

In addition to case report forms, copies of the following source documents verifying any study assessments that are performed are required to be maintained in the subjects' study records and be available for the study monitor to review as appropriate. These may include:

- Laboratory test results
- Imaging results
- Electrophysiological testing results

9.0DATA MANAGEMENT

9.1 Completion of Case Report Forms

To ensure data quality and completeness, all required study data shall be recorded on case report forms (CRFs). The Principal Investigator or appointed designee is responsible for capturing the information or transferring the information from source documents onto the CRFs. The PI, Co-Investigator, or appointed designee must review case report forms, and validate/sign the completed CRFs.

9.2 Data Review

Study monitors will review the information documented in the CRFs and verify the information recorded is consistent with medical records or other source documents. Errors or incomplete entries will be rectified by study staff and principal investigator for correction and preventive measures.

Additional information and source documentation will be reviewed during monitoring visits or be submitted to the Sponsor for evaluation, for example, for

adjudication of adverse events by the DMC or when device deficiencies are reported.

All clinical centers will be monitored periodically by the Sponsor for protocol adherence, accuracy of CRFs, and compliance to applicable regulations.

9.3 Data Analysis Plan

All safety and outcome assessments will be summarized with appropriate descriptive statistics. Non-continuous variables will be summarized by percentages and frequency distributions. Data will be tabulated for all enrolled patients. Exploratory statistical analyses will be performed to identify potential relationships between the treatment and the outcome variables.

Adverse event data will be reported and tabulated according to the severity and likely relationship with the device, procedure or stimulation.

Events will be considered to be permanent if the adverse event does not resolve despite medical management, changes in device programming, or stopping treatment.

10.0 STUDY ADMINISTRATION

10.1 Data Monitoring Committee

An independent Data Monitoring Committee (DMC) will be established to provide additional, independent oversight to enhance safety of study participants and ensure subjects' rights and welfare. The DMC will be responsible for safeguarding the interests of trial participants by assessing the safety and efficacy of the interventions during the trial, and for monitoring the overall conduct of the clinical trial.

The DMC will consist of three experts with relevant clinical specialties. This will include at least one physician that practices neurology or physical medicine and rehabilitation and specialize in post stroke care and at least one neurosurgeon experienced in DBS therapy. They should also be familiar with FDA regulated clinical research with investigational implantable devices and/or investigational drugs. Experience with serving on other DMC, DSMB and IRB are desirable. These three experts are the core members with voting responsibility.

Other experts such as physical therapist, rehabilitation professionals, and medical ethicists may be invited to participate on an as needed basis as additional advisors to evaluate complex and difficult issues.

10.1.1 DMC Responsibilities

The DMC is governed by its Charter[11]. The DMC will be responsible for safeguarding the interests of trial participants by assessing the safety of the

[11] See Section 8.4 of this IDE.

interventions during the trial, and for monitoring the overall conduct of the clinical trial. The DMC is responsible for defining its deliberative processes, including event triggers that would call for an unscheduled review, stopping guidelines, and voting procedures prior to initiating any data review.

10.1.2 Monitoring for Safety

The DMC reviews safety data from the study and will make recommendations, if appropriate.

One member of the DMC will be assigned as an independent reviewer of all serious adverse events. The independent reviewer will be notified within 2 business days of becoming aware of an adverse event to assess whether the event requires review by the full board. If full board review is required, an unscheduled DMC board meeting will be held via teleconference within 5 business days to determine if enrolment should be halted until the event has been satisfactorily resolved. Specific rules will be determined by the DMC during the first organizational meeting.

10.1.3 DMC Meetings

Scheduled Meetings

The frequency of scheduled meetings depends on subject enrollment and adverse event rates. The DMC will convene according to the following schedule: first subject implanted; and when the first subject completes the testing phase. Upon FDA approval to allow DBS implant for the 4 enrolled subjects, the DMC will review data after the 4 additional subjects have been implanted and all the enrolled 5 patients have completed the Testing Phase (DBS + Rehab).

If FDA approves further expansion of the trial to enroll a total of 12 subjects [as per original study plan], the DMC will review data when the additional 7 subjects have completed the Testing Phase, and then when the last subject completes the Testing Phase.

The DMC will review data related to: implant and other surgical safety; stimulation (therapy) safety for at least one-month data of " DBS + rehab " phase for each subject, and any safety information collected, whether it is related to the device/therapy or not.

Meeting To Evaluate Safety Data for First patient: For the first patient, the DMC will review safety data within 10 days of availability of the safety dataset once the first the subject has completed one (1) month of the Testing Phase. The objective of this meeting is to evaluate safety data for the first subject and make recommendations to the Sponsor. Sponsor then requests expansion of the IDE to enroll additional patients. The DMC will also make recommendations regarding whether it is appropriate to change the inclusion/exclusion criteria to enroll subjects with FMA-UE scores of > 20, if initial safety data warrant enrolling less severely affected stroke patients.

Unscheduled Meeting to Evaluate Important and Urgent Safety Data: If significant and urgent safety data emerges, e.g. unanticipated serious device or treatment related adverse events, the DMC will convene a meeting to evaluate the safety within 5 days.

10.1.4 Making Recommendations

The DMC may recommend one of the following actions to the study Sponsor; note this is not an exhaustive list:

- May expand the study beyond 5 subjects and enroll additional subjects with the current inclusion/exclusion criteria.
- Subject's inclusion criteria can be expanded to subjects with less severe symptoms, i.e. with FMA-UE scores higher than 20.
- Modify the study protocol. Modifications may include, but are not limited to, changes in inclusion/exclusion criteria, frequency of visits or safety monitoring, alterations in study procedures, adjustments in sample size, changes in duration of observation and follow up.
- Discontinue the study (with provisions for orderly discontinuation in accord with good medical practice) due to safety concerns.

10.1.5 DMC Decision Guidelines

General Considerations: The trial is a feasibility trial where the primary focus is on patient safety. Therefore, DMC recommendations will be made based on clinical judgment rather than statistical results or rules.

Stopping for significant safety risk:[12] Any recommendation to terminate the trial due to significant safety risk will be made on the basis of sound medical judgment. For the first cohort of 5 subjects, upon review and deliberation by the DMC, the DMC may immediately halt enrollment/implant of additional patient(s) for the clinical trial if there is no satisfactory explanation or resolution of the UADE.

If two or more UADE with un-explained etiology and lack of satisfactory resolution of the event, the DMC may recommend terminating the clinical trial as designed. The DMC may also recommend making changes to the protocol to improve the safety of the clinical trial.

For this early phase safety study with no control arm, and especially for the first cohort of subjects, no or little efficacy would be expected and therefore a benefit-risk analysis may not be appropriate. The DMC should take into consideration the potential scientific gain from continuing the study evaluated in the context of ethical considerations for ensuring subjects' rights and welfare.

[12] Stopping rule as per FDA feedback, August 27, 2015, Question 14

10.2 Study Registration.

The study will be registered with www.clinicaltrials.gov as recommended by the International Committee of Medical Journal Editors.[152]

11.0 SCIENTIFIC SOUNDNESS

It is expected that the results of this early feasibility study will provide adequate information on which to base decisions regarding the need for, and the design and conduct of future trials of deep brain stimulation of the cerebellar dentate nucleus for treatment of post-stroke motor impairment.

The rationale for the treatment suggests that the potential benefits outweigh the risks of the treatment for the population under study. Bias in patient selection is minimized by clearly defined enrollment criteria, and procedures to track and report patient recruitment and screening activities. Outcome assessment bias and measurement error is minimized by selecting qualified professionals who are experienced in the diagnosis, assessment and treatment of individuals with post-stroke upper extremity hemiparesis to perform the outcome evaluations, use of standard assessment tools that are known to be valid and reliable.

The sample size selected for this early feasibility study, if approved by FDA to expand to a total of 12 subjects will provide safety information and estimates of the study outcome assessments.

As a feasibility study, the results will be carefully interpreted in light of the postulated mechanism of action of stimulation to improve motor function, the expected risks and benefits of cerebellar dentate nucleus stimulation, and the risks and benefits of alternative treatments.

12.0 RISK/BENEFIT ANALYSIS

12.1 Potential Benefits of Electrical Stimulation of the Dentate Nucleus

There are no guaranteed benefits from participation in this study; however, it is possible that electrical stimulation of the dentate nucleus may reduce upper extremity motor impairment and improves motor functioning. Participants may achieve a greater degree of functional use of their arm, and as a result, may increase their ability to independently perform activities of daily living and enhance their social and community participation.

12.2 Potential Risks of Electrical Stimulation of the Dentate Nucleus

Risks associated with the Vercise DBS system and implant procedure are summarized in the device labeling.[153] In addition, adverse events and complications associated with deep brain stimulation have been reported in the peer-reviewed literature.

The risks associated with participation in this study of deep brain electrical stimulation of the dentate nucleus for the treatment of chronic, post-stroke hemiparesis are classified into three categories: risks associated with the device implant procedure, risks associated with the DBS system and components of the system, and risks associated with the stimulation therapy.

12.2.1 Risks Associated with Vercise DBS System Implantation Procedure

Risks associated with the procedure for implantation of a deep brain stimulation system include all possible risks related to any neurosurgical intervention. In essence, any organ system is at risk perioperatively, particularly in a person with presumed vasculopathy. These risks include (but are not limited to):

- Blood clot forming, for example in the veins of the legs, and thromboembolism. (i.e. pulmonary embolism).
- Hospital acquired conditions such as pneumonia.
- Blood clot or air in the blood stream, which can block blood flow to parts of the lungs or other tissue
- Perioperative medical complications including cardiovascular complications (i.e. myocardial infarction, arrhythmias), kidney complications (including renal insufficiency), liver problems (including failure), pulmonary problems (including respiratory failure), abdominal problems (including acute abdominal problems, peritonitis), other medical problems (including potentially serious or fatal).
- Cerebral spinal fluid (CSF) leaking outside the skull
- Confusion or problems with attention, thinking, or memory, speech or any other cognitive or behavioral problem, including psychosis and hallucination.
- Death
- Infection, including infection of the central nervous system
- erosion / exposure or the hardware in which case the device may need to be partially or completely removed
- Injury to areas next to the implant, such as blood vessels, nerves, the chest wall, the lungs, pleural space and the brain
- Neurosurgery/anesthesia risks, including unsuccessful implant
- Pain, headache, loss of sensation or discomfort
- Seizures
- Stroke resulting in temporary or permanent problems, such as paralysis, inability to swallow, cranial nerve deficits or inability or difficulty speaking
- Intracranial hemorrhages, resulting in transient or permanent neurological problems including paralysis, inability to swallow, cranial nerve deficits or inability or difficulty speaking.
- Brain edema resulting in transient or permanent neurological problems including paralysis, inability to swallow, cranial nerve deficits or inability or difficulty speaking.
- Swelling (seroma), bruising or hematomas
- Movement disturbances,
- Double vision, blindness or other vision problems

Table 4 summarizes the most common risks known to be associated with implantation of deep brain stimulation systems for approved indications reported in the literature.

Table 4. Risks of Deep Brain Stimulation Implant Procedure

Risk	Expected Rate	Source
Hemorrhage	2.25. to 4%	Binder, 2003<br>Terao, 2003<br>Binder, 2005<br>Deogaonkar, 2007
Infection or erosion	3.8% to 8.1 %	Umemura, 2003<br>Sillay, 2008<br>Hamani, 2006<br>Temel, 2004<br>Deogaonkar, 2007
Hardware complications	5 to 26%	Lyons, 2004[154]<br>Hamani, 2006<br>Voges, 2006

Risks of a DBS implant procedure of special concern in a deep cerebellar target are: bleeding, infarct, stroke, cerebrospinal fluid leak, and air embolism. These risks have been minimized by the proposed implant procedures. The patient will be implanted in the prone position which is known to reduce the risk of air embolism compared to the sitting position. Careful direct brain targeting will be accomplished with state of the art stereotactic systems

Furthermore, only one functional neurosurgeon (study PI) will be performing the implant procedures. The Cleveland Clinic Foundation has an experienced DBS team, and has performed hundreds of deep brain stimulation procedures in a variety of brain target areas for a variety of indications. CCF has rigorous operative procedures, and perioperative procedures care to ensure complications of surgery and DBS stimulation are minimized.

12.2.2 Risks Associated with Vercise DBS System

Risks associated with the deep brain stimulation system include:

- Allergic or immune system response to implanted materials
- Failure or malfunction of any part of the device, including but not limited to: Battery leakage, battery failure, lead or extension breakage, hardware malfunctions, problems recharging the device, loose connections, electrical shorts or open circuits, and lead insulation breaches, whether or not these problems require device removal and/or replacement
- Implant site complications such as pain, poor healing, redness, warmth, swelling or wound reopening
- Implanted device components (stimulator, lead, or extension) may move from original implanted location or wear through the skin, which may lead to the need for additional surgery

- Infection
- Interference from external electromagnetic sources
- Loss of adequate stimulation
- Pain, headache or discomfort.
- Skin irritation or burns at the stimulator site
- Chemical burns may result if the Vercise Stimulator housing is ruptured or pierced and your tissue is exposed to battery chemicals.
- Stiffness in muscles or with joint movement
- Swelling, including fluid collecting around the device

The stimulator lead may need to be re-positioned surgically if the clinical benefit has not been optimized or if programming the device causes intolerable side effects or side effects that cannot be avoided with reprogramming. Every effort is made to minimize the risks of the lead repositioning procedure. The complications of lead repositioning are similar to the risks associated with the initial procedure.

Risks Associated with the DBS System MRI Contraindication

Risk of Recurrent Stroke in Patients with a Prior Ischemic Stroke

MRI testing following DBS system implant is partially contraindicated. Based on the Cleveland Clinic experience, MRI sequences considered desirable in the investigation of suspected acute strokes will not be considered safe for these patients[155, 156]. Given the data on the risk of a recurrent stroke between 1 and 5 years after an initial ischemic stroke it is conservatively estimated that 1 patient in the study may have a recurrent stroke during the study (~12 x 10%). This estimate is based on the following review of the literature on the risk of recurrent stroke (Table 5).

Table 5. Literature Review on Risk of Recurrent Stroke Post Ischemic Stroke

Source	Rate
Lehigh Valley Recurrent Stroke Study[157]	12% with average follow-up of 24 months
Perth Community Stroke Study[158]	12.5% @ 1 year.<br>13.4% @ 2 years<br>17.7% @ 3 years<br>22.4% @ 5 years
Hillen et al[159]	total anterior circulation infarcts (TACI)

	5.8% @ 1 year<br>12.8% @ 3 years<br>21.5% @ 5 years<br>partial anterior circulation infarcts (PACI)<br>9.3% @ 1 year<br>16.3% @ 3 years<br>17.6% @ 5 years<br>posterior circulation infarcts (POCI)<br>9.5% @ 1 year<br>15.5% @ 3 years<br>15,5% @ 5 years
Brown et al[31]	11.8% @ 2 years
Petty et al[160]	atherosclerotic strokes with stenosis<br>24.4% @ 1 year<br>29.3% @ 2 years<br>40.2% at 5 years<br>cardio-embolic strokes<br>13.7% @ 1 year<br>16.8% @ 2 years<br>31.7% at 5 years<br>lacunar strokes<br>7.1% @ 1 year<br>11.6% @ 2 years<br>24.8% at 5 years<br>ischemic, unknown cause<br>13.2% @ 1 year<br>20.6% @ 2 years<br>33.2% at 5 years

Clinical Consequences of MRI Contraindication for Stroke Patients

The most important goal in the acute setting is to determine whether the patient is a candidate for thrombolytic therapy (i.e. tPA) or other acute interventions in a timely fashion.

The risk associated with having a contraindication for MRI for the patients in the proposed study is that an accurate diagnosis might be compromised because only CTs but not MRIs will be available to the patient. Available thrombolytic therapies or other interventions may be delayed or not provided.[13]

12.2.3 Risks Associated with Deep Brain Stimulation Therapy

Stimulation related adverse events are expected in 100% of the patients. During programming of the DBS systems, the amplitude will be increased in stepwise fashion until a side effect is noted or until the charge density limit is reached. Hence, the investigators will be deliberately seeking to find the threshold for

[13] Revised as per FDA feedback, August 16, 2015, Question 1i

adverse events in each individual. Chronic, irreversible stimulation related adverse events are rare. Most stimulation side effects are reversible and are corrected by reprogramming the neurostimulator or turning the neurostimulator off. A variety of stimulation-related adverse events have been reported for approved DBS stimulation brain studies, including:

- Confusion or problems with attention, thinking, or memory, speech or any other cognitive or behavioral problem. Pain, headache or discomfort
- Psychiatric disturbances such as anxiety, depression, lessened interest or emotion, mania, hypomania, emotional sensitivity, sleep problems, suicide, or suicidal thoughts or attempts, psychosis, hallucinations.
- Seizures, including epilepsy
- Sensory changes
- Speech or language problems, difficulty swallowing, as well as complications such as aspiration pneumonia
- Systemic effects such as rapid heartbeat, sweating, fever, dizziness, changes in kidney function, difficulty passing urine, sexual effects, nausea, difficulty having bowel movements, bloating
- Weakness, paralysis, muscle contractions, muscle spasms, shaking, restlessness, or problems with movement, walking, coordination, or body positioning, and falls or injuries resulting from these problems
- Undesirable sensations such as tingling or pain
- Visual problems, eyelid or eye movement difficulties or other eye-related symptoms
- Problems related to the function of any cranial nerve.
- Weight changes
- High levels of stimulation may damage brain tissue, including permanent damage.
- Effectiveness or comfort of stimulation changing over time. Temporary worsening of symptoms when stimulation is stopped, or "rebound."

In addition to these effects, based on the anatomy of the region to be stimulated (dentate nucleus), we expect that possible stimulation related side effects will be:

- Motor changes such as worsening of coordination, twitching or pulling, paresthesias, changes in balance or vertigo, modulation of mood[161]
- Laughing or relaxation, behavioral problems.[162]
- Reduction in tonus
- Oculomotor side effects[86, 87]
- Changes in cognitive function may also occur.

Given the early-phase nature of this study and the pooled literature experience from deep brain stimulation of the dentate nucleus, we currently cannot attribute specific rates to these potential stimulation related adverse events.

12.2.4 Risk Minimization

The risks associated with the DBS implant procedure have been minimized by the proposed implant procedures. The patient will be implanted in the prone or lateral

position, which is known to reduce the risk of air embolism compared to the sitting position. Careful direct brain targeting will be accomplished with state of the art stereotactic systems and supplemented with intraoperative tests to minimize the chance of missing the target. The risk of CSF leak is minimized by careful surgical technique and use of fibrin glue over the bur hole intraoperatively. Furthermore, only one functional neurosurgeon (study PI) will be performing the procedures. The Cleveland Clinic Foundation (CCF) has an experienced DBS team, and has performed over 1000 deep brain stimulation procedures in a variety of brain target areas for a variety of indications. CCF has rigorous operative procedures, and procedures for postoperative care to ensure complications of surgery and DBS stimulation are minimized.

The close safety reporting, monitoring by the investigator and sponsor, and oversight by the DMC in this first-in-human trial described in Sections 8, 9 and 10 of this protocol provide further risk mitigation measures to protect the safety, rights and welfare of enrolled patients.

12.3 Risk Benefit Analysis

While we believe that the risk for significant injury or death due DBS system implantation and electrical stimulation of the dentate nucleus will be equivalent to the risks of DBS implantation in other brain targets, these risks have yet to be adequately and fully quantified in the patient population under study.

Eligibility criteria that exclude subjects who are at higher risk for experiencing an anticipated Adverse Event have been selected in order to reduce the potential risks to subjects that participate in this study. The potential risk of further impairing function is greater in a moderately impaired subject compared to a more severely impaired individual. Furthermore, while subjects with more impairment may have less chance for benefit, it does not necessarily mean that they have no chance for any benefit from the therapy. Since this feasibility study is focused primarily on safety, the study will initially enroll more severely impaired subjects to provide initial safety information as described in §1.4.1Staged above. Specifically, only severely impaired subjects with a score of ≤ 20 on the UE Fugl-Meyer be initially enrolled in this study.

The proposed IDE feasibility study includes efforts to minimize the potential for risk, and is based on a well-defined clinical rationale. Stimulation of the dentate is expected to chronically activate the dentate-thalamo-cortical pathway and, consequently, enhance cortical excitability and facilitate plasticity, cortical reorganization and motor recovery of upper extremity hemiparesis.

13.0 MONITORING PROCEDURES

13.1 Monitoring Procedures

The Sponsor will have a dedicated clinical study monitor assigned to this study. He/she will be responsible for ensuring that appropriate monitoring of the study is performed.

The clinical study monitor will review data in submitted Case Report Forms as they are generated for accuracy and completeness as per data review process. Monitors will conduct visits to the Cleveland Clinic to ensure accuracy of data, timeliness of data submissions, adequate subject enrollment, investigational device accountability, compliance with applicable laws and regulations, compliance with the protocol, compliance with the signed investigator agreement, and compliance with IRB conditions and guidelines. Any non-compliance with these items will be discussed with the PI who will be responsible for ensuring that the non-compliance is adequately addressed with relevant corrective and preventative actions.

Because this is a first-in-human study, the clinical monitor plans to:

- Conduct training of all study personnel regarding the protocol, GCP compliance and study assessment procedures.
- Be present at all implant, TMS testing, and baseline FMA-UE assessment
- Be at study sites frequently to review documentation of informed consent, adherence to inclusion/exclusion criteria, case report forms, and adherence to study procedures, to assess compliance with the protocol and investigational product accountability

For the first subject, the clinical monitor plans to be at the clinical site for all monthly visits to verify accuracy and completeness of data from the CRF (as compared to source documents). In addition, safety data for the first implanted subject will be reported to the FDA early and frequent intervals.

13.1.1 Monitoring Reports

After each monitoring visit, the monitor will compile and send to the PI a report summarizing the monitoring visit. The PI will be responsible for ensuring that any follow-up actions needed to resolve issues are completed in an accurate and timely manner and corrective and preventative actions are implemented.

13.2 Final Site Visit

A final visit will be conducted at the close of the study. The purpose of the final visit is to collect all outstanding study data documents, ensure that the principal investigator’s files are accurate and complete, review record retention requirements, and ensure that all applicable requirements are met for the study.

14.0 COMPLIANCE

14.1 Statement of Compliance

This study will be conducted according to Good Clinical Practice (GCP) regulations and guidance issued by the Food and Drug Administration (FDA) which are included in the following parts of the FDA Code of Federal Regulations (CFR):

- 21 CFR Part 50: Protection of Human Subjects,
- 21 CFR Part 54: Financial Disclosure
- 21 CFR Part 56: Institutional Review Boards,
- 21 CFR Part 812: Investigational Device Exemptions

The purpose of these regulations is to define the standards and principles for the proper conduct of clinical studies. The ethical standards defined within GCP are intended to ensure that human subjects are provided with an adequate understanding of the possible risks of their participation in the study, and that they have a free choice to participate or not; the study is conducted with diligence and in conformance with the protocol in such a way as to ensure the integrity of the findings; and the potential benefits of the research justify the risks.

14.2 Device Accountability

Information on each DBS device used in the study will be documented. Device accountability for each device provided to the site and for each device used for the study will be maintained by the study site. Only approved investigators will implant devices in subjects participating in this study. During monitoring visits the study monitor will evaluate investigational device accountability. Any unused device will be returned to the Sponsor.

14.3 Sponsor Responsibilities

The sponsor is responsible for the following:

- ensuring the study is reviewed and approved by the FDA and that the study is compliant with the IDE regulations (21 CRF 812),
- ensuring the investigative center obtains IRB approval prior to initiating the study,
- selecting qualified investigators,
- obtaining a signed investigator's agreement,
- providing investigators with the information and training they need to properly conduct the study,
- ensuring patient informed consent is obtained,
- ensuring proper monitoring of the study,
- ensuring that the study is conducted according to the clinical protocol ,
- ensuring that the investigation treatment is made available only to qualified investigators participating in the study,

- ensuring no changes that effect the scientific soundness of the study or the rights safety and welfare of the subjects are made to the investigational plan without prior FDA and IRB approval,
- ensuring that regulatory agencies and all participating investigators are properly informed of significant new information regarding adverse effects or risks associated with the device being studied.
- ensure that adverse event are reported to FDA and IRB within the required timeframe.

14.3.1 Sponsor Records

The sponsor must maintain accurate, complete and current records relating to the study. These records include:

- Correspondence with another sponsor, study monitors, investigators, an IRB and FDA.
- Records of investigational device shipment and disposition
- Signed investigator agreements and financial disclosure if required under CFR 812.43(c)(5)
- Adverse device effects (whether anticipated or unanticipated) and complaints

14.3.2 Sponsor Reports

Refer to Table 6 for a tabular listing of sponsor reporting requirements. The sponsor must prepare and submit the following reports:

- Results of evaluation of any reported unanticipated adverse device effects (UADEs) to FDA, all IRBs, and investigators within 10 working days after notification by the investigator,
- current investigator list to FDA every 6 months,
- an annual progress report to FDA and IRBs,
- any withdrawals of IRB approval to FDA, all IRBs, and investigators, within 5 working days after receipt of notice of withdrawal of IRB approval,
- any withdrawal of FDA approval to the IRB, and investigators, within 5 working days after receipt of notice of withdrawal of FDA approval,
- any device withdrawals or recalls to FDA and IRB within 30 working days after the request is made to an investigator,
- Emergency protocol deviations to FDA within 5 working days after receipt of notice of such emergency use,
- Use of the investigational device or treatment without obtaining informed consent to FDA within 5 working days after sponsor is notified of such use,
- A final report to FDA, IRBs, and investigators within six months of completion or termination of the study.

14.3.3 Sponsor Inspections

The sponsor is required to permit FDA to enter and inspect any establishment where investigational devices are held and where records and results from use of the devices are kept. FDA may also inspect and copy all records relating to an

investigation including, in certain circumstances, records which identify subjects. Investigator Responsibilities

14.4 Investigator Responsibilities

An investigator is responsible for ensuring that the study is conducted according to the signed investigator agreement, the investigational plan, and applicable FDA regulations; for protecting the rights, safety, and welfare of subjects under the investigator's care; and for the control of devices under investigation. The investigator and clinical site is to allow and support Sponsor audits and inspections by FDA and other regulatory agencies.

14.4.1 Protection of Human Subjects

The investigator must submit the clinical investigational plan and the patient informed consent form to the governing Institutional Review Board (IRB) and obtain written approval from the IRB before enrolling patients in the study. The Investigator is also responsible for fulfilling any conditions of approval imposed by the IRB.

14.4.2 Investigator Records

The principal investigator is responsible for maintaining the following records for a period of two years following the termination or completion of the study. The principle investigator/center must maintain adequate records on all aspects of the study, including the following:

- IRB approvals
- Device disposition
- Informed Consent Forms
- Case Report Forms
- Adverse Event Form and information
- Protocol Deviations
- Correspondence file regarding study
- Subject termination information
- All study-related correspondence with the IRB, another investigator, Sponsor, Study Monitor, and regulatory agencies, including required reports;
- Records of receipt, use, and disposition of the test device system, including receipt dates, lot numbers, and final device disposition;
- Records of each subject’s case history, including information reported on all study-required Case Report Forms (CRFs), evidence of informed consent, all relevant observations of adverse events, the results of diagnostic testing, and the date of each study treatment.
- Copies of the approved clinical investigational plan and any amendments and documentation of any deviations from the clinical investigational plan including documented dates and reasons for each deviation.

14.4.3 Investigator Reports

Required investigator reports are listed in Table 6. The table also displays information regarding to whom this information is to be sent, and the frequency and time constraints around report submission. If applicable laws, regulations, or IRB requirements mandate stricter reporting requirements then those listed, the stricter requirements must be followed.

The Investigator is responsible for the following reports:

- Any serious adverse event (SAE) which has a possibility of being related to the device, stimulation, or study surgery, including all UADEs, are to be reported to the sponsor soon as possible but no later than 24 hours after the event is known to the Investigator,
- Unanticipated adverse device effects (UADEs) to be reported to the IRB within 10 days after the UADE is known to the Investigator),
- Withdrawal of IRB approval (to be reported to the Sponsor and FDA within five working days after the withdrawal of IRB approval is known to the investigator),
- Progress reports (provided to the Sponsor and IRB at regular intervals (as requested by the governing IRB) but no less than yearly),
- Deviations from the protocol. Use of the investigational device without informed consent (to be reported to the IRB within five working days after the use occurs).
- A final report (to be provided to the IRB within six months after termination or completion of the investigation).
- Other study-related reports (upon request by a reviewing IRB, study Clinical Events Committee, and/or the FDA).

Immediate reporting of SAEs which have a possibility of being device related is necessary to allow for further investigating of the SAE by the Sponsor and DMC when necessary to determine whether the event was caused by the device, stimulation, or study surgery.

14.4.4 Investigative Center Inspections

Regulatory agency may audit the investigational center. The purpose of an audit is to assess the accuracy, adequacy and consistency of the study records and subject data and to assess adherence to the procedures described in this clinical investigational plan. A typical audit visit will include the following:

- upon arrival, an interview with the investigator and study personnel,
- a tour of the facility,
- a review of the study records,
- a review of the case report forms and source documents,
- at the conclusion of the audit, a discussion of any key audit observations.

Table 6. Investigator and Sponsor Reporting Responsibilities

Type of Report	Principle Investigator/Sponsor Reporting Responsibilities			
	Report Prepared by Investigators For	Reporting Time Frame	Report Prepared by Sponsor For	Reporting Time Frame
Serious Adverse Events, which have a possibility of being related to the device, stimulation or study surgery.	Sponsor	Immediately, but no later than 24 hours after investigator is first notified of the event.	See below for UADE, if the result of investigation determines that the event is a UADE.	See below for UADE.
Unanticipated Adverse Device Effects	Sponsor and IRB	Immediately, but no later than 24 hours after investigator is first notified of the event.	FDA, IRBs, and Investigators	Within 10 working days of notifications of Unanticipated Adverse Device Effects, the event.
Withdrawal of IRB Approval or other action on part of the IRB that affects the study	Sponsor	Within 5 working days of notice of IRB decision.	FDA, IRBs, and Investigators	Within 5 working days of notification of such withdrawal.
Progress Reports	Sponsor, study monitors and IRB	At regular intervals, but no less than yearly.	FDA and IRBs	Annually on the anniversary of the IDE approval date
Deviations from Protocol	Sponsor and IRB	Emergency – ASAP but no later than 5 working days after the use occurs.<br><br>Other non-emergency protocol deviations require prior sponsor approval. If deviation occurs without approval – within 10 working days the deviation becomes known to the Investigator.	FDA	Emergency - Within 5 working days of notification of such emergency use.<br><br>Other non-emergency protocol deviations are reported in the FDA progress and final reports.
Use of an Investigational Device or Treatment without Informed Consent	Sponsor and IRB	Within 5 working days after the deviation occurs.	FDA	Within 5 working days after notification of such use.
Withdrawal of FDA approval	N/A	N/A	IRBs and Investigators	Within 5 working days of notification of such withdrawal.
Current Investigator List	N/A	N/A	FDA	Every six months

Device Recall and Disposition	N/A	N/A	FDA and IRBs	Within 30 working days after a device recall or withdrawal request is made to an investigator
Final Report	Sponsor and IRB	Within 3 months after termination or completion of study or investigator's participation.	FDA	Within 6 months of completion or termination of the study.
Other	As Required	Upon request by the IRB or FDA, provide accurate, complete, and current information about any aspect of the study.		Upon request by the IRB or FDA, provide accurate, complete, and current information about any aspect of the study.

15.0 INFORMED CONSENT MATERIALS

Informed consent will be obtained from each study participant. The investigator and/or the study coordinator will approach the potential subject, verbally explain the nature of the study, and provide an FDA and IRB approved written informed consent document for his/her review and signature. The investigator and/or study coordinator will be available to answer all questions the subject may have about the study. In addition, Dr. Paul Ford (Cleveland Clinic Bioethics) or one of his designees will participate in the study as a consent monitor.[14] The informed consent document includes all the required elements as outlined in 21 CFR Part 50.25.

16.0 REFERENCES

1 Go, A.S., et al., *Heart disease and stroke statistics--2014 update: a report from the american heart association.* Circulation, 2014. **129**(3): p. e28-e292.

2 Kelly-Hayes M, e.a., *The influence of age and gender on disability following ischemic stroke. The Framingham study.* . J Stroke Cerebrovasc Dis., 2003. **12**: p. 119-126.

3 Sudlow, C.L. and C.P. Warlow, *Comparing stroke incidence worldwide: what makes studies comparable?* Stroke, 1996. **27**(3): p. 550-8.

4 Go, A.S., et al., *Executive summary: heart disease and stroke statistics--2014 update: a report from the american heart association.* Circulation, 2014. **129**(3): p. 399-410.

5 Rosamond, W.D., et al., *Stroke incidence and survival among middle-aged adults: 9-year follow-up of the Atherosclerosis Risk in Communities (ARIC) cohort.* Stroke, 1999. **30**(4): p. 736-43.

6 El-Saed, A., et al., *Geographic variations in stroke incidence and mortality among older populations in four US communities.* Stroke, 2006. **37**(8): p. 1975-9.

[14] As per discussion at the pre-sub meeting, July 20, 2015.

7 Goldstein, L.B., *Acute ischemic stroke treatment in 2007.* Circulation, 2007. **116**(13): p. 1504-14.

8 Granger, C.V., B.B. Hamilton, and G.E. Gresham, *The stroke rehabilitation outcome study--Part I: General description.* Arch Phys Med Rehabil, 1988. **69**(7): p. 506-9.

9 Gresham, G.E., *Stroke outcome research.* Stroke, 1986. **17**(3): p. 358-60.

10 van Kordelaar, J., E. van Wegen, and G. Kwakkel, *Impact of time on quality of motor control of the paretic upper limb after stroke.* Arch Phys Med Rehabil, 2014. **95**(2): p. 338-44.

11 Kwakkel, G., B. Kollen, and J. Twisk, *Impact of time on improvement of outcome after stroke.* Stroke, 2006. **37**(9): p. 2348-53.

12 Plow, E.B., et al., *Rethinking Stimulation of the Brain in Stroke Rehabilitation: Why Higher Motor Areas Might Be Better Alternatives for Patients with Greater Impairments.* Neuroscientist, 2014.

13 Butler, A.J. and S.L. Wolf, *Transcranial magnetic stimulation to assess cortical plasticity: a critical perspective for stroke rehabilitation.* J Rehabil Med, 2003(41 Suppl): p. 20-6.

14 Kollen, B., G. Kwakkel, and E. Lindeman, *Functional recovery after stroke: a review of current developments in stroke rehabilitation research.* Rev Recent Clin Trials, 2006. **1**(1): p. 75-80.

15 Duncan, P.W., et al., *Management of Adult Stroke Rehabilitation Care: a clinical practice guideline.* Stroke, 2005. **36**(9): p. e100-43.

16 Veerbeek JM, v.W.E., van Peppen R, van der Wees PJ, Nendriks E, Rietberg M, Kwakkel G, *What is the evidence for physical therapy poststroke; A systematic review and meta-analysis.* PLoS One, 2014. **9**(2).

17 Hafsteinsdottir TB, A.A., Kappelle LJ, Grypdonck MHF, *Neurodevelopmental treatment after stroke: A comparative study.* Journal of Neurology, Neurosurgery & Psychiatry, 2005. **76**: p. 788-792.

18 Kwakkel G, B.J., van Wegen EEH, Wolf SL, *Constraint-induced movement therapy after stroke: A systematic review and meta-analysis of current evidence.* . Lancet (Neurology), 2015. **14**: p. 224-234.

19 Reiss A, B.S., Wolf SL, *Contemporary Concepts in Upper Extremity Rehabilitation,*, in *Textbook of Neural Repair and Rehabilitation,*. 2014, Cambridge University Press: Cambridge. p. 330-342.

20 Wolf, S.L., et al., *Effect of constraint-induced movement therapy on upper extremity function 3 to 9 months after stroke: the EXCITE randomized clinical trial.* JAMA, 2006. **296**(17): p. 2095-104.

21 Hayward, K., R. Barker, and S. Brauer, *Interventions to promote upper limb recovery in stroke survivors with severe paresis: a systematic review.* Disabil Rehabil, 2010. **32**(24): p. 1973-86.

22 Fregni, F., et al., *A sham-controlled trial of a 5-day course of repetitive transcranial magnetic stimulation of the unaffected hemisphere in stroke patients.* Stroke, 2006. **37**(8): p. 2115-22.

23 Kim, Y.H., et al., *Repetitive transcranial magnetic stimulation-induced corticomotor excitability and associated motor skill acquisition in chronic stroke.* Stroke, 2006. **37**(6): p. 1471-6.

24 Tsubokawa, T., et al., *Chronic motor cortex stimulation in patients with thalamic pain.* J Neurosurg, 1993. **78**(3): p. 393-401.

25 Machado, A., H. Azmi, and A.R. Rezai, *Motor cortex stimulation for refractory benign pain.* Clin Neurosurg, 2007. **54**: p. 70-7.

26 Adkins, D.L., J.E. Hsu, and T.A. Jones, *Motor cortical stimulation promotes synaptic plasticity and behavioral improvements following sensorimotor cortex lesions.* Exp Neurol, 2008. **212**(1): p. 14-28.

27 Adkins, D.L., et al., *Epidural cortical stimulation enhances motor function after sensorimotor cortical infarcts in rats.* Exp Neurol, 2006. **200**(2): p. 356-70.

28 Adkins-Muir, D.L. and T.A. Jones, *Cortical electrical stimulation combined with rehabilitative training: enhanced functional recovery and dendritic plasticity following focal cortical ischemia in rats.* Neurol Res, 2003. **25**(8): p. 780-8.

29 Plautz, E.J., et al., *Post-infarct cortical plasticity and behavioral recovery using concurrent cortical stimulation and rehabilitative training: a feasibility study in primates.* Neurol Res, 2003. **25**(8): p. 801-10.

30 Robert Levy, M.D., Ph.D., Sean Ruland, D.O., Martin Weinand, M.D., David Lowry, M.D., Rima Dafer, M.D., M.P.H., and Roy Bakay, M.D., *Cortical stimulation for the rehabilitation of patients with hemiparetic stroke: a multicenter feasibility study of safety and efficacy.* Journal of Neurosurgery, 2008. **108**(4).

31 Brown, J.A.M.D.L., Helmi L. M.D.; Weinand, Martin M.D.; Cramer, Steven C. M.D., *Motor Cortex Stimulation for the Enhancement of Recovery from Stroke: A Prospective, Multicenter Safety Study.* Neurosurgery, 2006. **58**(3).

32 Plow, E.B., et al., *Invasive cortical stimulation to promote recovery of function after stroke: a critical appraisal.* Stroke, 2009. **40**(5): p. 1926-31.

33 Holsheimer, J., et al., *Cathodal, anodal or bifocal stimulation of the motor cortex in the management of chronic pain?* Acta Neurochir Suppl, 2007. **97**(Pt 2): p. 57-66.

34 Manola, L., et al., *Anodal vs cathodal stimulation of motor cortex: a modeling study.* Clin Neurophysiol, 2007. **118**(2): p. 464-74.

35 Kwakkel, G., et al., *Probability of regaining dexterity in the flaccid upper limb: impact of severity of paresis and time since onset in acute stroke.* Stroke, 2003. **34**(9): p. 2181-6.

36 Benabid, A.L., et al., *Long-term suppression of tremor by chronic stimulation of the ventral intermediate thalamic nucleus.* Lancet, 1991. **337**(8738): p. 403-6.

37 Schiff, N.D., et al., *Behavioural improvements with thalamic stimulation after severe traumatic brain injury.* Nature, 2007. **448**(7153): p. 600-3.

38 Machado, A.G., et al., *Chronic electrical stimulation of the contralesional lateral cerebellar nucleus enhances recovery of motor function after cerebral ischemia in rats.* Brain Res, 2009. **1280**: p. 107-16.

39 Malone, D.A., Jr., et al., *Deep brain stimulation of the ventral capsule/ventral striatum for treatment-resistant depression.* Biol Psychiatry, 2009. **65**(4): p. 267-75.

40 Greenberg, B.D., et al., *Deep brain stimulation of the ventral internal capsule/ventral striatum for obsessive-compulsive disorder: worldwide experience.* Mol Psychiatry, 2010. **15**(1): p. 64-79.

41 Ghosh, P.S., et al., *Deep brain stimulation in children with dystonia: experience from a tertiary care center.* Pediatr Neurosurg, 2012. **48**(3): p. 146-51.

42 Giacino, J., et al., *Central thalamic deep brain stimulation to promote recovery from chronic posttraumatic minimally conscious state: challenges and opportunities.* Neuromodulation, 2012. **15**(4): p. 339-49.

43 Plow, E.B., A. Pascual-Leone, and A. Machado, *Brain stimulation in the treatment of chronic neuropathic and non-cancerous pain.* J Pain, 2012. **13**(5): p. 411-24.

44 Lim, T.T., et al., *Successful deep brain stimulation surgery with intraoperative magnetic resonance imaging on a difficult neuroacanthocytosis case: case report.* Neurosurgery, 2013. **73**(1): p. E184-7; discussion E188.

45 Schrock, L.E., et al., *Tourette syndrome deep brain stimulation: A review and updated recommendations.* Mov Disord, 2014.

46 Lega, B.C., et al., *Deep brain stimulation in the treatment of refractory epilepsy: update on current data and future directions.* Neurobiol Dis, 2010. **38**(3): p. 354-60.

47 Sprengers, M., et al., *Deep brain and cortical stimulation for epilepsy.* Cochrane Database Syst Rev, 2014. **6**: p. CD008497.

48 Pantano, P., et al., *Crossed cerebellar diaschisis.* Brain, 1986. **109**: p. 677 - 694.

49 Yamauchi, H., H. Fukuyama, and J. Kimura, *Hemodynamic and metabolic changes in crossed cerebellar hypoperfusion.* Stroke, 1992. **23**(6): p. 855-60.

50 Shamoto, H. and H.T. Chugani, *Glucose metabolism in the human cerebellum: an analysis of crossed cerebellar diaschisis in children with unilateral cerebral injury.* J Child Neurol, 1997. **12**(7): p. 407-14.

51 Yamauchi, H., et al., *A decrease in regional cerebral blood volume and hematocrit in crossed cerebellar diaschisis.* Stroke, 1999. **30**(7): p. 1429-31.

52 Brunberg, J.A., et al., *Crossed cerebellar diaschisis: occurrence and resolution demonstrated with PET during carotid temporary balloon occlusion.* AJNR Am J Neuroradiol, 1992. **13**(1): p. 58-61.

53 Tanaka, M., et al., *Crossed cerebellar diaschisis accompanied by hemiataxia: a PET study.* J Neurol Neurosurg Psychiatry, 1992. **55**(2): p. 121-5.

54 Kim, S.E. and M.C. Lee, *Cerebellar vasoreactivity in stroke patients with crossed cerebellar diaschisis assessed by acetazolamide and 99mTc-HMPAO SPECT.* J Nucl Med, 2000. **41**(3): p. 416-20.

55 Suga, K., et al., *Crossed cerebellar diaschisis demonstrated by brain SPECT with Tc-99m MAA in a child with an intracardiac right-to-left shunt.* Clin Nucl Med, 1999. **24**(4): p. 282-3.

56 Pantano, P., et al., *Crossed cerebellar diaschisis in patients with cerebral ischemia assessed by SPECT and 123I-HIPDM.* Eur Neurol, 1987. **27**(3): p. 142-8.

57 Flores, L.G., 2nd, et al., *Crossed cerebellar diaschisis: analysis of iodine-123-IMP SPECT imaging.* J Nucl Med, 1995. **36**(3): p. 399-402.

58 Miyazawa, N., et al., *Evaluation of crossed cerebellar diaschisis in 30 patients with major cerebral artery occlusion by means of quantitative I-123 IMP SPECT.* Ann Nucl Med, 2001. **15**(6): p. 513-9.

59 Dum, R.P., C. Li, and P.L. Strick, *Motor and nonmotor domains in the monkey dentate.* Ann N Y Acad Sci, 2002. **978**: p. 289-301.

60 Dum, R.P. and P.L. Strick, *Motor areas in the frontal lobe of the primate.* Physiol Behav, 2002. **77**(4-5): p. 677-82.

61 Dum, R.P. and P.L. Strick, *An unfolded map of the cerebellar dentate nucleus and its projections to the cerebral cortex.* J Neurophysiol, 2003. **89**(1): p. 634-9.

62 Takasawa, M., et al., *Prognostic value of subacute crossed cerebellar diaschisis: single-photon emission CT study in patients with middle cerebral artery territory infarct.* AJNR Am J Neuroradiol, 2002. **23**(2): p. 189-93.

63 Machado, A. and K.B. Baker, *Upside down crossed cerebellar diaschisis: proposing chronic stimulation of the dentatothalamocortical pathway for post-stroke motor recovery.* Front Integr Neurosci, 2012. **6**: p. 20.

64 Spiegel, E.A., et al., *Stereotaxic Apparatus for Operations on the Human Brain.* Science, 1947. **106**(2754): p. 349-50.

65 Spiegel, E.A. and H.T. Wycis, *Mesencephalothalamotomy for relief of intractable pain.* Am J Med, 1949. **6**(4): p. 504.

66 Spiegel, E.A. and H.T. Wycis, *Electroencephalographic studies following thalamic lesions in humans.* Fed Proc, 1948. **7**(1 Pt 1): p. 119.

67 Spiegel, E.A., H.T. Wycis, and et al., *Stereoencephalotomy.* Proc Soc Exp Biol Med, 1948. **69**(1): p. 175-7.

68 Dieckmann, G. and R. Hassler, *Stereotaxic treatment of extrapyramidal myoclonus.* Confin Neurol, 1972. **34**(1): p. 57-63.

69 Hassler, R. and G. Dieckmann, *[Stereotaxic treatment of tics and inarticulate cries or coprolalia considered as motor obsessional phenomena in Gilles de la Tourette's disease].* Rev Neurol (Paris), 1970. **123**(2): p. 89-100.

70 Hassler, R. and G. Dieckmann, *Stereotactic treatment of different kinds of spasmodic torticollis.* Confin Neurol, 1970. **32**(2): p. 135-43.

71 Laitinen, L.V., A.T. Bergenheim, and M.I. Hariz, *Leksell's posteroventral pallidotomy in the treatment of Parkinson's disease.* J Neurosurg, 1992. **76**(1): p. 53-61.

72 Laitinen, L.V., *Brain targets in surgery for Parkinson's disease. Results of a survey of neurosurgeons.* J Neurosurg, 1985. **62**(3): p. 349-51.

73 Laitinen, L.V. and Y. Ohno, *Effects of thalamic stimulation and thalamotomy on the H reflex.* Electroencephalogr Clin Neurophysiol, 1970. **28**(6): p. 586-91.

74 Laitinen, L., *Thalamic targets in the stereotaxic treatment of Parkinson's disease.* J Neurosurg, 1966. **24**(1): p. 82-5.

75 Johansson, G. and L. Laitinen, *Electrical stimulation of the thalamic and subthalamic area in Parkinson's disease.* Confin Neurol, 1965. **26**(3): p. 445-50.

76 Laitinen, L.V., *Leksell's unpublished pallidotomies of 1958-1962.* Stereotact Funct Neurosurg, 2000. **74**(1): p. 1-10.

77 Leksell, L., *Stereotactic apparatus for intracerebral surgery.* acta chir scand, 1949. **99**: p. 229-33.

78 Talairach, J. and G. Szikla, *Atlas of stereotactic concepts to the surgery of epilepsy.* 1967.

79 Talairach, J. and P. Tournoux, *[Stereotaxic localization of central gray nuclei].* Neurochirurgia (Stuttg), 1958. **1**(1): p. 88-93.

80 Alesch, F., et al., *Stimulation of the ventral intermediate thalamic nucleus in tremor dominated Parkinson's disease and essential tremor.* Acta Neurochir (Wien), 1995. **136**(1-2): p. 75-81.

81 Tasker, R.R., et al., *Deep brain stimulation and thalamotomy for tremor compared.* Acta Neurochir Suppl, 1997. **68**: p. 49-53.

82 Pahwa, R., et al., *Comparison of thalamotomy to deep brain stimulation of the thalamus in essential tremor.* Mov Disord, 2001. **16**(1): p. 140-3.

83 Siegfried, J. and J.C. Verdie, *Long-term assessment of stereotactic dentatotomy for spasticity and other disorders.* Acta Neurochir (Wien), 1977(Suppl 24): p. 41-8.

84 Krayenbuhl, H. and J. Siegfried, *Dentatotomies or thalamotomies in the treatment of hyperkinesia.* Confin Neurol, 1972. **34**(2): p. 29-33.

85 Slaughter, D.G., B.S. Nashold, Jr., and G.G. Somjen, *Electrical recording with micro- and macroelectrodes from the cerebellum of man.* J Neurosurg, 1970. **33**(5): p. 524-8.

86 Nashold, B.S., Jr. and D.G. Slaughter, *Effects of stimulating or destroying the deep cerebellar regions in man.* J Neurosurg, 1969. **31**(2): p. 172-86.

87 Nasold, B.S., Jr., D.G. Slaughter, Jr., and J. Harrison, *A stereotaxic approach and evaluation of the cerebellar nuclei of man.* Confin Neurol, 1969. **31**(1): p. 56.

88 Slaughter, D.G. and B.S. Nashold, Jr., *Stereotactic coordinates for the human dentate nucleus.* Confin Neurol, 1968. **30**(5): p. 375-84.

89 Schvarcz, J.R., R.E. Sica, and E. Morita, *Chronic self-stimulation of the dentate nucleus for the relief of spasticity.* Acta Neurochir Suppl (Wien), 1980. **30**: p. 351-9.

90 Schvarcz, J.R., *Stimulation of the dentate nuclei for spasticity.* Acta Neurochir Suppl (Wien), 1987. **39**: p. 124-5.

91 Sica, R.E., et al., *Changes in the excitability of segmental moton eurone polls by chronic cerebellar dentate nucleus stimulation.* Electromyogr Clin Neurophysiol, 1984. **24**(3): p. 163-76.

92 Schvarcz, J.R., et al., *Electrophysiological changes induced by chronic stimulation of the dentate nuclei for cerebral palsy.* Appl Neurophysiol, 1982. **45**(1-2): p. 55-61.

93 Galanda, M. and S. Horvath, *Effect of stereotactic high-frequency stimulation in the anterior lobe of the cerebellum in cerebral palsy: a new suboccipital approach.* Stereotact Funct Neurosurg, 2003. **80**(1-4): p. 102-7.

94 Galanda, M. and S. Horvath, *Stereotactic stimulation of the anterior lobe of the cerebellum in cerebral palsy from a suboccipital approach.* Acta Neurochir Suppl, 2007. **97**(Pt 2): p. 239-43.

95 Adkins-Muir DL, J.T., *Cortical electrical stimulation combined with rehabilitative training: enhanced functional recovery and dendritic plasticity following focal cortical ischemia in rats.* Neurol Res., 2003. **60**(4): p. 447-55.

96 Allred, R.P. and T.A. Jones, *Unilateral ischemic sensorimotor cortical damage in female rats: forelimb behavioral effects and dendritic structural plasticity in the contralateral homotopic cortex.* Exp Neurol, 2004. **190**(2): p. 433-45.

97 Kleim, J.A., J.A. Boychuk, and D.L. Adkins, *Rat models of upper extremity impairment in stroke.* ILAR J, 2007. **48**(4): p. 374-84.

98 Park, H.J., et al., *Semi-automated method for estimating lesion volumes.* J Neurosci Methods, 2013. **213**(1): p. 76-83.

99 Windle, V., et al., *An analysis of four different methods of producing focal cerebral ischemia with endothelin-1 in the rat.* Exp Neurol, 2006. **201**(2): p. 324-34.

100 Yanamoto, H., et al., *Evaluation of MCAO stroke models in normotensive rats: standardized neocortical infarction by the 3VO technique.* Exp Neurol, 2003. **182**(2): p. 261-74.

101 Luke, L.M., R.P. Allred, and T.A. Jones, *Unilateral ischemic sensorimotor cortical damage induces contralesional synaptogenesis and enhances skilled reaching with the ipsilateral forelimb in adult male rats.* Synapse, 2004. **54**(4): p. 187-99.

102 Yanamoto, H., et al., *Three-vessel occlusion using a micro-clip for the proximal left middle cerebral artery produces a reliable neocortical infarct in rats.* Brain Res Brain Res Protoc. , 1998. **3**(4): p. 209-20.

103 DeBow, S.B., et al., *Constraint-induced movement therapy and rehabilitation exercises lessen motor deficits and volume of brain injury after striatal hemorrhagic stroke in rats.* Stroke, 2003. **34**(4): p. 1021-6.

104 Hudzik, T.J., et al., *Long-term functional end points following middle cerebral artery occlusion in the rat.* Pharmacol Biochem Behav, 2000. **65**(3): p. 553-62.

105 Montoya, C.P., et al., *The "staircase test": a measure of independent forelimb reaching and grasping abilities in rats.* Journal of neuroscience methods., 1991. **36**(2-3): p. 219-28.

106 Rasmussen, R.S., et al., *Effects of microplasmin on recovery in a rat embolic stroke model.* Neurol Res, 2008. **30**(1): p. 75-81.

107 Tuor, U.I., et al., *Long-term deficits following cerebral hypoxia-ischemia in four-week-old rats: correspondence between behavioral, histological, and magnetic resonance imaging assessments.* Exp Neurol, 2001. **167**(2): p. 272-81.

108 Ballermann, M., et al., *The pasta matrix reaching task: a simple test for measuring skilled reaching distance, direction, and dexterity in rats.* J Neurosci Methods, 2001. **106**(1): p. 39-45.

109 Chiken, S. and H. Tokuno, *Impairment of skilled forelimb use after ablation of striatal interneurons expressing substance P receptors in rats: an analysis using a pasta matrix reaching task.* Exp Brain Res., 2005. **162**(4): p. 532-6.

110 Hicks, A.U., et al., *Enriched environment enhances transplanted subventricular zone stem cell migration and functional recovery after stroke.* Neuroscience, 2007. **146**(1): p. 31-40.

111 Colbourne, F., et al., *Prolonged but delayed postischemic hypothermia: a long-term outcome study in the rat middle cerebral artery occlusion model.* J Cereb Blood Flow Metab, 2000. **20**(12): p. 1702-8.

112 Kerr, A.L. and T.K. A., *Compensatory limb use and behavioral assessment of motor skill learning following sensorimotor cortex injury in a mouse model of ischemic stroke.* J Vis Exp. , 2014(89).

113 Angaut, P. and F. Cicirata, *Dentate control pathways of cortical motor activity. Anatomical and physiological studies in rat: comparative considerations.* Arch Ital Biol, 1990. **128**(2-4): p. 315-30.

114 Rispal-Padel, L., F. Cicirata, and C. Pons, *Contribution of the dentato-thalamo-cortical system to control of motor synergy.* Neurosci Lett, 1981. **22**(2): p. 137-44.

115 Panto MR, Z.A., Parenti R, Serapide MF, Cicirata F., *Corticonuclear projections of the cerebellum preserve both anteroposterior and mediolateral pairing patterns.* Eur J Neurosci., 2001. **13**(4): p. 694-708.

116 Cicirata, F., et al., *Multiple representation in the nucleus lateralis of the cerebellum: an electrophysiologic study in the rat.* Exp Brain Res, 1992. **89**(2): p. 352-62.

117 Angaut, P. and F. Cicirata, *The dentatorubral projection in the rat: an autoradiographic study.* Behav Brain Res, 1988. **28**(1-2): p. 71-3.

118 Angaut, P., F. Cicirata, and F. Serapide, *Topographic organization of the cerebellothalamic projections in the rat. An autoradiographic study.* Neuroscience, 1985. **15**(2): p. 389-401.

119 Massion, J. and L. Rispal-Padel, *Spatial organization of the cerebello-thalamo-cortical pathway.* Brain Res, 1972. **40**(1): p. 61-5.

120 Meftah, E.M. and L. Rispal-Padel, *Cerebello-cortical plasticity. Role of somesthetic influx in the change of the cerebellar effects on the musculature.* C R Acad Sci III, 1995. **318**(2): p. 219-27.

121 Baker, K.B., et al., *Deep brain stimulation of the lateral cerebellar nucleus produces frequency-specific alterations in motor evoked potentials in the rat in vivo.* Exp Neurol, 2010. **226**(2): p. 259-64.

122 Furmaga, H., et al., *Effects of ketamine and propofol on motor evoked potentials elicited by intracranial microstimulation during deep brain stimulation.* Front Syst Neurosci, 2014. **8**: p. 89.

123 Machado, A.G., et al., *Chronic 30-Hz deep cerebellar stimulation coupled with training enhances post-ischemia motor recovery and peri-infarct synaptophysin expression in rodents.* Neurosurgery, 2013. **73**(2): p. 344-53; discussion 353.

124 Cooperrider, J., et al., *Chronic deep cerebellar stimulation promotes long-term potentiation, microstructural plasticity, and reorganization of perilesional cortical representation in a rodent model.* J Neurosci, 2014. **34**(27): p. 9040-50.

125 Platz, T., Pinkowski, C., et al., *Reliability and validity of arm function assessment with standardized guidelines for the Fugl-Meyer Test, Action Research Arm Test and Box and Block Test: a multicentre study.* Clin Rehabil 2005. **19**(4): p. 404-411.

126 Sanford, J., Moreland, J., et al., *Reliability of the Fugl-Meyer assessment for testing motor performance in patients following stroke.* Phys Ther, 1993. **73**: p. 447-454.

127 Dettmann, M.A., Linder, M. T., et al., *Relationships among walking performance, postural stability, and functional assessments of the hemiplegic patient.* Am J Phys Med 1987. **66**: p. 77-90.

128 Hsieh, Y.W., Wu, C. Y., et al., *Responsiveness and validity of three outcome measures of motor function after stroke rehabilitation.* Stroke, 2009. **40**(4): p. 1386-1391.

129 Lin, J.H., Hsueh, I. P., et al., *Psychometric properties of the sensory scale of the Fugl-Meyer Assessment in stroke patients.* Clin Rehabil, 2004. **18**(4): p. 391-397.

130 Duncan, P.W., Lai, S. M., et al., *Defining post-stroke recovery: implications for design and interpretation of drug trials.* Neuropharmacology, 2000. **39**(5): p. 835-841.

131 Chen, H.M., Chen, C. C., et al., *Test-retest reproducibility and smallest real difference of 5 hand function tests in patients with stroke.* Neurorehabilitation and Neural Repair, 2009. **23**(5): p. 435-440.

132 Beebe, J.A.a.L., C. E. , *Relationships and responsiveness of six upper extremity function tests during the first six months of recovery after stroke.* J Neurol Phys Ther, 2009. **33**(2): p. 96-103.

133 JL, A. and W. SL, *The use of kinetics as a marker for manual dexterity after stroke and stroke recovery. .* Topics in stroke rehabilitation, 2099. **16**(4): p. 223-36.

134 Gorniak SL, A.J., *Effects of aging on force coordination in bimanual task performance.* Exp Brain Res., 2013. **229**(2): p. 273-84.

135 Gorniak SL, P.M., McDaniel C, Alberts JL, *Impaired Object Handling during Bimanual Task Performance in Multiple Sclerosis.* Multiple Sclerosis International, 2014. **2014**.

136 Rudick RA, M.D., Béthoux F, Rao SM, Lee J-C, Stough D, Reece C, Schindler D, Mamone B, Alberts J, *The Multiple Sclerosis Performance Test (MSPT): An iPad-Based Disability Assessment Tool.* JoVE, 2014(88).

137 Kopp, B., Kunkel, A., et al., *The Arm Motor Ability Test: reliability, validity, and sensitivity to change of an instrument for assessing disabilities in activities of daily living.* Arch Phys Med Rehabil 1997. **78**(6): p. 615-620.

138 Chae, J., Labatia, I., et al., *Upper limb motor function in hemiparesis: concurrent validity of the Arm Motor Ability test.* Am J Phys Med Rehabil 2003. **82**(1): p. 1-8.

139 Anderson, C., Laubscher, S., et al., *Validation of the Short Form 36 (SF-36) health survey questionnaire among stroke patients.* Stroke, 1996. **27**: p. 1812-1816.

140 Dorman, P., Slattery, J., et al., *Qualitative comparison of the reliability of health status assessments with the EuroQol and SF-36 questionnaires after stroke. United Kingdom Collaborators in the International Stroke Trial.* Stroke, 1998. **29**: p. 63-68.

141 Dorman, P.J., Dennis, M., et al., *How do scores on the EuroQol relate to scores on the SF-36 after stroke?* Stroke, 1999. **30**: p. 2146-2151.

142 Pinto EB1, M.I., Vilela RN, Santos LC, Oliveira-Filho J., *Validation of the EuroQol quality of life questionnaire on stroke victims.* Arq Neuropsiquiatr, 2011. **69**(2B): p. 320-3.

143 Kotila, M., Numminen, H., et al., *Depression after stroke: results of the FINNSTROKE Study.* Stroke, 1998. **29**: p. 368-372.

144 Aben, I., Verhey, F., et al., *Validity of the Beck Depression Inventory, Hospital Anxiety and Depression Scale, SCL-90, and Hamilton Depression Rating Scale as screening instruments for depression in stroke patients.* Psychosomatics 2002. **43**(5): p. 386.

145 Butler AJ, W.S., *Putting the brain on the map: Use of Transcranial magnetic stimulation to assess and induce cortical plasticity of upper-extremity movement. .* Physical Therapy. , 2007. **87**: p. 719-736.

146 *MagPro R30 Magnetic Stimulator Insturctions for Use reference.*

147 Kuhn, A.A., et al., *Modulation of motor cortex excitability by pallidal stimulation in patients with severe dystonia.* Neurology, 2003. **60**(5): p. 768-74.

148 Rossi, S., et al., *Safety, ethical considerations, and application guidelines for the use of transcranial magnetic stimulation in clinical practice and research.* Clin Neurophysiol., 2009. **120**(12): p. 2008-2039.

149 Kuhn, A.A., et al., *Pseudo-bilateral hand motor responses evoked by transcranial magnetic stimulation in patients with deep brain stimulators.* Clin Neurophysiol, 2002. **113**(3): p. 341-5.

150 Hidding, U., et al., *MEP latency shift after implantation of deep brain stimulation systems in the subthalamic nucleus in patients with advanced Parkinson's disease.* Mov Disord, 2006. **21**(9): p. 1471-6.

151 Cunningham, D.A., et al., *Assessment of Inter-Hemispheric Imbalance Using Imaging and Noninvasive Brain Stimulation in Patients With Chronic Stroke.* Archives of Physical Medicine and Rehabilitation, 2015. **96**: p. S94-103.

152 Angelis, C.D., et al., *Clinical Trial Registration: A Statement from the International Committee of Medical Journal Editors.* N Engl J Med 2004. **351**: p. 1250-1251.

153 *Medtronic DBS Therapy for Parkinson's Disease and Essential Tremor Clinical Summary.* 2013.

154 Lyons KE, W.S., Overman J, et al., *Surgical and hardware complications of subthalamic stimulation: a series of 160 procedures. .* Neurology., 2004. **63**: p. 612-616.

155 Rezai AR, P.M., Baker KB, Sharan AD, Nyenhuis J, Tkach J, et al., *Neurostimulation system used for deep brain stimulation (DBS): MR safety issues and implications of failing to follow safety recommendations.* Invest Radiol, 2004. **39**(5): p. 300-3.

156 Sharan A, R.A., Nyenhuis JA, Hrdlicka G, Tkach J, Baker K, et al. , *MR safety in patients with implanted deep brain stimulation systems (DBS). .* Acta Neurochir Suppl., 2003. **87**: p. 141-5.

157 Alter, M., et al., *The Lehigh Valley Recurrent Stroke Study: description of design and methods.* Neuroepidemiology., 1993. **12**(2): p. 241-8.

158 Hankey RJ, J.K., Broadhurst RJ, et al., *Long-term risk of first recurrent stroke in the Perth Community Stroke Study. .* Stroke, 1998. **29**: p. 2491-2500.

159 Hillen T, C.C., Tilling K, et al., *Cause of stroke recurrence is multifactorial.* Stroke, 2003. **34**: p. 1457-1463.

160 Petty GW, B.R., Whisnant JP, et al., *Ischemia stroke subtypes: a population-based study of functional outcome, survival, and recurrence.* Stroke, 2000. **31**: p. 1062-1068.

161 Galanda, M., L. Mistina, and O. Zoltan, *Behavioural responses to cerebellar stimulation in cerebral palsy.* Acta Neurochir Suppl (Wien), 1989. **46**: p. 37-8.

162 Galanda, M. and O. Zoltán, *Motor and psychological responses to deep cerebellar stimulation in cerebral palsy (correlation with organization of cerebellum into zones).* Acta Neurochir Suppl (Wien), 1987. **39**: p. 129-31.

Electrical Stimulation of the Dentate Nucleus area (EDEN) for Improvement of Upper Extremity Hemiparesis due to Ischemic Stroke: A Safety and Feasibility Study

Protocol Number: REDD 0002

Dec 29, 2020

Sponsor Information:
Enspire DBS Therapy, Inc.
2 Oliver St. STE 616
Boston, MA 02446

Contact Person:
[redacted]

PROTOCOL SYNOPSIS

Electrical Stimulation of the Dentate Nucleus area (EDEN)<br>for Improvement of Upper Extremity Hemiparesis due to Ischemic Stroke:<br>A Safety and Feasibility Study	
**Study Overview**	
**Study Objective**	The objective of this study is to document the safety and patient outcomes of electrical stimulation of the dentate nucleus area for the management of chronic, moderate to severe upper extremity hemiparesis due to ischemic stroke.<br><br>The proposed study is a first in human safety and feasibility study intended to provide preliminary data to design a future pilot study.
**Test Device**	Boston Scientific, Inc. Vercise™ Deep Brain Stimulation System.
**Investigational Treatment**	Unilateral electrical stimulation of the cerebellar dentate nucleus on the side ipsilateral to the hemiparesis (contralesional to stroke).
**Study design**	
**Study Design**	Prospective, open-label, single arm, safety and feasibility study
**Control**	Due to heterogeneity of ischemic stroke, each patient will serve as his or her own control. Comparison will be made across time (e.g. rehabilitation vs. test, and follow-up)
**Investigative Sites**	One site (Cleveland Clinic).
**Number of Subjects**	Up to 12 subjects implanted.
**Patient Population**	Survivors of an initial ischemic stroke between 12 and 36 months post stroke with residual severe unilateral, upper extremity hemiparesis defined as ≤ 42 on the upper extremity subscale of the Fugl-Meyer Assessment.
**Primary Endpoint**	The primary endpoint will be the incidence of all serious adverse events, including Serious Adverse Events (SAEs), Serious Adverse Device Events (SADEs), and Unanticipated (Serious) Adverse Device Events (UADE), from the time of enrollment through follow-up.<br><br>All serious adverse events will be further categorized as procedure-, DBS device-, and stimulation-related.

	All non-serious adverse events will also be tabulated and reported.
**Secondary Endpoints**	• Arm Motor Ability Test (AMAT)<br>• Bilateral Box and Block Test (BBT)<br>• Bimanual Grip strength test<br>• EuroQol (EQ-5D)<br>• Fugl-Meyer Assessment, Upper Extremity (FMA-UE)<br>• 9-Hole Peg Test<br>• Short Form Health Survey (SF-12)<br>• Modified Ashworth
**Other Endpoints**	• Beck Anxiety Inventory (BAI)<br>• Beck Depression Inventory (BDI)<br>• PET<br>• Local Field Potentials<br>• TMS Motor Maps<br>• H-reflex<br>• TMS Cerebellar Brain Inhibition (CBI)
**Statistical Hypothesis**	No formal statistical hypotheses are proposed for this feasibility study.
**Study Phases**	
**Study Phases Summary**	• Baseline<br>• DBS Implant Procedure<br>• Postoperative Recovery (4 weeks)<br>• Rehab Baseline (8 weeks)<br>• DBS Programming (4 to 10 weeks) with rehab continuation<br>• Testing: Stimulation of the dentate nucleus + Rehab (16 weeks)<br>• Rehab Follow-up (4 weeks)<br>• Long Term Follow-up<br>• DBS Explant Procedure<br>• Explant Follow-up (4 weeks)
**Rehabilitation Program**	All patients will undergo an outpatient rehabilitation program after postoperative recovery. The frequency and duration of therapy sessions will be two times per week for 1-1.5 hours of treatment time over a 2-hour scheduled contact interval. In addition to the formal outpatient therapy sessions, participants will sign a behavioral contract that obligates them and their caregivers to continue with their upper extremity rehabilitation program, as specified by the treating therapist, at home.

**Testing (Test Treatment + Rehab)**	Subjects will receive stimulation of the dentate nucleus area and continue their ongoing rehabilitation program (test treatment + rehab) for 16 weeks.
**Rehab Follow-up**	Once the testing phase (test treatment + rehab) has concluded, investigators will gradually wean OFF the device. This will be to assess if gains achieved during the testing phase will persist without continuous stimulation. Loss of more than 50% of improvements achieved during the testing phase will prompt the investigators to turn the devices back ON. Improvements will be indexed with sub-scores (proximal, distal and total limb) of the Fugl-Meyer Scale. Subjects will continue their ongoing rehabilitation program for 4 weeks.
**Long-term Follow-up and Study Completion**	Once the Rehab Follow-up phase is completed, subjects will be followed until their one-year post implant follow-up. There will be no mandatory structured rehabilitation provided by the study and the device will remain OFF until explanted during this phase. Then the device will be explanted as per study procedure.<br><br>Subjects will have a final follow up visit 6-month post explant. At the conclusion of the 6-month post explant follow-up the subject will have completed this protocol.

CONTENTS

1.0 INTRODUCTION 10

1.1 General Overview 10
1.2 Purpose 10
1.3 Duration of the Investigation 11
1.4 Number of Sites and Subjects 11
1.4.1 Staged Implant 11

2.0 DEVICE DESCRIPTION 11

2.1 Name of Device 11
2.2 Vercise DBS System Principle of Operation 13
2.3 Vercise DBS System and TMS System Regulatory Status 13
2.4 Proposed Indications for Use 14
2.5 Manufacturing information 14
2.6 Labeling 14

3.0 BACKGROUND AND SIGNIFICANCE 14

3.1 Overview of Stroke Epidemiology 14
3.2 Acute Management of Stroke 15
3.3 Management of Risk of Recurrent Stroke 15
3.4 Disability and Recovery from Stroke 16
3.5 Rehabilitation of Post-Stroke Upper Extremity Paresis 17
3.6 Treatments for Upper Extremity Paresis 17
3.6.1 Motor Training 17
3.6.2 Non-invasive Neurostimulation Therapies for Post-Stroke Motor Recovery 18
3.6.3 Epidural Cortical Stimulation. 18
3.7 Unmet Medical Need 19
3.8 Deep Brain Stimulation Overview 19
3.9 Clinical Rationale of DBS for Enhancing Post-Stroke Motor Recovery 20

4.0 REPORT OF PRIOR INVESTIGATIONS 22

4.1 Literature Review. 22
4.2 Summary of Preclinical Studies 25
4.3 Conclusion 26

5.0 STUDY DESIGN 27

5.1 Study Objective 27
5.2 Primary Endpoint 27
5.3 Secondary Endpoints 27
5.3.1 Impairment 27
5.3.2 Activity 28
5.3.3 Participation/Quality of Life 29
5.3.4 Other 29

6.0 SUBJECT SELECTION 31

6.1 Study Patient Population 31
6.1.1 General Inclusion Criteria 31
6.1.2 General Exclusion Criteria 32
6.1.3 Transcranial Magnetic Stimulation Subject Inclusion Criterion 33
6.2 Subject Screening 35
6.3 Subject Enrollment 36

7.0 STUDY VISITS 36

7.1 Study Schedule 36
7.1.1 Schedule of Study Visits 38
7.1.2 Schedule of Study Assessments 39
7.2 Baseline and Eligibility Evaluation 39
7.3 Unilateral DBS Electrode Implantation 40
7.3.1 Intraoperative Macroelectrode Testing 42
7.3.2 Local Field Potential Recordings 43
7.3.3 Implantation of the Neurostimulator 45
7.3.4 Hospital Discharge 46
7.4 Postoperative Recovery Period 46
7.5 Rehabilitation 46
7.5.1 Description of the Rehabilitation Program 47
7.6 Device Programming 48
7.6.1 Transcranial Magnetic Stimulation (TMS) 50
7.6.2 Safety of Transcranial Magnetic Stimulation and DBS 51
7.7 Testing phase (stimulation + rehabilitation) 52
7.7.1 Testing phase Continutation and Stoppage Rules 52
7.8 Rehab Follow-up 53
7.9 Long-Term Follow-up: 53
7.10 Explant 54
7.10.1 Explant Procedures 54

7.11 Exit of Participation 54
7.11.1 Loss to Follow-Up 55
7.11.2 Subject Withdrawal from Study 55
7.12 Unscheduled Visits 55
7.13 Protocol Deviations 55
8.0 SAFETY REPORTING 55
8.1 Definitions and Classification 56
8.1.1 Adverse Event (AE) 56
8.1.2 Serious Adverse Event (SAE) 56
8.1.3 Device deficiency 57
8.1.4 Adverse Device Effect (ADE) 57
8.1.5 Serious Adverse Device Effect (SADE) 57
8.1.6 Unanticipated (Serious) Adverse Device Effect (UADE) 57
8.1.7 Relatedness to DBS Device 57
8.2 Device Deficiencies 58
8.3 Deaths 58
9.0 DATA MANAGEMENT 58
9.1 Completion of Case Report Forms 58
9.2 Data Review 59
9.3 Data Analysis Plan 59
10.0 STUDY ADMINISTRATION 59
10.1 Data Monitoring Committee 59
10.1.1 DMC Responsibilities 60
10.1.2 Monitoring for Safety 60
10.1.3 DMC Meetings 60
10.1.4 Making Recommendations 61
10.1.5 DMC Decision Guidelines 61
10.2 Study Registration. 62
11.0 SCIENTIFIC SOUNDNESS 62
12.0 RISK/BENEFIT ANALYSIS 62
12.1 Potential Benefits 62
12.1.1 Chronic Electrical Stimulation of the Dentate Nucleus 62
12.1.2 Advancing Scientific Knowledge and Optimizing Stroke Therapy 63
12.2 Potential Risks of Electrical Stimulation of the Dentate Nucleus 63
12.2.1 Risks Associated with Vercise DBS System Implantation Procedure 63

12.2.2 Risks Associated with Temporary Percutaneous Externalization of DBS Lead..65
12.2.3 Risks Associated with Vercise DBS System......66
12.2.4 Risks Associated with Deep Brain Stimulation Therapy......69

12.3 Risk Management......70

12.3.1 Risk Minimization through Study Design......70
12.3.2 Risk Minimization of DBS Lead Implanted in the Dentate Nucleolus......71
12.3.3 Safety Monitoring and Oversite......72
12.3.4 SARS-CoV-2 Mitigation......72

12.4 Communication of Risks Benefit......73

12.5 Consideration of Patient Preference Information......73

12.6 Assessment of Uncertainty......73

12.7 Risk Benefit Analysis......73

13.0 MONITORING PROCEDURES......74

13.1 Monitoring Procedures......74

13.1.1 Monitoring Reports......74

13.2 Final Site Visit......75

14.0 COMPLIANCE......75

14.1 Statement of Compliance......75

14.2 Device Accountability......75

14.3 Sponsor Responsibilities......75

14.3.1 Sponsor Records......76
14.3.2 Sponsor Reports......76
14.3.3 Sponsor Inspections......77

14.4 Investigator Responsibilities......77

14.4.1 Protection of Human Subjects......77
14.4.2 Investigator Records......77
14.4.3 Investigator Reports......78
14.4.4 Investigative Center Inspections......78

15.0 INFORMED CONSENT MATERIALS......80

16.0 REFERENCES......80

List of Tables

Table 1. Description of Devices 12
Table 2. Schedule of Study Visits 38
Table 3. Schedule of Study Assessments 39
Table 4. Risks of Deep Brain Stimulation Implant Procedure 64
Table 5. Literature Review on Risk of Recurrent Stroke Post Ischemic Stroke 68
Table 6. Investigator and Sponsor Reporting Responsibilities 79

List of Figures

Figure 1: DBS Vercise System 13
Figure 2: Study Timeline 36
Figure 3: Study Flow Diagram 37
Figure 4. Illustration of Patient Position and the Placement of the Head Frame 41
Figure 5. Illustration of Electrode Placement for the Dentate Nucleus Target 42
Figure 6. Final DBS System Placement 46

1.0 INTRODUCTION

General Overview

Stroke is a disease of epidemiological proportions in the industrialized world and a leading cause of long-term disabilities. Approximately 795,000 people in the United States alone suffer strokes every year[1]. While the majority of patients will survive the acute phase, persistent neurological sequelae likely will jeopardize quality of life and productivity, with approximately 50% of survivors still exhibiting some degree of hemiparesis at 6 months after stroke and 30% requiring assistance with activities of daily living.[2] According to the American Heart Association Statistics Committee and Stroke Statistics Subcommittee, the estimated indirect and direct cost of stroke for 2014 is U$ 36.5 billion.[1] These numbers underscore the need for translational research aimed at enhancing motor outcomes after stroke.

The duration and type of rehabilitation provided varies depending on an individual's level of impairment, progress in therapy and other factors such as patient preferences, and insurance coverage for rehabilitation. Deep brain stimulation (DBS) is a treatment that utilizes surgically implanted electrodes and an electrical pulse generator, sometimes called a "brain pacemaker" or neurostimulator, to deliver electrical pulses to specific targeted subcortical brain structures. It is routinely used to manage movement disorders such as Parkinson's disease and essential tremor. In this feasibility study, we propose to evaluate the effects of DBS of the dentate nucleus on motor recovery of chronically hemiparetic patients due to ischemic stroke.

Purpose

The purpose of this feasibility study is to obtain clinical experience and scientific evidence to evaluate the feasibility, safety and effectiveness of electrical stimulation of the dentate nucleus of the cerebellum for the treatment of chronic upper extremity hemiparesis due to ischemic stroke. The results of this study are expected to provide preliminary data on the risks and benefits of the therapy, and adequate information on which to base decisions regarding the need for, and the design and conduct of, future trials of deep brain stimulation (DBS) of the dentate nucleus of the cerebellum for post-stroke upper extremity hemiparesis.

The goals of this feasibility study are to:

- develop preliminary scientific evidence to evaluate the feasibility of DBS therapy;
- calculate estimates of event rates that can be used for sample size determination for future pivotal studies;
- evaluate the success of procedures for recruitment and enrollment of patients for any future pivotal trials;
- learn about the electrophysiological signatures and imaging correlates that may indicate the ideal location for electrode implantation in future clinical trials. (Based on the experience in DBS for movement disorders, these signatures also are expected to streamline the surgical procedure in the future.)

Duration of the Investigation

The estimated duration of this feasibility study is 2 years from the time of first subject enrollment to the last follow-up visit for all patients enrolled.

Number of Sites and Subjects

The study will enroll and implant up to 12 subjects at one investigative surgical site, the Cleveland Clinic (CC). Patients will undergo evaluation, surgery and DBS programming and follow-up at the Cleveland Clinic main campus and hospital. Patients will receive rehabilitation and have follow-up outcome assessments conducted at the Cleveland Clinic.

1.1.1 Staged Implant

The study will initially be conducted with a staged FDA approval process. Initially 2 subjects will be implanted with the DBS System with the limitation that a new subject can be enrolled only after the previous subject has completed 1-month post implantation follow-up.

Safety data will be reported to the FDA, for all subjects enrolled in the study to date, after each subject has completed 1-month post implantation.

After the first 2 subjects have completed one-month post implantation, the number of subjects that can be implanted with the device will be expanded to 12. In addition, the patient pool will be expanded for enrollment of subjects that are moderately to severely impaired as defined by a UE Fugl-Meyer of ≤ 33 and then expanded to ≤ 42. Initially, the first 2 subjects implanted will be more severely impaired as defined by a UE Fugl-Meyer of ≤ 20.

2.0 DEVICE DESCRIPTION

Name of Device

This study will be conducted with the Boston Scientific Vercise family of deep brain stimulation systems, the Vercise™ and Vercise™ PC. The Vercise and Vercise PC DBS systems are approved in the United States (U.S.) by the Food and Drug Administration (FDA) for bilateral stimulation of the subthalamic nucleus (STN) as adjunctive therapy for Parkinson Disease (PD).. The FDA has not approved any Vercise DBS system for this patient population and the method of deep brain stimulation utilized in this study. The Vercise deep brain stimulation systems are Class III implantable devices, and the proposed study is a significant risk study. Therefore, this study requires United States (US) FDA approval of an Investigational Device Exemption (IDE), and local Institutional Review Board (IRB) approval prior to initiating the study.

The Vercisedeep brain stimulation systems are designed to deliver targeted electrical stimulation through implanted electrodes and neurostimulator. The Vercise system components to be used in this study are listed in Table 1.

Table 1. Description of Devices

System	Device Component	Model
Vercise™ Parkinson's Control System	Neurostimulator	Vercise™ Neurostimulator<br>Vercise™ PC Neurostimulator
	DBS Lead	30cm or 45cm<br>Cartesia™ 30cm or 45cm
	Extension	55cm
	Accessories	Lead Boot<br>Lead Stop<br>Torque Wrench<br>Suture Sleeve (1cm, 1cm split, 2.3cm 4cm)<br>Remote Control<br>Charger<br>Vercise™ External Trial Stimulator<br>Vercise™ Clinician Programmer<br>OR Cable and Extension<br>IR Interface<br>IR Interface Holder<br>USB Serial Cable<br>Tunneling tool (shaft and handle)<br>Stimulator Header Plugs<br>Torque Wrench<br>SureTek™ Burr Hole Cover<br>Remote Control 2<br>External Trial Stimulator 2<br>Programming Wand<br>Guide DBS

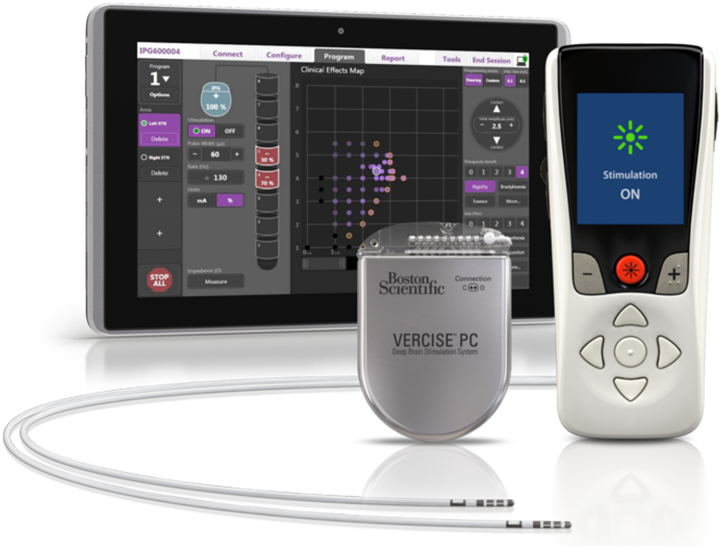

Figure 1: DBS Vercise PC System

Vercise DBS System Principle of Operation

For the specific steps on how to operate the Vercise system, refer to the device labeling, (DBS Vercise Physician Manual and Vercise Burr Hole Cover Manual).

Deep brain stimulation is treatment that utilizes surgically implanted electrodes and a neurostimulator to deliver electrical energy to specific targeted structures deep within either one or both hemispheres of the brain.

For the proposed indication, the aim is to deliver chronic unilateral electrical stimulation to the dentate nucleus of the cerebellum. The hypothesis proposed here (and demonstrated in our pre-clinical studies) is that stimulation of the dentate will chronically activate the dentate-thalamo-cortical pathway and promote rehabilitation beyond the effects of physical therapy alone. Stimulation is expected to enhance cortical excitability and facilitate plasticity, cortical reorganization and, consequently, motor recovery of upper extremity hemiparesis.

Vercise DBS System and TMS System Regulatory Status

In 2012, Boston Scientific Corporation announced receipt of CE Mark approval for use of its Vercise™ Deep Brain Stimulation (DBS) System for the treatment of Parkinson's disease.

The Vercise DBS System has both CE Mark and TGA (Australia Therapeutic Goods Administration) approval for the treatment of Parkinson's disease. It also has CE Mark for intractable primary and secondary dystonia, and is available for sale in Europe, Israel, Australia and select countries in Latin America.

More recently, in 2014, Boston Scientific announced receipt of CE Mark for the Vercise™ Deep Brain Stimulation (DBS) System for the treatment of tremor, including

the most common form of this movement disorder known as essential tremor (ET). In 2015, Boston Scientific Corporation announced receipt of CE Mark approval of the Vercise™ PC DBS System for the treatment of Parkinson's disease, primary and secondary dystonia, and essential tremor.

In the U.S., the Vercise DBS System received FDA PMA approval December 2017 for bilateral stimulation of the subthalamic nucleus (STN) as adjunctive therapy for Parkinson's. The Vercise PC DBS System received FDA approval January 2019 under the same PMA. The use of transcranial magnetic stimulation (TMS) is not approved with DBS and the use of TMS during the study after the DBS system is implanted is investigational.

Magstim Co. Ltd. manufactures the TMS System, consisting of 200^2 Magnetic Stimulators, $Bistim^2$ connecting module, 110mm double-cone coil, and 70mm figure-of-eight coil. The 200^2 and 70mm figure-of-eight coil devices have 510(k) clearances for other intended use. The intended use of TMS used in this protocol is investigational. The 110mm double-cone coil and $Bistim^2$ connecting module are investigational.

Proposed Indications for Use

The Vercise deep brain stimulation system will be used in this proposed feasibility study in the management of chronic, post-stroke, upper extremity hemiparesis in addition to rehabilitation therapy in individuals 12 to 36 months post stroke who have residual moderate to severe upper extremity motor impairment, defined as a Fugl-Myer Upper Extremity subscale score ≤ 42.

Manufacturing information

Manufacturing information for the Vercise Deep Brain Stimulation System is available in Boston Scientific's approved PMA P150031.

Manufacturing information for the Magstim 200^2 Magnetic Stimulator and 70mm figure-of-eight coil is available in Magstim Co. Ltd. cleared 510k (K060847). Manufacturing information for the 110mm double-cone coil and $Bistim^2$ connecting module are available in Magstim Co. Ltd.

Labeling

The Vercise DBS System components and the TMS system to be used in this clinical study will bear a label with the following statement: "CAUTION--Investigational device. Limited by Federal (or United States) law to investigational use."

3.0 BACKGROUND AND SIGNIFICANCE

Overview of Stroke Epidemiology

Cerebral vascular accident or stroke is the neurological injury that occurs because of brain ischemia or hemorrhage. The World Health Organization standard definition of

stroke is a focal (or at times global) neurological impairment of sudden onset and lasting more than 24 hours (or leading to death) and of presumed vascular origin[3].

The incidence of stroke in the United States is estimated to 795,000 of which approximately 185,000 are recurrent strokes[1, 4]. Of all strokes, approximately 85% have an ischemic etiology; intracerebral and subarachnoid hemorrhage account for the remainder. Mortality from stroke is high, and varies with etiology. In persons 45 to 64 years of age, the 30-day mortality rate for ischemic stroke is 8%-12%, and 37%-38% for hemorrhagic stroke[3]. In persons 65 years of age or older the overall 30-day mortality is 12.6%; 8.1% for ischemic strokes and 44.6% for hemorrhagic strokes. Overall, in individuals age 40 years and older, 21% of men and 24% of women are dead one year after a first stroke; 47% of men and 51% of women are dead within 5 years of a first stroke. [5, 6]

Stroke is a leading cause of serious and long-term disability in the United States. The estimated total direct and indirect cost of stroke in the US is $36.5 billion. The mean lifetime cost of an ischemic stroke has been estimated at $140,080 for inpatient care, rehabilitation, and follow-up care for lasting deficits. [1, 4]

Acute Management of Stroke

Today the approach to management of acute stroke is highly proactive. The goal of acute management of stroke is to ensure rapid diagnosis and treatment. Rapid identification and diagnosis of stroke facilitates the administration of the most effective acute stroke therapies including intravenous tissue plasminogen activator (tPA) for ischemic stroke, early management of blood pressure and other neurointensive care measures, surgical and endovascular options when indicated, management of complications of stroke, and institution of appropriate secondary prevention strategies. In ischemic stroke, the emphasis in the acute setting is on maximizing reperfusion in order to minimize tissue injury.[7] There are several clinical trials and emerging technologies aimed at facilitating post-stroke rehabilitation. Timely restoration of blood flow to the structurally intact ischemic areas around the infarct improves the potential for limiting the extent of the injury.

Management of Risk of Recurrent Stroke

Individuals that have experienced an initial ischemic stroke are at high risk for recurrent stroke. The annual risk has been estimated to be anywhere from 3% to 22% with an average of about 15%. Reports of rates of recurrent ischemic stroke vary due to methodological differences in analysis, as well as differences in age, gender, coexistent morbidities and risk factors, and background medical treatments among the cohorts studied. The American Heart Association reports 5-year recurrence rates for individuals that survive a first stroke (of any type) as a function of age, gender and race. These 5-year recurrence rates range from a low of 10% in black men aged 40 to 69 years to a high of 32% for black women over the age of 70 years.

Disability and Recovery from Stroke

Recovery after stroke displays a nonlinear pattern with the largest improvements occurring within the first three months and improvement gradually stabilizing thereafter[8, 9]. The course of recovery from stroke varies according to the type and severity of the stroke, patient characteristics, and treatment regimen. A majority of stroke survivors, from 50% to 70%, achieve functional independence. However, 15% to 30% of stroke survivors are permanently disabled, and 20% require institutional care at three months post onset.[10, 11]

Up to 85% of stroke survivors experience some degree of upper extremity hemiparesis, and only 25% to 55% of these individuals achieve functional recovery of their arm.[10, 11]

Rehabilitation involves a comprehensive, multidisciplinary effort to utilize medical, therapy, social, vocational, and educational techniques to assist the stroke survivor to achieve maximal recovery. Rehabilitation includes strategies to optimize recovery of neurologic impairment, to teach compensatory strategies and skills for activities of daily living and independent living, and to provide psychosocial support to the patient and their families.

Patients recover after stroke in two ways. First, patients recover through spontaneous recovery of neurological function, through treatments that limit the extent of damage from the stroke, and through other interventions that enhance neurological functioning. Secondly, patients are able to adapt and learn to accomplish their daily activities within the limitations of their neurological impairments.

Rehabilitation typically includes some combination of physical therapy, occupational therapy, speech therapy, family education, and home therapy programs. Therapy may be provided in the acute, sub-acute or outpatient settings.

Rehabilitation techniques directed at reducing motor impairments are based on the concepts of neuroplasticity and reorganization of cerebral activity. While the primary motor cortex is well defined anatomically, there are secondary motor areas that originate motor neural pathways. Improvements in motor function can be promoted by promoting cortical reorganization of the primary motor representation as well as vicariation of function from secondary motor areas including the premotor cortex[12]. Modifications in neural networks are use dependent, and active participation by patients with motor impairments in therapy programs directly influences the process of functional reorganization in the brain to enhance neurological recovery.[12, 13]

The increased availability of advanced neuroimaging techniques such as positron emission tomography (PET) and function magnetic resonance imaging (fMRI) in the last decade has made human research on the mechanisms and biology of motor recovery after stroke possible. New techniques for stroke rehabilitation are emerging that take advantage of advances in understanding of the mechanisms of stroke recovery and the concept of brain plasticity.

Rehabilitation of Post-Stroke Upper Extremity Paresis

Current practice guidelines for stroke rehabilitation recommend early initiation of rehabilitation once a patient is medically stable, and that patients receive as much therapy "as needed" to recover motor function. Rehabilitation care often requires a multidisciplinary team of experienced professionals. This may include physical medicine, nursing, physical therapy, occupational therapy, kinesiology, speech-language therapy, psychology, and recreational therapy.[14 15]

Rehabilitation for post-stroke upper extremity paresis includes both compensatory and restorative approaches. Compensatory approaches focus on training the individual to utilize alternative strategies and tactics to improve motor function and ability to carry out daily activities. Restorative therapies focus on motor retraining and recruiting surviving areas of the brain to perform the functions of the damaged areas.

There are a variety of rehabilitation techniques available, including neurodevelopmental therapy, sensory motor integration, proprioceptive neuromuscular facilitation, constraint-induced movement therapy, mental imagery and biofeedback. Rehabilitation programs are usually individualized for the patient based on their presenting deficits and medical status. The patient's tolerance for therapy depend on multiple factors including the severity of the stroke, medical stability, cognitive status, communication ability, level of function, and the extent of social support.

In this study, the investigation will not evaluate early rehabilitation strategies for strokes. Instead, we will re-initiate rehabilitation intervention after dentate nucleus DBS implantation in a fashion similar to strategies applied in the post-stroke, chronic outpatient rehabilitation phase. It is the investigator's expectation that chronic dentate nucleus electrical stimulation will open a new window for effectiveness from rehabilitation training in the chronic post-stroke phase.

Treatments for Upper Extremity Paresis

There are multiple treatment strategies for post-stroke rehabilitation. These include motor training and non-invasive stimulation. Invasive stimulation with epidural stimulation, as discussed below, has been attempted, but has not achieved the desired outcomes to date.

3.1.1 Motor Training

There are several motor training techniques, including task-specific training[16], neurodevelopmental therapy[17], and constraint induced movement therapy (CIMT)[18]. Accumulating evidence over the past decade indicates that task specific training oriented toward repetition with or without shaping can lead to significant improvement in functionally based upper extremity movement capability following stroke[19]. Constraint-induced movement therapy (or CIMT) is a unique form of upper extremity "forced use" therapy and truly represents a subset of functional training. CIMT uses repetitive task practice or adaptive task practice through intense training. This modality has been evaluated in the multi-site EXtremity Constraint Induced Therapy Evaluation

(EXCITE) Trial. The study showed that CIMT resulted in sustained benefits in patients who had suffered strokes 3 to 9 months prior[20].

3.1.2 Non-invasive Neurostimulation Therapies for Post-Stroke Motor Recovery

The use of electrical stimulation to facilitate post-stroke recovery seems intuitive. The goals can be defined as boosting neural function of the perilesional and other spared areas of the nervous system in order to promote plasticity and neurorehabilitation. Techniques for non-invasive stimulation include functional electrical stimulation (FES), transcranial magnetic stimulation (TMS) and transcranial direct current stimulation (tDCS). Electrostimulation delivered to the peripheral neuromuscular system to improve movement and function following a stroke is an option for the treatment of upper extremity hemiparesis that is under study. A Cochrane meta-analysis that reviewed the data from existing trials concluded that while the current data suggests some benefit from electrical stimulation, the current data is insufficient to conclude that the therapy is effective[21]. Currently these therapies are not broadly utilized and remain under research. Repetitive transcranial magnetic stimulation (rTMS) has been under evaluation for several years. Clinical studies published to date involve small numbers of patients, and apply this therapy for the treatment of motor impairment from subcortical strokes[22, 23]. There is uncertainty regarding patient selection and the most appropriate stimulation parameters as well as site of stimulation for clinical application. For example, the optimal frequency for stimulation remains a matter of debate as well as whether stimulation is best applied to the perilesional motor cortex or to secondary cortical areas. Furthermore, contralateral stimulation remains an option but it is unclear if it should be aimed at a particular subpopulation of patients. We have recently reviewed the use, limitations and alternative approaches for TMS and tDCS[12].

3.1.3 Epidural Cortical Stimulation.

Chronic epidural cortical stimulation can be applied in humans as well as in animal models. There are no currently available commercial systems for chronic cortical stimulation in humans. However, clinicians often make off-label use of spinal cord stimulation hardware to implant epidural leads over the motor cortex and connect to implanted pulse generators. The most common utilization of cortical stimulation in the U.S. and Europe is for pain management, following the initial publication by Tsubokawa and collaborators[24]. However, outcomes are limited and reviews have shown that the method is less efficacious than initially thought, particularly for post-stroke pain[25]. Motor cortex stimulation has also been hypothesized to promote perilesional plasticity and to facilitate post-stroke recovery. The approach once held great promise for promoting motor rehabilitation following stroke based on preclinical work in animal models[26-29]. Early phase clinical trials also showed promising [30, 31]; however a large randomized clinical trial failed to meet its intended endpoints[32]. The reason for the lack of success of the phase III trial is not completely clear, though methodological issues may have contributed to the negative results.

It is possible that chronic epidural cortical stimulation is not a viable option for post-stroke motor rehabilitation in humans. The cerebral cortices of the rodent and some non-human primate models (i.e., squirrel monkey) are relatively flat in comparison to

the profound convolutions found in the human brain, resulting in a more consistent pattern of organization of the pyramidal and inter-neurons across large expanses of cortex. In humans, the orientation of neurons in relation to the dura can vary dramatically depending on their location relative to the crown or sulcus of a gyri. Cathodal and anodal stimulation have different effects on the cortical surface and the orientation of the neuron plays a key role in determining its response to epidural stimulation.[33, 34] Hence, it is conceivable that in human clinical trials the effects of epidural stimulation had a net neutral effect or negative effect, or perhaps simply did not achieve a necessary volume of effect, due to this variation in the neuron orientation relative to the epidural stimulating electrodes. One of the potential benefits of the approach proposed herein lies in taking advantage of the dentatothalamocortical pathway fibers directing the effects of stimulation to the perilesional cortical area, regardless of the orientation of neurons in the corresponding gyri.

Unmet Medical Need

Upper extremity hemiparesis initially is seen in up to 85% of patients following a stroke and by 6 months post stroke between 45% and 75% of patients continue to have some degree of upper extremity functional impairment, and as many as 30% to 60% of patients are unable to use their affected arm functionally after discharge from rehabilitation.

In a study of 102 severely impaired acute stroke survivors with almost total upper extremity paresis (mean Upper Extremity Fugal-Myer score of 7 out of a possible 66 points), only 38% showed some recovery of dexterity in the hemiplegic arm and only 4.4% achieved complete functional recovery at six months post stroke. All patients had experienced ischemic strokes in the region of the middle cerebral artery (MCA), and had participated in a randomized trial of acute rehabilitation approaches involving 5 hours of therapy a week for 20 weeks[35].

Although extensive research is under way on acute interventional therapies aimed at tissue protection and reduction of infarct volume, relatively few efforts have been made at enhancing plasticity and recovery of function in the chronic phase after strokes. The prevalence of stroke survivors in the United States is greater than five million individuals, highlighting the importance of investigation on emerging therapies aimed at alleviating the burden of permanent hemiparesis on individuals, family and society. There is immense need for novel therapies to be tested given the limited benefits observed to-date with existing technologies aimed at promoting post-stroke rehabilitation.

Deep Brain Stimulation Overview

Deep brain stimulation (DBS) is treatment that utilizes surgically implanted electrodes and a neurostimulator to deliver electrical energy to specific targeted structures deep within either one or both hemispheres of the brain.

Neurologists and neurosurgeons have used microelectrode recording and stimulation since the 1960s to locate and map specific subcortical structures in the basal ganglia,

diencephalon and cerebellum. Microelectrode recording has also been extensively performed in the dentate nucleus region that will be targeted in this study.

Early experiences with subcortical electrical stimulation were acquired during stereotactic lesioning procedures such as pallidotomies and thalamotomies. In these procedures, surgeons used depth probes to generate heat by radiofrequency to ablate structures thought to be involved in the pathology of movement disorders such as essential tremor. During thalamotomies for tremor, neurosurgeons would electrically stimulate the thalamic nucleus prior to ablation. The purpose was to observe if electrical stimulation would influence tremors in the awake patient prior to making the permanent, non-reversible lesions (the "thalamotomy"). This experience showed pioneers in the field that electrical stimulation at high frequency could control tremors for the short period of time during which stimulation was tested. This indicated that, possibly, chronic electrical stimulation could chronically modulate tremors or other symptoms of movement disorders. The first report of long-term chronic electrical stimulation with DBS therapy for the treatment of tremor was published in 1991 by Drs Benebid and Pollak of the University of Grenoble in France[36]. The first FDA approved use (1997) of DBS was for the treatment of essential tremor or tremor due to Parkinson's disease. Since then DBS has been considered for the treatment of a variety of neurological disorders, and Medtronic's DBS Therapy has been approved for use in the United States by the FDA for:

- Unilateral thalamic stimulation of the ventral intermediate nucleus of the thalamus (VIM) for tremor,
- Bilateral stimulation of the internal globus pallidus (GPi) or the subthalamic nucleus (STN) for Parkinson's Disease
- Unilateral or bilateral stimulation of the internal globus pallidus (GPi) or the subthalamic nucleus (STN) for dystonia, under a Humanitarian Device Exemption.
- Bilateral stimulation of the anterior limb of the internal capsule, AIC, for treatment of chronic, severe, treatment-resistant obsessive compulsive disorder (OCD), under a Humanitarian Device Exemption.

The literature provides a number of additional reports of clinical experience with DBS for treatment of disorders including rare movement disorders, epilepsy, pain, Tourette's syndrome, and Alzheimer's disease.[37-44 45-47]

Clinical Rationale of DBS for Enhancing Post-Stroke Motor Recovery

The proposed IDE feasibility study is designed to evaluate a novel use of DBS for enhancing motor recovery in patients with chronic, upper extremity weakness following an ischemic stroke. The brain target selected for electrical stimulation is the dentate nucleus of the cerebellum. The clinical rationale for the use of DBS and specifically the selection of the dentate nucleus as the stimulation target is based on an understanding of the anatomy and physiology of the brain, and the neurophysiology of motor function.

The cerebral cortex is tightly and reciprocally linked to the cerebellum, with the majority of the fibers decussating at the level of the brainstem to connect with the contralateral hemisphere. The bulk of the projections from the cerebral cortex to the

cerebellum is carried by the corticopontocerebellar pathway, with a synapse at the pontine nuclei in the brainstem. This pathway reaches the cerebellum through the middle cerebellar peduncle and its interruption is believed to cause massive deafferentation of the cerebellum. This lack of afferent input is believed to be the cause of decreased metabolism and activity that represent the hallmark of crossed cerebellar diaschisis (CCD)[48-51], as reflected in imaging studies such as positron emission tomography (PET)[52, 53] and single photon emission tomography (SPECT)[54-58]. The corticopontine system is the corticofugal part of this two-step pathway. The projections from the motor cortex reach the pons with the crossover to the contralateral cerebellar hemisphere occurring after this connection6.

The dentatothalamocortical (DTC) pathway, the ascending part of cerebro-cerebellar interactions, is the main connection from the cerebellar hemispheres to the cerebral cortex. The fibers exiting the dentate decussate at the level of the inferior colliculus and surround the contralateral red nucleus (where a minority of the fibers terminates). The majority of the fibers terminate in the ventroposterolateral pars oralis (VPLo), mediodorsal and ventrolateral pars caudalis (VLc) nuclei of the thalamus. These thalamic nuclei project, in a somatotopically organized fashion, to the primary motor cortex as well as premotor and parietal regions. The dentatothalamic projections were once thought to project only to the primary motor cortex. However, more recent elegant studies using virus retrograde labeling in the primate brain demonstrated that the projections extend to the premotor and parietal regions[59-61]. This is important for our research because stimulation carried by the dentatothalamocortical pathway would not likely produce much benefit if carried only to the motor cortex, which is expected to be injured by the MCA infarct that produces hemiparesis. Because the dentatothalamocortical pathway in fact projects to prefrontal and parietal areas, it can therefore carry stimulation to areas that are potentially preserved after MCA infarction.

At the pontocerebellar level, the projections from the pontine nuclei to the cerebellum are mossy fibers, with excitatory input to the granule fibers (and consequently to the Purkinje cells). There are also excitatory collateral branches to the deep cerebellar nuclei. The output of the cerebellar cortex to the deep nuclei is inhibitory, mediated by the Purkinje cells. The dentate nucleus, however, maintains an excitatory discharge pattern. This highly active output may be driven by extra-cerebellar excitatory input to the nucleus that overcomes the inhibitory input from the cerebellar cortex. In the scenario of disruption of the corticopontocerebellar pathway from a hemispheric stroke, part of the excitatory input to the cerebellar hemisphere will be damaged, accounting for the reduction of metabolism that is translated as CCD. The dentate nucleus also loses the excitatory input from the collateral contribution. The reduced excitatory output from the dentate nucleus has been linked to worsened motor outcome seen in patients with CCD[62]. A central part of the rationale for our hypothesis is based on CCD and its negative effect on motor outcomes. If reduced cerebello-cerebral output results in poor motor outcomes, it is possible that enhancing the cerebello-cerebro output (or normalizing it) may enhance motor outcomes.

The dentate nucleus was selected as the primary target in this study in order to activate the output of the DTC pathway and, consequently, enhance cortical excitability in individuals recovering from ischemic strokes. An alternative target for this therapeutic

approach would be the motor thalamus. However, in order to encompass the entirety of the influence of the DTC pathway, at least three thalamic nuclei would have to be stimulated simultaneously (MD, VPLo and VLc). Additionally, our approach aimed at the dentate may allow us to influence cortical excitability not only directly through the DTC pathway but also indirectly through enhancement of intrinsic thalamocortical rhythms. It is possible that focal thalamic stimulation alone could result in a more direct, “meaningless” stimulation of the cortex. Furthermore, the concentration of DTC pathway fibers at the dentate nucleus output region, projecting to the motor, premotor and parietal cortices, makes this target an anatomically advantageous node for targeting with a single DBS electrode, thus reducing risk associated with surgical intervention. For a detailed review on the rationale for stimulating the cerebellothalamocortical pathway to promote motor recovery please refer to Machado and Baker, 2012[63].

4.0 REPORT OF PRIOR INVESTIGATIONS

Clinical investigation of DBS in the cerebellar dentate nucleus for the treatment of post-stroke upper extremity hemiparesis is in the feasibility stage of development. We have conducted several preclinical studies to assess the efficacy of this emerging therapy as well as possible mechanisms underlying the motor improvements that have been observed.

In addition, a thorough literature review was conducted to learn of prior studies reporting safety data related to stereotactic targeting the cerebellar nuclei and posterior fossa approaches. This review provides preliminary evidence for the safety of the proposed deep brain stimulation therapy.

Literature Review.

Human stereotactic neurosurgery is a broad field within neurosurgery, dating back to the late 1940’s when Spiegel and Wycis pioneered the first apparatus for stereotactic neurosurgery in humans[64-67]. For decades, the most common stereotactic procedures involved insertion of probes or electrodes for lesioning selected brain targets. Most commonly, the purpose was to ablate a brain nucleus involved in the pathophysiology of a movement disorder such as essential tremor or Parkinson’s disease. A few neurosurgeons were key to the development of pallidotomies and thalamotomies, procedures that were popularized in the 1960’s and 1970’s and are still used today. Among many, the works of Hassler and Dieckman[68-75], Leksell and Laitinen,[71, 76, 77] Talairach[78, 79] can be highlighted.

In the 1980’s, chronic subcortical stimulation (deep brain stimulation) was pioneered by Benabid and colleagues in Grenoble, France[36, 80]. During the past two decades, deep brain stimulation gradually replaced stereotactic lesioning procedures as the method of choice for the surgical treatment of movement disorders. The reason for this technical migration was not only the exploration of new cerebral targets such as the subthalamic nucleus but also, safety. Direct comparisons have been made between thalamotomies and deep brain stimulation of the ventral intermedial nucleus (VIM) for the treatment of tremor. Although the efficacy is similar, the rate of complications with deep brain stimulation is significantly lower, as demonstrated by Tasker in 1997[81]. A 2001 study

compared a historical series of stereotactic thalamotomies to a cohort of patients with VIM DBS[82]. Although the effects in tremor suppression were very similar between both groups, complications were more common among patients with thalamotomies, particularly intracerebral hemorrhages (35% vs. 0%). Likewise, cognitive deterioration and hemiparesis occurred, respectively, in 29% and 12% of patients who had undergone thalamotomies, but in none of those with thalamic stimulation. For this feasibility study IDE proposal, the importance of Tasker's and Pahwa's studies is to indicate that, although ablative stereotactic procedures such as thalamotomies can be considered safe, stimulation is likely safer.

The surgical target for the present study is the region of the cerebellar dentate nucleus. Although there is only limited literature documenting the safety of implantation of deep brain stimulation electrodes in this region, a large body of evidence exists for the safety of ablative procedures of the dentate nucleus (dentatotomy).

Surgical exploration of the dentate nucleus of the cerebellum in humans was pioneered and developed by Siegfried, as early as 1968.[83] The purpose of these surgical procedures was not to improve motor function (as is the case in the present study) but, rather, to alleviate spasticity and hyperkinetic disorders. A total of 72 dentatotomies for spasticity were analysed. No neurological morbidity to dentatotomy was observed "as long as the lesion does not extend to the medial nuclei." [83, 84] Although mistargetting is a small but present risk in stereotactic neurosurgery, a permanent lesion causes a non-reversible effect – as probably seen by Siegfried in lesions that were too medial. Deep brain stimulation is reversible – an adverse effect related to stimulation, when noted, can be reversed. In addition to establishing outcomes, Siegfried's experience also provides valuable knowledge of the human functional anatomy.

These studies report the safety and feasibility of performing radiofrequency intentional ablations of the dentate. Much like the experience with deep brain stimulation and ablation for the thalamus for movement disorders, it is expected that deep brain stimulation may be a safer method of targeting the cerebellar nucleus than ablative procedures. The analogy can also be traced in surgery for psychiatric disorders where DBS of the ventral striatum and anterior limb of the internal capsule (now approved under a Humanitarian Device Exemption) was found to be as safe or safer than lesions performed by radiofrequency ablation or radiosurgery in the anterior limb of the internal capsule.

Functional explorations of the dentate nucleus in humans were also performed and elegantly reported by Slaughter and Nashold[85-88]. These authors evaluated the deep cerebellar structures with microelectrodes and macroelectrodes, demonstrating the feasibility and safety of recording from this region in patients.

Chronic electrical stimulation of the dentate nucleus was first reported by Schvarcz in 1980.[89] The initial experience in four patients demonstrated good efficacy in improving spasticity (as previously seen with dentatotomies), with no complications reported. In Schvarcz et al 1980(6), the authors report on one patient with cerebral palsy who was implanted via a posterior fossa approach (radiography indicates an approach similar to the approach in this study) with the patient in the sitting position. The report does not indicate the exact model of hardware other than that it was a "platinum multipolar

electrode array" by Medtronic. The authors indicate that "acute stimulation produced a noticeable improvement (in spasticity) and the patient was percutaneously stimulated for 10 weeks. Pre- and postoperative neurological, psychiatric and speech examinations were performed independently. Posture, balance and spasticity were much improved. Speech was improved. Psychological testing showed "improved psychological status. Higher intellectual performances were improvement, alertness and concentration abilities were increased and verbal output was significantly enhanced"

These findings were later corroborated by the same group, in a larger series of 22 patients.[90-92] In the study by Schvarcz et al, 1982, stimulation was initially tested for 8-12 weeks with an externalized lead and the system was then internalized. The report does not specify the surgical approach other than to indicate that the patients were operated in the sitting position. We have assumed that as for their 1980 study the approach was also through the posterior fossa although a radiograph is not shown in the second paper. The report does not indicate the exact model of hardware that was utilized other than to indicate that the device was from Medtronic. Clinical benefits were measured by electrophysiological measures (EMG) but long-term clinical outcomes are not reported.

The use of modern deep brain stimulation hardware in deep cerebellar structures was reported in 2003 by Galanda and Horvarth. A series of 3 patients showed that stimulation was effective in alleviating spasticity. No severe complications were reported. The authors noted reversible improvements in mood associated with stimulation.[93] In Galanda and Horvath 2003(8), the authors report on three patients who underwent deep cerebellar stimulation for 3, 8 and 29 months. The surgical approach was via the posterior fossa (radiography shows an approach that is equivalent to the present study) with a stereotactic headframe. The patients were implanted with Medtronic 3387 lead models and then internalized with a Soletra Model, Medtronic. Monopolar stimulation was tested at 100 Hz, 500 microseconds and up to 4V. The authors increase stimulation until postural changes were aggravated and then reduced the amplitude, resulting in a transient pleasurable sensation and reduction in spasticity. Chronic stimulation with the internalized system was set to 185Hz, 210 microseconds and 0.5-2.5V. The authors report no complications and report improvements in mood and posture, spasticity. Activities of daily living were improved.

A follow-up study reported on one additional patient and the long term effects of deep cerebellar stimulation in the previously implanted individuals[94]. Chronic implantation of electrodes was accomplished via a suboccipital (posterior fossa) approach, as planned in this feasibility study. The report by the same authors in 2007(9) is similar. The surgery was also done via a suboccipital approach and aimed at the deep cerebellar nuclei and anterior lobe region. The patients were implanted with Medtronic leads model 3387 or 3389 and Medtronic pulse generators were also by Medtronic model Soletra or Kinetra. Patients were implanted either unilaterally or bilaterally. The surgical approach was the same and the amplitudes and settings were the same as the prior publication. The authors concluded that "chronic stimulation of the anterior lobe of the cerebellum seems to be an effective and safe treatment for patients with cerebral palsy".

Summary of Preclinical Studies

The Machado laboratory at the Lerner Research Institute has conducted several studies in animal models of stroke to assess the effects of chronic deep cerebellar stimulation on motor recovery as well as possible neurophysiological mechanisms underlying the observed effects.

There are extensively validated models of stroke in the rodent model, including behavioral measures. The rodent stroke model have been well characterized [95-100] for producing predictable and stable motor deficits and the methods for measuring motor function - including skilled reaching - in the rodent model have been shown to be reproducible, reliable and to be sensitive to the effects of treatment [95-109]. The preclinical data presented stem from experimentation that utilized validated methods for both inducing a stroke [95 97-102 110] as well as validated measures for measuring motor outcome after stroke [99 104, 105 107-109 111, 112]. All techniques utilized for assessing the mechanisms underlying the observed effects have also been reported in the neuroscience literature.

The dentate nucleus anatomy, physiology and projections have been very well characterized in the rodent model and correlate strongly with the anatomy, physiology and projections in the primate [59, 113-118]. In other words, it is possible to produce reliable changes in the activity and excitability of the contralateral hemisphere in the rodent model in a fashion similar to that already demonstrated in the non-human primate model. Dr. Rispal-padel studied the baboon models and demonstrated that stimulation of the dentate nucleus produced activation of the contralateral cerebral hemisphere [114 119 120]. We have reproduced and expanded on these findings in the rodent model. We demonstrated that stimulation of the lateral cerebellar (dentate) nucleus can produce strong activation of the contralateral hemisphere, thus establishing the functional correlation between the cerebello-thalamo-cortical pathway of the primate and the rodent [121, 122]. We have further studied the rodent electrophysiology and relevance of the dentato-thalamocortical pathway to show that the effects of stimulation on cortical excitability are frequency-dependent and that stimulation can sustainably enhance cortical excitability over time. These findings are strong corroboration to the presence and physiological relevance for this pathway in the rodent as it is for the human and primate.

Our first study in the rodent model of ischemia was published in 2009[38]. The study assessed the effects of chronic stimulation of the lateral cerebellar nucleus (LCN), the equivalent of the dentate nucleus in primates, in Wistar rats that had suffered and survived large middle cerebral artery infarctions. The goal of these studies is to evaluate if chronic stimulation can promote recovery after well-established strokes rather than attempt to reduce the size of impact of the initial injury. For this reason, animals that survive the initial injury are allowed to recover for two weeks before stimulation is initiated. In this first proof of principle study we assessed the effects of chronic stimulation at 20 Hz, 50 Hz or 100 Hz compared to sham (electrode implanted but stimulation not activated). All animals underwent training in a motor task (the Montoya Staircase Task) prior to stroke and then underwent daily training in the same task during

the stimulation period. While animals receiving 100 Hz chronic stimulation of the LCN showed no signs of improved recovery compared to SHAM, animals receiving stimulation at 50 Hz showed a trend for better outcomes. However, the best results were observed with stimulation at 20 Hz, which was associated with significantly better recovery than training alone (sham). The results not only pointed towards a first demonstration of viability for this approach, it also pointed that the results of stimulation were clearly frequency-dependent.

Because the frequency-dependent effects of stimulation of the DTC were poorly characterized in the literature, we conducted experiments specifically aimed at addressing the effects of LCN stimulation on cortical excitability, indexed by the magnitude of intracortical microstimulation motor evoked potentials[121, 122]. Animals implanted with deep cerebellar leads in the LCN underwent continuous 10-min blocks of stimulation at various frequencies separated by 10-min blocks of no stimulation. The following frequencies were tested in a pseudorandom order: 10, 20, 30, 40, 50 and 100 Hz. While the net effects of LCN stimulation at 100 Hz were net inhibitory (reduced the amplitude of motor evoked potentials) frequencies of 50 Hz and below had an excitatory effect. Specifically, stimulation at 30 Hz produced the greatest and most sustained effects on cortical excitability[121].

The next step of experimentation was to test the long-term effects of 30 Hz stimulation on long-term motor outcomes. The purpose of this experimentation was three-fold: a) to assess the reproducibility of effects of chronic LCN stimulation on post-stroke motor recovery; b) assess the effects of stimulation specifically set at 30 Hz, given that it produced the most robust and sustained effects on cortical excitability; c) evaluate the synergistic effects of chronic stimulation with motor training. In prior experiments, all animals underwent both motor training and stimulation (or sham) but stimulation had not been delivered *during* motor training. This study[123] showed that a) the results were reproducible and animals receiving stimulation recovered to a significantly greater extent than animals undergoing motor training alone; b) 30 Hz stimulation was associated with excellent recovery of motor function, animals returned to the pre-stroke baseline in the motor task and c) stimulation was well tolerated during motor training.

Given the consistency of the behavioral improvements associated with chronic stimulation of the LCN, our group then dedicated its efforts to assess the mechanisms underlying the effects of stimulation. In this recent work[124] it was found that a) chronic LCN stimulation enhances motor recovery, further reproducing the prior results once again; b) that chronic LCN stimulation promotes the expression of markers of long-term potentiation in the perilesional cortex compared to sham; c) that recovery and expression of such markers are associated with repairing of motor representation of the proximal and distal forepaw in the perilesional cortex and d) that chronic stimulation is associated with synaptogenesis.

Conclusion

Together, the preclinical studies indicate that chronic stimulation has reproducible effects in promoting motor recovery after cortical ischemia in the rodent model. Furthermore, it suggests that the mechanisms underlying behavioral improvements are

likely involve electrophysiological facilitation with enhancement of cortical excitability as well as promotion of perilesional reorganization and cortical plasticity.

5.0 STUDY DESIGN

Study Objective

This is a prospective, open-label, non-randomized, first in human, early feasibility study. The objective of this study is to obtain preliminary data on the safety and feasibility of deep brain stimulation in the dentate nucleus area of the cerebellum when used to treat patients with chronic, moderate to severe, upper extremity hemiparesis due to ischemic stroke.

Primary Endpoint

The primary endpoint will be the incidence of all serious adverse events, including Serious Adverse Events (SAEs), Serious Adverse Device Events (SADEs), and Unanticipated (Serious) Adverse Device Events (UADE), from the time of enrollment through follow-up. The incidence of all UADE rates will be categorized according to procedure, device, and stimulation.

All non-serious adverse events will also be tabulated and reported.

Secondary Endpoints

To characterize how the investigative treatment (DBS + Rehab) effects patients' upper extremity motor function and quality of life, a variety of tests will be performed at baseline and repeated at selected time points during the study.

The assessments will evaluate impairments, activity limitations and participation restrictions which are World Health Organization's (WHO) framework for measuring health and disability based on the International Classification of Functioning, Disability and Health (IFC).

5.1.1 Impairment

Fugl-Meyer Assessment, Upper Extremity (FMA-UE)

The FMA-UE is a disease specific impairment index designed specifically for assessment of recovery from post-stroke hemiplegia. The scale has 33 items assessing upper extremity impairment. Each item is scored as 0 = cannot perform, 1 = performs partially, or 2 = performs fully. A total score of 66 points indicates normal upper extremity motor function, and a score of 0 represents complete motor impairment.

The FMA-UE is a highly-recommended instrument for stroke assessment by the Neurology Section of the American Physical Therapy Association's Stroke Taskforce (StrokEDGE) Reliability. Validity has been assessed[125-129] and norms for stoke are available[130].

9-Hole Peg Test

The bilateral 9 Hole Peg Test (9-HPT) is a simple, timed test of fine motor coordination. Reliability and validity have been assessed[131] and norms for stoke are available[132]. The test involves the subject placing 9 dowels (9 mm in diameter and 32 mm long) in 9 holes. Subjects are scored on the amount of time it takes to place and remove all nine pegs. Two scores are collected, one for each hand. An iPad Based disability assessment tool will be used to determine the exact time of insertion and removal of each pin and the total time to complete one cycle[133].

Bilateral Box and Block Test

The Box and Block Test (BBT) is a standard assessment tool used by occupational therapists to assess gross manual dexterity. The score on the test represents the number of blocks that can be transported from one compartment of a box to another within one minute, and requires that the participant extend the wrist to both retrieve and place the blocks from one box to another. BBT has demonstrated excellent test-retest reliability for acute and chronic stroke[131] plus excellent interrater reliability for upper extremity paresis[125].

Bimanual Grip Test

Kinetic features of manual dexterity and fine motor control during a task that resembles an activity of daily living will assess level of recovery in bimanual function. A two-transducer system will be used to evaluate a realistic and frequently performed bimanual task, that is, opening and closing of a jar using different configurations of the hands to open the lid and stabilize the jar[7, 134-136].

Modified Ashworth

The Modified Ashworth Scale (MAS) is a clinical scale used to assess muscle spasticity. The Scale evaluates the spasticity of individual joints on a range from 0-4 (including 1+) with a score of 0 indicating no increase in tone and 5 indicating rigidity. Evidence indicates that the MAS is a reliable assessment for upper limb and distal muscle groups[177]. Within a patient population who have had a stroke, it has demonstrated excellent test-retest reliability for elbow spasticity[178] and convergencey with the Fugl-Myer, Box-Block and grip strength making it a congruent test for this study[179,180].

5.1.2 Activity

Arm Motor Ability Test (AMAT)

The AMAT is designed to evaluate qualitative and quantitative aspects of an individual's ability to use their upper extremity in the execution of activities of daily living. The AMAT includes 13 ADL activities involving one to three component tasks or movement segments. Each of tasks is timed and rated according based on the extent to which the impaired extremity was used to complete the task (aka functional ability), and the quality of the movement. AMAT is a recommended instrument for stroke assessment by the Neurology Section of the American Physical Therapy Association's

Stroke Taskforce (StrokEDGE) with excellent test-retest reliability and interrater reliability[137] and concurrent validity with the Fugl-Meyer Assessment[138].

5.1.3 Participation/Quality of Life

Short Form Health Survey (SF-12)

The Short Form Health Survey is a patient-reported survey of patient health. Scores from the 12 questions result in two scales of mental and physical functioning and overall health-related quality of life. For stoke norms have been established[139] and reliability[140] and consistency[139] validity[141]. Short form Health Survey is a recommended instrument for stroke assessment by the Neurology Section of the American Physical Therapy Association’s Stroke Taskforce (StrokEDGE).

EuroQol (EQ-5D)

The EuroQol (EQ-5D) is a standardized instrument for use as a measure of health outcome. The EQ-5D captures health-related quality of life states consisting mobility, self-care, usual activities, pain/discomfort, anxiety/depression. EQ-5D has been assessed from stoke[142].

5.1.4 *Other*

Beck Anxiety Inventory (BAI)

The Beck Anxiety Inventory (BAI) is a 21-question multiple-choice self-report inventory that is used for measuring the severity of an individual's anxiety.

Beck Depression Inventory (BDI)

The Beck Depression Inventory (BDI) is a 21-question multiple-choice self-report inventory, one of the most widely used instruments for measuring the severity of depression. Normative data and internal consistency has been established for the BDI for both Acute[143, 144] and Chronic Stroke[143].

Positron Emission Tomography (PET)

PET studies will be used as an exploratory measure to assess possible mechanisms of post-stroke motor function recovery and possible mechanisms underlying the effects of deep brain stimulation of the cerebellar dentate nucleus. PET will be used to add to the knowledge of the physiology of the dentatothalamocortical pathway and on the mechanisms of deep brain stimulation of the dentate nucleus.

Although this part of the investigation is not expected to provide direct beneficence to each patient, we anticipate that it may provide important information regarding the neural networks that participate in post-stroke motor recovery and the effects of DBS. This exploration of mechanism may inform the optimization of the intervention. It is possible, but due to DBS artifact images acquired will not be useful in the research. The investigators will work to filter the artifact as much as possible in order to make this data scientifically meaningful.

PET will be acquired at the following phases:

- At baseline, prior to DBS lead implantation

- During the rehabilitation phase
- During the rehabilitation + stimulation phase
- During the rehabilitation follow up phase

Local Field Potentials (LFPs)

Local field potential recordings will serve as an exploratory measure to evaluate the physiology of the dentate nucleus during both motor and non-motor behaviors. Preclinical models have revealed frequency-specific oscillatory changes within the region of the cerebellar dentate during reach and grasp tasks, with the nature and magnitude of those changes differing between successful vs. "missed" reach trials [181]. It is anticipated that these results will provide unique insight into the role of cerebellar activity in behavior generally while informing the future development of classifiers for a more physiologically-based, closed loop DBS therapy. A key advantage of closed-loop DBS would be a more selective "on demand" stimulation paradigm aimed at affecting, quasi-exclusively, cerebellar output specifically in relation to motor activity. It is also anticipated that characterizing electrophysiological activity in relation to the topographic distribution of the DBS contacts may help to further refine surgical targeting by delineating, for the first time in humans, the motor and non-motor areas of the dentate nucleus.

TMS Motor Maps

TMS motor maps will serve as an exploratory measure to characterize and compare cortical changes associated with physical rehabilitation alone and in combination with DBS. Expansion of motor maps has been observed to correlate with treatment-related improvements in motor function in stroke animal models[124] and in human studies[164, 182, 183]. It is hypothesized that enlargement of TMS motor maps will correspond to therapeutic improvements in upper extremity function and that enlargement will be greater in response to study treatment (Rehab + DBS) than rehabilitation only. It is anticipated that mapping of the perilesional cortex can serve as one added measure of efficacy that, if corroborated, will provide additional supportive evidence to advance this research beyond feasibility towards a multi-center pilot study.

H-Reflex

H-reflex will serve as a measure of spasticity and the neurophysiology of movement control. H-reflex is an electrical analogue of the stretch reflex and is commonly used to study motor control. The technique involves stimulation of a peripheral nerve (Median Nerve) using a short duration rectangular electrical pulse (~1msec) and collecting the response from the target muscle (carpi radialis) using surface electromyography (sEMG). The intensity (mA) of the stimulation is gradually increased and changes in characteristics of responses captured. Larger diameter Ia afferent fibers are recruited at a lower intensity that lead to postsynaptic depolarization of alpha motor neurons, which further lead to depolarization of the target muscle. This reflex response is called H-reflex. As intensity is increased, the smaller diameter motor axons are directly stimulated leading to a direct activation of the muscle. This direct activation response is called M wave.

TMS Cerebellar Brain Inhibition (CBI)

Cerebellar brain inhibition (CBI) will serve as an exploratory measure to characterize and compare cortical changes associated with the facilitatory activity in the dentatothalamocortical pathway. Evaluating CBI before cerebellar implant may contain valuable prognostic information. It is hypothesized that patients who demonstrate a greater response to cerebellar TMS on cortical motor output (i.e., CBI) may have a more robust dentatothalamocortical pathway and be more likely to benefit from cerebellar DBS (i.e., test treatment). Also, changes in CBI values pre-implant compared to post-explant may reflect an increase in cerebellar influence on motor cortices and, thereby, potentiation of its contribution to restored output to the paretic upper limb.

6.0 SUBJECT SELECTION

Study Patient Population

Subject will not participate in any study-specific tests or procedures before written informed consent. The study will begin only after the study has received IDE approval from the FDA and Institutional Review Board approval.

Subjects participating in the study shall be medically and neurologically stable individuals who have experienced an ischemic stroke 12 to 36 months and who continue to have residual, severe, unilateral, upper extremity hemiparesis. Severity of upper extremity hemiparesis is operationally defined as a score of ≤ 42 on the upper extremity subscale of the Fugl-Meyer Assessment (FMA-UE).

Subjects will be selected to participate in the study based on the inclusion and exclusion criteria described below. Subjects will not be excluded on the basis of gender or minority status.

6.1.1 General Inclusion Criteria

Subjects must meet all of the following general inclusion criteria.

1. First-time ischemic stroke 12 to 36 months prior to implant. The index stroke must have been documented by computerized tomography (CT) or magnetic resonance imaging (MRI).
2. Unilateral stroke in the territory of the middle cerebral artery (MCA) sparing the diencephalon the basal ganglia.
3. 25 to 75 years of age.
4. Transcranial Magnetic Stimulation criterion: Ability to elicit a muscle evoked potential (MEP) in response to TMS delivered to scalp. In a contracted state of the paretic muscle (ranging from 20-50% of maximum voluntary contraction), the ability to evoke reliable criterion MEPs (≥ 100µV in 5/10 trials).
5. Medically and neurologically stable as determined by the investigator based on the medical history, physical and neurological examination.

6. Moderate to severe residual unilateral upper-extremity hemiparesis defined as ≤ 42 on the upper extremity subscale of the Fugl- Meyer Assessment (FMA-UE) of the affected side. The FMA-UE shall remain 44 or below (FMA-UE ≤ 44) after eligibility-rehab in order to be eligible for implant.
7. A score ≥ 1 on the FMA-UE elbow flexion, elbow extension, or finger mass.
8. A Modified Rankin Scale score (mRS) < 4.
9. Absent or moderate spasticity in affected limb in any region (shoulder internal rotators and adductors, elbow flexors, wrist flexors or finger flexors) defined as a score of <4 on the modified Ashworth Spasticity Scale.
10. A score of ≥24 on the Mini Mental State Examination.

6.1.2 General Exclusion Criteria

The subject must not meet any of the following general exclusion criteria.

11. Primary hemorrhagic stroke or major hemorrhagic transformation (Note: Individuals who experience an ischemic stroke with minor hemosiderin in the parenchyma are not excluded.)
12. Any progressive neurological or physical condition other than the index stroke impairing function of the target extremity.
13. Moderate to severe hemispatial neglect or anosognosia involving the affected side of the body.
14. Any other neurological condition that could reduce the safety of study participation including central nervous system vasculitis, intracranial tumor, intracranial aneurysm, multiple sclerosis, or arteriovenous malformations.
15. Pain in the affected limb greater than or equal to 5 on a 0-10 NRS.
16. Evidence of a severe sensory deficit as measured by a score of 2 on the Sensory item (item 8) of the NIH Stroke Scale.
17. Unable to discontinue anticlotting therapy (i.e. antiplatelets and/or anticoagulants) at least 10 days prior to surgery. In the event of a hemorrhagic complication, resuming anticoagulation may be contraindicated for several months.
18. Seizures since the time of stoke, with or without use of antiepileptic agents.
19. Diagnosis of an underlying seizure disorder or epilepsy
20. Change in oral spasticity medications 2 weeks prior to consent or Botox injections in the affected arm within 4 months prior to consent, and/or intention to initiate anti-spasticity medications or Botox injections during study follow-up through 12 months post implantation.
21. Major active psychiatric illness that may interfere with treatment, such as psychotic disorders or severe personality disorders.

22. Untreated or inadequately treated depression defined by a score of 20 or greater on the Beck Depression Inventory-II at the time of enrolment.
23. A diagnosis of dementia.
24. Chronic, uncontrolled high blood pressure, history of severe and unmanaged cardiovascular disorder.
25. Contraindication to magnetic resonance (MR) imaging, e.g. weight incompatible with scanner, implanted metallic devices or electrical devices (pacemaker, defibrillator, spinal cord stimulator).
26. Severe and poorly managed medical disorders that, in the opinion of the PI, requires exclusion from the study due to expected risk likely greater than expected for the stroke population.
27. Enrolled in another device, biologic or pharmaceutical study within 30 days of consent in the current study, (i.e. patient cannot be enrolled if participation in another study was not completed at least 30 days prior to consent.)
28. Non-pregnant women. Women of childbearing potential must be using acceptable forms of contraception. Pregnancy will result in exclusion or discontinuation from the study.
29. Undergone a decompressive hemicraniectomy.
30. Patient has significant chronic small vessel ischemic disease, vertebrobasilar vascular disease, and/or any other structural abnormalities of the cerebellum, cerebellar peduncles, and brain stem that would preclude safe placement of the DBS lead.
31. Patient has a condition that, in the opinion of the investigator, would significantly increase the risk for interfere with study compliance, safety or outcome
32. Patient has an active implantable device (e.g. pacemaker, implantable cardiac defibrillator, implantable neurostimulator, or implantable drug pump) that will not been explanted prior to implant of the DBS system.

6.1.3 Transcranial Magnetic Stimulation Subject Inclusion Criterion

Transcranial magnetic stimulation (TMS) is a non-invasive method for mapping cortical motor representation. When TMS is used over the motor cortex at low stimulus intensities, it indirectly stimulates the corticospinal tract.[13, 145] This stimulation can be recorded as motor-evoked potentials via surface or indwelling electrodes at the target peripheral nerves. The proposed study will utilize the Magstim 200^2 magnetic stimulator for the TMS testing.[146]

TMS is included as an inclusion criterion in order to select subjects most likely to benefit from the proposed feasibility study of DBS therapy in post-stroke survivors. It is expected that verifying the integrity of the corticospinal tract (CST) with TMS will select subjects that are most likely to benefit from enhanced cortical excitability produced by DBS.

<u>TMS Inclusion Criterion</u>

The extensor digitorum communis (EDC) of the impaired upper extremity is the target muscle. TMS stimulation will be applied to the ipsilesional hemisphere (contralateral to the impaired limb). Single-pulse TMS (Magpro) will be delivered using a figure-of-eight coil. The coil will be placed tangentially on the scalp with the handle oriented backwards and laterally at 45° from mid-sagittal axis. Patient's MRI will be used to localize and guide the coil using frameless stereotaxy. Stereotaxic localization of the coil will help ensure that application of TMS is repeatable and consistent. The location specifically would lie in the motor cortex. In this region, we would identify the location of the "hot spot". The "hot spot" is defined as the scalp site where single pulse stimulation (.2 Hz) results in reliable muscle evoked potentials (MEPs) (criterion: >=100µV in 5/10 trials) in the active state of the muscle (20-50% of maximum voluntary contraction) at the lowest intensity. Closely spaced surface EMG electrodes will be placed over the mid-belly of the target muscle.

An individual meets the inclusion criterion if there is an ability to elicit MEPs that meet the above criterion. An inability to elicit MEPs that meets the above criterion at any location over the ipsilesional motor cortex at maximum intensity of the device will exclude the potential candidate from being enrolled in the study. A seizure at any point during the testing of the TMS inclusion criteria will discontinue all further testing and exclude the potential candidate from being enrolled. Since patients below UE FMA <20 will recruited as well, it is likely that such patients EDC may not evoke any MEP. In such cases, electrodes will be applied to proximal muscles that are more likely to have spared pathways in patients with severe damage. We will elicit MEPs in triceps and middle deltoid muscles as alternatives. Electrodes will be placed over the muscle belly at the junction of the middle and the lower third of the muscle. Electrodes will be applied in a direction that is parallel to the direction of the muscle fibers.

<u>TMS Procedure</u>

Single-pulse TMS (Magstim) will be delivered using a figure-of-eight coil (70mm, Magstim). The coil will be placed tangentially on the scalp with the handle oriented backwards and laterally at 45° from mid-sagittal axis. Patient's MRI will be used to localize and guide the coil using frameless stereotaxy. The software will register the MRI with patient's cranial landmarks. Stereotaxic localization of coil will ensure that application of TMS is repeatable and consistent.

MT will be determined for the contralateral muscle as explained in original guidelines for use of TMS in motor systems. While patients rest their hands on a flat surface, TMS-evoked responses, called motor evoked potentials (MEP), will be recorded using surface electromyography via bipolar electrodes (silver-silver chloride, 8 mm diameter) positioned over muscle belly. The skin overlying the forearm will be cleansed and rubbed briskly to reduce inter-electrode skin impedance to below 10 Kohm. A ground electrode will be secured over the acromion after the skin is prepared so differences in impedance between each ground electrode to reference electrode array is less than 5 Kohm.

Essentially, using stereotactic guidance and patient's MRI, TMS would be delivered to a number of candidate sites located in the motor cortices. These sites will be targeted one by one at intensities starting from 50% (expressed as 50% of the maximum output of the TMS device). Intensity will be increased till a site is found to elicit criterion-level MEPs (criterion: >=100µV in 5/10 trials) in the contralateral muscle. Intensity will be titrated so the lowest TMS intensity that generates criterion-level MEPs is identified. MT will be defined as the lowest TMS intensity that generates criterion-level, minimally perceptible MEPs in the muscle. The site that elicits criterion-level MEPs at MT intensity will be called the 'hotspot'. In patients with severe paresis, MT may not be elicited in resting state of the muscle; in this case, patients will be asked to contract their paretic muscle (20-50% contraction) to facilitate elicitation of MEPs. In such cases, the lowest intensity required to generate criterion-level MEPs in the paretic muscles will be termed active MT or aMT. Location of 'hotspot' and its position on patent's MRI will be registered with the stereotactic software so that at each visit we can reliably target the hotspot. With treatment and recovery over time, location of hotspot can shift/reorganize, and intensity of MT/aMT can adapt. To account for this possibility, besides targeting original locations using original MT values, changes in location of hotspot and changes in MT will also be identified to reliably track recovery.

MRI-guided stereotactic software ensures consistency in application of TMS. The software registers patient's head, and their MRI with the location of the coil in 3-D space. As such, the software informs the investigator of real-time location of all three elements. The software provides constant information about displacement of the coil in 3 axes (x, y, and z) and its rotation in 3 axes, i.e. yaw, pitch, and roll. This information is offered as graphical illustrations of real-time relation between elements, and as data reflecting the degree of mismatch, if any. Once a hotspot is identified and labeled as a target for TMS application, then the stereotactic software ensures that there is no linear displacement or rotation of the coil in any plane with respect to the original targeting of the site. If there is any displacement or rotation, then the software alerts the investigator by showing 'in red' as to what and where the error lies. Errors and their corrections are all displayed in real-time so adjustments can be made online during testing.

Subject Screening

All potential study subjects will be approached for screening in the study. It is expected that eligible participants will be competent to provide informed consent. Each potential study subject will be given an explanation of the study, instructed regarding the screening process and any required testing, educated on the possible risks and benefits to participating, and be asked to provide written informed consent.

An independent consent monitor will conduct a separate interview to verify that patients understand the consent well. This will provide an added layer of safety that is good in a first-in-human study.[1]

[1] An independent consent monitor is added as per discussion in face-to-face meeting on July 20, 2015 with FDA.

Written informed consent will be obtained prior to any study-specific testing. Study personnel will explain that even after written informed consent is provided, subsequent MRI or other testing may demonstrate that the candidate is not a suitable subject for DBS treatment and the candidate will not continue participation in the study

Subject Enrollment

A subject is enrolled in the study after they have signed the informed consent, and it has been determined by the investigator that they meet all of the inclusion criteria and none of the exclusion criteria.

7.0 STUDY VISITS

Study Schedule

Figure 2 provides a study timeline and Figure 3 provides a study flow diagram from the point of subject consent through completion of follow-up visits. Schedule of Study Visits

Table 2 displays the schedule of study visits during the study phases.

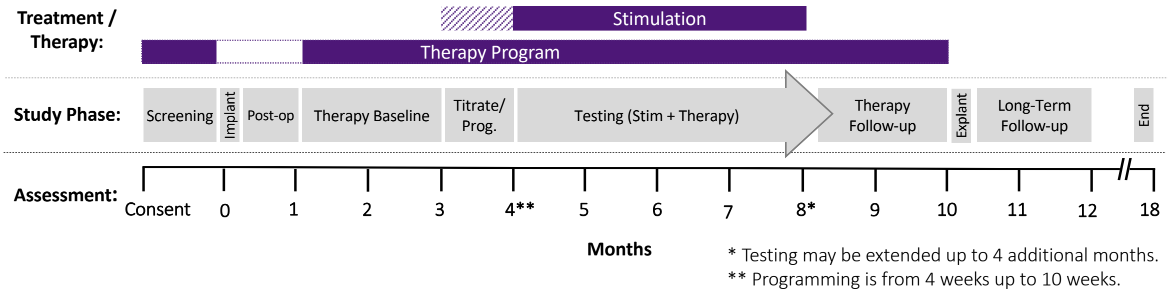

Figure 2: Study Timeline

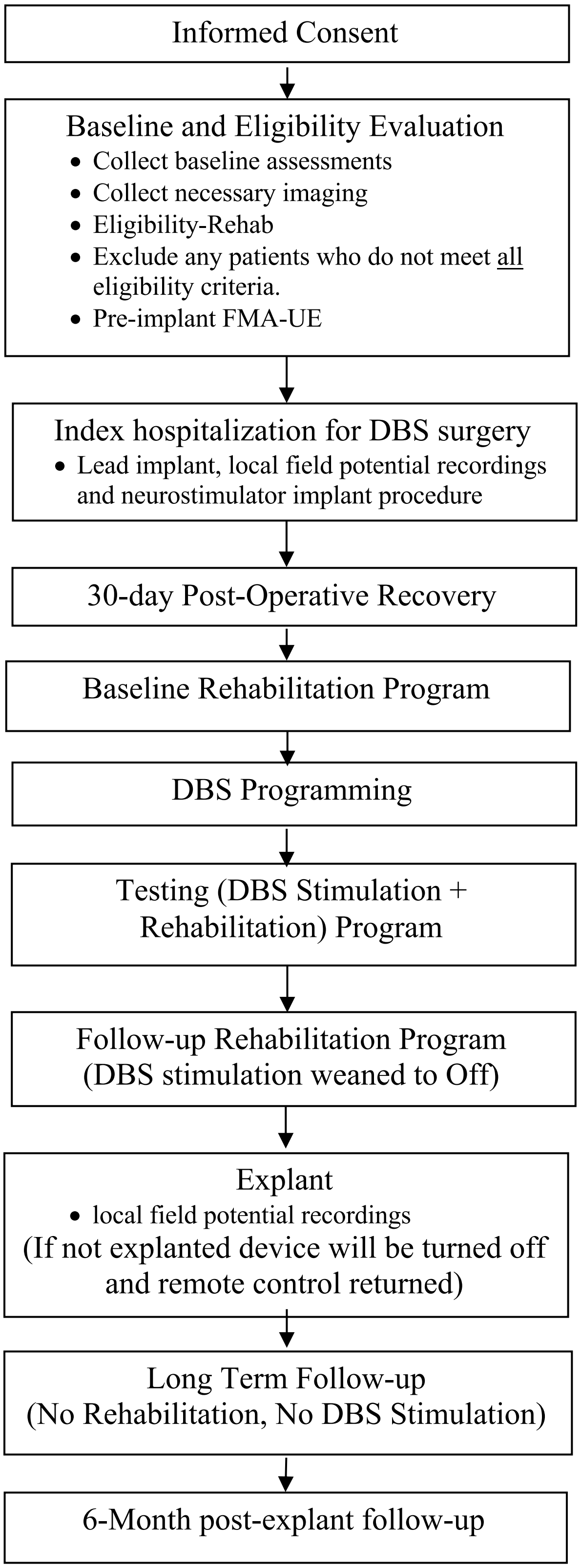

Figure 3: Study Flow Diagram

7.1.1 Schedule of Study Visits

Table 2. Schedule of Study Visits

Study Phase	Visit	Schedule
Baseline and Eligibility Evaluation	As needed to complete screening for eligibility and baseline evaluations including pre-implant FMA-UE	~Two months
DBS Implantation Hospitalization	DBS Electrode Implant, Local Field Potential Recordings, and DBS neurostimulator Implant Procedure	Up to 7- day hospitalization.
Postoperative Recovery (4 weeks)	N/A	DBS Electrode Implant = day 0
Baseline Rehab (8 weeks)	Month-1 Visit<br>Month-2 Visit	Starts day 28 (± 10 days)<br>day 56 (± 10 days)
Programming (4-10 weeks)	Month-3 Visit	Starts day 84 (± 10 days)<br>Up to 16 visits in a period of 4 - 10 weeks for programming the DBS system prior to the testing phase
Testing: stimulation of the dentate nucleus + Rehab 2x/week (16 weeks)	Month-4 Visit<br>Month-5 Visit<br>Month-6 Visit<br>Month-7 Visit	Starts day 112** (± 10 days)<br>day 140 (± 10 days)<br>day 168 (± 10 days)<br>day 196 (± 10 days)*
Rehab Follow-up (8 weeks)	Month-8 Visit<br>Month-9 Visit<br>Month-10 Visit	Starts day 224 (± 10 days)<br>day 252 (± 10 days)<br>day 280 (± 10 days)
Explant	DBS neurostimulator Explant, Local Field Potential Recordings, and DBS Electrode Explant procedure	Up to 7-day hospitalization.<br>Explant will occur within 2 months of prior visit (≤ day 336)
Long-term Follow-up	Month-11 Visit<br>Month-12 Visit	336 (± 10 days)<br>365 (± 10 days)
Study Completion	Month-18	547 (± 20 days)

** Programming was extended from 4 weeks up to 10 weeks. The subsequent visits schedule will be shifted by the number of weeks (7-day increments) beyond the original 4 weeks.
* Testing may be extended up to 4 additional months (see §7.7.1) with each visit scheduled an additional 28 days (± 10 days) from the previous visit. All subsequent visits will be shifted 28 days.

7.1.2 Schedule of Study Assessments

Table 3 provides an overview of the schedule of assessments required for the study. Study assessments, at other unscheduled visits, will be performed as clinically indicated and corresponding data shall be reported on appropriate study case report forms.

Table 3. Schedule of Study Assessments

Study Phase:	Screening	Baseline	Implant	Recovery	Rehab Baseline		Prog.	Testing (Rehab + Stim)				Rehab Follow Up			Explant	Long-term follow-up
					Monthly Visits:											
					1	2	3	4	5	6	7	8	9	10		
Demographics	X															
Medical History	X															
Stroke History	X															
Physical/General Clinical Exam	X			X										X		
Neurological Exam	X															
NIH Stroke Scale (Item 8)	X															
Ashworth Scale	X				X	X	X	X	X	X	X*	X	X	X	X	X
Neuropsychological evaluation	X							X						X		
Laboratory Tests (Blood and Urine)	X		X												X	
Structural MRI†	X															X
PET		X				X					X*			X		
Diffusion Tensor Imaging		X														
CT scan			X				X									
Local Field Potential Recordings			X												X	
TMS – MEPs	X		X			X	X	X	X	X	X	X		X	X	
TMS – Motor Maps	X	X				X	X	X	X	X	X*	X		X	X	
TMS – CBI	X	X														X
H-reflex	X					X	X	X	X	X	X*	X		X	X	
9-Hole Peg Test		X			X	X	X	X	X	X	X	X	X	X		X
Arm Mobility Action Test (AMAT)		X			X	X	X	X	X	X	X	X	X	X		X
Beck Depression Inventory (BDI)		X			X	X	X	X	X	X	X	X	X	X		X
Bilateral Box and Block Test (BBT)		X			X	X	X	X	X	X	X	X	X	X		X
Bimanual Grip Strength		X			X	X	X	X	X	X	X	X	X	X		X
EuroQol (EQ-5D)		X			X	X	X	X	X	X	X	X	X	X		X
Fugl-Meyer Assessment, (FMA-UE)	X	X			X	X	X	X	X	X	X	X	X	X		X
Short Form Health Survey (SF-12)		X			X	X	X	X	X	X	X	X	X	X		X
Concomitant Medications		X	X	X	X	X	X	X	X	X	X	X	X	X		X
Adverse Events			X	X	X	X	X	X	X	X	X	X	X	X	X	X
DBS Device Check			X				X	X	X	X	X	X	X	X		
Rehabilitation Sessions		X			X	X	X	X	X	X	X	X	X			

* These assessments will occur only at the final month of the Testing Phase.
† If a participant has an MR Conditional implant, the MR imaging used for the study will be performed in conformance to the MR Conditional labeling and may be performed at a field strength lower than 7T.

Baseline and Eligibility Evaluation

The following is a list of all required screening and baseline evaluation assessments:

- Medical History
- Stroke History
- Rehabilitation History
- Physical Examination (pertinent to their medical and neurological status)
- Neurological Examination (Include assessment of spasticity with the Ashworth Scale, and sensory assessment with the NIH Stroke Scale Item 8)
- Neuropsychological Examination
- Rating with all primary and secondary outcome measures
- Diffusion Tensor Imaging (DTI)
- FMA-UE
- PET

Assessments collected during screening will be used for baseline to avoid duplication. At the conclusion of the baseline testing, enrolled patients will be scheduled for DBS implant surgery.

During the baseline and eligibility evaluation, the subject will undergo a period of eligibility-rehab prior to implantation to avoid enrolling patients that can still benefit from physical therapy. The rehabilitation will include 4 weeks of in-clinic rehab. As described in §7.5.1 "Description of the Rehabilitation Program" the therapy sessions will occur two times per week for 1-1.5hrs and focus on functional tasks using adaptive task practice (ATP) and repetitive task practice (RTP). Subjects shall be rescreened at least 2 weeks prior to surgery and the FMA-UE shall remain 44 or below (FMA-UE $\leq$ 44) after eligibility-rehab in order to be eligible for the DBS Implantation Procedure.

Unilateral DBS Electrode Implantation

The subject will be admitted to the Cleveland Clinic for the DBS surgery.

The brain target site is the dentate nucleus of the cerebellum ipsilateral to the side of the body with upper extremity weakness (contralateral to the lesioned hemisphere). The brain target site will be localized for stereotactic implantation of the DBS lead using CT scans and MRI. A preoperative MRI will be fused with a preoperative stereotactic CT, to be acquired with the Leksell head frame depicts the position of the patient and the placement of the stereotactic head frame.

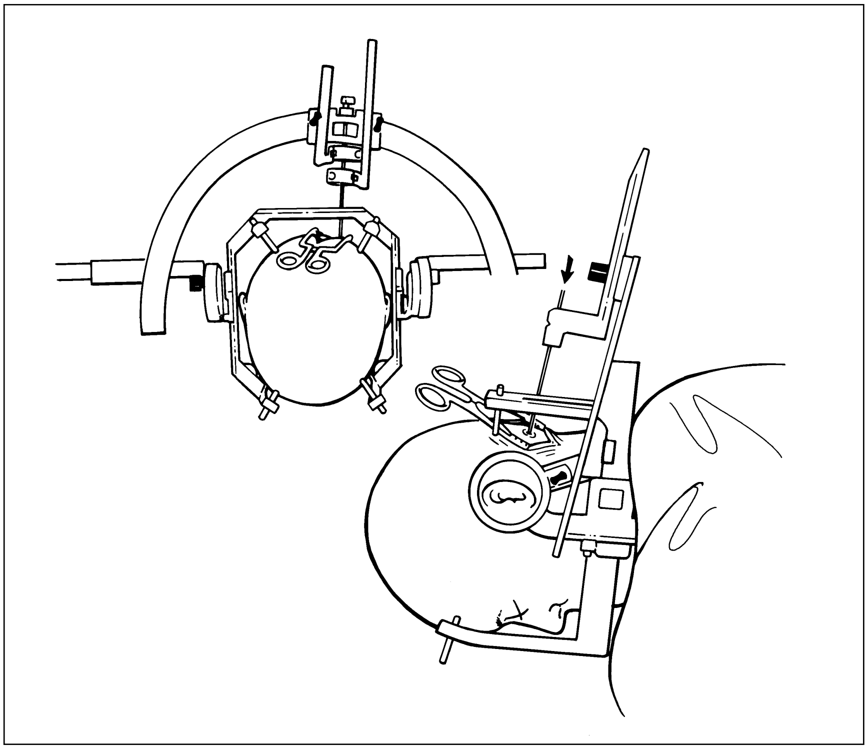

Figure 4. Illustration of Patient Position and the Placement of the Head Frame

The target will be selected on triplanar reformatted MR images by means of direct targeting. The intended target is the output of the dentate nucleus, as it projects to the superior cerebellar peduncle. In the proposed dentate nucleus region, implantation of the electrode will assume a trajectory from posterior to anterior, extending from an entry point in the occipital surface of the cerebellum to the rostral limit of the dentate nucleus and its output. The surgical goal will be to place the contacts flanking the area of interest which ranges from the region immediately rostral to the dentate nucleus (where white matter output of the nucleus projects to the superior cerebellar peduncle) to the caudal-lateral region of the dentate nucleus.

The trajectory will be planned using standard stereotactic techniques as to enter the occipital surface of the cerebellum, from a lateral to medial approach. As for any stereotactic procedure, the trajectory will avoid dural sinuses and the midline. Variability in the trajectory angles of approach to the dentate nucleus will depend on individual vascular anatomy. As with other stereotactic procedures, it is anticipated that there will be some variance in the location of superficial vessels of the cerebellum. Superficial vessels will be avoided and trajectories that will avoid the topography of larger sub-cortical vessels will be favored. The trajectories will also avoid the IV ventricle. The trajectories will therefore transverse the following structures:

1. Skin, muscle over the planned burr hole
2. Occipital bone, lateral to the midline and medial and inferior to the sigmoid and transverse sinuses, respectfully. It will be superior to the foramen magnum.

3. Dura mater, arachnoid and pia. These will be coagulated with bipolar coagulation prior to opening as we routinely do in stereotactic procedures at Cleveland Clinic.
4. Cerebellar cortex
5. Cerebellar white matter
6. Dentate nucleus

The patient will be under general endotracheal anesthesia. In this protocol, we anticipate patients to be positioned prone under general anesthesia. If the choice is for general anesthesia, we will ask the anesthesia team to maintain anesthesia with agents that minimize interference with intraoperative evoked responses during the intraoperative physiology time. Intraoperative imaging (fluoroscopy or O-Arm acquisition) will be used to provide intraoperative feedback, following the routine for frame-based DBS lead implantation at Cleveland Clinic.

An O-Arm will be utilized to acquire three-dimensional images intraoperatively. Once the electrode is inserted with stereotactic techniques, a volumetric stereo image with the O-Arm will be acquired and co-registered with the preoperative image using the planning station. Then the extent of error between the intended location of the lead and the actual location of the lead will be estimated. Large differences between the intended and actual location of the lead, such as 1.5 mm, may require the lead to be relocated.

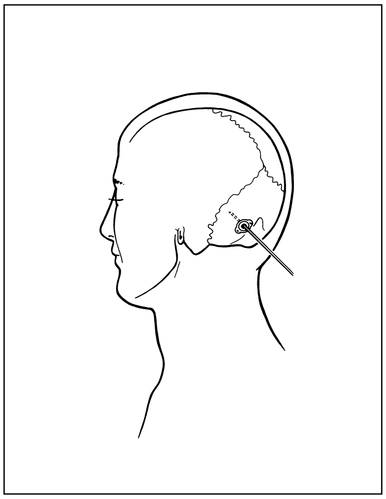

Figure 5. Illustration of Electrode Placement for the Dentate Nucleus Target

7.1.3 Intraoperative Macroelectrode Testing

Electroencephalography will be used during surgery for evoked potentials. Evoked potentials will be generated from the cerebellar electrode and recorded by EEG. In addition, the patient will also be monitored by median nerve somatosensory evoked potentials (SEPs). It is anticipated that the effective contact for dentate stimulation

will generate evoked potentials around and outside the infarcted zone in the motor, pre-motor and parietal regions of the brain.

Once the DBS electrode is inserted, macroelectrode testing will be performed. Test stimulation with the DBS electrode will be attempted. Direct observation, EMG and EEG recordings will be used to evaluate motor and electrophysiological effects of macrostimulation with an external stimulator. In addition, MEPs and SSEPs may be used as test stimulation while evaluating the effects of deep brain stimulation of the dentate nucleus on the magnitude of these evoked potentials.

It is anticipated that in the first few subjects implanted in the study, that these electrophysiological measures would not be used to confirm or refine lead location. However, as experience is gained during the course of this investigation, it is possible that some electrophysiological measures will be used to confirm or refine lead location. As such, the electrophysiological measures to be collected in this study will be exploratory but may be used in some subjects to confirm or refine the location of the lead.

Undesirable effects (i.e. motor contractions at low thresholds) will be evaluated by increasing stimulation amplitude until a motor response is noted (i.e. motor threshold). All stimulation will be performed within charge density safety limits. Any effects observed during surgery such as motor contractions are expected to be fully reversible and not associated with any long-term deficits.

1. For each of the electrical contacts in the DBS lead, the amplitude will be set at 1mA, the pulse width at 90 microseconds, and the frequency at 30 Hz. The patient will be monitored for SSEPs, EEG and EMG. The amplitude will be increased in increments of 0.5mA or 1.0mA. The maximum amplitude will be determined by charge density limits, as discussed below. In addition, we will consider an amplitude "maximal" and no longer increase the amplitude if motor contractions are noted.

2. Once each of the electrical contacts has been tested as outlined above, additional parameters may be tested depending on intraoperative conditions (i.e. stability of vital signs, etc.). The testing, as outlined above, will be repeated at the same frequency (30 Hz) but a pulse width of 120 microseconds.

3. Finally, depending on surgical progress and anesthesiology, the testing will be repeated at a frequency of 100 Hz and pulse width of 90 microseconds.

After the final location for the lead is selected in the target area, the lead will be secured in the burr hole ring. Then the lead will be coiled in an upwards direction and the wound closed with a connector protecting the tip of the DBS lead. The wound will then be closed in standard surgical fashion.

7.1.4 Local Field Potential Recordings

The DBS lead will be externalized via an extension to allow for study-related local field potential recordings (LFP) from the region of the dentate nucleus. The subject will remain in the hospital throughout this period at implant or in the on-campus Cleveland Clinic hotel between staged explants. EMG, scalp EEG, and LFP using

the eight contacts of the implanted DBS lead will be recorded throughout the electroencephalography to characterize changes and to identify task- or stimulus related neural activity in response to sensory stimulus, motor, non-motor, and direct activation. Spontaneous (i.e., not related to any specific task or stimulus) neural recordings may also be made during times when the subject is at rest, including overnight recordings to examine changes in scalp EEG and subcortical LFP synchrony.

Subjects will participate in daily data collection tasks for up to 8 hours per day. Subject activities will be performed in short segments, broken up across a morning and afternoon session, allowing for frequent rest to accommodate the subject and improve data quality which would be affected by subject fatigue. In order to accommodate the subject (e.g. does not feel up to it, has a headache, etc.) or to work around nursing care, the data collection will be distributed throughout externalization. Attempts will be made to have the 2-staged surgical procedures occur during the same week with data collection activates in-between. For instance, if the first procedure occurred at the beginning of the week (e.g. Monday) and the second occurred at the end of the week (e.g. Friday) all data collection would be completed in a contiguous week. In the event data collection occurs across a weekend, the subject may have an externalized lead for up to 7 days.

Tasks will consist of computerized cognitive tasks and arm activities:

Sensory Tasks. Standard techniques will be used to collect auditory and visual evoked potentials (e.g. light and checker box). Also, similar to its use during the intra-operative recording procedures (§7.3.1 Intraoperative Macroelectrode Testing), evoked potentials will be generated from the cerebellar electrode and recorded by EEG. The subject will also be monitored by median and tibial nerve somatosensory evoked potentials (SEPs) using standard techniques.

Passive Movement Task. Specific joints and combinations of joints across the subject's arm will be passively articulated either by an investigator or through the use of the InMotion ARM robotic system. The state of the muscle may be confirmed using EMG throughout the examination. Individual articulations will be performed cyclically with a brief delay between cycles. This will be repeated for elbow flexion/extension, shoulder protraction/retraction, and shoulder internal/external rotation, roughly 10 minutes per condition.

Active Motor Tasks. The subject will perform a series of simple motor tasks involving active articulation of one or more joints of the affected and unaffected upper extremity or eye movements. When eye movements are used as part of the task, a video-based eye tracking system will be used to monitor movement. Tasks will be computer controlled, such that all motor-related cues (e.g., 'ready', 'go') synchronized to the electrophysiology recording system for subsequent time-locking analysis. Movement tasks will be unsupported (i.e., spontaneous performance) and supported, with the latter facilitated by the InMotion robotic system, which provides measured, therapeutic assistance to the patient during task performance. Additional tasks will include hand and finger precision grip or joystick-based tasks to characterize control. Typically, each task begins with the subject grasping the

manipulandum with the affected or unaffected hand and positioning the cursor within the 'home' location, such as a circle, displayed on the computer screen. Each trial begins in that location with movement preparation or 'go' subsequently indicated audibly or by the appearance of one or more additional cues on the display screen.

Non-Motor Tasks. A selection of computerized tasks will be used to interrogate non-motor functions that are thought to involve the reciprocal connections between cerebellum and frontal associative areas. These tasks will incorporate various constructs such as choice reaction time, temporal processing, stimulus-evoked attention, lexical search, cognitive control, visuospatial organization, and affective processing. All tasks will involve visual or auditory stimuli presented via computer and require either passive attention, or timed or untimed verbal, ocular, or manual responses (dependent on the gross motor capabilities of the patient). A classical eye blink conditioning task may be used, involving the pairing of an auditory or visual stimulus with an eye blink-eliciting stimulus (i.e., a mild puff of air to the eye). For certain tasks, the DBS Lead will be used to examine the facilitatory effects of the DBS on performance. Individual tasks will be separated by short breaks to minimize fatigue.

Direct Activation. Stable passive- or active-movement events may not be identified using LFPs. As such, the effect of acute TMS of perilesional cortex (previously identified motor regions) on LFP activity may be examined. It is anticipated that TMS will produce strong, synchronized evoked LFPs via the descending corticopontocerebellar pathway. In addition to providing insight into the nature and strength of coupling between the areas, both statically and as a function of treatment, this approach may provide topographical insight to guide future surgical targeting. A similar alternative will be to explore the use of the DBS Lead as the stimulus source and examine the timing and topography of scalp EEG changes across perilesional regions as well stimulus temporally-paired with motor evoked potential activity elicited by TMS [165, 166].

7.1.5 Implantation of the Neurostimulator

In a second surgery, the DBS lead will be connected via an implantable extension to the neurostimulator. Prior to implantation of the neurostimulator, a new volumetric CT scan will be collected to verify lead location. The CT scan will be co-registered with the postoperative CT scan. While minor differences are expected between the two scans revision of the DBS lead will be considered if dislodgements greater than 2 mm are identified.

The neurostimulator will be implanted in the standard fashion, similar to the procedure routinely performed at Cleveland Clinic for the management of movement disorders. Briefly, the site for implantation will be planned for the infraclavicular region. The existing extension will be replaced. The extension will be disconnected from the DBS lead (clean site) and then pulled away through the percutaneous exit site in order not to bring any contaminants towards the implanted hardware. The percutaneous exit site will remain outside the prepped and draped area and only closed after the end of the surgery. A tunneling tool will be used to

create a tunnel from the head to the chest and an extension cable will be passed. The pocket for placement of the neurostimulator will be created subcutaneously and the neurostimulator will be implanted and connected to the extension. The proximal end of the extension will be connected to the DBS lead and the wounds will then be approximated in the standard fashion.

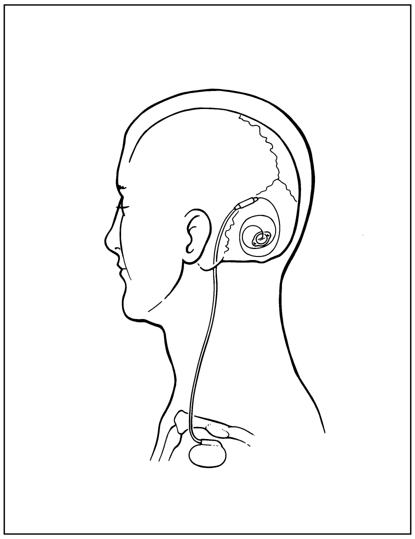

Figure 6. Final DBS System Placement

7.1.6 Hospital Discharge

Prior to hospital discharge the patient will be assessed to determine whether any adverse events occurred during the hospitalization. All adverse events shall be documented. Medications will be reviewed and changes documented. The DBS system will remain OFF.

Postoperative Recovery Period

All subjects will be discharged from the hospital with the DBS system programmed OFF. During the month following hospital discharge the patient will recover from implant surgery and will not receive DBS stimulation or any rehabilitation therapy. At the conclusion of the postoperative recovery period, the patients will return for a general clinical exam, and all patients will be asked about the occurrence of adverse events since hospital discharge. All adverse events shall be documented. All patients and/or caregiver will be trained on care of their devices as per patient labeling. Further training will be provided during Device Programing phase.

Rehabilitation

An outpatient rehabilitation therapy program will be initiated at the start of the Baseline Rehabilitation phase and continue throughout the Programing, Testing phase (Stimulation + Rehab) and Rehab Follow-up phase.

During the rehabilitation therapy program, there will be in-clinic outcome assessment visits (one per month) in addition to physical therapy visits. Physical therapy visits will occur either in-clinic or in an at-home setting via a Cleveland Clinic telehealth platform approved for patient care, with at last 25% of therapy sessions happening in-clinic. In each visit, patients will be asked about the occurrence of adverse events since their last study visit. All adverse events shall be documented. Medications will be reviewed and changes will be recorded. Patients also will be evaluated with the primary and secondary outcome measure.

Subject receiving therapy via the telehealth platform will be evaluated at the end of Therapy Baseline to ensure that their motor function does not regress. If motor function decreases during Therapy Baseline, more than 1 point on the FMA-UE, the subject will be required to receive in-clinic therapy.

7.1.7 Description of the Rehabilitation Program

The rehabilitation method is focused on practice of functional tasks using adaptive task practice (ATP) and repetitive task practice (RTP). Tasks will be chosen based on each individual's perception of their relevance in concert with optimizing therapy goals and reducing upper extremity motor impairment. Therapy will be provided by physical therapists or occupational therapists.

Stroke survivors who are candidates for deep brain stimulation present with limitations in movement across several upper extremity joints to the point where they cannot perform meaningful functions with the impaired upper extremity either independently or bilaterally (using both arms) much beyond perhaps placing the impaired arm into the sleeve of a shirt or jacket. Following the procedure, training must exploit the acquired movement capabilities across several joints beginning with the shoulder and moving down the upper extremity to the digits. The activation of these joints will be done to increase the active range of motion (AROM) the patient can achieve at each joint; for example, shoulder flexion to bring the arm onto a table and elbow extension to reach towards an object. AROM will then be integrated into activities that are progressively segmented towards a functional goal. Such segmentation is referred to as adaptive task practice (ATP) also called "shaping". The patient will be encouraged to increase their AROM in progressive stages with much verbal encouragement and praise based upon whether they can move faster or more times per unit of time. As reaching the target (for example, to pick up a can or cup) is achieved, the goal becomes better quality of function through repetitive task practice (RTP), repeating the task to completion over many efforts to reinforce the success that was first achieved through the shaping procedure.

The determination around which tasks to practice cannot be prescriptive or patients will not comply with task practice whether in the clinic or in the home. Therefore, we determine together those tasks which we call "challenges" and which are determined by the patient based upon how the patient values their importance. This negotiated approach fosters compliance and reinforces the desire to engage in more tasks. With each new task, we progress from the adaptive task practice mode to the repetitive task practice mode. The amount of time in this progression is dictated by

patient compliance and reacquisition of more motion. Most often patients select tasks related to grooming, eating, and bathing. Men often choose tasks that might be described as more physical and woman tasks that they associate with optimizing their functional behaviors prior to sustaining a stroke. The rate of progression is dependent on the severity of stroke and the motivation of the patient, so total numbers of tasks and their sequence can vary considerably. The ultimate goal is to make the patient motivated to undertake self-directed and sustained task reacquisition.

The therapy sessions will follow the same frequency and duration throughout the study. The frequency and duration of therapy sessions will be two times per week for 1-1.5 hours of treatment time over a 2-hour contact interval. The frequency of sessions during the variable length Programming Phase will be one time per week. In addition to the formal outpatient therapy sessions, subject will sign a behavioral contract that obligates them and their caregivers to continue with the upper extremity rehabilitation program at home.

During the Eligibility Rehabilitation period of 4 weeks the patient will participate in 8 therapy sessions. During the Baseline Rehabilitation period, patients will participate in 16 therapy sessions. For sessions 1 through 8, therapy will involve a 1:1 ratio of repetitive task practice (RTP) activities to adaptive task practice (ATP) activities. Sessions 9 through 16 will include a 1:2 ratio of RTP to ATP activities. This entire sequence of 16 sessions will be repeated at the start of Testing Period so that the same ratiometric relationships in training is used when their stimulation is turned ON. After they repeat the task ratios they will advance in their training based on the progress of the patient and recommendations of the therapist.

Throughout the study, the frequency and duration of outpatient rehabilitation sessions and the proportion of time spent on RTP and ATP exercises will be documented. Patients and caregivers also will be expected to record the type, frequency, and duration of home practice activities.

Therapists from the Cleveland Clinic will be providing the rehabilitation therapy and performing the outcome assessments for the study. To ensure consistency and compliance with the study protocol, therapists will receive training on the rehabilitation methods and administration of the outcome measures employed for the study.

Device Programming

The study team at the Cleveland Clinic Center for Neurological Restoration (CNR) will be responsible for programming and device follow-up. Programming of the device will utilize standard DBS techniques for assessing effects and side effects during a cathode survey of the DBS lead. In addition, programming will be guided by changes in electrophysiology during active and passive tasks (see §7.3.2) and by measuring the effects of acute stimulation on the magnitude of motor evoked potentials (MEPs) evoked with transcranial magnetic stimulation (TMS).

The primary purpose of this programming phase is to select the optimal chronic stimulation parameters to use during the testing (Stim + Rehab) phase of the study.

The principal investigator will be responsible for determining the final stimulation settings. Given the nature of this feasibility study, the selection of initial stimulation parameters for testing will be partially based on available animal data on deep cerebellar stimulation. It is expected, from the results of these animal studies (see preclinical data section), that stimulation frequency will be set in the range of 20-25 Hz. Final selection of chronic frequency, active contacts, pulse widths and amplitudes will be based on the titration testing using a systematic cathode survey.

Prior to the initial programming visit, a CT will be collected to verify lead location. The neurostimulator will remain Off during the CT. At the start of programming, the neurostimulator operation will be verified and impedances check. During the programming phase visits, a cathodal survey will be performed by activating, in a systematic fashion, each contact as the cathode. All stimulation testing will be conducted below the safe change density limit of 30 $\mu C/cm^2$/phase as described in the Boston Scientific Implant Manual. It has been demonstrated that TMS can induce currents in a DBS lead resulting in stimulation at the electrodes [147-150]. During the delivery of TMS, induced current will be included in the calculation of charge density to ensure that the overall charge density never exceeds 30 $\mu C/cm^2$/phase. The induced current will be calculated utilizing the measured contact impedance and an estimate of voltage conservatively estimates twice that reported by Rossi (i.e. 2 V). The charger density will be calculated, based on the programmed pulse width, overall current (induced current plus programmed current), and contact surface area.

Each cathode will be tested with stepwise increments in amplitude from 0 to a maximum of 10 mA. Pulse widths will be initially set at 90 microseconds and will not exceed 210 microseconds. Amplitudes will be increased until a side effect is noted or reported by the patient or the charge density limit is reached. Patient reports of subjective responses such as paresthesia, changes in the visual field or perception, or other unpleasant psychological or perceptual changes also will be considered in defining a maximal amplitude threshold for each contact.

In addition to direct observation, electromyography and transcranial magnetic stimulation will be used during DBS programming to determine the motor threshold (MT) and measure the effects of dentate deep brain stimulation on motor evoked potential (MEP) amplitude. Since, neurophysiological techniques are prone to patient variability and nonspecific factors such as fatigue, task-related movement activity may be characterized. Passive and active movements (supported, unsupported and against variable resistance) would be feasible with the use of an InMotion ARM™ robotic system. These performance measures with electrophysiology would address limitations and characterize clinically relevant functional benefit. Global Vigor and Affect Scale (GVA) and the Brief Fatigue Inventory (BFI) will be used in multivariable models to control for confounding effects of fatigue during programming. The GVA will be administered prior to each programming session (twice per day) to account for within-day variability. The BFI will be administered at the beginning of each programming day (once per day) to account for across day variability.

Any concern for long-term side effects will be avoided by reprogramming stimulation as soon as a side effect is noted during the programing phase.

7.1.8 Transcranial Magnetic Stimulation (TMS)

Transcranial magnetic stimulation (TMS) will be used with the goal of identifying changes (increments) in cortical excitability that may result from cerebellar stimulation. The objective is to select an optimal stimulation setting that will not cause adverse effects while at the same time resulting in a measurable increment of cortical excitability. TMS will be done at baseline (prior to stimulation testing), and intermittently throughout the systematic cathode survey. Enhancement of cortical excitability will be defined as a reduction in the motor threshold (aMT) elicited by TMS and gain in corticospinal output will be defined as an increase in the amplitude of the motor evoked potentials (MEPs) elicited by TMS.

To measure changes in corticospinal output, we will adopt a new standard-recruitment or input-output curve. The curve is plotted by investigating the MEP responses of a given muscle at incremental intensities. The curve is believed to reflect the output and the gain of the descending corticospinal tracts. In general, it has been established that all skeletal muscles demonstrate a sigmoidal curve—as stimulus intensity increases, a steep increase in recruitment occurs that eventually plateaus due to maximal activation of all available CST. The slope of this curve is generally stunted in patients with stroke, but change in slope across the incremental intensities is a strong indicator of increase in corticospinal output and its gain. In addition, the ipsilesional motor cortex will be mapped in such fashion as to establish the motor representation of the affected forelimb in the ipsilesional cortex during the study (i.e. before and after training and training + stimulation)

In patients who are below FMA < 20, standard recruitment curves or input-output curves can be challenging to acquire. This is because these curves rely on eliciting MEPs at several different TMS intensities, ranging from 90-150% aMT. Since in patients with severe deficit (FMA < 20) aMTs are typically high, it would preclude testing of curves at high intensities. We will study additional metrics that will serve as alternatives in such a case. We will record MEP amplitude in test muscle at 120% aMT (EDC typically, but triceps or middle deltoid in patients who have no MEP in EDC). While patients voluntarily contract their target muscle (EDC, triceps, middle deltoid) to 50-100%, we will apply TMS to ipsilesional and contralesional M1 separately. TMS can evoke transient suppression of on-going EMG in the paretic muscles that reflects change in intra-cortical excitability in ipsilesional M1 and inter-hemispheric influence from contralesional cortices. In our work [151] and of others, these metrics are acquired feasibly in patients with severe impairment as well.

Motor maps of the affected hemisphere will be created using stereotaxic-guided techniques[151, 163, 164]. An initial motor map will be repeated with two separate occurrences spaced at least one week apart, to ensure reproducibility. Stimulation will be applied at specific scalp sites at an intensity of 110% RMT[168], while the target muscle (EDC, triceps, or middle deltoid) remains at rest as confirmed by EMG. Starting from the hotspot, sites at incremental distances of 1 cm will be

targeted in a 3x5 rectangular grid as commonly used in our work and other studies[151,164,167]. Sites will be deemed responsive if they produced peak-to-peak MEPs of at least 30 μV over 3 out of 5 trials as discussed in recent work at the Cleveland Clinc[151,164,171]and used by others[172,173].

CBI of the affected hemisphere will be created using stereotaxic-guided techniques[151, 163, 164]. A pre-implant and post-implant CBI will be repeated with two separate occurrences spaced at least one week apart, to ensure reproducibility. CBI will be obtained from paired-pulse TMS stimulation with conditioning stimulation delivered to the cerebellar cortex, ipsilateral to the evaluated upper extremity, followed, roughly 5 ms later, by a test stimulus delivered to the contralateral motor cortex. The test stimulation will be applied with the 70 mm figure-of-eight coil. Inhibitory cerebellar TMS will be applied using the 110 mm double-cone coil operated below the threshold that elicits discomfort or pain.

7.1.9 Safety of Transcranial Magnetic Stimulation and DBS

To address any safety concerns about the proposed use of TMS to evaluate cortical excitability during interoperative stimulation testing with the DBS electrode implanted, and during follow-up with the DBS system implanted, a literature review was performed.

A significant body of literature clearly documents the safety of the proposed use of TMS to evaluate cortical excitability during testing of deep brain stimulation parameters with only a slight modification of standard TMS protocol. At least two independent groups have developed phantom skull models to test whether TMS induced currents in the scalp-coiled leads could result in damage to the patient's brain or to the implanted stimulators. Both studies concluded that stimulation over the skull at 1 cm from the coiled leads induced very small transient currents in the electrode wires that remain well below the amplitudes used for DBS and are therefore considered to be safe. Kumar (1999) [169] further showed that TMS over the scalp leads does not deliver damaging currents to the stimulators. The only potential concern raised by these studies was that inadvertent TMS directly over the neurostimulator could cause the neurostimulator to malfunction. Since the original phantom study by Kumar greater than 50 patients have undergone TMS evaluations of cortical excitability during deep brain stimulation. These have included TMS during DBS in either bilateral or ipsilateral basal ganglia and/or thalamus in patients with Parkinson's Disease, severe dystonia,[170] essential tremor, and intractable epilepsy. In all these DBS studies, no adverse effects of TMS were reported. The only modifications to standard TMS procedures was that in some of these cases, patients wore protective jackets that served as a cue for investigators to protect against the potential for inadvertent TMS application directly over the neurostimulator.

For TMS performed during post-neurostimulator implantation DBS programming, care will be taken to avoid stimulation near the neurostimulator. A Protective foam jacket with ≥ 7 cm thickness over the site of the neurostimulator will be worn by patients to avoid stimulation near the neurostimulator. Further, the expected induced currents in the DBS electrodes will be even smaller than those reported in the

phantom models because the DBS lead will be coiled under the muscle layer of the contralateral suboccipital region, relatively far from the placement of the TMS coil above the motor cortex. If the neurostimulator is placed on the non-paretic side of the body, which would be closer to the test hemisphere (ipsilesional) for TMS, then caution will be practiced in additional ways. Patients who require greater than 80% of TMS intensity will not be included. We do not believe that this extra precaution would be exclusionary because we will measure and record all TMS variables in the active state of the paretic muscle. To still ensure that neurostimulator does not malfunction, we will take a printout of all DBS parameters at beginning and will keep checking during TMS testing and at the end to ensure that values do not vary with TMS. TMS has been delivered with DBS in cases where exit site for leads is close to the motor cortex, i.e. in proximity to scalp site where TMS is applied. But, in our proposed project, the leads will be significantly posterior and thus are expected to have little interference from TMS. Therefore, the specific DBS protocol stimulating the DTC, and the proposed application, and safeguards for TMS testing will allow a safe synergistic application of both modalities in this feasibility study.

The literature cited above specifically address the use and safety of TMS to assess cortical excitability in patients with implanted DBS devices, and supports the conclusion that TMS over the scalp will not cause damaging stimuli to the patient's brain, to the DBS electrode or neurostimulator following the testing procedures described in this section.

Testing Phase (stimulation + rehabilitation)

After completing the Baseline Rehabilitation phase and DBS programing the DBS stimulation will be initiated. The Subject will continue their rehabilitation program. Settings for chronic deep brain stimulation will be selected based on the prior DBS programming phase. However, the investigators will repeat the procedures for DBS programming which will include TMS monthly during the Testing phase (rehabilitation + DBS). This procedure will be done in order to account and compensate for possible accommodation of the neural network to stimulation during the testing phase.

During the testing phase visits, all patients will be asked about the occurrence of adverse events since their last study visit. All adverse events shall be documented. Medications will be reviewed and changes will be recorded.

Outcome measures will be assessed once/month. Programming of the neurostimulator will be performed in the same manner as the initial device programming and subjects will have their stimulation parameters adjusted. Subjects will be programmed to stimulation settings found to be optimal for enhancement of cortical excitability while avoiding side effects. Again, TMS will be used to index cortical excitability.

The goal is to re-measure cortical excitability, again indexed by TMS, and learn if there are changes with chronic stimulation. If changes are noted, the DBS will be re-programmed in the same fashion as during the programming phase. If changes are

not noted, DBS will be checked (i.e. evaluation of impedances, ascertain that stimulation is ON) but not reprogrammed

A cathodal survey may be performed by activating, in a systematic fashion, each contact as the cathode. All stimulation testing will be conducted below the safe charge density limit of 30 microcoulombs/cm2/phase as described in the Boston Scientific Implant Manual. Each cathode will be tested with stepwise increments in amplitude from 0 to a maximum of 10 mA. Pulse widths will be initially set at 90 microseconds and will not exceed 210 microseconds. Amplitudes will be increased in stepwise fashion in increments no greater than 1 mA at a time until a side effect is noted or reported by the patient or the charge density limit is reached. Patient reports of subjective responses such as paresthesia, changes in the visual field or perception, or other unpleasant psychological or perceptual changes also will be considered in defining a maximal amplitude threshold for each contact.

7.1.10 Testing Phase Continuation and Stoppage Rules

All subjects will receive a minimum of four months of Rehab + DBS, after which the Testing phase may continue on a month-by-month basis for up to four additional months. Rehab + DBS will continue as long as the subject demonstrates an improvement greater than the minimal detectable change (MDC), as measured by the FMA-UE. MCD will be defined as an average change of 3.2 points[184] during a period of eight weeks where the average rate of change will be calculated using linear regression across the previous three months (i.e. 4 data points). Continuation or stoppage will be determined one month in advance to allow adequate time for scheduling. All outcome measures will be collected once/month as described in §7.7 Testing Phase.

Rehab Follow-up

During this phase, the subject will have their stimulation gradually reduced and subjects will be weaned of continuous DBS. Weaning will occur during the course of one month at weekly visits. In each visit, the power will be reduced by approximately one quarter of the power used at the end of the testing phase until the power is reduced to zero.

During each visit, patients will be asked about the occurrence of adverse events since their last study visit. All adverse events must be documented/reported. Medications will be reviewed and changes will be recorded. Patients will complete primary and secondary outcome measures. During the last visit, a general clinical exam will be performed.

Long-Term Follow-up:

Long-term follow-up continues until the patient reaches the study conclusion at 12 months post implant. Patients will be monitored for adverse events and will be assessed for any continuing changes in any of the primary or secondary outcome measures. No formal rehabilitation therapy is required by the study protocol during long-term follow-up.

Explant

After Rehab Follow-up, subjects will have the DBS system explanted. Before explant, subjects will participate in up to three programing visits to evaluate the effects of DBS stimulation magnitude (amplitude and pulse width) on cortical excitability. Subjects will participate in data collection tasks for up to 8 hours per day. Subject activities will be performed in short segments, broken up across a morning and afternoon session, allowing for frequent rest to accommodate the subject and improve data quality which would be affected by subject fatigue. The programming session will include electrophysiology during active and passive tasks (see §7.3.2). All subjects will return 6 months after explant for a post-explant follow-up to evaluate any adverse events due to the device or therapy.

In the event that treatment (Stim + Rehab) demonstrates benefit but decreases significantly once stimulation is discontinued, a Compassionate Use IDE supplement will be submitted to FDA requesting that subjects may keep their DBS system ‘on’ and continue their DBS therapy while Enspire pursues the next subsequent study. A significant decrease in benefit will be defined as a loss of more than 50% of the motor gains attributed to deep cerebellar stimulation (i.e. gains achieved only after stimulation was activated). In order to continue receiving therapy the subjects will re-consent for this longer-term study.

In the event that a subject does not have the device removed, the system will be turned OFF and the Remote Control and Charger (if applicable) will be returned. Subjects that do not have the device removed will be followed up after 6 months and adverse events will be monitored. Enspire will seek FDA approval for a long-term safety surveillance study (e.g. up to 5 years) for these subjects (i.e. subjects that has the DBS system implanted but turned off). A separate informed consent will be done with this surveillance study.

7.1.11 Explant Procedures

The neurostimulator will be explanted, and prior to DBS lead removal, subjects will have the DBS lead externalized via an extension to allow for study-related local field potential (LFP) recordings from the region of the dentate nucleus. LFPs will be recorded as previously described in §7.1.4 Local Field Potential Recordings. In a second surgery, the DBS lead and extension will be explanted.

Subjects who were implanted before the protocol was modified to include LFP recordings will not be required to have the DBS lead externalized during explant. These subjects will have the option to have the DBS lead externalized or have the system explanted during a single procedure, as originally proposed, with overnight observation in the hospital. The subjects’ decisions will be documented.

Exit of Participation

All subjects who exit from the study shall have the reason for their exit documented. Reasons include: completion of study, lost-to-follow-up, subject withdrawal, discontinue due to adverse event, physician-directed subject withdrawal, and death.

7.1.12 Loss to Follow-Up

Every attempt shall be made to have all subjects complete the study visit schedule. A subject will not be considered lost-to-follow-up unless efforts to obtain compliance are unsuccessful. At a minimum, the effort to obtain follow-up information must include three documented attempts to make contact via telephone or email, and if contact via phone or email is not successful, then a certified letter from the Principal Investigator shall be sent to the subject's last known place of residence.

7.1.13 Subject Withdrawal from Study

All study subjects have the right to withdraw their consent and withdraw participation at any time during the study. Whenever possible, the site staff should get written documentation from the subject that wishes to withdraw consent for future study participation. If the site's staff is unable to obtain written documentation, all known information regarding the subject's withdrawal shall be recorded. If the patient has received the implant at the time of withdrawal from the study the device will be programmed to OFF and zero volts. Explant of the device will be recommended unless there is a medical reason that contraindicates procedure for explanting.

Unscheduled Visits

All unscheduled visits shall be documented and any adverse events reported during an unscheduled visit shall be recorded. Performance of study assessments at unscheduled visits should be done as clinically indicated, and results documented on the appropriate study case report forms.

Protocol Deviations

The Principal Investigator, co-investigators and study staff must avoid all protocol deviations. The investigators should not implement any deviation from, or changes to, the IDE protocol without prior review and documented approval from the IRB of the deviation, except where necessary in an emergency to eliminate an immediate hazard to subjects, or when the change(s) involves only logistical or administrative aspects of the trial (e.g., change of monitor(s), change of telephone number(s)). If such a deviation is due to an emergency, the principal investigator will report the deviation to the Sponsor immediately, but no later than 24 hours after its occurrence. The sponsor of the study will seek FDA approval through an IDE supplement for any changes to the study protocol that might affect the rights, safety or welfare of the subjects or scientific soundness of the study.

8.0 SAFETY REPORTING

Adverse events will be assessed and documented by the investigator at all study visits. The investigational site will provide source documentation to the Sponsor to facilitate review and adjudication of AEs.

The principal investigator or the co-investigator is to report all deaths, life threatening events, and any unanticipated, serious adverse events to the Sponsor within 24 hours for investigation and required reporting to FDA, device manufacturers and IRB within the required timeframe. This includes Unanticipated Adverse Device Effects (UADE). The principal investigator will support the investigation and follow up of any serious adverse events until resolved.

Definitions and Classification

The following definitions are from Good Clinical Practice (ISO 14155:2011) and FDA Code of Federal Regulations for Medical Devices (21 CFR 812.3(s))

8.1.1 Adverse Event (AE)

An AE is any untoward medical occurrence, unintended disease or injury or any untoward clinical signs (including an abnormal laboratory finding) in subjects, users or other persons whether or not related to the investigational medical device.

AE includes all hospitalizations and events related to the investigational device or the comparator. This includes events related to the procedures involved (any procedure in the clinical investigation plan). For users or other persons this is restricted to events related to the investigational medical device.

AE does not include conditions pre-existing to the subject's enrollment. Pre-existing conditions will not be reported as AEs unless the condition has an increased occurrence or intensity.

8.1.2 Serious Adverse Event (SAE)

An SAE is any AE that:[2]

(a) led to a death,
(b) led to a serious deterioration in health that either:
  (1) resulted in a life-threatening illness or injury,
  (2) resulted in a permanent impairment of a body structure or a body function,
  (3) required in-patient hospitalization or prolongation of existing hospitalization,
  (4) resulted in medical or surgical intervention to prevent life threatening illness or injury or permanent impairment to a body structure or a body function,
  (5) resulted in a substantial disruption in ability to conduct normal life functions.
(c) led to fetal distress, fetal death or a congenital abnormality or birth defect.
(d) when the event does not fit the above outcomes, but the event may jeopardize the patient and may require medical or surgical intervention (treatment) to prevent one of the other outcomes.

[2] Updated as per FDA feedback, August 27, 2015, Question 14

SAE does not include in-patient hospitalization for a planned study procedure. Planned study-related in-patient hospitalization is not an SAE.

SAE includes device deficiencies that might have led to a serious adverse event if (a) suitable action had not been taken or (b) intervention had not been made or (c) if circumstances had been less fortunate. These are handled under the SAE reporting system. A planned hospitalization for pre-existing condition, or a procedure required by the Clinical Investigation Plan, without a serious deterioration in health or to prevent life threatening illness or injury or permanent impairment to a body structure or a body function, is not considered to be a serious adverse event.

8.1.3 Device deficiency

A Device Deficiency is any inadequacy of a medical device related to its identity, quality, durability, reliability, safety or performance, such as malfunction, misuse or use error and inadequate labelling. A Device Deficiency occurs in any case where the device does not perform in its intended function and when used in accordance with the device labelling.

8.1.4 Adverse Device Effect (ADE)

An ADE is an AE related to the use of an investigational medical device.

ADE includes any adverse event resulting from insufficiencies or inadequacies in the instructions for use, the deployment, the implantation, the installation, the operation, or any malfunction of the investigational medical device. This includes any event that is a result of a use error or intentional misuse.

8.1.5 Serious Adverse Device Effect (SADE)

An SADE is an ADE that has resulted in any of the consequences characteristic of a serious adverse event.

8.1.6 Unanticipated (Serious) Adverse Device Effect (UADE)

A UADE is a SADE which by its nature, incidence, severity or outcome has not been identified in the protocol or in the investigational device manuals.

Per 812.3(s) this includes any SAE caused by, or associated with, a device, if that SAE was not previously identified in nature, severity, or degree of incidence in the investigational plan or application (including a supplementary plan or application), or any other unanticipated serious problem associated with a device that relates to the rights, safety, or welfare of subjects.

8.1.7 *Relatedness to DBS Device*

Implantation procedure-, device-, and stimulation-related adverse events are defined as any adverse events that are considered by the investigator and evaluated by the Sponsor to be related to the implant procedure, DBS device, or stimulation, with consideration of the strength of the temporal relationship to the implant procedure, onset or cessation of stimulation and the presence or performance of the DBS

system, and the presence or absence of an alternative etiology such as the underlying disease, co-morbidities, and concomitant drugs/treatments.

The probability that a particular AE is related to the procedure, device or stimulation shall be coded on the AE case report form as *unrelated*, *possibly related* and *related*. Probability ratings are based on the temporal relationship to intervention, the likelihood that the symptom could have been produced by the participant's clinical state, the environment or other interventions, whether the participant's symptom course follows a known pattern of response to the intervention, and the experience and judgment of the investigators.

Device Deficiencies

All Device Deficiency shall be documented and reported in the IDE Annual report, it will include those that require explanation of the device. If a Device Deficiency results in an adverse event for the subject, including those that require explanation of the device, will be reported as an adverse event and will be classified appropriately. Device Deficiency that do not result in an adverse event for the subject do not need to be recorded as an AE, as they are not considered an AE.

If a Device Deficiency occurs with the Vercise system the investigators will follow the procedures outlined in the device labeling regarding return of the product to Boston Scientific, Inc. This applies to deficiencies that are identified prior to implantation as well as those that that require explanation of the device.

Deaths

Each subject death shall be reported. A copy of the death certificate and a copy of the autopsy report, if available, should be obtained. Any other source documents relied upon to make a determination of death classification and cause of death will also be filed with the subject's study documents. Data Management

In addition to case report forms, copies of the following source documents verifying any study assessments that are performed are required to be maintained in the subjects' study records and be available for the study monitor to review as appropriate. These may include:

- Laboratory test results
- Imaging results
- Electrophysiological testing results

9.0 DATA MANAGEMENT

Completion of Case Report Forms

To ensure data quality and completeness, all required study data shall be recorded on case report forms (CRFs). The Principal Investigator or appointed designee is responsible for capturing the information or transferring the information from

source documents onto the CRFs. The PI, Co-Investigator, or appointed designee must review case report forms, and validate/sign the completed CRFs.

Data Review

Study monitors will review the information documented in the CRFs and verify the information recorded is consistent with medical records or other source documents. Errors or incomplete entries will be rectified by study staff and principal investigator for correction and preventive measures.

Additional information and source documentation will be reviewed during monitoring visits or be submitted to the Sponsor for evaluation, for example, for adjudication of adverse events by the DMC or when device deficiencies are reported.

All clinical centers will be monitored periodically by the Sponsor for protocol adherence, accuracy of CRFs, and compliance to applicable regulations.

Data Analysis Plan

All safety and outcome assessments will be summarized with appropriate descriptive statistics. Non-continuous variables will be summarized by percentages and frequency distributions. Data will be tabulated for all enrolled patients. Exploratory statistical analyses will be performed to identify potential relationships between the treatment and the outcome variables.

Adverse event data will be reported and tabulated according to the severity and likely relationship with the device, procedure or stimulation.

Events will be considered to be permanent if the adverse event does not resolve despite medical management, changes in device programming, or stopping treatment**.**

10.0 STUDY ADMINISTRATION

Data Monitoring Committee

An independent Data Monitoring Committee (DMC) will be established to provide additional, independent oversight to enhance safety of study participants and ensure subjects' rights and welfare. The DMC will be responsible for safeguarding the interests of trial participants by assessing the safety and efficacy of the interventions during the trial, and for monitoring the overall conduct of the clinical trial.

The DMC will consist of three experts with relevant clinical specialties. This will include at least one physician that practices neurology or physical medicine and rehabilitation and specialize in post stroke care and at least one neurosurgeon experienced in DBS therapy. They should also be familiar with FDA regulated clinical research with investigational implantable devices and/or investigational

drugs. Experience with serving on another DMC, DSMB and IRB are desirable. These three experts are the core members with voting responsibility.

Other experts such as physical therapist, rehabilitation professionals, and medical ethicists may be invited to participate on an as needed basis as additional advisors to evaluate complex and difficult issues.

10.1.1 DMC Responsibilities

The DMC is governed by its Charter[3]. The DMC will be responsible for safeguarding the interests of trial participants by assessing the safety of the interventions during the trial, and for monitoring the overall conduct of the clinical trial. The DMC is responsible for defining its deliberative processes, including event triggers that would call for an unscheduled review, stopping guidelines, and voting procedures prior to initiating any data review.

10.1.2 Monitoring for Safety

The DMC reviews safety data from the study and will make recommendations, if appropriate.

One member of the DMC will be assigned as an independent reviewer of all serious adverse events. The independent reviewer will be notified within 2 business days of becoming aware of an adverse event to assess whether the event requires review by the full board. If full board review is required, an unscheduled DMC board meeting will be held via teleconference within 5 business days to determine if enrolment should be halted until the event has been satisfactorily resolved. Specific rules will be determined by the DMC during the first organizational meeting.

10.1.3 DMC Meetings

Scheduled Meetings
The frequency of scheduled meetings depends on subject enrollment and adverse event rates. Initially, the DMC will convene after each subject has completed 1-month post implantation follow-up. This will allow the DMC to review the safety data routinely throughout the study at various time points such as: implantation, DBS+TMS stimulation, and chronic DBS stimulation. After the first six subjects have been implanted, the frequency of the DMC meetings will occur 1-month after every third subject is implanted.

The DMC will review safety data within 10 days of availability of the safety dataset once the subject has completed one (1) month post implant. The objective of these meetings is to evaluate safety data for the first subjects and make recommendations to the Sponsor.

[3] See Section 8.4 of this IDE.

The DMC will review data related to: implant and other surgical safety; stimulation (therapy) safety for at least one-month data of "DBS + rehab" phase for each subject, and any safety information collected, whether it is related to the device/therapy or not.

Unscheduled Meeting to Evaluate Important and Urgent Safety Data: If significant and urgent safety data emerges, e.g. unanticipated serious device or treatment related adverse events, the DMC will convene a meeting to evaluate the safety within 5 days.

10.1.4 Making Recommendations

The DMC may recommend one of the following actions to the study Sponsor; note this is not an exhaustive list:

- May expand the study beyond 5 subjects and enroll additional subjects with the current inclusion/exclusion criteria.
- Subject's inclusion criteria can be expanded to subjects with less severe symptoms, i.e. with FMA-UE scores higher than 20.
- Modify the study protocol. Modifications may include, but are not limited to, changes in inclusion/exclusion criteria, frequency of visits or safety monitoring, alterations in study procedures, adjustments in sample size, changes in duration of observation and follow up.
- Discontinue the study (with provisions for orderly discontinuation in accord with good medical practice) due to safety concerns.

10.1.5 DMC Decision Guidelines

General Considerations: The trial is a feasibility trial where the primary focus is on patient safety. Therefore, DMC recommendations will be made based on clinical judgment rather than statistical results or rules.

Stopping for significant safety risk:[4] Any recommendation to terminate the trial due to significant safety risk will be made on the basis of sound medical judgment. For the first cohort of 5 subjects, upon review and deliberation by the DMC, the DMC may immediately halt enrollment/implant of additional patient(s) for the clinical trial if there is no satisfactory explanation or resolution of the reported Serious Adverse Events(s).

If one or more SAEs with un-explained etiology and lack of satisfactory resolution of the SAE, the DMC may recommend terminating the clinical trial as designed. The DMC may also recommend making changes to the protocol to improve the safety of the clinical trial.

For this early phase safety study with no control arm, and especially for the first cohort of subjects, no or little efficacy would be expected and therefore a benefit-risk analysis may not be appropriate. The DMC should take into consideration the potential scientific

[4] Stopping rule as per FDA feedback, August 27, 2015, Question 14

gain from continuing the study evaluated in the context of ethical considerations for ensuring subjects' rights and welfare.

Study Registration.

The study will be registered with www.clinicaltrials.gov as recommended by the International Committee of Medical Journal Editors.[152]

11.0 SCIENTIFIC SOUNDNESS

It is expected that the results of this early feasibility study will provide adequate information on which to base decisions regarding the need for, and the design and conduct of future trials of deep brain stimulation of the cerebellar dentate nucleus for treatment of post-stroke motor impairment.

The rationale for the treatment suggests that the potential benefits outweigh the risks of the treatment for the population under study. Bias in patient selection is minimized by clearly defined enrollment criteria, and procedures to track and report patient recruitment and screening activities. Outcome assessment bias and measurement error is minimized by selecting qualified professionals who are experienced in the diagnosis, assessment and treatment of individuals with post-stroke upper extremity hemiparesis to perform the outcome evaluations, use of standard assessment tools that are known to be valid and reliable.

The sample size selected for this early feasibility study, if approved by FDA to expand to a total of 12 subjects will provide safety information and estimates of the study outcome assessments.

As a feasibility study, the results will be carefully interpreted in light of the postulated mechanism of action of stimulation to improve motor function, the expected risks and benefits of cerebellar dentate nucleus stimulation, and the risks and benefits of alternative treatments.

12.0 RISK/BENEFIT ANALYSIS

Potential Benefits

12.1.1 Chronic Electrical Stimulation of the Dentate Nucleus

Since this is an early feasibility study, very limited human data is available to-date. As of May 1, 2017, one subject has been implanted with the DBS system and experienced chronic stimulation of the Dentate Nucleus for approximately 2 weeks. As expected, no specific therapeutic benefit has yet been measured in the first subject, however, the subject self-reported a reduction in spasticity from acute stimulation (< 2 hours) during device programming.

There are no guaranteed benefits from participation in this study; however, it is possible that electrical stimulation of the dentate nucleus may reduce upper extremity motor impairment and improve motor functioning. Subjects may achieve a greater degree of functional use of their arm, and as a result, may increase their

ability to independently perform activities of daily living and enhance their social and community participation.

12.1.2 Advancing Scientific Knowledge and Optimizing Stroke Therapy

Temporary externalization of the DBS lead, for approximately one week withinpatient care at the Cleveland Clinic, may help advance scientific knowledge. The proposed gain in scientific knowledge are aimed at improving the future design of large clinical trials for DBS of the dentate nucleolus, informing the next generation of DBS devices (e.g. feedback mechanisms), and providing "personalized" stimulation parameters/algorithms that will be custom tailored for augmenting post-stroke rehabilitation. Characterizing local field potential (LFP) changes in the area of the dentate nucleolus may:

- Inform lead localization and/or therapeutic contact selection
- Improve understanding of the role of oscillatory activity in the cerebellar dentate nucleolus in motor and cognitive processes.
- Inform the development of physiologically-driven classifier(s) for closed-loop DBS of the dentate nucleolus and other novel treatment strategies.

Potential Risks of Electrical Stimulation of the Dentate Nucleus

Risks associated with the Vercise DBS system and implant procedure are summarized in the device labeling.[153] In addition, adverse events and complications associated with deep brain stimulation have been reported in the peer-reviewed literature.

The risks associated with participation in this study of deep brain electrical stimulation of the dentate nucleus for the treatment of chronic, post-stroke hemiparesis are classified into three categories: risks associated with the device implant procedure, risks associated with the DBS system and components of the system, and risks associated with the stimulation therapy.

12.1.3 Risks Associated with Vercise DBS System Implantation Procedure

Risks associated with the procedure for implantation of a deep brain stimulation system include all possible risks related to any neurosurgical intervention. In essence, any organ system is at risk perioperatively, particularly in a person with presumed vasculopathy. These risks include (but are not limited to):

- Blood clot forming, for example in the veins of the legs, and thromboembolism. (i.e. pulmonary embolism).
- Hospital acquired conditions such as pneumonia.
- Blood clot or air in the blood stream, which can block blood flow to parts of the lungs or other tissue
- Perioperative medical complications including cardiovascular complications (i.e. myocardial infarction, arrhythmias), kidney complications (including renal insufficiency), liver problems (including failure), pulmonary problems (including

respiratory failure), abdominal problems (including acute abdominal problems, peritonitis), other medical problems (including potentially serious or fatal).
- Cerebral spinal fluid (CSF) leaking outside the skull or collecting in the skull abnormally.
- Confusion or problems with attention, thinking, or memory, speech or any other cognitive or behavioral problem, including psychosis and hallucination.
- Death
- Infection, including infection of the central nervous system
- erosion / exposure or the hardware in which case the device may need to be partially or completely removed
- Injury to areas next to the implant, such as blood vessels, nerves, the chest wall, the lungs, pleural space and the brain
- Neurosurgery/anesthesia risks, including unsuccessful implant
- Pain, headache, loss of sensation or discomfort
- Seizures
- Stroke resulting in temporary or permanent problems, such as hemiparesis or paralysis, inability to swallow, cranial nerve deficits or inability or difficulty speaking
- Intracranial hemorrhages, resulting in transient or permanent neurological problems including paralysis, inability to swallow, cranial nerve deficits or inability or difficulty speaking.
- Brain edema resulting in transient or permanent neurological problems including paralysis, inability to swallow, cranial nerve deficits or inability or difficulty speaking.
- Swelling (seroma), bruising or hematomas
- Movement disturbances,
- Double vision, blindness or other vision problems
- Intraparenchymal cyst

Table 4 summarizes the most common risks known to be associated with implantation of deep brain stimulation systems for approved indications reported in the literature.

Table 4. Risks of Deep Brain Stimulation Implant Procedure

Risk	Expected Rate	Source
Hemorrhage	2.25 to 4%	Binder, 2003<br>Terao, 2003<br>Binder, 2005<br>Deogaonkar, 2007
Infection or erosion	3.8 to 8.1%	Umemura, 2003<br>Sillay, 2008<br>Hamani, 2006<br>Temel, 2004<br>Deogaonkar, 2007

Hardware complications	5 to 26%	Lyons, 2004[154]<br>Hamani, 2006<br>Voges, 2006

Risks of a DBS implant procedure of special concern in a deep cerebellar target are: bleeding, infarct, stroke, cerebrospinal fluid leak, and air embolism. These risks have been minimized by the proposed implant procedures. The patient will be implanted in the prone position which is known to reduce the risk of air embolism compared to the sitting position. Careful direct brain targeting will be accomplished with state of the art stereotactic systems

Furthermore, only one functional neurosurgeon (study PI) will be performing the implant procedures. The Cleveland Clinic Foundation has an experienced DBS team, and has performed hundreds of deep brain stimulation procedures in a variety of brain target areas for a variety of indications. CCF has rigorous operative procedures, and perioperative procedures care to ensure complications of surgery and DBS stimulation are minimized.

12.1.4 Risks Associated with Temporary Percutaneous Externalization of DBS Lead

Externalization of the DBS leads for recording local field potentials (LFPs) may increase the risk of post-operative infections. However, the rate of these risks is expected to be similar to the implantation of deep brain stimulation (DBS) systems for approved indications.

Dr. Machado (principal investigator) has over ten years of experience with DBS implantation procedures for both approved (i.e., Parkinson's disease, essential tremor, dystonia, and obsessive-compulsive disorder) and investigational (e.g. traumatic brain injury, major depression, pain) indications and has performed well over 500 system implants to date. Most of these implants (roughly 90%) are 2-staged with the neurostimulator and DBS lead implanted in separate procedures. The rate of infection for DBS procedures at the Cleveland Clinic is 1-2% for the last 5 years. In addition, Dr. Machado has performed more than 20 procedures with externalized DBS leads and there has never been an infection in a patient who underwent an externalized procedure.

From other experience, Rosa and colleagues[174] explicitly evaluated the risk of infection from lead externalization during DBS procedures. A total of 105 patients underwent lead externalization as part of a 2-staged bilateral DBS implant for the treatment of Parkinson disease (PD). The incidence of infections in patients who underwent lead externalization was 2.8%. Furthermore, comparing lead externalization with or without LFP recordings did not statistically increase the risk of infection. By comparison, this rate of infection is consistent with the postoperative infectious risk reported in the literature. Specifically, the same study tabulated the mean incidence of infection from DBS for PD as 4.3% (n=2848). Similarly, from Medtronic's 2015 Product Performance Report, the incidence of infection for DBS across all indications was 3.2% (n=1580).

Comparatively, epileptiform activity is routinely monitored by extraoperative invasive subdural grid electrodes and depth electrodes. The incidence of neurological infection for grid electrodes was 2.3% (n=1342 individuals) with a recording duration of 5 to 17 days[175]. The incidence of infection for depth electrodes was 1.8% (n=491 individuals) with a mean recording duration of 14.8 days[176]. Similarly, the risk for intracranial hemorrhage was 4.0% for subdural recordings[175] and intracranial hematoma was 0.8% for depth electrode recordings[176].

In addition to infection, updated risk analysis identified the possibility of lead migration, unintended electrical activity (shock), and subject fatigue could occur but the likelihood of these risks is low.

12.1.5 Risks Associated with Vercise DBS System

Risks associated with the deep brain stimulation system include:

- Allergic or immune system response to implanted materials
- Failure or malfunction of any part of the device, including but not limited to: Battery leakage, battery failure, lead or extension breakage, hardware malfunctions, problems recharging the device, loose connections, electrical shorts or open circuits, and lead insulation breaches, whether or not these problems require device removal and/or replacement
- Implant site complications such as pain, poor healing, redness, warmth, swelling or wound reopening
- Implanted device components (stimulator, lead, or extension) may move from original implanted location or wear through the skin, which may lead to the need for additional surgery
- Fibrosis around the lead extension including tightening, tethering and bowstringing.
- Infection
- Interference from external electromagnetic sources
- Loss of adequate stimulation
- Pain, headache or discomfort.
- Skin irritation or burns at the stimulator site
- Chemical burns may result if the Vercise Stimulator housing is ruptured or pierced and your tissue is exposed to battery chemicals.
- Stiffness in muscles or with joint movement
- Swelling, including fluid collecting around the device

The stimulator lead may need to be re-positioned surgically if the clinical benefit has not been optimized or if programming the device causes intolerable side effects or side effects that cannot be avoided with reprogramming. Every effort is made to minimize the risks of the lead repositioning procedure. The complications of lead repositioning are similar to the risks associated with the initial procedure.

<u>Risks Associated with the DBS System MRI Contraindication</u>

Risk of Recurrent Stroke in Patients with a Prior Ischemic Stroke

MRI testing following DBS system implant is partially contraindicated. Based on the Cleveland Clinic experience, MRI sequences considered desirable in the investigation of suspected acute strokes will not be considered safe for these patients[155, 156]. Given the data on the risk of a recurrent stroke between 1 and 5 years after an initial ischemic stroke it is conservatively estimated that 1 patient in the study may have a recurrent stroke during the study (~12 x 10%). This estimate is based on the following review of the literature on the risk of recurrent stroke (Table 5).

Table 5. Literature Review on Risk of Recurrent Stroke Post Ischemic Stroke

Source	Rate
Lehigh Valley Recurrent Stroke Study[157]	12% with average follow-up of 24 months
Perth Community Stroke Study[158]	12.5% @ 1 year.<br>13.4% @ 2 years<br>17.7% @ 3 years<br>22.4% @ 5 years
Hillen et al[159]	total anterior circulation infarcts (TACI)<br>5.8% @ 1 year<br>12.8% @ 3 years<br>21.5% @ 5 years<br>partial anterior circulation infarcts (PACI)<br>9.3% @ 1 year<br>16.3% @ 3 years<br>17.6% @ 5 years<br>posterior circulation infarcts (POCI)<br>9.5% @ 1 year<br>15.5% @ 3 years<br>15,5% @ 5 years
Brown et al[31]	11.8% @ 2 years
Petty et al[160]	atherosclerotic strokes with stenosis<br>24.4% @ 1 year<br>29.3% @ 2 years<br>40.2% at 5 years<br>cardio-embolic strokes<br>13.7% @ 1 year<br>16.8% @ 2 years<br>31.7% at 5 years<br>lacunar strokes<br>7.1% @ 1 year<br>11.6% @ 2 years<br>24.8% at 5 years<br>ischemic, unknown cause<br>13.2% @ 1 year<br>20.6% @ 2 years<br>33.2% at 5 years

Clinical Consequences of MRI Contraindication for Stroke Patients

The most important goal in the acute setting is to determine whether the patient is a candidate for thrombolytic therapy (i.e. tPA) or other acute interventions in a timely fashion.

The risk associated with having a contraindication for MRI for the patients in the proposed study is that an accurate diagnosis might be compromised because only CTs but not MRIs will be available to the patient. Available thrombolytic therapies or other interventions may be delayed or not provided.[5]

Risks Associated with the DBS System and CT

The following are potential adverse events that can occur with CT scans after device implantation:

- involuntary movements (Dyskinesia)
- pain
- shocks
- dizziness
- shaking
- loss of therapy
- discomfort
- overstimulation
- tingling sensation
- burning sensation
- headache and
- spontaneously turning on or off of the device during or immediately after CT scans

Risks Associated with TMS

- headache or neck pain
- undesirable tingling or pain
- ringing in the ear and temporary shifts in your ability to determine the pitch or loudness of sounds.
- dental pain during TMS
- may have a seizure induced by TMS
- memory or attention may be affected for a short period of time (a few minutes)
- TMS on the back of the head may cause contractions of muscles in the face and neck and may be uncomfortable or painful.

12.1.6 Risks Associated with Deep Brain Stimulation Therapy

Stimulation related adverse events are expected in 100% of the patients. During programming of the DBS systems, the amplitude will be increased in stepwise fashion until a side effect is noted or until the charge density limit is reached. Hence, the investigators will be deliberately seeking to find the threshold for adverse events in each individual. Chronic, irreversible stimulation related adverse events are rare. Most stimulation side effects are reversible and are corrected by reprogramming the neurostimulator or turning the neurostimulator off. A variety of

[5] Revised as per FDA feedback, August 16, 2015, Question 1i

stimulation-related adverse events have been reported for approved DBS stimulation brain studies, including:

- Confusion or problems with attention, thinking, or memory, speech or any other cognitive or behavioral problem. Pain, headache or discomfort
- Psychiatric disturbances such as anxiety, depression, lessened interest or emotion, mania, hypomania, emotional sensitivity, sleep problems, suicide, or suicidal thoughts or attempts, psychosis, hallucinations.
- Seizures, including epilepsy
- Sensory changes
- Speech or language problems, difficulty swallowing, as well as complications such as aspiration pneumonia
- Systemic effects such as rapid heartbeat, sweating, fever, dizziness, changes in kidney function, difficulty passing urine, sexual effects, nausea, difficulty having bowel movements, bloating
- Weakness, paralysis, muscle contractions, muscle spasms, shaking, restlessness, or problems with movement, walking, coordination, or body positioning, and falls or injuries resulting from these problems
- Undesirable sensations such as tingling, nausea, or pain
- Visual problems, eyelid or eye movement difficulties or other eye-related symptoms
- Problems related to the function of any cranial nerve.
- Weight changes
- High levels of stimulation may damage brain tissue, including permanent damage.
- Effectiveness or comfort of stimulation changing over time. Temporary worsening of symptoms when stimulation is stopped, or "rebound."

In addition to these effects, based on the anatomy of the region to be stimulated (dentate nucleus), we expect that possible stimulation related side effects will be:

- Motor changes such as worsening of coordination, twitching or pulling, paresthesias, changes in balance or vertigo, modulation of mood[161]
- Laughing or relaxation, behavioral problems.[162]
- Reduction in tonus
- Oculomotor side effects[86, 87]
- Changes in cognitive function may also occur.

Given the early-phase nature of this study and the pooled literature experience from deep brain stimulation of the dentate nucleus, we currently cannot attribute specific rates to these potential stimulation related adverse events.

Risk Management

12.1.7 Risk Minimization through Study Design

To minimize risk, this early feasibility study will initially utilize staggered enrolment to limit risk exposure. Initially, 2 subjects will be implanted with a staggered design

that specifies a new subject can be enrolled only after the previous subject has completed 1-month post-implant with reporting to the FDA. Staggering subject enrolment ensures that subsequent DBS implants, or other events such a device activation, are spaced several months apart from one subject to next subject.

Eligibility criteria that exclude subjects who are at higher risk for experiencing an anticipated Adverse Event have been selected in order to reduce the potential risks to subjects that participate in this study. The potential risk of further impairing function is greater in a moderately impaired subject compared to a more severely impaired individual. Furthermore, while subjects with more impairment may have less chance for benefit, it does not necessarily mean that they have no chance for any benefit from the therapy. Since this feasibility study is focused primarily on safety, the study will initially enroll more severely impaired subjects to provide initial safety information as described in §1.1.1Staged above. Specifically, only severely impaired subjects with a score of ≤ 20 on the UE Fugl-Meyer be initially enrolled in this study.

12.1.8 Risk Minimization of DBS Lead Implanted in the Dentate Nucleolus

The risks associated with the DBS implant procedure have been minimized by the proposed implant procedures. The patient will be implanted in the prone or lateral position, which is known to reduce the risk of air embolism compared to the sitting position. Careful direct brain targeting will be accomplished with state of the art stereotactic systems and supplemented with intraoperative tests to minimize the chance of missing the target. The risk of CSF leak is minimized by careful surgical technique and use of fibrin glue over the bur hole intraoperatively. Furthermore, only one functional neurosurgeon (study PI) will be performing the procedures. The Cleveland Clinic Foundation (CCF) has an experienced DBS team, and has performed over 1000 deep brain stimulation procedures in a variety of brain target areas for a variety of indications. CCF has rigorous operative procedures, and procedures for postoperative care to ensure complications of surgery and DBS stimulation are minimized.

Hardware externalization may increase the risk of post-operative infection. Several precautions will be taken to minimize the risks of externalization.

- The subject will remain hospitalized throughout the period of device externalization.
- During lead implant, the extension used for the externalized trial will be tunneled to a percutaneous exit site that is distant from the site where the neurostimulator will be implanted.
- During the neurostimulator implant procedure, the entire extension will be replaced. First the extension will be disconnected from the DBS lead (clean site) and then pulled away through the percutaneous exit site in order not to bring any contaminants towards the implanted hardware. The percutaneous exit site will remain outside the prepped and draped area and only closed after the end of the surgery.

- The subject will be prescribed oral antibiotics during the externalization period and for one day after implantation of the neurostimulator.

Additional clinical and research support will be available for this study at the Clinical Research Unit (CRU) of the Cleveland Clinic which provides:

- Provides clinical nursing support to monitor and safeguard against any risks to patients during research procedures.
- Comprises of a core group of clinical research administrators who help ensure the most efficient and effective ways for researchers and clinical trial participants to interact.
- Coordinates and provides a culturally sensitive and welcoming clinical environment for volunteer research participants and highly trained personnel to carry out research protocols in the hospital and in outpatient medical centers.

12.1.9 Safety Monitoring and Oversite

The close safety reporting, monitoring by the investigator and sponsor, and oversight by the IRB and DMC in this first-in-human trial described in Sections 8, 9 and 10 of this protocol provide further risk mitigation measures to protect the safety, rights and welfare of enrolled patients.

In addition to DMC and local IRB oversight, this study also requires reporting to the FDA after each subject completes 1-month post-implant. Furthermore, aspects of the study, including local field potential recording, include additional oversights established as part of the NIH funded research. The NIH funded research has established a Scientific Steering Group (SSG). The SSG includes all PIs and additional representatives from collaborating institutes. Dr. Stephen Jones, the Vice-Chairman of the Imaging Institute, will conduct the MRI studies, including 7T MRI, tailored to guide deep cerebellar electrode implantations and basis for computational modelling. Dr. Paul Ford, the head of Bioethics for neurological care at Cleveland Clinic has agreed to participate when requested and will remain updated on any concerns related to the conduct of first-in-man clinical research. Per the NIH grant application, the SSG holds teleconference every two weeks. The SSG will govern the overall activities of the NIH study, make decisions on scientific direction, intellectual property development, and adjudicate conflicts should they arise.

12.1.10 SARS-CoV-2 Mitigation

To mitigate exposure to SARS-CoV-2 therapy visits will occur either in-clinic or in an at-home setting via a Cleveland Clinic approved telehealth platform as described in section 7.5 Rehabilitation.

Subjects with scheduling issues due to COVID-19 related disturbances (such as havening missed visits because they are ill, need to self-quarantine, have issue with campus accessibility, or have concerns due to exposure or possible exposure to SARS-CoV-2) may have their study visit schedule shifted of up to 2 weeks to ensure that they complete all subsequent visits as planned without omission. As such, the study staff will maintain continued communication with the subjects to

report safety issues. In the evet of a COVID related schedule shift, the number of rehab therapy sessions (with their therapist) will be adjusted to maintain a consistent therapy dose of approximately 8 sessions between monthly assessments throughout the study. In the event of a schedule shift that exceeds two weeks due to a prolonged COVID schedule disruption, such as hospitalization, the Principal Investigator will determine if the monthly assessments should be omitted.

Communication of Risks Benefit

The investigator and/or the study coordinator will explain the nature of the study including study related risks. The investigator and/or study coordinator will be available to answer all questions the subject may have about the study. In addition, Dr. Paul Ford (Cleveland Clinic Bioethics) or one of his designees will participate in the study as a consent monitor to verify the subject made informed consent including understanding of the research process, risks, burdens, uncertainty, as well as the novel nature of this research.

Consideration of Patient Preference Information

This study enrolls subjects that have severe functional limitation, as per their FMA-UA, whose functional recovery has plateaued and have a limited chance of additional improvement. Study screening requires the subjects to have minimal response to less invasive treatment (i.e. rehab) prior to being eligible for DBS implant which is corroborated through study specific period of eligibility-rehab.

Assessment of Uncertainty

Since this an early feasibility study, it is not possible to provide an accurate assessment of uncertainty of stimulation of the dentate nucleolus. The experience of the first implanted patient (with 4 months of post-implant data, to date) and the historical performance at the Cleveland Clinic and of the principal investigator (Dr. Machado, an experienced neurosurgeon) provide some historical data on the likely outcome of enrolled patients as far as surgical risks are concerned.

Risk Benefit Analysis

While we believe that the risk for significant injury or death due DBS system implantation and electrical stimulation of the dentate nucleus will be equivalent to the risks of DBS implantation in other brain targets, these risks have yet to be adequately and fully quantified in the patient population under study.

The lack of serious AE in the first patient implanted provide some human experience with this procedure. With the risks identified in previous subsections and the extensive risk mitigation strategies documented in section 0 Risk Management, we believe that the risk mitigation strategies reduce the known residual risks as much as possible and would result in favorable IDE benefit-risk for this protocol. The indirect benefit is potentially gaining scientific knowledge which

may contribute to the development of a better device and personalized stimulation parameters to improve patients' lives with an optimized therapy

The proposed IDE feasibility study includes many efforts to minimize the potential for risk, and is based on a well-defined clinical rationale. Stimulation of the dentate is expected to chronically activate the dentate-thalamo-cortical pathway and, consequently, enhance cortical excitability and facilitate plasticity, cortical reorganization and motor recovery of upper extremity hemiparesis.

13.0 MONITORING PROCEDURES

Monitoring Procedures

The Sponsor will have a dedicated clinical study monitor assigned to this study. He/she will be responsible for ensuring that appropriate monitoring of the study is performed.

The clinical study monitor will review data in submitted Case Report Forms as they are generated for accuracy and completeness as per data review process. Monitors will conduct visits to the Cleveland Clinic to ensure accuracy of data, timeliness of data submissions, adequate subject enrollment, investigational device accountability, compliance with applicable laws and regulations, compliance with the protocol, compliance with the signed investigator agreement, and compliance with IRB conditions and guidelines. Any non-compliance with these items will be discussed with the PI who will be responsible for ensuring that the non-compliance is adequately addressed with relevant corrective and preventative actions.

Because this is a first-in-human study, the clinical monitor plans to:

- Conduct training of all study personnel regarding the protocol, GCP compliance and study assessment procedures.
- Be present at all implant, TMS testing, and baseline FMA-UE assessment
- Be at study sites frequently to review documentation of informed consent, adherence to inclusion/exclusion criteria, case report forms, and adherence to study procedures, to assess compliance with the protocol and investigational product accountability

For the first subject, the clinical monitor plans to be at the clinical site for all monthly visits to verify accuracy and completeness of data from the CRF (as compared to source documents). In addition, safety data for the first implanted subject will be reported to the FDA early and frequent intervals.

13.1.1 Monitoring Reports

After each monitoring visit, the monitor will compile and send to the PI a report summarizing the monitoring visit. The PI will be responsible for ensuring that any follow-up actions needed to resolve issues are completed in an accurate and timely manner and corrective and preventative actions are implemented.

Final Site Visit

A final visit will be conducted at the close of the study. The purpose of the final visit is to collect all outstanding study data documents, ensure that the principal investigator's files are accurate and complete, review record retention requirements, and ensure that all applicable requirements are met for the study.

14.0 COMPLIANCE

Statement of Compliance

This study will be conducted according to Good Clinical Practice (GCP) regulations and guidance issued by the Food and Drug Administration (FDA) which are included in the following parts of the FDA Code of Federal Regulations (CFR):

- 21 CFR Part 50: Protection of Human Subjects,
- 21 CFR Part 54: Financial Disclosure
- 21 CFR Part 56: Institutional Review Boards,
- 21 CFR Part 812: Investigational Device Exemptions

The purpose of these regulations is to define the standards and principles for the proper conduct of clinical studies. The ethical standards defined within GCP are intended to ensure that human subjects are provided with an adequate understanding of the possible risks of their participation in the study, and that they have a free choice to participate or not; the study is conducted with diligence and in conformance with the protocol in such a way as to ensure the integrity of the findings; and the potential benefits of the research justify the risks.

Device Accountability

Information on each DBS device used in the study will be documented. Device accountability for each device provided to the site and for each device used for the study will be maintained by the study site. Only approved investigators will implant devices in subjects participating in this study. During monitoring visits the study monitor will evaluate investigational device accountability. Any unused device will be returned to the Sponsor.

Sponsor Responsibilities

The sponsor is responsible for the following:

- ensuring the study is reviewed and approved by the FDA and that the study is compliant with the IDE regulations (21 CRF 812),
- ensuring the investigative center obtains IRB approval prior to initiating the study,
- selecting qualified investigators,
- obtaining a signed investigator's agreement,
- providing investigators with the information and training they need to properly conduct the study,

- ensuring patient informed consent is obtained,
- ensuring proper monitoring of the study,
- ensuring that the study is conducted according to the clinical protocol,
- ensuring that the investigation treatment is made available only to qualified investigators participating in the study,
- ensuring no changes that effect the scientific soundness of the study or the rights safety and welfare of the subjects are made to the investigational plan without prior FDA and IRB approval,
- ensuring that regulatory agencies and all participating investigators are properly informed of significant new information regarding adverse effects or risks associated with the device being studied.
- ensure that adverse events are reported to FDA and IRB within the required timeframe.

14.1.1 Sponsor Records

The sponsor must maintain accurate, complete and current records relating to the study. These records include:

- Correspondence with another sponsor, study monitors, investigators, an IRB and FDA.
- Records of investigational device shipment and disposition
- Signed investigator agreements and financial disclosure if required under CFR 812.43(c)(5)
- Adverse device effects (whether anticipated or unanticipated) and complaints

14.1.2 Sponsor Reports

Refer to Table 6 for a tabular listing of sponsor reporting requirements. The sponsor must prepare and submit the following reports:

- Results of evaluation of any reported unanticipated adverse device effects (UADEs) to FDA, all IRBs, and investigators within 10 working days after notification by the investigator,
- current investigator list to FDA every 6 months,
- an annual progress report to FDA and IRBs,
- any withdrawals of IRB approval to FDA, all IRBs, and investigators, within 5 working days after receipt of notice of withdrawal of IRB approval,
- any withdrawal of FDA approval to the IRB, and investigators, within 5 working days after receipt of notice of withdrawal of FDA approval,
- any device withdrawals or recalls to FDA and IRB within 30 working days after the request is made to an investigator,
- Emergency protocol deviations to FDA within 5 working days after receipt of notice of such emergency use,
- Use of the investigational device or treatment without obtaining informed consent to FDA within 5 working days after sponsor is notified of such use,
- A final report to FDA, IRBs, and investigators within six months of completion or termination of the study.

14.1.3 Sponsor Inspections

The sponsor is required to permit FDA to enter and inspect any establishment where investigational devices are held and where records and results from use of the devices are kept. FDA may also inspect and copy all records relating to an investigation including, in certain circumstances, records which identify subjects. Investigator Responsibilities

Investigator Responsibilities

An investigator is responsible for ensuring that the study is conducted according to the signed investigator agreement, the investigational plan, and applicable FDA regulations; for protecting the rights, safety, and welfare of subjects under the investigator's care; and for the control of devices under investigation. The investigator and clinical site is to allow and support Sponsor audits and inspections by FDA and other regulatory agencies.

14.1.4 Protection of Human Subjects

The investigator must submit the clinical investigational plan and the patient informed consent form to the governing Institutional Review Board (IRB) and obtain written approval from the IRB before enrolling patients in the study. The Investigator is also responsible for fulfilling any conditions of approval imposed by the IRB.

14.1.5 Investigator Records

The principal investigator is responsible for maintaining the following records for a period of two years following the termination or completion of the study. The principle investigator/center must maintain adequate records on all aspects of the study, including the following:

- IRB approvals
- Device disposition
- Informed Consent Forms
- Case Report Forms
- Adverse Event Form and information
- Protocol Deviations
- Correspondence file regarding study
- Subject termination information
- All study-related correspondence with the IRB, another investigator, Sponsor, Study Monitor, and regulatory agencies, including required reports;
- Records of receipt, use, and disposition of the test device system, including receipt dates, lot numbers, and final device disposition;
- Records of each subject’s case history, including information reported on all study-required Case Report Forms (CRFs), evidence of informed consent, all relevant observations of adverse events, the results of diagnostic testing, and the date of each study treatment.

- Copies of the approved clinical investigational plan and any amendments and documentation of any deviations from the clinical investigational plan including documented dates and reasons for each deviation.

14.1.6 Investigator Reports

Required investigator reports are listed in Table 6. The table also displays information regarding to whom this information is to be sent, and the frequency and time constraints around report submission. If applicable laws, regulations, or IRB requirements mandate stricter reporting requirements then those listed, the stricter requirements must be followed.

The Investigator is responsible for the following reports:

- Any serious adverse event (SAE) which has a possibility of being related to the device, stimulation, or study surgery, including all UADEs, are to be reported to the sponsor soon as possible but no later than 24 hours after the event is known to the Investigator,
- Unanticipated adverse device effects (UADEs) to be reported to the IRB within 10 days after the UADE is known to the Investigator),
- Withdrawal of IRB approval (to be reported to the Sponsor and FDA within five working days after the withdrawal of IRB approval is known to the investigator),
- Progress reports (provided to the Sponsor and IRB at regular intervals (as requested by the governing IRB) but no less than yearly),
- Deviations from the protocol. Use of the investigational device without informed consent (to be reported to the IRB within five working days after the use occurs).
- A final report (to be provided to the IRB within six months after termination or completion of the investigation).
- Other study-related reports (upon request by a reviewing IRB, study Clinical Events Committee, and/or the FDA).

Immediate reporting of SAEs which have a possibility of being device related is necessary to allow for further investigating of the SAE by the Sponsor and DMC when necessary to determine whether the event was caused by the device, stimulation, or study surgery.

14.1.7 Investigative Center Inspections

Regulatory agency may audit the investigational center. The purpose of an audit is to assess the accuracy, adequacy and consistency of the study records and subject data and to assess adherence to the procedures described in this clinical investigational plan. A typical audit visit will include the following:

- upon arrival, an interview with the investigator and study personnel,
- a tour of the facility,
- a review of the study records,
- a review of the case report forms and source documents,
- at the conclusion of the audit, a discussion of any key audit observations.

Table 6. Investigator and Sponsor Reporting Responsibilities

Type of Report	Principle Investigator/Sponsor Reporting Responsibilities			
	Report Prepared by Investigators For	Reporting Time Frame	Report Prepared by Sponsor For	Reporting Time Frame
Serious Adverse Events, which have a possibility of being related to the device, stimulation or study surgery.	Sponsor	Immediately, but no later than 24 hours after investigator is first notified of the event.	See below for UADE, if the result of investigation determines that the event is a UADE.	See below for UADE.
Unanticipated Adverse Device Effects	Sponsor and IRB	Immediately, but no later than 24 hours after investigator is first notified of the event.	FDA, IRBs, and Investigators	Within 10 working days of notifications of Unanticipated Adverse Device Effects, the event.
Withdrawal of IRB Approval or other action on part of the IRB that affects the study	Sponsor	Within 5 working days of notice of IRB decision.	FDA, IRBs, and Investigators	Within 5 working days of notification of such withdrawal.
Progress Reports	Sponsor, study monitors and IRB	At regular intervals, but no less than yearly.	FDA and IRBs	Annually on the anniversary of the IDE approval date
Deviations from Protocol	Sponsor and IRB	Emergency – ASAP but no later than 5 working days after the use occurs.<br><br>Other non-emergency protocol deviations require prior sponsor approval. If deviation occurs without approval – within 10 working days the deviation becomes known to the Investigator.	FDA	Emergency - Within 5 working days of notification of such emergency use.<br><br>Other non-emergency protocol deviations are reported in the FDA progress and final reports.
Use of an Investigational Device or Treatment without Informed Consent	Sponsor and IRB	Within 5 working days after the deviation occurs.	FDA	Within 5 working days after notification of such use.
Withdrawal of FDA approval	N/A	N/A	IRBs and Investigators	Within 5 working days of notification of such withdrawal.
Current Investigator List	N/A	N/A	FDA	Every six months

Device Recall and Disposition	N/A	N/A	FDA and IRBs	Within 30 working days after a device recall or withdrawal request is made to an investigator
Final Report	Sponsor and IRB	Within 3 months after termination or completion of study or investigator's participation.	FDA	Within 6 months of completion or termination of the study.
Other	As Required	Upon request by the IRB or FDA, provide accurate, complete, and current information about any aspect of the study.		Upon request by the IRB or FDA, provide accurate, complete, and current information about any aspect of the study.

15.0 INFORMED CONSENT MATERIALS

Informed consent will be obtained from each study participant. The investigator and/or the study coordinator will approach the potential subject, verbally explain the nature of the study, and provide an FDA and IRB approved written informed consent document for his/her review and signature. The investigator and/or study coordinator will be available to answer all questions the subject may have about the study. In addition, Dr. Paul Ford (Cleveland Clinic Bioethics) or one of his designees will participate in the study as a consent monitor.[6] The informed consent document includes all the required elements as outlined in 21 CFR Part 50.25.

16.0 REFERENCES

1 Go, A.S., et al., *Heart disease and stroke statistics--2014 update: a report from the american heart association.* Circulation, 2014. **129**(3): p. e28-e292.

2 Kelly-Hayes M, e.a., *The influence of age and gender on disability following ischemic stroke. The Framingham study.* . J Stroke Cerebrovasc Dis., 2003. **12**: p. 119-126.

3 Sudlow, C.L. and C.P. Warlow, *Comparing stroke incidence worldwide: what makes studies comparable?* Stroke, 1996. **27**(3): p. 550-8.

4 Go, A.S., et al., *Executive summary: heart disease and stroke statistics--2014 update: a report from the american heart association.* Circulation, 2014. **129**(3): p. 399-410.

5 Rosamond, W.D., et al., *Stroke incidence and survival among middle-aged adults: 9-year follow-up of the Atherosclerosis Risk in Communities (ARIC) cohort.* Stroke, 1999. **30**(4): p. 736-43.

6 El-Saed, A., et al., *Geographic variations in stroke incidence and mortality among older populations in four US communities.* Stroke, 2006. **37**(8): p. 1975-9.

[6] As per discussion at the pre-sub meeting, July 20, 2015.

7 Goldstein, L.B., *Acute ischemic stroke treatment in 2007.* Circulation, 2007. **116**(13): p. 1504-14.

8 Granger, C.V., B.B. Hamilton, and G.E. Gresham, *The stroke rehabilitation outcome study--Part I: General description.* Arch Phys Med Rehabil, 1988. **69**(7): p. 506-9.

9 Gresham, G.E., *Stroke outcome research.* Stroke, 1986. **17**(3): p. 358-60.

10 van Kordelaar, J., E. van Wegen, and G. Kwakkel, *Impact of time on quality of motor control of the paretic upper limb after stroke.* Arch Phys Med Rehabil, 2014. **95**(2): p. 338-44.

11 Kwakkel, G., B. Kollen, and J. Twisk, *Impact of time on improvement of outcome after stroke.* Stroke, 2006. **37**(9): p. 2348-53.

12 Plow, E.B., et al., *Rethinking Stimulation of the Brain in Stroke Rehabilitation: Why Higher Motor Areas Might Be Better Alternatives for Patients with Greater Impairments.* Neuroscientist, 2014.

13 Butler, A.J. and S.L. Wolf, *Transcranial magnetic stimulation to assess cortical plasticity: a critical perspective for stroke rehabilitation.* J Rehabil Med, 2003(41 Suppl): p. 20-6.

14 Kollen, B., G. Kwakkel, and E. Lindeman, *Functional recovery after stroke: a review of current developments in stroke rehabilitation research.* Rev Recent Clin Trials, 2006. **1**(1): p. 75-80.

15 Duncan, P.W., et al., *Management of Adult Stroke Rehabilitation Care: a clinical practice guideline.* Stroke, 2005. **36**(9): p. e100-43.

16 Veerbeek JM, v.W.E., van Peppen R, van der Wees PJ, Nendriks E, Rietberg M, Kwakkel G, *What is the evidence for physical therapy poststroke; A systematic review and meta-analysis.* PLoS One, 2014. **9**(2).

17 Hafsteinsdottir TB, A.A., Kappelle LJ, Grypdonck MHF, *Neurodevelopmental treatment after stroke: A comparative study.* Journal of Neurology, Neurosurgery & Psychiatry, 2005. **76**: p. 788-792.

18 Kwakkel G, B.J., van Wegen EEH, Wolf SL, *Constraint-induced movement therapy after stroke: A systematic review and meta-analysis of current evidence. .* Lancet (Neurology), 2015. **14**: p. 224-234.

19 Reiss A, B.S., Wolf SL, *Contemporary Concepts in Upper Extremity Rehabilitation,,* in *Textbook of Neural Repair and Rehabilitation,.* 2014, Cambridge University Press: Cambridge. p. 330-342.

20 Wolf, S.L., et al., *Effect of constraint-induced movement therapy on upper extremity function 3 to 9 months after stroke: the EXCITE randomized clinical trial.* JAMA, 2006. **296**(17): p. 2095-104.

21 Hayward, K., R. Barker, and S. Brauer, *Interventions to promote upper limb recovery in stroke survivors with severe paresis: a systematic review.* Disabil Rehabil, 2010. **32**(24): p. 1973-86.

22 Fregni, F., et al., *A sham-controlled trial of a 5-day course of repetitive transcranial magnetic stimulation of the unaffected hemisphere in stroke patients.* Stroke, 2006. **37**(8): p. 2115-22.

23 Kim, Y.H., et al., *Repetitive transcranial magnetic stimulation-induced corticomotor excitability and associated motor skill acquisition in chronic stroke.* Stroke, 2006. **37**(6): p. 1471-6.

24 Tsubokawa, T., et al., *Chronic motor cortex stimulation in patients with thalamic pain.* J Neurosurg, 1993. **78**(3): p. 393-401.

25 Machado, A., H. Azmi, and A.R. Rezai, *Motor cortex stimulation for refractory benign pain.* Clin Neurosurg, 2007. **54**: p. 70-7.

26 Adkins, D.L., J.E. Hsu, and T.A. Jones, *Motor cortical stimulation promotes synaptic plasticity and behavioral improvements following sensorimotor cortex lesions.* Exp Neurol, 2008. **212**(1): p. 14-28.

27 Adkins, D.L., et al., *Epidural cortical stimulation enhances motor function after sensorimotor cortical infarcts in rats.* Exp Neurol, 2006. **200**(2): p. 356-70.

28 Adkins-Muir, D.L. and T.A. Jones, *Cortical electrical stimulation combined with rehabilitative training: enhanced functional recovery and dendritic plasticity following focal cortical ischemia in rats.* Neurol Res, 2003. **25**(8): p. 780-8.

29 Plautz, E.J., et al., *Post-infarct cortical plasticity and behavioral recovery using concurrent cortical stimulation and rehabilitative training: a feasibility study in primates.* Neurol Res, 2003. **25**(8): p. 801-10.

30 Robert Levy, M.D., Ph.D., Sean Ruland, D.O., Martin Weinand, M.D., David Lowry, M.D., Rima Dafer, M.D., M.P.H., and Roy Bakay, M.D., *Cortical stimulation for the rehabilitation of patients with hemiparetic stroke: a multicenter feasibility study of safety and efficacy.* Journal of Neurosurgery, 2008. **108**(4).

31 Brown, J.A.M.D.L., Helmi L. M.D.; Weinand, Martin M.D.; Cramer, Steven C. M.D., *Motor Cortex Stimulation for the Enhancement of Recovery from Stroke: A Prospective, Multicenter Safety Study.* Neurosurgery, 2006. **58**(3).

32 Plow, E.B., et al., *Invasive cortical stimulation to promote recovery of function after stroke: a critical appraisal.* Stroke, 2009. **40**(5): p. 1926-31.

33 Holsheimer, J., et al., *Cathodal, anodal or bifocal stimulation of the motor cortex in the management of chronic pain?* Acta Neurochir Suppl, 2007. **97**(Pt 2): p. 57-66.

34 Manola, L., et al., *Anodal vs cathodal stimulation of motor cortex: a modeling study.* Clin Neurophysiol, 2007. **118**(2): p. 464-74.

35 Kwakkel, G., et al., *Probability of regaining dexterity in the flaccid upper limb: impact of severity of paresis and time since onset in acute stroke.* Stroke, 2003. **34**(9): p. 2181-6.

36 Benabid, A.L., et al., *Long-term suppression of tremor by chronic stimulation of the ventral intermediate thalamic nucleus.* Lancet, 1991. **337**(8738): p. 403-6.

37 Schiff, N.D., et al., *Behavioural improvements with thalamic stimulation after severe traumatic brain injury.* Nature, 2007. **448**(7153): p. 600-3.

38 Machado, A.G., et al., *Chronic electrical stimulation of the contralesional lateral cerebellar nucleus enhances recovery of motor function after cerebral ischemia in rats.* Brain Res, 2009. **1280**: p. 107-16.

39 Malone, D.A., Jr., et al., *Deep brain stimulation of the ventral capsule/ventral striatum for treatment-resistant depression.* Biol Psychiatry, 2009. **65**(4): p. 267-75.

40 Greenberg, B.D., et al., *Deep brain stimulation of the ventral internal capsule/ventral striatum for obsessive-compulsive disorder: worldwide experience.* Mol Psychiatry, 2010. **15**(1): p. 64-79.

41 Ghosh, P.S., et al., *Deep brain stimulation in children with dystonia: experience from a tertiary care center.* Pediatr Neurosurg, 2012. **48**(3): p. 146-51.

42 Giacino, J., et al., *Central thalamic deep brain stimulation to promote recovery from chronic posttraumatic minimally conscious state: challenges and opportunities.* Neuromodulation, 2012. **15**(4): p. 339-49.

43 Plow, E.B., A. Pascual-Leone, and A. Machado, *Brain stimulation in the treatment of chronic neuropathic and non-cancerous pain.* J Pain, 2012. **13**(5): p. 411-24.

44 Lim, T.T., et al., *Successful deep brain stimulation surgery with intraoperative magnetic resonance imaging on a difficult neuroacanthocytosis case: case report.* Neurosurgery, 2013. **73**(1): p. E184-7; discussion E188.

45 Schrock, L.E., et al., *Tourette syndrome deep brain stimulation: A review and updated recommendations.* Mov Disord, 2014.

46 Lega, B.C., et al., *Deep brain stimulation in the treatment of refractory epilepsy: update on current data and future directions.* Neurobiol Dis, 2010. **38**(3): p. 354-60.

47 Sprengers, M., et al., *Deep brain and cortical stimulation for epilepsy.* Cochrane Database Syst Rev, 2014. **6**: p. CD008497.

48 Pantano, P., et al., *Crossed cerebellar diaschisis.* Brain, 1986. **109**: p. 677 - 694.

49 Yamauchi, H., H. Fukuyama, and J. Kimura, *Hemodynamic and metabolic changes in crossed cerebellar hypoperfusion.* Stroke, 1992. **23**(6): p. 855-60.

50 Shamoto, H. and H.T. Chugani, *Glucose metabolism in the human cerebellum: an analysis of crossed cerebellar diaschisis in children with unilateral cerebral injury.* J Child Neurol, 1997. **12**(7): p. 407-14.

51 Yamauchi, H., et al., *A decrease in regional cerebral blood volume and hematocrit in crossed cerebellar diaschisis.* Stroke, 1999. **30**(7): p. 1429-31.

52 Brunberg, J.A., et al., *Crossed cerebellar diaschisis: occurrence and resolution demonstrated with PET during carotid temporary balloon occlusion.* AJNR Am J Neuroradiol, 1992. **13**(1): p. 58-61.

53 Tanaka, M., et al., *Crossed cerebellar diaschisis accompanied by hemiataxia: a PET study.* J Neurol Neurosurg Psychiatry, 1992. **55**(2): p. 121-5.

54 Kim, S.E. and M.C. Lee, *Cerebellar vasoreactivity in stroke patients with crossed cerebellar diaschisis assessed by acetazolamide and 99mTc-HMPAO SPECT.* J Nucl Med, 2000. **41**(3): p. 416-20.

55 Suga, K., et al., *Crossed cerebellar diaschisis demonstrated by brain SPECT with Tc-99m MAA in a child with an intracardiac right-to-left shunt.* Clin Nucl Med, 1999. **24**(4): p. 282-3.

56 Pantano, P., et al., *Crossed cerebellar diaschisis in patients with cerebral ischemia assessed by SPECT and 123I-HIPDM.* Eur Neurol, 1987. **27**(3): p. 142-8.

57 Flores, L.G., 2nd, et al., *Crossed cerebellar diaschisis: analysis of iodine-123-IMP SPECT imaging.* J Nucl Med, 1995. **36**(3): p. 399-402.

58 Miyazawa, N., et al., *Evaluation of crossed cerebellar diaschisis in 30 patients with major cerebral artery occlusion by means of quantitative I-123 IMP SPECT.* Ann Nucl Med, 2001. **15**(6): p. 513-9.

59 Dum, R.P., C. Li, and P.L. Strick, *Motor and nonmotor domains in the monkey dentate.* Ann N Y Acad Sci, 2002. **978**: p. 289-301.

60 Dum, R.P. and P.L. Strick, *Motor areas in the frontal lobe of the primate.* Physiol Behav, 2002. **77**(4-5): p. 677-82.

61 Dum, R.P. and P.L. Strick, *An unfolded map of the cerebellar dentate nucleus and its projections to the cerebral cortex.* J Neurophysiol, 2003. **89**(1): p. 634-9.

62 Takasawa, M., et al., *Prognostic value of subacute crossed cerebellar diaschisis: single-photon emission CT study in patients with middle cerebral artery territory infarct.* AJNR Am J Neuroradiol, 2002. **23**(2): p. 189-93.

63 Machado, A. and K.B. Baker, *Upside down crossed cerebellar diaschisis: proposing chronic stimulation of the dentatothalamocortical pathway for post-stroke motor recovery.* Front Integr Neurosci, 2012. **6**: p. 20.

64 Spiegel, E.A., et al., *Stereotaxic Apparatus for Operations on the Human Brain.* Science, 1947. **106**(2754): p. 349-50.

65 Spiegel, E.A. and H.T. Wycis, *Mesencephalothalamotomy for relief of intractable pain.* Am J Med, 1949. **6**(4): p. 504.

66 Spiegel, E.A. and H.T. Wycis, *Electroencephalographic studies following thalamic lesions in humans.* Fed Proc, 1948. **7**(1 Pt 1): p. 119.

67 Spiegel, E.A., H.T. Wycis, and et al., *Stereoencephalotomy.* Proc Soc Exp Biol Med, 1948. **69**(1): p. 175-7.

68 Dieckmann, G. and R. Hassler, *Stereotaxic treatment of extrapyramidal myoclonus.* Confin Neurol, 1972. **34**(1): p. 57-63.

69 Hassler, R. and G. Dieckmann, *[Stereotaxic treatment of tics and inarticulate cries or coprolalia considered as motor obsessional phenomena in Gilles de la Tourette's disease].* Rev Neurol (Paris), 1970. **123**(2): p. 89-100.

70 Hassler, R. and G. Dieckmann, *Stereotactic treatment of different kinds of spasmodic torticollis.* Confin Neurol, 1970. **32**(2): p. 135-43.

71 Laitinen, L.V., A.T. Bergenheim, and M.I. Hariz, *Leksell's posteroventral pallidotomy in the treatment of Parkinson's disease.* J Neurosurg, 1992. **76**(1): p. 53-61.

72 Laitinen, L.V., *Brain targets in surgery for Parkinson's disease. Results of a survey of neurosurgeons.* J Neurosurg, 1985. **62**(3): p. 349-51.

73 Laitinen, L.V. and Y. Ohno, *Effects of thalamic stimulation and thalamotomy on the H reflex.* Electroencephalogr Clin Neurophysiol, 1970. **28**(6): p. 586-91.

74 Laitinen, L., *Thalamic targets in the stereotaxic treatment of Parkinson's disease.* J Neurosurg, 1966. **24**(1): p. 82-5.

75 Johansson, G. and L. Laitinen, *Electrical stimulation of the thalamic and subthalamic area in Parkinson's disease.* Confin Neurol, 1965. **26**(3): p. 445-50.

76 Laitinen, L.V., *Leksell's unpublished pallidotomies of 1958-1962.* Stereotact Funct Neurosurg, 2000. **74**(1): p. 1-10.

77 Leksell, L., *Stereotactic apparatus for intracerebral surgery.* acta chir scand, 1949. **99**: p. 229-33.

78 Talairach, J. and G. Szikla, *Atlas of stereotactic concepts to the surgery of epilepsy.* 1967.

79 Talairach, J. and P. Tournoux, *[Stereotaxic localization of central gray nuclei].* Neurochirurgia (Stuttg), 1958. **1**(1): p. 88-93.

80 Alesch, F., et al., *Stimulation of the ventral intermediate thalamic nucleus in tremor dominated Parkinson's disease and essential tremor.* Acta Neurochir (Wien), 1995. **136**(1-2): p. 75-81.

81 Tasker, R.R., et al., *Deep brain stimulation and thalamotomy for tremor compared.* Acta Neurochir Suppl, 1997. **68**: p. 49-53.

82 Pahwa, R., et al., *Comparison of thalamotomy to deep brain stimulation of the thalamus in essential tremor.* Mov Disord, 2001. **16**(1): p. 140-3.

83 Siegfried, J. and J.C. Verdie, *Long-term assessment of stereotactic dentatotomy for spasticity and other disorders.* Acta Neurochir (Wien), 1977(Suppl 24): p. 41-8.

84 Krayenbuhl, H. and J. Siegfried, *Dentatotomies or thalamotomies in the treatment of hyperkinesia.* Confin Neurol, 1972. **34**(2): p. 29-33.

85 Slaughter, D.G., B.S. Nashold, Jr., and G.G. Somjen, *Electrical recording with micro- and macroelectrodes from the cerebellum of man.* J Neurosurg, 1970. **33**(5): p. 524-8.

86 Nashold, B.S., Jr. and D.G. Slaughter, *Effects of stimulating or destroying the deep cerebellar regions in man.* J Neurosurg, 1969. **31**(2): p. 172-86.

87 Nasold, B.S., Jr., D.G. Slaughter, Jr., and J. Harrison, *A stereotaxic approach and evaluation of the cerebellar nuclei of man.* Confin Neurol, 1969. **31**(1): p. 56.

88 Slaughter, D.G. and B.S. Nashold, Jr., *Stereotactic coordinates for the human dentate nucleus.* Confin Neurol, 1968. **30**(5): p. 375-84.

89 Schvarcz, J.R., R.E. Sica, and E. Morita, *Chronic self-stimulation of the dentate nucleus for the relief of spasticity.* Acta Neurochir Suppl (Wien), 1980. **30**: p. 351-9.

90 Schvarcz, J.R., *Stimulation of the dentate nuclei for spasticity.* Acta Neurochir Suppl (Wien), 1987. **39**: p. 124-5.

91 Sica, R.E., et al., *Changes in the excitability of segmental moton eurone polls by chronic cerebellar dentate nucleus stimulation.* Electromyogr Clin Neurophysiol, 1984. **24**(3): p. 163-76.

92 Schvarcz, J.R., et al., *Electrophysiological changes induced by chronic stimulation of the dentate nuclei for cerebral palsy.* Appl Neurophysiol, 1982. **45**(1-2): p. 55-61.

93 Galanda, M. and S. Horvath, *Effect of stereotactic high-frequency stimulation in the anterior lobe of the cerebellum in cerebral palsy: a new suboccipital approach.* Stereotact Funct Neurosurg, 2003. **80**(1-4): p. 102-7.

94 Galanda, M. and S. Horvath, *Stereotactic stimulation of the anterior lobe of the cerebellum in cerebral palsy from a suboccipital approach.* Acta Neurochir Suppl, 2007. **97**(Pt 2): p. 239-43.

95 Adkins-Muir DL, J.T., *Cortical electrical stimulation combined with rehabilitative training: enhanced functional recovery and dendritic plasticity following focal cortical ischemia in rats.* Neurol Res., 2003. **60**(4): p. 447-55.

96 Allred, R.P. and T.A. Jones, *Unilateral ischemic sensorimotor cortical damage in female rats: forelimb behavioral effects and dendritic structural plasticity in the contralateral homotopic cortex.* Exp Neurol, 2004. **190**(2): p. 433-45.

97 Kleim, J.A., J.A. Boychuk, and D.L. Adkins, *Rat models of upper extremity impairment in stroke.* ILAR J, 2007. **48**(4): p. 374-84.

98 Park, H.J., et al., *Semi-automated method for estimating lesion volumes.* J Neurosci Methods, 2013. **213**(1): p. 76-83.

99 Windle, V., et al., *An analysis of four different methods of producing focal cerebral ischemia with endothelin-1 in the rat.* Exp Neurol, 2006. **201**(2): p. 324-34.

100 Yanamoto, H., et al., *Evaluation of MCAO stroke models in normotensive rats: standardized neocortical infarction by the 3VO technique.* Exp Neurol, 2003. **182**(2): p. 261-74.

101 Luke, L.M., R.P. Allred, and T.A. Jones, *Unilateral ischemic sensorimotor cortical damage induces contralesional synaptogenesis and enhances skilled reaching with the ipsilateral forelimb in adult male rats.* Synapse, 2004. **54**(4): p. 187-99.

102 Yanamoto, H., et al., *Three-vessel occlusion using a micro-clip for the proximal left middle cerebral artery produces a reliable neocortical infarct in rats.* Brain Res Brain Res Protoc. , 1998. **3**(4): p. 209-20.

103 DeBow, S.B., et al., *Constraint-induced movement therapy and rehabilitation exercises lessen motor deficits and volume of brain injury after striatal hemorrhagic stroke in rats.* Stroke, 2003. **34**(4): p. 1021-6.

104 Hudzik, T.J., et al., *Long-term functional end points following middle cerebral artery occlusion in the rat.* Pharmacol Biochem Behav, 2000. **65**(3): p. 553-62.

105 Montoya, C.P., et al., *The "staircase test": a measure of independent forelimb reaching and grasping abilities in rats.* Journal of neuroscience methods., 1991. **36**(2-3): p. 219-28.

106 Rasmussen, R.S., et al., *Effects of microplasmin on recovery in a rat embolic stroke model.* Neurol Res, 2008. **30**(1): p. 75-81.

107 Tuor, U.I., et al., *Long-term deficits following cerebral hypoxia-ischemia in four-week-old rats: correspondence between behavioral, histological, and magnetic resonance imaging assessments.* Exp Neurol, 2001. **167**(2): p. 272-81.

108 Ballermann, M., et al., *The pasta matrix reaching task: a simple test for measuring skilled reaching distance, direction, and dexterity in rats.* J Neurosci Methods, 2001. **106**(1): p. 39-45.

109 Chiken, S. and H. Tokuno, *Impairment of skilled forelimb use after ablation of striatal interneurons expressing substance P receptors in rats: an analysis using a pasta matrix reaching task.* Exp Brain Res., 2005. **162**(4): p. 532-6.

110 Hicks, A.U., et al., *Enriched environment enhances transplanted subventricular zone stem cell migration and functional recovery after stroke.* Neuroscience, 2007. **146**(1): p. 31-40.

111 Colbourne, F., et al., *Prolonged but delayed postischemic hypothermia: a long-term outcome study in the rat middle cerebral artery occlusion model.* J Cereb Blood Flow Metab, 2000. **20**(12): p. 1702-8.

112 Kerr, A.L. and T.K. A., *Compensatory limb use and behavioral assessment of motor skill learning following sensorimotor cortex injury in a mouse model of ischemic stroke.* J Vis Exp. , 2014(89).

113 Angaut, P. and F. Cicirata, *Dentate control pathways of cortical motor activity. Anatomical and physiological studies in rat: comparative considerations.* Arch Ital Biol, 1990. **128**(2-4): p. 315-30.

114 Rispal-Padel, L., F. Cicirata, and C. Pons, *Contribution of the dentato-thalamo-cortical system to control of motor synergy.* Neurosci Lett, 1981. **22**(2): p. 137-44.

115 Panto MR, Z.A., Parenti R, Serapide MF, Cicirata F., *Corticonuclear projections of the cerebellum preserve both anteroposterior and mediolateral pairing patterns.* Eur J Neurosci., 2001. **13**(4): p. 694-708.

116 Cicirata, F., et al., *Multiple representation in the nucleus lateralis of the cerebellum: an electrophysiologic study in the rat.* Exp Brain Res, 1992. **89**(2): p. 352-62.

117 Angaut, P. and F. Cicirata, *The dentatorubral projection in the rat: an autoradiographic study.* Behav Brain Res, 1988. **28**(1-2): p. 71-3.

118 Angaut, P., F. Cicirata, and F. Serapide, *Topographic organization of the cerebellothalamic projections in the rat. An autoradiographic study.* Neuroscience, 1985. **15**(2): p. 389-401.

119 Massion, J. and L. Rispal-Padel, *Spatial organization of the cerebello-thalamo-cortical pathway.* Brain Res, 1972. **40**(1): p. 61-5.

120 Meftah, E.M. and L. Rispal-Padel, *Cerebello-cortical plasticity. Role of somesthetic influx in the change of the cerebellar effects on the musculature.* C R Acad Sci III, 1995. **318**(2): p. 219-27.

121 Baker, K.B., et al., *Deep brain stimulation of the lateral cerebellar nucleus produces frequency-specific alterations in motor evoked potentials in the rat in vivo.* Exp Neurol, 2010. **226**(2): p. 259-64.

122 Furmaga, H., et al., *Effects of ketamine and propofol on motor evoked potentials elicited by intracranial microstimulation during deep brain stimulation.* Front Syst Neurosci, 2014. **8**: p. 89.

123 Machado, A.G., et al., *Chronic 30-Hz deep cerebellar stimulation coupled with training enhances post-ischemia motor recovery and peri-infarct synaptophysin expression in rodents.* Neurosurgery, 2013. **73**(2): p. 344-53; discussion 353.

124 Cooperrider, J., et al., *Chronic deep cerebellar stimulation promotes long-term potentiation, microstructural plasticity, and reorganization of perilesional cortical representation in a rodent model.* J Neurosci, 2014. **34**(27): p. 9040-50.

125 Platz, T., Pinkowski, C., et al., *Reliability and validity of arm function assessment with standardized guidelines for the Fugl-Meyer Test, Action Research Arm Test and Box and Block Test: a multicentre study.* Clin Rehabil 2005. **19**(4): p. 404-411.

126 Sanford, J., Moreland, J., et al., *Reliability of the Fugl-Meyer assessment for testing motor performance in patients following stroke.* Phys Ther, 1993. **73**: p. 447-454.

127 Dettmann, M.A., Linder, M. T., et al., *Relationships among walking performance, postural stability, and functional assessments of the hemiplegic patient.* Am J Phys Med 1987. **66**: p. 77-90.

128 Hsieh, Y.W., Wu, C. Y., et al., *Responsiveness and validity of three outcome measures of motor function after stroke rehabilitation.* Stroke, 2009. **40**(4): p. 1386-1391.

129 Lin, J.H., Hsueh, I. P., et al., *Psychometric properties of the sensory scale of the Fugl-Meyer Assessment in stroke patients.* Clin Rehabil, 2004. **18**(4): p. 391-397.

130 Duncan, P.W., Lai, S. M., et al., *Defining post-stroke recovery: implications for design and interpretation of drug trials.* Neuropharmacology, 2000. **39**(5): p. 835-841.

131 Chen, H.M., Chen, C. C., et al., *Test-retest reproducibility and smallest real difference of 5 hand function tests in patients with stroke.* Neurorehabilitation and Neural Repair, 2009. **23**(5): p. 435-440.

132 Beebe, J.A.a.L., C. E. , *Relationships and responsiveness of six upper extremity function tests during the first six months of recovery after stroke.* J Neurol Phys Ther, 2009. **33**(2): p. 96-103.

133 JL, A. and W. SL, *The use of kinetics as a marker for manual dexterity after stroke and stroke recovery. .* Topics in stroke rehabilitation, 2099. **16**(4): p. 223-36.

134 Gorniak SL, A.J., *Effects of aging on force coordination in bimanual task performance.* Exp Brain Res., 2013. **229**(2): p. 273-84.

135 Gorniak SL, P.M., McDaniel C, Alberts JL, *Impaired Object Handling during Bimanual Task Performance in Multiple Sclerosis.* Multiple Sclerosis International, 2014. **2014**.

136 Rudick RA, M.D., Béthoux F, Rao SM, Lee J-C, Stough D, Reece C, Schindler D, Mamone B, Alberts J, *The Multiple Sclerosis Performance Test (MSPT): An iPad-Based Disability Assessment Tool.* JoVE, 2014(88).

137 Kopp, B., Kunkel, A., et al., *The Arm Motor Ability Test: reliability, validity, and sensitivity to change of an instrument for assessing disabilities in activities of daily living.* Arch Phys Med Rehabil 1997. **78**(6): p. 615-620.

138 Chae, J., Labatia, I., et al., *Upper limb motor function in hemiparesis: concurrent validity of the Arm Motor Ability test.* Am J Phys Med Rehabil 2003. **82**(1): p. 1-8.

139 Anderson, C., Laubscher, S., et al., *Validation of the Short Form 36 (SF-36) health survey questionnaire among stroke patients.* Stroke, 1996. **27**: p. 1812-1816.

140 Dorman, P., Slattery, J., et al., *Qualitative comparison of the reliability of health status assessments with the EuroQol and SF-36 questionnaires after stroke. United Kingdom Collaborators in the International Stroke Trial.* Stroke, 1998. **29**: p. 63-68.

141 Dorman, P.J., Dennis, M., et al., *How do scores on the EuroQol relate to scores on the SF-36 after stroke?* Stroke, 1999. **30**: p. 2146-2151.

142 Pinto EB1, M.I., Vilela RN, Santos LC, Oliveira-Filho J., *Validation of the EuroQol quality of life questionnaire on stroke victims.* Arq Neuropsiquiatr, 2011. **69**(2B): p. 320-3.

143 Kotila, M., Numminen, H., et al., *Depression after stroke: results of the FINNSTROKE Study.* Stroke, 1998. **29**: p. 368-372.

144 Aben, I., Verhey, F., et al., *Validity of the Beck Depression Inventory, Hospital Anxiety and Depression Scale, SCL-90, and Hamilton Depression Rating Scale as screening instruments for depression in stroke patients.* Psychosomatics 2002. **43**(5): p. 386.

145 Butler AJ, W.S., *Putting the brain on the map: Use of Transcranial magnetic stimulation to assess and induce cortical plasticity of upper-extremity movement. .* Physical Therapy. , 2007. **87**: p. 719-736.

146 *MagPro R30 Magnetic Stimulator Insturctions for Use reference.*

147 Kuhn, A.A., et al., *Modulation of motor cortex excitability by pallidal stimulation in patients with severe dystonia.* Neurology, 2003. **60**(5): p. 768-74.

148 Rossi, S., et al., *Safety, ethical considerations, and application guidelines for the use of transcranial magnetic stimulation in clinical practice and research.* Clin Neurophysiol., 2009. **120**(12): p. 2008-2039.

149 Kuhn, A.A., et al., *Pseudo-bilateral hand motor responses evoked by transcranial magnetic stimulation in patients with deep brain stimulators.* Clin Neurophysiol, 2002. **113**(3): p. 341-5.

150 Hidding, U., et al., *MEP latency shift after implantation of deep brain stimulation systems in the subthalamic nucleus in patients with advanced Parkinson's disease.* Mov Disord, 2006. **21**(9): p. 1471-6.

151 Cunningham, D.A., et al., *Assessment of Inter-Hemispheric Imbalance Using Imaging and Noninvasive Brain Stimulation in Patients With Chronic Stroke.* Archives of Physical Medicine and Rehabilitation, 2015. **96**: p. S94-103.

152 Angelis, C.D., et al., *Clinical Trial Registration: A Statement from the International Committee of Medical Journal Editors.* N Engl J Med 2004. **351**: p. 1250-1251.

153 *Medtronic DBS Therapy for Parkinson's Disease and Essential Tremor Clinical Summary.* 2013.

154 Lyons KE, W.S., Overman J, et al., *Surgical and hardware complications of subthalamic stimulation: a series of 160 procedures. .* Neurology., 2004. **63**: p. 612-616.

155 Rezai AR, P.M., Baker KB, Sharan AD, Nyenhuis J, Tkach J, et al., *Neurostimulation system used for deep brain stimulation (DBS): MR safety issues and implications of failing to follow safety recommendations.* Invest Radiol, 2004. **39**(5): p. 300-3.

156 Sharan A, R.A., Nyenhuis JA, Hrdlicka G, Tkach J, Baker K, et al. , *MR safety in patients with implanted deep brain stimulation systems (DBS). .* Acta Neurochir Suppl., 2003. **87**: p. 141-5.

157 Alter, M., et al., *The Lehigh Valley Recurrent Stroke Study: description of design and methods.* Neuroepidemiology., 1993. **12**(2): p. 241-8.

158 Hankey RJ, J.K., Broadhurst RJ, et al., *Long-term risk of first recurrent stroke in the Perth Community Stroke Study. .* Stroke, 1998. **29**: p. 2491-2500.

159 Hillen T, C.C., Tilling K, et al., *Cause of stroke recurrence is multifactorial.* Stroke, 2003. **34**: p. 1457-1463.

160 Petty GW, B.R., Whisnant JP, et al., *Ischemia stroke subtypes: a population-based study of functional outcome, survival, and recurrence.* Stroke, 2000. **31**: p. 1062-1068.

161 Galanda, M., L. Mistina, and O. Zoltan, *Behavioural responses to cerebellar stimulation in cerebral palsy.* Acta Neurochir Suppl (Wien), 1989. **46**: p. 37-8.

162 Galanda, M. and O. Zoltán, *Motor and psychological responses to deep cerebellar stimulation in cerebral palsy (correlation with organization of cerebellum into zones).* Acta Neurochir Suppl (Wien), 1987. **39**: p. 129-31.

163 Sankarasubramanian V., et al., *Reproducibility of transcranial magnetic stimulation metrics in the study of proximal upper limb muscles*. Journal of electromyography and kinesiology : official journal of the International Society of Electrophysiological Kinesiology. 2015;25(5):754-64.

164 Cunningham D.A., et al., *Stimulation targeting higher motor areas in stroke rehabilitation: A proof-of-concept, randomized, double-blinded placebo-controlled study of effectiveness and underlying mechanisms.* Restorative neurology and neuroscience. 2015;33(6):911-26.

165 Baker KB, et al., *Subthalamic nucleus deep brain stimulus evoked potentials: physiological and therapeutic implications.* Movement Disorders. 2002;17(5):969-83.

166 Kuriakose R, et al., *The nature and time course of cortical activation following subthalamic stimulation in Parkinson's disease*. Cereb Cortex. 2010;20(8):1926-36.

167 Wittenberg GF, et al., Constraint-induced therapy in stroke: magnetic-stimulation motor maps and cerebral activation. Neurorehabilitation and neural repair. 2003;17(1):48-57.

168 Sawaki L, et al., Constraint-induced movement therapy results in increased motor map area in subjects 3 to 9 months after stroke. Neurorehabilitation and neural repair. 2008;22(5):505-13.

169 Kumar R, et al., *Safety of transcranial magnetic stimulation in patients with implanted deep brain stimulators*. Mov Disord. 1999;14(1):157-8.

170 Tisch S, et al., stimulation modifies after-effects of paired associative stimulation on motor cortex excitability in primary generalized dystonia. Experimental neurology. 2007;206(1):80-5.

171 Plow EB, et al., *Age-related weakness of proximal muscle studied with motor cortical mapping: a TMS study*. PloS one. 2014;9(2):e89371.

172 Marconi B, et al., *Long-term effects on cortical excitability and motor recovery induced by repeated muscle vibration in chronic stroke patients*. Neurorehabilitation and neural repair. 2011;25(1):48-60.

173 Streletz LJ, et al., *Transcranial magnetic stimulation: cortical motor maps in acute spinal cord injury.* Brain topography. 1995;7(3):245-50.

174 Rosa, M, et al., Risk of Infection After Local Field Potential Recording from Externalized Deep Brain Stimulation Leads in Parkinson's Disease. World Neurosurgery, 2017; 97:64-69.

175 Arya, R., et al., Adverse events related to extraoperative invasive EEG monitoring with subdural grid electrodes: A systematic review and meta-analysis Epilepsia. 2013 May;54(5):828-39.

176 Tanriverdi T, et al., *Morbidity in epilepsy surgery: an experience based on 2449 epilepsy surgery procedures from a single institution.* Journal of Neurosurgery 2009 June; 110(6):1111-1123.

177 Bohannon, R. and Smith, M. *Interrater reliability of a modified Ashworth scale of muscle spasticity.*" Physical Therapy. 1987;67(2): 206.

178 Gregson, J., et al. *Reliability of the Tone Assessment Scale and the modified Ashworth scale as clinical tools for assessing poststroke spasticity.* Archives of physical medicine and rehabilitation. 1999;80(9): 1013-1016.

179 Katz, R., Rovai, G., et al. *Objective quantification of spastic hypertonia: correlation with clinical findings.* Archives of physical medicine and rehabilitation. 1992; 73(4): 339

180 Lin, F. and Sabbahi, M. "*Correlation of spasticity with hyperactive stretch reflexes and motor dysfunction in hemiplegia.* Archives of physical medicine and rehabilitation.1999;80(5): 526-530.

181 Cooperrider J, et al., Differential frequency modulation of neural activity in the lateral cerebellar nucleus in failed and successful grasps. Experimental neurology. 2016; 277:27-34

182 Liepert J, et al., *Motor cortex plasticity during forced-use therapy in stroke patients: a preliminary study*. Journal of neurology. 2001;248(4):315-21.

183 Wagle Shukla A, et al., *Long-term subthalamic nucleus stimulation improves sensorimotor integration and proprioception.* Journal of neurology, neurosurgery, and psychiatry. 2013;84(9):1020-8.

184 See J, et al., *A Standardized Approach to the Fugl-Meyer Assessment and Its Implications for Clinical Trials.* Neurorehabilitation and Neural Repair. 2013;27:732–741.

Summary of changes to the protocol

The list provided below summarizes the changes to the study for each revision of the protocol.

There were no protocol changes related to the target area (dentate nucleus) or the intended patient population: severe and moderate-to-severe stroke survivors. Because this was a first-in-human study investigating a novel indication (DBS for post-stroke rehabilitation) with a novel target (dentate nucleus), the FDA initially approved one implant at a time (staggered implantation) and limited enrollment only to individuals with severe stroke. As there were no significant perioperative complications among the first participants, the FDA then approved protocol changes that permitted all remaining subjects to be enrolled without completion of the prior subject's implantation as well as enrollment of subjects with moderate-to-severe stroke. Other changes included those related to pre-and-postoperative imaging, exploratory mechanistic studies and minor changes to correct oversights from prior versions.

The most meaningful change to protocol was Rev. 8.0. This change was made in response to the extensive therapeutic effects noted in our first participant. As for all other participants subsequently enrolled with distal preservation of motor function, this individual showed meaningful improvements of impairment during each of the originally planned four months of DBS+Rehab. The magnitude and duration of recovery surpassed our original estimation, which had been based on prior literature of neuromodulation for post-stroke rehabilitation that included studies investigating epidural electrical stimulation. A discussion was carried out with the FDA to change the protocol, which resulted in adding up to four additional months of DBS+Rehab depending on the rate of improvement in the prior 8 weeks. This was deemed to be in the best interest of this first and subsequent participants, maximizing their beneficence for the risk taken. Note that ALL participants underwent the study under this modification, including the first participant. Another meaningful change to the protocol was the addition of tele-rehabilitation under Rev. 17.0. This was an important operational change that enabled study continuation during the COVID-19 pandemic.

Subject numbers indicate what protocol was IRB approved on the date the participant signed consent. Please note that participants who were already undergoing the study at the time of the change were also affected by changes, including the addition of mechanistic studies or imaging prior to programming.

Rev 4.0

- Original Protocol

Rev 5.0 - Subject 001, and 002

- FDA approved five subjects with staggered implants; the next subject enrolls 1-month after the previous implant.
- Replace the MagPro R30 TMS device for measuring cortical excitability with a Magstim 200^2 TMS device.

Rev 6.0

- Added post-explant 7T MRI to verify the final lead location.
- Added pre-programming CT to verify lead location before programming.
- Added FDA specified CT risks.

Rev 7.0

- Added temporary (sub-acute) percutaneous externalization of the DBS lead for local field potential recordings before neurostimulator implant and at the end of the implanted period, before the DBS leads are explanted.
- Included InMotion robotic ARM™ for assisted active and passive arm movements during percutaneous externalization and DBS device programming.
- Added transcranial magnetic stimulation (TMS) motor maps during TMS evaluations.
- Added time points for collecting the Ashworth Spasticity scale.

Rev 8.0

- Subjects received a minimum of four months of test treatment (as defined in the original protocol), after which test treatment was extended on a month-by-month basis for up to four additional months. Test treatment was extended only for subjects who demonstrated improvement greater than the minimal detectable change (MDC) as determined by the FMA-UE across eight weeks (i.e., two months).

Rev 9.0 - Subjects 003, 004, and 005

- Expanded selection criteria to include moderate to severely impaired patients as defined by the Fugl-Meyer assessment of FMA-UE ≤ 33 (initially FMA ≤ 20)
- Expand qualifying age range to include subjects 25-75 years of age.
- Expand time since stroke to include subjects who had a first-time ischemic stroke 1-3 years ago
- Expand approval to 12 subjects implanted (Enrollment was initially limited to five subjects enrolled in a staggered manner).
- Change the 7T MRI to be an optional imaging study such that subjects with an implant indicated for 3T (e.g., knee replacement) but not explicitly indicated for 7T may participate in the study.

Rev 10.0

- Updated labeling to reflect Boston Scientific's PMA approval for bilateral stimulation of the subthalamic nucleus (STN) as adjunctive therapy for Parkinson's Disease (PD)

Rev 11.0 - Subjects 007, and 008

- Included Boston Scientific's Vercise™ PC (instead of rechargeable) with directional lead (Cartesia™ lead) for implant.
- Included the use of Boston Scientific's GUIDE™ modeling system for retrospective analysis of lead position and stimulation.

Rev 12.0

- Use of on-campus Cleveland Clinic Hotel, during 1-week lead externalization at explant instead of requiring to be hospitalized).
- Added additional programming visits (from 8 to 16) to allow for a complete evaluation of possible DBS parameters before turning DBS ON.
- Added H-reflexes to quantify improvements in spasticity due to DBS
- Reduction of interim safety reporting to FDA and DMC review to occur after every third subject implanted (previously after every implant).

Rev 13.0

- Expanded selection criteria to include moderately impaired patients as defined by the Fugl-Meyer assessment of FMA-UE ≤ 42 (previously FMA ≤ 33, and FMA ≤ 20)
- Added Global Vigor and Affect Scale (GVA) and Brief Fatigue Inventory (BFI) to control fatigue during programming.

Rev 14.0 - Subjects 010, and 011

- Updated labeling to reflect Boston Scientific's PMA approval of Vercise™ PC.

Rev 15.0

- Added cerebellar brain inhibition (CBI), a TMS method for quantifying the inhibitory projections from the cerebellar to the motor cortex.

Rev 16.0 - Subject 013

- Included the option for therapists to provide therapeutic intervention via telerehabilitation (tele-rehab), utilizing the existing Cleveland Clinic telehealth platform.

Rev 17.0 - Subjects 014, and 015

- Added questionnaires to evaluate the use of tele-rehab.

Rev 18.0

- Added three programming visits after DBS OFF to further evaluate the relationship between DBS amplitude and evoked cortical (i.e., scalp) EEG response.

Rev 19.0

- Included mitigations to reduce potential exposure to SARS-CoV-2.

1 INTRODUCTION AND CHARTER OF DMC

The Data Monitoring Committee (DMC) serves as an independent advisory board to provide counsel and oversight for Enspire DBS Therapy, Inc.'s IDE Study: Electrical Stimulation of the Dentate Nucleus area (EDEN) for Improvement of Upper Extremity Hemiparesis due to Ischemic Stroke: A Safety and Feasibility Study, Protocol REDD 0002.

The DMC will be established to provide additional, independent oversight to enhance safety of study participants and ensure subjects' rights and welfare. The DMC will be responsible for safeguarding the interests of trial participants by assessing the safety and efficacy of the interventions during the trial, and for monitoring the overall conduct of the clinical trial.

The DMC periodically reviews study results, evaluates safety data for the IDE study and make recommendations to the Sponsor. The Sponsor has the responsibility to accept or reject the DMC recommendations.

2 ORGANIZATION

2.1 Composition of the DMC

The DMC will consist of three experts with relevant clinical specialties. This will include at least one physician that practices neurology or physical medicine/rehabilitation and specialize in post stroke care and at least one neurosurgeon experienced in DBS therapy. They should also be familiar with FDA regulated clinical research with investigational implantable devices and/or investigational drugs. Experience with serving on other DMC, DSMB and IRB are desirable. These three experts are the core members with voting responsibility.

Other experts such as physical therapist, rehabilitation professionals, and medical ethicists may be invited to participate on an as needed basis as additional advisors to evaluate complex and difficult issues.

2.2 Selection of DMC Members

The DMC and members are selected by the Sponsor.

3 CONFIDENTIALITY

All members will treat as confidential the reports, meeting discussions, and summary notes. They will sign a Confidential Agreement with the Sponsor.

The Sponsor will make every effort to protect individual identifiable data for each study subject. The reports to the DMC should have been de-identified as per HIPPA Privacy Rules. Subjects should be identified by subject # as per protocol.

The DMC is also responsible for maintaining the confidentiality of its internal discussions and activities as well as the contents of reports provided to it.

4 CONFLICT OF INTEREST

Financial Conflict of Interest: Members of the DMC will not buy, sell, or hold stock or stock options in the Sponsor company for the following periods: from the first meeting of the DMC until the last meeting and the study results are made public. Each member will be reimbursed for their time working in the DMC but agrees not to serve as a paid consultant to the Sponsor for other duties and will not receive any other type of compensation during the study. The guidelines will also apply to the member's spouse and dependents. The Sponsor will hold conflict-of-interest statements from each DMC member.

Intellectual and Research Conflict of Interest: DMC members may not participate in the study as principal or co-investigators, or as study subject care physicians. Individuals known to have strong views on the relative merits of the interventions under study may not be DMC members. A DMC member ideally should not have a relationship with the principal investigators or sponsor employees that could be considered reasonably likely to affect their objectivity. DMC members should not engage in competing research or related research using another manufacturer's devices.

Certain other activities are not viewed as constituting conflicts of interest but must be reported annually to the Sponsor. These include: the participation of members in other research projects supported by the Sponsor, and occasional scientific consulting to the Sponsor on issues not related to the product in the trial and for which there is no financial payment or other compensation.

5 DMC RESPONSIBILITIES

The DMC will be responsible for safeguarding the interests of trial participants by assessing the safety of the interventions during the trial, and for monitoring the overall conduct of the clinical trial. The DMC is responsible for defining its deliberative processes, including event triggers that would call for an unscheduled review, stopping guidelines, and voting procedures prior to initiating any data review.

Monitoring for Safety: The DMC reviews safety data from the study and will make recommendations, if appropriate. The DMC will review data related to: implant and other surgical safety; stimulation (therapy) safety for at least one-month data of " DBS + rehab " phase for each subject, and any safety information collected, whether it is related to the device/therapy or not.

Making Recommendations: The DMC may recommend one of the following actions to the study Sponsor; note this is not an exhaustive list:

- May expand the study beyond five subjects and enroll additional subjects with the current inclusion/exclusion criteria.

- Subject’s inclusion criteria can be expanded to subjects with less severe symptoms, i.e. with FMA-UE scores higher than 20.
- May enroll additional subjects with the current inclusion/exclusion criteria.
- Modify the study protocol. Modifications may include, but are not limited to, changes in inclusion/exclusion criteria, frequency of visits or safety monitoring, alterations in study procedures, adjustments in sample size, changes in duration of observation and follow up.
- Discontinue the study (with provisions for orderly discontinuation in accord with good medical practice) due to safety concerns.

6 RESPONSIBILITIES OF THE SPONSOR

The Sponsor is responsible to the DMC for the following (not an exhaustive list):

- Collection of CRFs and relevant clinical data and conduct on-site monitoring as per monitoring plan.
- Ensuring the completeness and accuracy of all data collected to the extent required by the DMC; this includes CRF data, pertinent imaging data and comprehensive follow up of any adverse event data, whether it is considered to be related by the device, procedure, or stimulation therapy or not.
- Providing aggregate data sets and prepares summary report to DMC. Prepare patient data in an agreement upon format for review by the DMC.
- The Sponsor is to de-identify all subject personal information as per HIPPA guidelines. Provide subject level safety data to DMC for review, especially for the first five subjects.

- Provide such data reports to the DMC members approximately three days prior to date of the meeting.
- Report to DMC chair in the event of an Unanticipated Serious Adverse Event within three days for the Chair to call an unscheduled meeting to evaluate such data.
- Making resources available to the DMC as required, to carry out its designated functions.
- Report all Adverse Events to FDA as per regulatory requirements. Inform IRB of the DMC recommendations, in the event that the clinical trial be stopped or modified.

7 CONDUCT OF DMC MEETINGS

7.1 Meetings Schedule

Meeting To Evaluate Safety Data for First patient: For the first patient, the DMC will review safety data within 10 days of availability of the safety dataset once the first the subject has completed one (1) month of the Testing Phase. The objective of this meeting is to evaluate safety data for the first subject and makes recommendations to the Sponsor.

Sponsor then requests expansion of the IDE to enroll additional patients. The DMC will also make recommendations regarding whether it is appropriate to change the inclusion/exclusion criteria to enroll subjects with FMA-UE scores of > 20, if initial safety data warrant enrolling less severely affected stroke patients.

Scheduled Meetings: The frequency of scheduled meetings depends on subject enrollment and adverse event rates. The DMC will convene according to the following schedule: first subject implanted; and when the first subject completes the testing phase. Upon FDA approval to allow DBS implant for the four enrolled subjects, the DMC will review data after the four additional subjects have been implanted and all the enrolled five patients have completed the Testing Phase (DBS + Rehab).

If FDA approves further expansion of the trial to enroll a total of 12 subjects (as per original study plan), the DMC will review data when the additional seven subjects have completed the Testing Phase, and then when the last subject completes the Testing Phase.

Unscheduled Meeting to Evaluate Important and Urgent Safety Data: If significant and urgent safety data emerges, e.g. serious device or treatment related adverse events, the DMC will convene a meeting to evaluate the safety within five days.

One member of the DMC will be assigned as an independent reviewer of all serious adverse events. The independent reviewer will be notified within two business days of becoming aware of an adverse event to assess whether the event requires review by the full board. If full board review is required, an unscheduled DMC board meeting will be held via teleconference within five business days to determine if enrolment should be halted until the event has been satisfactorily resolved. Specific rules will be determined by the DMC during the first

organizational meeting.

7.2 Quorum

All three committee members are required at all scheduled meetings, phone conferences, or unscheduled meetings.

7.3 Meeting Format and Schedules

Meeting Format: DMC meetings can be face to face or via teleconference or videoconference. Meetings will consist of open and closed portions.

Open Session: During the initial open portion of a meeting, the Sponsor/Principal and Co-Investigator(s) may be invited to make brief presentations and be available for questions from the DMC members. The sponsor, PI and co-PI must not attempt to influence the outcome of the evaluation.

Closed Session: The closed session will be restricted to the DMC for the evaluation and discussion of aggregate data, and subject level data safety, if appropriate. Following the closed session, an open session can be held with the Sponsor at which the DMC will communicate their decision, recommendation for changes or any action items for follow- up, if any. The decision and the recommendation to the sponsor can be communicated verbally, followed up with a brief written summary.

7.4 Voting

The DMC members vote on all recommendations to be submitted to the Sponsor. To vote, a Committee member must be present at convened scheduled meetings or participate through conference calls.

7.5 Meeting Minutes of DMC

Written meeting minutes of the DMC meeting will be prepared by the Chair of the DMC. The chair of the DMC may assign an administrator to prepare draft minutes. However, all DMC members must approve the minutes.

7.6 Procedures for Recommendations to the Sponsor

Duly voted and passed DMC recommendations to the Sponsor are transmitted in writing to the Sponsor.

8 COMMUICATION WITH FDA

In rare cases, FDA may wish to interact with the DMC to ensure that specific issues of urgent concern to FDA are fully considered by the DMC or to address questions to the DMC regarding the safety data in the on-going trials. DMC members will agree to communicate with FDA directly if such request occurs. The Sponsor is to arrange this communication.

APPENDIX I - DMC DECISION GUIDELINES

A. General Considerations

The trial is a feasibility trial where the primary focus is on patient safety. Therefore, DMC recommendations will be made based on clinical judgment rather than statistical results or rules.

Stopping for significant safety risk:

- Any recommendation to terminate the trial due to significant safety risk will be made on the basis of sound medical judgment. For the first cohort of 4 patients, upon review and deliberation by the DMC, the DMC may immediately halt enrollment/implant of additional patient(s) for the clinical trial if there is no satisfactory explanation or resolution of the reported Serious Adverse Event(s).
- If one or more SAEs with un-explained etiology and lack of satisfactory resolution of the SAE(s), the DMC may recommend terminating the clinical trial as designed. The DMC may also recommend making changes to the protocol to improve the safety of the clinical trial, if it is allowed to re-start.
- For this early phase safety study with no control arm, and especially for the first cohort of subjects, no or little efficacy would be expected and therefore a benefit- risk analysis may not be appropriate. The DMC should take into consideration of the potential scientific gain from continuing a study must be evaluated in the context of ethical considerations for ensuring subjects' rights and welfare.
