# Supplementary material for: Cerebellar deep brain stimulation for chronic post-stroke motor rehabilitation: a phase I trial
Source: Nat Med. 2023 Aug 14;29(9):2366–74. doi: 10.1038/s41591-023-02507-0 (PMC10504081; doi:10.1038/s41591-023-02507-0)
Supplement: Supplementary file 2 — Reporting Summary [file 41591_2023_2507_MOESM2_ESM.pdf]

nature portfolio

Corresponding author(s): Dr. Andre Machado

Last updated by author(s): Jul 12, 2023

Reporting Summary

Nature Portfolio wishes to improve the reproducibility of the work that we publish. This form provides structure for consistency and transparency in reporting. For further information on Nature Portfolio policies, see our Editorial Policies and the Editorial Policy Checklist.

Statistics

For all statistical analyses, confirm that the following items are present in the figure legend, table legend, main text, or Methods section.

n/a	Confirmed	
☐	☒	The exact sample size (*n*) for each experimental group/condition, given as a discrete number and unit of measurement
☐	☒	A statement on whether measurements were taken from distinct samples or whether the same sample was measured repeatedly
☐	☒	The statistical test(s) used AND whether they are one- or two-sided<br>*Only common tests should be described solely by name; describe more complex techniques in the Methods section.*
☐	☒	A description of all covariates tested
☐	☒	A description of any assumptions or corrections, such as tests of normality and adjustment for multiple comparisons
☐	☒	A full description of the statistical parameters including central tendency (e.g. means) or other basic estimates (e.g. regression coefficient) AND variation (e.g. standard deviation) or associated estimates of uncertainty (e.g. confidence intervals)
☐	☒	For null hypothesis testing, the test statistic (e.g. *F*, *t*, *r*) with confidence intervals, effect sizes, degrees of freedom and *P* value noted<br>*Give P values as exact values whenever suitable.*
☒	☐	For Bayesian analysis, information on the choice of priors and Markov chain Monte Carlo settings
☒	☐	For hierarchical and complex designs, identification of the appropriate level for tests and full reporting of outcomes
☒	☐	Estimates of effect sizes (e.g. Cohen's *d*, Pearson's *r*), indicating how they were calculated

Our web collection on statistics for biologists contains articles on many of the points above.

Software and code

Policy information about availability of computer code

Data collection	No software was used.
Data analysis	SAS Studio v 3.6; ANTs-2.3.5; FreeSurfer v7.1.1; FSL version 6.0; R version 4.2.1

For manuscripts utilizing custom algorithms or software that are central to the research but not yet described in published literature, software must be made available to editors and reviewers. We strongly encourage code deposition in a community repository (e.g. GitHub). See the Nature Portfolio guidelines for submitting code & software for further information.

Data

Policy information about availability of data

All manuscripts must include a data availability statement. This statement should provide the following information, where applicable:

- Accession codes, unique identifiers, or web links for publicly available datasets
- A description of any restrictions on data availability
- For clinical datasets or third party data, please ensure that the statement adheres to our policy

Raw data related to safety and feasibility, the primary endpoints of the study, can be shared upon request and review by Cleveland Clinic and the study sponsors. Secondary endpoint data as well as PET imaging data, analyzed or raw, may also be shared. Depending on the data that are requested, we will need to consult with the IRB and sponsors before sharing. Cleveland Clinic has regulations related to data sharing, in particular data that could be used as identifiers. The investigators and the IRB will need to verify that data sharing would be acceptable and within policy for patient protection and within the limits of the informed consent provided

by participants when enrolling in the study. The investigators will also have to consult the sponsors prior to data sharing. Patient-related information not included in this report was collected as part of a clinical trial and may be subject to patient confidentiality.

Given the restricted study population and small sample size, even though any dataset will be stripped of identifiers prior to release for sharing, we believe that there remains the possibility of deductive disclosure of subjects by unique combinations of characteristics. Therefore, we will make the data and associated documentation available to users only under a data-sharing agreement that provides for a commitment to: (1) sing the data only for research purposes and not to identify any individual participant; (2) securing the data using appropriate computer technology; and (3) destroying or returning the data after analyses are completed.

Requests for data can be sent via email to the principal investigator, Dr. Andre Machado (machada@ccf.org).

All requests will be answered within 4 weeks. We anticipate that data will be shared, if there are no risks or low risks to the participants.

Human research participants

Policy information about studies involving human research participants and Sex and Gender in Research.

Reporting on sex and gender	Study team attempted to enroll both male and female subjects, however gender was not considered in the study design. Sex based analyses were not performed due to small sample size. Overall, 4 females and 8 males completed study participation. Sex assignment was based on self-identification.
Population characteristics	Of the 12 subjects that completed participation, 10 identified as "white", while 2 identified as "black". Subjects ranged in age from 48 - 70 years in age, with an average age of 57.4 years. All subjects are ischemic stroke survivors with upper extremity Fugl-Meyer Assessment [FM-UE] ≤ 42.
Recruitment	The study team used a multimodal recruitment and enrollment strategy to increase access to study to all participants who might fulfill the inclusion criteria. Over 11,000 potential participants were identified through a combination of physician referrals, EMR review, community outreach to local stroke survivor support networks, presentations to physical therapy groups and use of Cleveland Clinic's 'Stroke Rehabilitation Research Participant Registry '. This registry is a free service open to all persons who have had a stroke so that they may be contacted at a later date for future studies involving stroke research.<br>Strict Inclusion and exclusion criteria were employed in an effort to identify candidates, thus reducing individual bias.
Ethics oversight	Cleveland Clinic Foundation IRB

Note that full information on the approval of the study protocol must also be provided in the manuscript.

Field-specific reporting

Please select the one below that is the best fit for your research. If you are not sure, read the appropriate sections before making your selection.

☒ Life sciences ☐ Behavioural & social sciences ☐ Ecological, evolutionary & environmental sciences

For a reference copy of the document with all sections, see nature.com/documents/nr-reporting-summary-flat.pdf

Life sciences study design

All studies must disclose on these points even when the disclosure is negative.

Sample size	No sample size calculations were made for this study. As an early, Phase I study focused on safety and feasibility of an invasive, neurosurgical approach, the sample size of 12 was established through discussions with the FDA during the IDE application process.
Data exclusions	3 candidates were screen failures. No data from included subjects has been excluded.
Replication	Safety and preliminary efficacy analyses were not subject to replication because this is a phase 1 trial. The primary outcome is safety and all additional analyses are considered exploratory and to be replicated to confirm results in future trials. For brain imaging analyses, model performance was validated using leave-one-out cross validation.
Randomization	This is an open-label, single arm study.
Blinding	This is an open-label, single arm study.

Reporting for specific materials, systems and methods

We require information from authors about some types of materials, experimental systems and methods used in many studies. Here, indicate whether each material, system or method listed is relevant to your study. If you are not sure if a list item applies to your research, read the appropriate section before selecting a response.

Materials & experimental systems

n/a	Involved in the study
☒	☐ Antibodies
☒	☐ Eukaryotic cell lines
☒	☐ Palaeontology and archaeology
☒	☐ Animals and other organisms
☐	☒ Clinical data
☒	☐ Dual use research of concern

Methods

n/a	Involved in the study
☒	☐ ChIP-seq
☒	☐ Flow cytometry
☐	☒ MRI-based neuroimaging

Clinical data

Policy information about clinical studies

All manuscripts should comply with the ICMJE guidelines for publication of clinical research and a completed CONSORT checklist must be included with all submissions.

Clinical trial registration	NCT02835443
Study protocol	A full trial protocol can be found within the supplemental material that were submitted along with the manuscript.
Data collection	Subject recruitment began on June 1, 2016. The final participant appointment for data collection was in November 2022. All patient appointments and data collection was performed at Cleveland Clinic Main Campus in Cleveland, OH.
Outcomes	This study is a Phase 1 designed to collect safety and feasibility data. The primary outcome measure of safety was evaluated by the incidence of adverse and serious adverse events. AEs and SAEs were evaluated by the PI as to their severity, expectedness, and relatedness (to the study intervention and/or participation) . Secondary outcome measures of feasibility evaluated motor impairment and function over the course of the trial by focusing on changes of the Upper Extremity Fugl-Meyer Assessment (FM-UE) across 5 key intervals: 1) Pre- versus post-surgery 2) over the two-month, Rehab-Only baseline period 3) over the experimental, DBS+Rehab phase 4) across the two-month, Rehab carry-over phase in the absence of DBS 5) at long-term follow-up.

Magnetic resonance imaging

Experimental design

Design type	structural MRI
Design specifications	There were no design specifications of MRI protocol relevant for this acquisition.
Behavioral performance measures	There was no specified behavior performed in association with this acquisition.

Acquisition

Imaging type(s)	structural
Field strength	3 Tesla
Sequence & imaging parameters	magnetization prepared rapid gradient echo, 3D, field of view=256 mm x 256 mm, matrix size=256 x 256, slice thickness=1mm, orientation=axial, echo time=2.44 ms, repetition time=2300 ms, inversion time=900 ms, flip angle=8 degrees
Area of acquisition	whole brain

Diffusion MRI ☐ Used ☒ Not used

Preprocessing

Preprocessing software	Brain extraction: antsBrainExtraction, ANTs-2.3.5; Segmentation: FreeSurfer v7.1.1
Normalization	Non-linear registration of positron emission tomography (PET) to MRI: antsRegistration, ANTs-2.3.5; non-linear registration of the Human Motor Area Template to each subject's MRI: antsRegistrationSynQuick, ANTs-2.3.5

Normalization template: non-linear registration of the Human Motor Area Template to each subject's MRI used the MNI152_T1_2009c template

Noise and artifact removal: not performed

Volume censoring: This was a single volume acquisition, so volume censoring is not relevant.

Statistical modeling & inference

Model type and settings: Linear mixed-effects models were used to test for significant change in the mean standardized uptake value ratio (SUVR) of perilesional and ipsilesional, motor-associated, and contralesional occipital (control) cortical regions between Rehab-Only and Rehab Carryover phases of the trial, with time-point and 18F-fluorodeoxyglucose (18F-FDG) uptake time as fixed effects, subject as the random effect intercept. Linear models were used to test for a significant association between change in Arm Motor Ability Test and change in the mean SUVR of perilesional, and ipsilesional motor-associated, and contralesional occipital cortical regions between Rehab-Only and Rehab Carryover phases of the trial, with change in 18F-FDG uptake time as a covariate.

Effect(s) tested: Linear mixed-effects models tested for significant change in the PET mean SUVR of perilesional and ipsilesional, motor-associated, and contralesional occipital (control) cortical regions between Rehab-Only and Rehab Carryover phases of the trial. Linear models tested for a significant association between change in AMAT and change in the mean SUVR of perilesional, and ipsilesional motor-associated, and contralesional occipital cortical regions between Rehab-Only and Rehab Carryover phases of the trial.

Specify type of analysis: ☐ Whole brain ☒ ROI-based ☐ Both

Anatomical location(s): The ipsilesional motor-associated cortical regions were defined using the Human Motor Area Template and consisted of primary motor, primary somatosensory, supplementary motor area (SMA), pre-SMA, dorsal and ventral pre-motor regions. Contralesional occipital cortex was not expected to be affected by dentate stimulation, therefore we defined nine control cortical regions in the occipital lobe from the FreeSurfer segmentation (cuneus gyrus, middle occipital gyrus, superior occipital gyrus, occipital pole, calcarine sulcus, middle occipital and lunatus sulci, superior and transverse occipital sulci, anterior occipital sulcus, occipitotemporal and lateral occipital sulci).

Statistic type for inference (See Eklund et al. 2016): linear mixed-effects models and linear models

Correction: not performed

Models & analysis

n/a	Involved in the study
☒	☐ Functional and/or effective connectivity
☒	☐ Graph analysis
☐	☒ Multivariate modeling or predictive analysis

Multivariate modeling and predictive analysis: Linear mixed-effects models were used to test for significant change in the mean SUVR of perilesional and ipsilesional, motor-associated, and contralesional occipital (control) cortical regions between Rehab-Only and Rehab Carryover phases of the trial, with time-point and 18F-FDG uptake time (i.e., time between 18-FDG injection and start of PET scan) as fixed effects, subject as the random effect intercept, and applying the default unstructured covariance structure. Normality of model residuals and random effect intercept were evaluated using the Shapiro-Wilk normality test. Model fit was evaluated using Bayes Information Criterion and visual examination of residuals. Linear models using change in 18F-FDG uptake time as a covariate were used to test for a significant association between change in AMAT and change in the mean SUVR of perilesional, and ipsilesional motor-associated, and contralesional occipital cortical regions between Rehab-Only and Rehab Carryover phases of the trial. The F-statistic of each model was calculated and model performance was evaluated using leave-one-out cross- validation.
Functional and/or effective connectivity were not calculated.
Graph analysis was not performed.
